# Sequence-regulated, lung-targeting heteropolypeptide nanoparticles via in situ erythrocyte hitchhiking

**DOI:** 10.1038/s41467-026-76188-x

**Published:** 2026-07-30

**Authors:** Ning Li, Jiahe Shen, Yuheng Lei, Junhong Wang, Wei Zhou, Aoting Li, Hui Liu, Shanshan Xiao, Jing Zhao, Shaobo Feng, Guanglin Wang, Lichen Yin, Ziyuan Song

**Affiliations:** 1 Institute of Functional Nano & Soft Materials (FUNSOM), Jiangsu Key Laboratory for Carbon-Based Functional Materials and Devices, Soochow University, Suzhou, China; 2 State Key Laboratory of Radiation Medicine and Protection, School for Radiological and Interdisciplinary Sciences (RAD-X), Collaborative Innovation Center of Radiation Medicine of Jiangsu Higher Education Institutions, Soochow University, Suzhou, China

**Keywords:** Drug delivery, Drug delivery, Polymers, Nanoparticles

## Abstract

Targeted lung delivery of therapeutics is critical for various respiratory diseases. However, the rational design of lung-targeting nanocarriers through fine-tuning of polymeric structures remains challenging. Herein, we reported the development of lung-targeting, heteropolypeptide-grafted nanoparticles (NPs), whose targeting ability was dependent on the copolymer sequence that mediated in situ erythrocyte hitchhiking. Specifically, the incorporation of β-branched amino acid residues in poly(_L_-glutamic acid)s, like valine and isoleucine, resulted in gradient copolymer sequence with terminal hydrophobic segments. The corresponding heteropolypeptide-decorated NPs with hydrophobic coronas thus showed high affinity to red blood cell membranes, leading to accumulation in lung tissues at up to 37% of the injected dose through erythrocyte hitchhiking. This strategy mediated effective lung-targeting of CeO_2_, showing anti-oxidant effect that alleviated pulmonary inflammation to treat acute lung injury. This work highlights the importance of copolymer sequence in tuning the biodistribution of polymer-decorated NPs, shedding light on the design of nanocarriers for pulmonary delivery.

## Abstract (web summary)

Targeted lung delivery of therapeutics is critical for various respiratory diseases. Here, authors develop heteropolypeptide-decorated nanoparticles that exhibit lung targeting through in situ erythrocyte hitchhiking in a sequence-dependent manner, which is further used for the treatment of acute lung injury.

## Introduction

As the central organ of the respiratory system, the lungs play a vital role in mediating the supply of oxygen and the removal of carbon dioxide from the body^1,2^. With the persistent exposure of the lungs to pathogens and airborne pollutants, pulmonary diseases are one of the major global public health challenges, including acute respiratory distress syndrome, asthma, chronic obstructive pulmonary disease, idiopathic pulmonary fibrosis, and lung cancer^3–9^. Numerous efforts have been devoted to developing lung-targeting therapeutics. Through the composition change of lipid nanoparticles, the decoration with antibodies, the change in administration route, and other strategies^10–16^, selective delivery to lung tissues over other organs are achieved, contributing to the enhanced therapeutic efficacy and decreased side effects.

Among various lung-targeting strategies, the red blood cell (RBC) hitchhiking serves as a promising method^12,17–20^. Considering the biocompatibility and prior clinical successes, RBCs are attractive intravascular carriers that overcome various in vivo barriers like mononuclear phagocyte clearance, exhibiting enhanced accumulation of adsorbed nanoparticles (NPs) in the lungs through intravenous (*i.v*.) injection^12,21^. However, the isolation of RBCs from donor blood and the ex vivo handling of RBCs are inevitable in conventional RBC hitchhiking design, which significantly complicates the procedures and increases the risk of contamination. It is therefore of great interest to design NPs with stronger interactions with RBCs, capable of in situ RBC hitchhiking for efficient lung-targeting^22^. In fact, recent studies demonstrated the use of ionic liquid coatings to alter the biodistribution of various NPs through in situ RBC hitchhiking^23,24^.

Previous studies reported that the RBC hitchhiking originated from electrostatic or hydrophobic interactions between NPs and erythrocytes^12,17,25^. Therefore, we reasoned that the introduction of heteropolymers onto the NP surface might allow for the fine-tuning of RBC-NP interactions, as the diverse side-chain design of heteropolymers offers rich non-covalent interactions that mimics natural proteins^26–28^. Specifically, polypeptides from the polymerization of amino acid *N*-carboxyanhydrides (NCAs), as the synthetic analogues of natural proteins, exhibited good biocompatibility and versatile side-chain chemistry^29–35^. With the recent advances in simplifying the preparation of heteropolypeptides, we believe that the screening and optimization of heteropolypeptide compositions would greatly change the affinity between corresponding nanocarriers and RBCs^36,37^, facilitating the pulmonary delivery of therapeutics through in situ RBC hitchhiking mechanism.

**Fig. 1:**
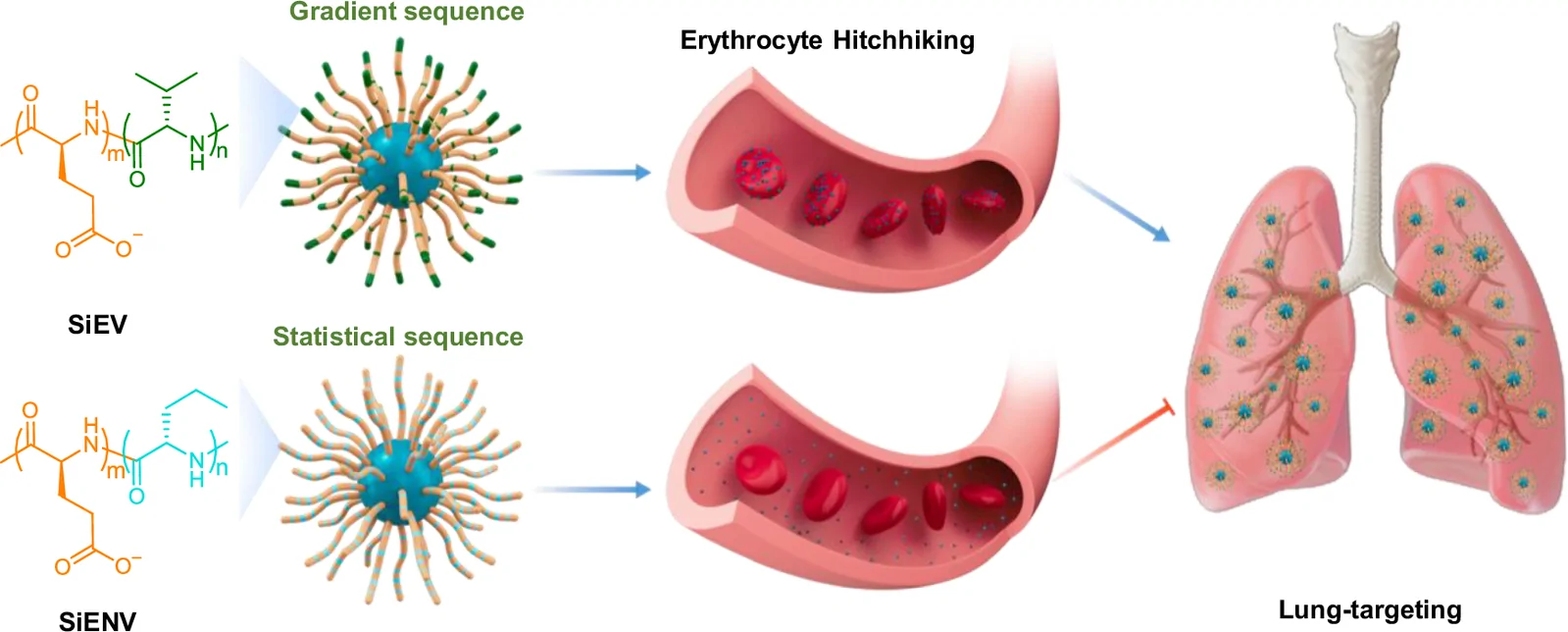
Scheme illustrating the sequence-dependent in situ red blood cell complexation and hitchhiking that mediates selective lung-targeting. The figure was created by Nanjing MyScimage Multimedia Technology Center based on the authors’ conceptual design.

Herein, we reported the development of lung-targeting heteropolypeptide NPs, which enabled efficient pulmonary accumulation (i.e., lung-to-liver ratio up to 14) through in situ erythrocyte hitchhiking in a sequence-regulated manner (Fig. 1). The top-performing NPs, bearing surface-grafted heteropolypeptides with gradient copolymer sequences, mediated effective adsorption onto the membrane of RBCs, facilitating retentions in lungs when RBCs squeezed through the pulmonary capillaries. The heteropolypeptide grafting strategy ensured pulmonary delivery of reactive oxygen species (ROS)-scavenging CeO_2_ for the treatment of acute lung injury (ALI). We believe that this work highlights the crucial role of copolymer sequence in fine-tuning their biological fate, offering effective lung-targeting nanocarriers for the treatment of pulmonary diseases.

## Results and discussion

### Lung-targeting of silica nanoparticles mediated by surface-grafted, valine- or isoleucine-based heteropolypeptides through RBC hitchhiking

**Fig. 2:**
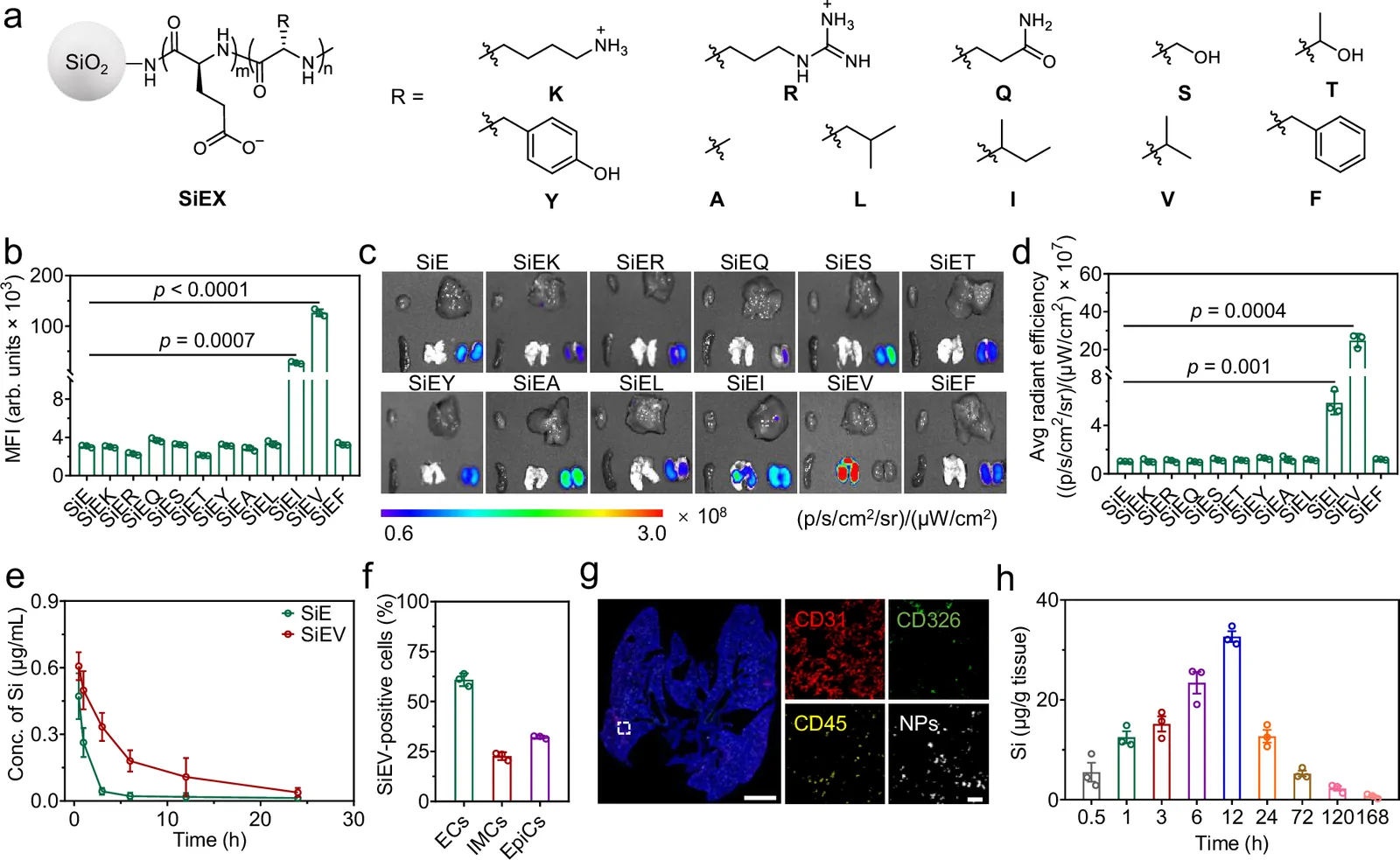
Lung-targeting of silica nanoparticles (NPs) with surface-grafted, valine/isoleucine-based heteropolypeptides via red blood cell (RBC) hitchhiking. **a** Chemical structure of heteropolypeptide-decorated NPs. **b** Quantitative flow cytometric analysis of the mean fluorescent intensity (MFI) of RBCs after incubation with various Cy5-labeled heteropolypeptide-grafted NPs. Data are presented as the mean ± S.D. (*n* = 3 biologically independent samples). **c**, **d** Ex vivo fluorescent images of major organs of mice (**c**) and corresponding semi-quantitative analysis of fluorescent signals in lungs (**d**) at 4 h post intravenous (*i.v*.) injection of various Cy5-labeled heteropolypeptide-grafted NPs. Data are presented as the mean ± S.D. (*n* = 3 female BALB/C mice per group, 6-8 weeks of age). **e** Plasma concentration of Cy5-labeled SiE or SiEV in mice over time as measured by inductively coupled plasma-optical emission spectroscopy (ICP-OES). Data are presented as the mean ± S.D. (*n* = 3 female BALB/C mice per group, 6-8 weeks of age). **f** Flow cytometric analysis of the percentage of SiEV-positive cells in lung tissues at 4 h post *i.v*. injection of Cy5-labeled SiEV. Data are presented as the mean ± S.D. (*n* = 3 female BALB/C mice per group, 6–8 weeks of age). **g** Merged confocal laser scanning microscopy (CLSM) image of pulmonary tissue section (Scale bar = 50 μm) and the corresponding split-channel close-up images (Scale bar = 2 μm). Endothelial cells (ECs, red), immune cells (IMCs, yellow), and epithelial cells (EpiCs, green) were visualized with corresponding dye-labeled antibodies for the analysis of cellular distribution of Cy5-labeled SiEV (white). **h** Quantitative analysis of Si levels at different time points post *i.v*. administration of SiEV by ICP-OES. Data are presented as the mean ± S.D. (*n* = 3 female BALB/C mice per group, 6-8 weeks of age). Statistical analysis for **b** and **d** was calculated via one-way ANOVA with Dunnett’s multiple comparisons test. Source data are provided as a Source Data file.

To examine our hypothesis that the modulation of heteropolypeptide composition could change the interactions with erythrocytes for in situ RBC hitchhiking, we designed a library of heteropolypeptide-modified silica NPs by copolymerizing glutamic acid (E)-derived NCA monomers, γ-*tert*-butyl-_L_-glutamate NCA (*t*Bu-Glu NCA), with other NCA monomers bearing different side-chain structures in a 9:1 molar ratio through surface-initiated polymerization (Supplementary Fig. 1)^38^. After side-chain deprotection, the negative charges of glutamic acid residues guaranteed good water solubility of the NPs, while minimizing the toxicity and protein absorption compared to cationic NPs^39^. The resulting silica NPs were referred to as SiEX, where X stands for the one-letter code of the 10 mol% other amino acid residues (Fig. 2a). Silica NPs decorated with homopolypeptide poly(_L_-glutamic acid) (PLG), on the other hand, was used as control NPs and referred to as SiE (Supplementary Fig. 2). ^1^H nuclear magnetic resonance (NMR) results suggested that the obtained heteropolypeptide composition was consistent with the theoretical values calculated from feeding ratios (i.e., 9:1) (Supplementary Table 1). Moreover, the sizes of all NPs ranged from 35–40 nm, with zeta potentials between -15 and -26 mV (Supplementary Table 1 and Supplementary Fig. 3), suggesting the successful grafting of anionic polypeptides onto the NP surface and minimizing the impact of differences in sizes or charges on later RBC adhesion and biodistribution studies. Meanwhile, transmission electron microscopy (TEM) characterization revealed the spherical morphology of the heteropolypeptide-decorated NPs, with sizes of ~35 nm that agreed well with the dynamic light scattering (DLS) results (Supplementary Fig. 3). Additionally, gel permeation chromatography (GPC) results indicated well-controlled surface-initiated polymerization process that generated monomodal polypeptides with a low dispersity (DP = 680, Supplementary Fig. 3).

Unlike the traditional RBC hitchhiking strategy, we developed a streamlined approach to achieve lung-targeting via the direct *i.v*. injection of NPs capable of in situ association with circulating RBCs, rather than pre-incubation of NPs and RBCs outside the body. Hemolysis studies revealed negligible RBC membrane disruption or hemoglobin release in the presence of NPs (Supplementary Fig. 4). The results of hemagglutination assay also suggested favorable hemocompatibility of heteropolypeptide-modified NPs, where the non-aggregated RBCs settled as a tight dot rather than diffuse haze (Supplementary Fig. 4). Additionally, the plasma level of hemoglobin remained low after *i.v*. injection of the NPs (Supplementary Fig. 4), validating their in vivo hemocompatibility. As a critical first step, cyanine 5 (Cy5)-labeled SiE and SiEX with similar sizes and surface charges but different heteropolypeptide compositions were screened to identify candidates with strong RBC affinity in vitro. While most SiEX NPs showed similar binding with RBCs compared to SiE after 1-h in vitro incubation, RBCs treated with SiEV and SiEI exhibited markedly stronger fluorescence signals than others (Fig. 2b). The mean fluorescent intensity (MFI) of SiEV- and SiEI-treated RBCs was 43- and 27-fold higher than that of SiE, respectively (Fig. 2b), highlighting the critical role of specific polypeptide compositions in mediating RBC affinity.

We next investigated whether the distinct RBC binding properties of heteropolypeptide-modified NPs would translate into enhanced lung-targeting in vivo. The SiE and SiEX NPs were intravenously injected into mice, whose enrichment in various major organs was visualized at 4 h post administration. While most NPs exhibited accumulation in kidney that was presumably attributed to their relatively small sizes, SiEV and SiEI NPs showed significantly higher fluorescent signals in lungs than all other SiEX NPs (Fig. 2c and Supplementary Fig. 5). Specifically, semi-quantification of Cy5 signals suggested that the lung accumulation of top-performing SiEV, with surface-grafted PLG-*co*-poly(_L_-valine) (PEV), was 25-fold higher than that of SiE (Fig. 2d). Additionally, confocal laser scanning microscopy (CLSM) analysis revealed overwhelming Cy5 fluorescence all over the pulmonary tissues of SiEV-treated mice, in sharp contrast to the negligible fluorescent intensity from their SiE analogues that substantiated the lung-targeting behaviors of SiEV (Supplementary Fig. 6). To verify that the lung-targeting of SiEV was mediated by in situ RBC hitchhiking, blood samples were collected for visualization at 1 h after *i.v*. injection of SiEV. As expected, the RBCs exhibited much stronger Cy5 fluorescence than other blood cells (Supplementary Fig. 7), confirming the in vivo complexation of SiEV with RBCs that mediated effective lung-targeting. On the other hand, the low uptake by leukocytes minimized the triggering of immune responses that validated the biocompatibility of SiEV. Notably, the stability of SiEV in plasma ruled out any significant size changes during circulation (Supplementary Fig. 8), thereby excluding the possibility of passive lung-targeting through mechanical filtration^40^. Additionally, pharmacokinetic analysis by inductively coupled plasma-optical emission spectroscopy (ICP-OES) revealed that SiEV, unlike SiE that was cleared within 3 h, exhibited prolonged blood circulation up to 12 h (i.e., 0.11 µg/mL in plasma) (Fig. 2e). The extended circulation of SiEV was attributable to its capacity of in situ RBC hitchhiking following *i.v*. injection, which enabled effective NP delivery to the lung tissues.

To further evaluate the NP delivery at the cellular levels, lung tissues of SiEV-treated mice were labeled with various markers and subjected to flow cytometric analysis. As illustrated in Fig. 2f, ~60% of all endothelial cells (ECs), ~20% of all immune cells (IMCs), and ~30% of all epithelial cells (EpiCs) were Cy5-positive. Indeed, CLSM images revealed efficient colocalization of SiEV NPs with the ECs (Fig. 2g), which was consistent with the flow cytometric results. By visualizing the lung tissues at different time points post injection, the fluorescent intensity of SiEV was the highest at 12 h after the administration (Supplementary Fig. 9). Additionally, the pulmonary concentration of Si was quantified by ICE-OES (Fig. 2h), which revealed ~4.9 μg of Si in the entire lung tissue (i.e., 32.7 μg of Si per g of the tissue) at 12 h post injection, corresponding to 37% of the injected dose (ID). Moreover, the pulmonary level of SiEV was also confirmed by fluorescent analysis of tissue homogenates or radioisotope tracing, which was consistent with the ICP-OES results that the pulmonary accumulation of SiEV reached a peak at 12 h post injection (Supplementary Figs. 9, 10). In fact, semi-quantitative ex vivo fluorescent imaging suggested that SiEV exhibited enhanced lung-targeting than Si^PECAM1^, the silica NPs decorated with the antibody of one of the most common receptors in pulmonary ECs, PECAM1 (Supplementary Fig. 11)^41–43^. SiEV thus served as a promising alternative choice to antibody-decorated, lung-targeting NPs. Finally, the Si signal gradually decreased and became nearly undetectable on 7 d after administration, suggesting the gradual clearance of the NPs from the body without long-term safety concerns (Supplementary Fig. 9).

### Important role of heteropolypeptide sequence in erythrocyte hitchhiking and pulmonary accumulation

**Fig. 3:**
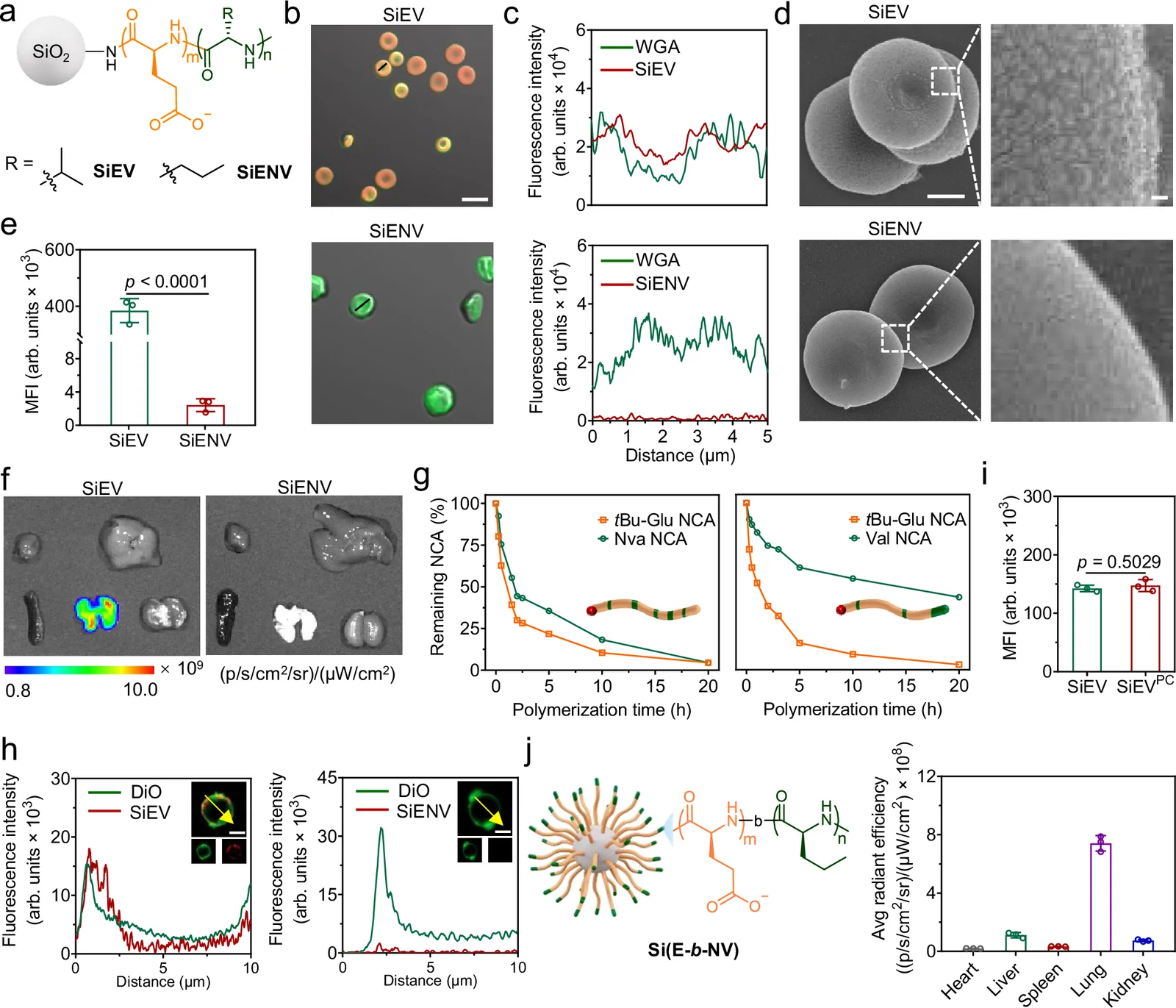
Structure-property relationship study of SiEV for red blood cell (RBC) hitchhiking. **a** Chemical structure of SiEV and SiENV. **b, c** Merged confocal laser scanning microscopy (CLSM) images (**b**) and corresponding co-localization analysis (**c**) of FITC-labeled RBCs (green) after in vivo complexation with Cy5-labeled SiEV or SiNEV (red). Scale bar = 10 μm. The colocalization analysis was performed along the selected direction (black) of a randomly picked RBC. **d** Scanning electron microscopy (SEM) images of RBCs after in vivo complexation with SiEV or SiENV. Scale bar = 2 μm (left) or 50 nm (right). **e** Quantitative flow cytometric analysis of the mean fluorescent intensity (MFI) of RBCs after in vivo complexation with Cy5-labeled SiEV or SiENV. Data are presented as the mean ± S.D. (*n* = 3 female BALB/C mice per group, 6–8 weeks of age). **f** Ex vivo fluorescent images of major organs of mice at 12 h post intravenous (*i.v*.) injection of Cy5-labeled SiEV and SiENV. **g** Copolymerization kinetics of *t*Bu-Glu NCA/Nva NCA (left) or *t*Bu-Glu NCA/Val NCA (right) at 9:1 molar ratio in anhydrous cosolvent as monitored by NMR. [M]_0_ = 0.4 M, [M]_0_/[I]_0_ = 50. The schemes illustrated the copolymer sequence structure, with the red spheres representing the C termini. **h** CLSM images (insets) and corresponding co-localization analysis (yellow arrow) of DiO-labeled giant unilamellar vesicle (GUV) (green) after incubation with Cy5-labeled SiEV or SiNEV (red). Scale bar = 10 μm. **i** Quantitative flow cytometric analysis of the MFI of RBCs after incubation with Cy5-labeled SiEV or SiEV^PC^. Data are presented as the mean ± S.D. (*n* = 3 biologically independent samples). **j** Schematic illustration, chemical structure, and semi-quantitative analysis of accumulation in major organs of Si(E-*b*-NV) decorated with block copolypeptides. Data are presented as the mean ± S.D. (*n* = 3 female BALB/C mice per group, 6-8 weeks of age). Statistical analysis for (**e**, **i**) was calculated with a two-tailed unpaired *t*-test. The scheme in (**j**) was created by Nanjing MyScimage Multimedia Technology Center based on the authors’ conceptual design. Source data are provided as a Source Data file.

The notable RBC hitchhiking ability of SiEV and SiEI NPs encouraged us to further study the structure-property relationship. Considering the side chain of valine (i.e., isopropyl groups) and isoleucine (i.e., isobutyl groups) residues, we reasoned that the specific β-branched structure of hydrophobic amino acids was crucial for the RBC complexation of the corresponding NPs. Indeed, previous screening results suggested negligible RBC adsorption of SiEA (i.e., non-branched side chains) and SiEL (i.e., γ-branched side chains) NPs. Therefore, another heteropolypeptide-decorated silica NP, SiENV, was designed and prepared to validate our hypothesis, which had 10 mol% non-natural amino acid, norvaline, that was an isomer of valine with a non-branched side-chain structure (i.e., *n*-propyl groups) (Fig. 3a). In an attempt to evaluate the in situ complexation between heteropolypeptide-decorated NPs and RBCs, Cy5-labeled SiEV or SiENV was intravenously injected into mice, whose blood was collected at 1 h post injection for RBC visualization. As expected, RBCs from SiEV-treated mice exhibited much stronger Cy5 fluorescence than their SiENV-treated analogues (Fig. 3b), substantiating the critical role of β-branched amino acids in mediating RBC hitchhiking of the heteropolypeptide-decorated NPs. Further line scan analysis suggested good overlap between fluorescence of SiEV and RBC membranes (Fig. 3c), the latter stained with fluorescein isothiocyanate (FITC)-labeled wheat germ agglutinin (WGA) that validated the surface adsorption of SiEV rather than cellular internalization. Meanwhile, scanning electron microscopy (SEM) further confirmed the rough surfaces of RBCs from SiEV-treated mice that were attributed to the adherence of NPs, in stark contrast to the smooth RBC surface after SiENV injection (Fig. 3d). Further energy-dispersive X-ray spectroscopy (EDS) analysis confirmed the presence of Si-based NPs on the surface of RBCs (Supplementary Fig. 12). Additionally, flow cytometric analysis indicated that 10.1% and 0.9% of RBCs was Cy5-positive after the *i.v*. injection of fluorescently-labeled SiEV and SiENV, respectively (Supplementary Fig. 13). The quantitative analysis revealed 2 orders of magnitude higher MFI of RBCs after SiEV administration compared to their SiENV analogues (Fig. 3e). Notably, the biodistribution of SiENV NPs resembled that of SiEA and SiEL, which exhibited low targeting efficiency to any major organs at 12 h post *i.v*. injection (Fig. 3f), substantiating the critical role of β-branched amino acids in mediating the lung-targeting of the heteropolypeptide-decorated NPs. At the same time, the internalization study by immortalized mouse pulmonary microvascular endothelial cells (iMPMVECs) revealed similar uptake of SiEV and SiENV (Supplementary Fig. 14), ruling out the possibility that the different lung-targeting of the two NPs originated from specific interactions with markers in lung tissues. In addition, the adsorption of SiEV onto RBC surface was further studied under both static, in vitro conditions and dynamic, in vivo conditions. The results showed the rapid adsorption of SiEV after ~10 min incubation, with more NPs adsorbed as the increase in incubation time (Supplementary Fig. 15). After 1-h static incubation with the whole blood, while only 4.8% of SiENV bound to RBCs, 71.7% of SiEV was associated with RBCs, corresponding to ~5700 SiEV adsorbed onto the RBC surface (Supplementary Fig. 15), indicating strong RBC-SiEV interactions even in the presence of other circulating blood cells. Meanwhile, the adsorption became obvious at 30 min post injection, suggesting slower adsorption kinetics in vivo that was attributed to the dynamic blood flow and complex physiological environment (Supplementary Fig. 15).

Considering the close proximity between side-chain β-branches and backbone, the polymerization of monomers based on β-branched amino acids was likely decelerated due to the steric hindrance^44^, thus altering the heteropolypeptide sequence and eventually influencing the NP fate. The copolymerization kinetics was therefore monitored in situ to characterize the heteropolypeptide sequence of SiEV and SiENV. As shown in Fig. 3g, the consumption rate of _L_-norvaline NCA (Nva NCA) and *t*Bu-Glu NCA was comparable throughout the copolymerization progress, generating heteropolypeptides with statistical sequential distributions. In sharp contrast, _L_-valine NCA (Val NCA) was consumed in a much slower manner during the copolymerization, which was attributed to the steric effect of the β-branched side-chain structure. As a result, there was ~50% Val NCA remaining after the full conversion of *t*Bu-Glu NCA monomers, yielding heteropolypeptides with a gradient or even block-like sequence (Fig. 3g). Therefore, hydrophobic poly(_L_-valine) (PVal) segments were presented at the outer layers of SiEV, which showed a significant impact on the NP biodistribution.

While the existence of a hydrophobic outer layer of a NP in aqueous solution was disfavored, NMR study suggested rather good solvation of the hydrophobic segments of SiEV in D_2_O at 10 mol% valine (Supplementary Fig. 16). Further increase in valine proportion to 20 mol%, nevertheless, led to the decrease in valine signals that suggested the assembly or folding of the hydrophobic segments to avoid excessive solvent exposure. Once injected into the blood vessels, the complexation of SiEV with RBC likely originated from the insertion of the hydrophobic segments into the bilayer of RBC membrane like lipid tethers^45,46^. To further support our hypothesis, giant unilamellar vesicles (GUVs) were prepared, which served as a synthetic analogue of RBC with only lipids on the membrane^47^. The pronounced co-localization of SiEV with the GUV membrane (Fig. 3h), in stark contrast to SiENV, provided visual evidence that the exposed hydrophobic segments drove the complexation with lipid membranes. Meanwhile, the exposed hydrophobic segments may also result in the adsorption of serum proteins. To check the impact of protein corona, SiEV was first pre-treated with plasma for the formation of protein corona, which was evidenced by the increase in size and change in zeta potential (Supplementary Fig. 17). Interestingly, the resulting protein-coated SiEV^PC^, with the adsorption of 37 μg protein/mg NPs, exhibited similar RBC affinity compared to bare SiEV (Fig. 3i). Therefore, even with the pre-formed protein corona, SiEV still complexed with the erythrocytes, suggesting robust lung-targeting driven by the hydrophobic interactions between hydrophobic segments and membrane bilayers. It has to be noted that the RBC-SiEV complexation might involve multiple non-covalent forces beyond the hydrophobic interactions between PVal segments and lipid bilayers^19^. While the specific receptor-mediated association was not observed at the moment (Supplementary Fig. 18), the Coulomb interactions and the hydrogen bonding interactions may further facilitate the stable anchoring of SiEV onto RBC membranes and contribute to the overall binding affinity.

To further validate the sequence-dependent RBC complexation and subsequent lung-targeting ability, a block copolypeptide analogue of SiENV, Si(E-*b*-NV), was prepared through sequential addition of monomers, which showed obvious accumulation in lung tissues (Fig. 3j). Meanwhile, the block copolymerization strategy was also used to boost the pulmonary enrichment of the analogues of SiEL and SiEF, referred to as Si(E-*b*-L) and Si(E-*b*-F) (Supplementary Fig. 19), demonstrating the general lung-targeting design of heteropolypeptide-modified NPs by introducing hydrophobic outer layers.

### In-depth studies on the erythrocyte-SiEV complexes

**Fig. 4:**
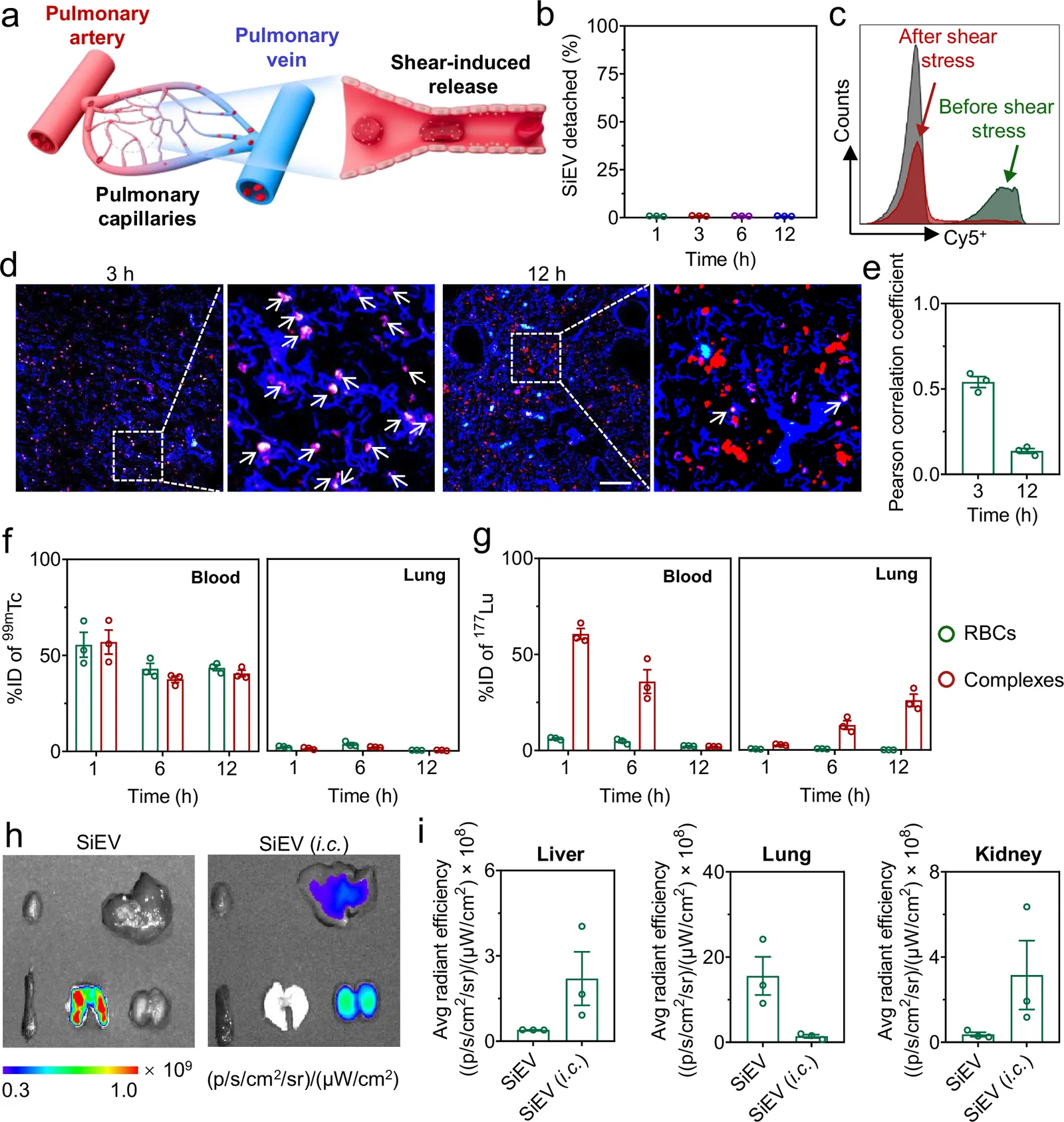
Shear stress-mediated detachment of SiEV from red blood cells (RBCs) for pulmonary accumulation. **a** Scheme illustrating the RBC hitchhiking of SiEV for lung-targeting. **b** The stability analysis of the RBC-SiEV complexes at different time points under gentle shaking. Data are presented as the mean ± S.D. (*n* = 3 biologically independent experiments). **c** Flow cytometric analysis of Cy5-positive RBCs before and after the introduction of shear stress to RBC-SiEV complexes. **d, e** Merged confocal laser scanning microscopy (CLSM) images of pulmonary tissue section (**d**) and corresponding co-localization analysis of SiEV (red) and RBCs (green) (**e**) at 3 h or 12 h post intravenous (*i.v*.) injection of RBC-SiEV complexes. The white arrows represent the co-localization of SiEV and RBCs. Scale bar = 200 μm. Data are presented as the mean ± S.D. (*n* = 3 female BALB/C mice per group, 6-8 weeks of age). **f, g** Radioisotope tracing of ^99m^Tc-labeled RBCs (**f**) or ^177^Lu-labeled SiEV (**g**) in blood and lung tissues at different time points post *i.v*. injection of RBC-SiEV complexes. Data are presented as the mean ± S.D. (*n* = 3 female BALB/C mice per group, 6-8 weeks of age). **h** Ex vivo fluorescent images of major organs of mice at 12 h post *i.v*. or intracardiac (*i.c*.) injection of Cy5-labeled SiEV. **i** Semi-quantitative analysis of liver, lung, and kidney in (**h**). Data are presented as the mean ± S.D. (*n* = 3 female BALB/C mice per group, 6-8 weeks of age). The scheme in **a** was created by Nanjing MyScimage Multimedia Technology Center based on the authors’ conceptual design. Source data are provided as a Source Data file.

With the hydrophobic segments of SiEV mediating efficient complexation with RBCs, the pulmonary accumulation process of heteropolypeptide-decorated NPs resembled the reported RBC hitchhiking NPs^12^, the difference being that SiEV interacts with the circulating erythrocytes in situ rather than through pre-incubation. Briefly, the SiEV adsorbed onto the erythrocyte surface upon *i.v*. injection, which was transferred to ECs in lung tissues when RBCs squeeze through pulmonary capillaries (Fig. 4a). Since RBC is slightly larger than the average diameter of pulmonary capillaries, the friction forces from endothelial wall and the cell-cell interactions likely contribute to the desorption process. While the quantification of the shear forces requires further studies, the moderate interactions between SiEV and RBC guarantees the dissociation in capillaries while maintaining the complex stability under normal blood flow conditions. To evaluate the impact of shear stress on the stability of complexes, the suspension of erythrocyte-SiEV was first placed on a shaker with gentle agitation (i.e., 100 rpm) to simulate the hemodynamic shear stress during normal circulation^48^. As shown in Fig. 4b, the RBC-SiEV complexes were quite stable under circulation-mimetic conditions, with negligible desorbed SiEV in the supernatant ( < 1%) after 12-h incubation. As a result, the off-target effect resulting from the premature release of SiEV from the complexes was minimized. In sharp contrast, a considerable amount of SiEV fell off from RBC when the complexes were loaded in a syringe and the shear stress was applied through manual extrusion^49^. Flow cytometric analysis revealed significantly decreased Cy5 signals of the RBCs with stronger shear stress that mimics the conditions in pulmonary capillaries (Fig. 4c).

To further investigate the biofate of erythrocyte-SiEV in lung tissue, the complexes were pre-formed in vitro with FITC-labeled RBCs (referred to as ^FITC^RBC) and Cy5-labeled SiEV, which were intravenously injected into the mice. CLSM characterization revealed that the RBC-SiEV complexes were still observed with good co-localization (Pearson coefficient ~0.54) at 3 h after *i.v*. injection. However, negligible FITC signal of RBCs was detected in lung tissues with low fluorescent co-localization (Pearson coefficient ~0.13) at 12 h post injection, suggesting the translocation of SiEV from the complexes to pulmonary tissues (Fig. 4d, e). Additionally, the percentage of FITC-positive RBCs in plasma were maintained at a constant level within 12 h, with negligible FITC signals observed in the lung tissues that resembled that from mice receiving only ^FITC^RBC injection (Supplementary Fig. 20). In stark contrast, the Cy5-positive cells exhibited a gradual decrease in plasma over time, coinciding with the increase in fluorescent signals in lung tissues (Supplementary Fig. 20). Meanwhile, different radioisotopes (i.e., ^99m^Tc and ^177^Lu) were used to separately label RBCs and SiEV in the complexes, which provided quantitative desorption results. While most ^99m^Tc-labeled RBCs were constantly found in circulating plasma, ^177^Lu-labeled SiEV gradually transferred from the blood to the lung tissues (Fig. 4f, g). Specifically, the blood level of SiEV decreased from 60.3% ID to 1.5% ID from 1 h to 12 h post injection. The pulmonary concentration of SiEV, on the other hand, increased from 2.8% ID to 25.3% ID. These results excluded the possibility that the erythrocyte-SiEV complexes were entirely trapped in lung tissues or internalized by ECs. In other words, the RBCs served as carriers, which stayed in the blood flow to deliver SiEV into lung tissues.

Both the formation of erythrocyte-SiEV complexes and the *i.v*. injection were critical for the lung-targeting of SiEV, as the pulmonary capillaries is the first capillary bed encountered by the complexes administered through veins, which mediated efficient translocation of NPs to ECs. Therefore, the change in injection route would likely alter the biodistribution of the NPs, especially when pulmonary capillaries is not the only downstream capillary bed^12^. To verify our hypothesis, Cy5-labeled SiEV was administered through intracardiac (*i.c*.) injection, where multiple downstream capillaries exist. The Cy5 fluorescent signals were observed in several organs including lungs, livers, and kidneys (Fig. 4h, i), substantiating that the lung-targeting of SiEV through *i.v*. injection was not correlated to specific receptors like antibodies. As expected, the pre-treatment of iMPMVECs with anti-PECAM1 exhibited negligible impact on the internalization of SiEV, but significantly blocked the cellular entrance of Si^PECAM1^ (Supplementary Fig. 21). In summary, the in situ RBC hitchhiking of SiEV to lung tissues requires the *i.v*. administration route, the interactions between erythrocytes and NPs to form complexes, as well as sufficient shear forces in pulmonary capillaries to unload the NPs.

### The effect of structural parameters of nanoparticles on lung-targeting

Motivated by the β-branched amino acid-mediated, sequence-dependent lung-targeting behavior of SiEV through RBC hitchhiking, we moved on to evaluate the impact of other structural parameters of NPs on the lung-targeting, aiming to optimize the targeting ability. Specifically, SiEV with different sizes and valine percentages were prepared, whose biodistribution was evaluated. The grafting of heteropolypeptides resulted in the increase of sizes compared to the initial silica NPs, with the final sizes ranging from 33 to 96 nm. Both TEM and DLS analyses confirmed the well-defined and uniform NPs with different sizes, which showed negative surface charges ~ -25 mV (Supplementary Figs. 22, 23, and Supplementary Table 2). As shown in Supplementary Fig. 24, smaller SiEV exhibited obvious accumulation in lung at 12 h post *i.v*. injection. As the size increased, significant liver-targeting behavior was observed. Through fluorescent quantification after tissue homogenization, filtration, and RBC lysis, the top-performing SiEV, with an initial silica NP size of 15 nm and a final size of 41 nm after heteropolypeptide decoration, exhibited the highest lung-to-liver ratio of 14 at 12 h post *i.v*. administration (Supplementary Fig. 24). As the size of SiEV approaching 70 nm, the lung-targeting ability rapidly diminished, with comparable Cy5 signals in lung and liver (Supplementary Fig. 24), suggesting the critical role of NP size in biodistribution. On the other hand, increasing valine percentage led to better lung-targeting that was attributed to the sufficiently long PVal blocks for efficient RBC interactions (Supplementary Fig. 25 and Supplementary Table 3). Notably, the incorporation of 5 mol% of valine residues was sufficient to show acceptable lung-targeting efficiency, corroborating the important role of β-branched amino acid in mediating pulmonary accumulations.

Besides silica NPs, the heteropolypeptide decoration strategy was also extended to other inorganic NPs to render them lung-targeting, including ferric oxides (Fe_3_O_4_) and quantum dots (QDs). Compared to the amine-functionalized cores, the grafting of heteropolypeptides led to significant accumulation in lung tissues (Supplementary Fig. 26). It has to be noted that the pulmonary accumulation of PEV-decorated quantum dots excluded the possibility that the previous Cy5 signals in lung tissues originated from degraded heteropolypeptides, further supporting the lung-targeting of the NPs. Meanwhile, linear heteropolypeptide (i.e., PEV) exhibited poor lung-targeting behaviors (Supplementary Fig. 27). We believe that the self-assembly of the linear polymer buried the hydrophobic PVal segments, failing to mediate efficient RBC interactions for pulmonary accumulations.

### Delivery of ceric oxide to pulmonary effectively alleviated acute lung injury

**Fig. 5:**
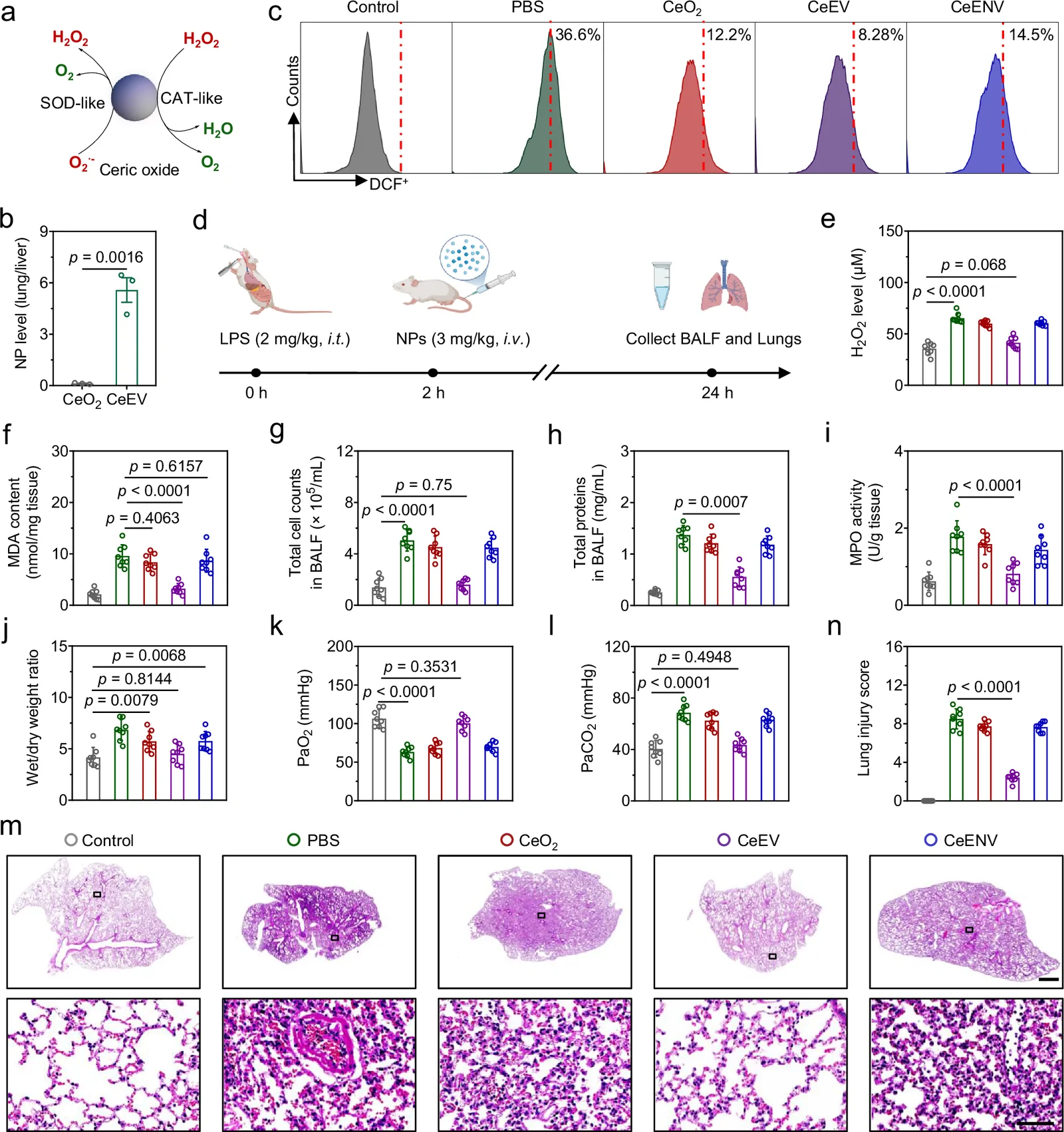
CeEV-mediated alleviation of oxidative stress and recovery of pulmonary functions in mice with acute lung injury (ALI). **a** Schematic presentation of enzyme-like activities of CeO_2_. **b** Quantitative analysis of lung-to-liver ratio of CeO_2_ and CeEV by analyzing the fluorescent intensity in the lung tissue homogenate. Data are presented as the mean ± S.D. (*n* = 3 female BALB/C mice per group, 6-8 weeks of age). **c** Flow cytometric results of RAW 264.7 cells for the determination of percentage of reactive oxygen species (ROS)-positive cells after incubation with different nanoparticles (NPs). The red dashed lines are gates to differentiate ROS-negative and ROS-positive cells. **d** Scheme of lipopolysaccharide (LPS)-mediated ALI modeling and treatment experiments. **e-l** Hydrogen peroxide (H_2_O_2_) levels in lung tissues (**e**), malondialdehyde (MDA) levels in lung tissues (**f**), total cell counts in bronchoalveolar lavage fluid (BALF) (**g**), total proteins in BALF (**h**), myeloperoxidase (MPO) levels in lung tissues (**i**), wet/dry ratios of lung tissues (**j**), partial pressure of oxygen (PaO_2_) (**k**), and partial pressure of carbon dioxide (PaCO_2_) (**l**) of arterial blood of ALI mice after intravenous (*i.v*.) injection of PBS, CeO_2_, CeEV, or CeENV (3 mg/kg). Data are presented as the mean ± S.D. (*n* = 8 male BALB/C mice per group, 6-8 weeks of age). **m** Representative hematoxylin-eosin staining of lung sections of ALI mice after treatment with PBS, CeO_2_, CeEV, or CeENV (3 mg/kg). Scale bar = 1 mm (top row) or 50 μm (bottom row). **n** Clinical lung injury scores after *i.v*. injection of PBS, CeO_2_, CeEV, or CeENV (3 mg/kg). Data are presented as the mean ± S.D. (*n* = 8 male BALB/C mice per group, 6-8 weeks of age). Normal mice without LPS challenge or NP treatment served as the control. Statistical analysis in (**b**, **h**, **i**, and **n**) was calculated using a two-tailed unpaired *t*-test, and others were calculated using one-way ANOVA with Dunnett’s multiple comparisons test. Scheme in (**a**) was created with Microsoft PowerPoint, scheme in **d** was created in BioRender. Wu, F. (https://BioRender.com/p9fw4qf). Source data are provided as a Source Data file.

Encouraged by the universal strategy of heteropolypeptide grafting to mediate effective lung-targeting, we further explored the therapeutic potential of NPs decorated with valine-based heteropolypeptides. CeO_2_ was selected as the core, which has been reported to exhibit multiple enzyme-like properties for the clearance of ROS. Specifically, CeO_2_ catalyzed the transformation of superoxide anion (O_2_.^-^) and hydrogen peroxide (H_2_O_2_) in a superoxide dismutase (SOD)- and catalase (CAT)-like manner^50,51^, respectively, yielding oxygen and water (Fig. 5a). Amine-functionalized CeO_2_ was therefore used to initiate the copolymerization, yielding CeEV and CeENV. Both NPs exhibited negligible toxicity to the cells up to 100 μg/mL (Supplementary Fig. 28), confirming their biocompatibility as lung-targeting nanocarriers. As expected, the modification of PEV mediated effective lung-targeting of CeEV (final size of ~28 nm), whose lung-to-liver ratio was determined to be 5.6, 51-fold higher than that of CeO_2_ (Fig. 5b and Supplementary Fig. 29). Meanwhile, the modification of heteropolypeptides did not significantly alter the catalytic activity compared to bare CeO_2_ (Supplementary Fig. 30), leading to effective clearance of ROS for anti-inflammatory effects in vitro. The ROS-positive RAW 264.7 cells, induced by lipopolysaccharide (LPS), accounted for 12.2%, 8.28%, and 14.5% in the presence of CeO_2_, CeEV, and CeENV, respectively, in sharp contrast to 36.6% cells without any treatment (Fig. 5c). Additionally, all three NPs reduced the production of inflammatory factors in cells, including tumor necrosis factor-α (TNF-α), interleukin-6 (IL-6), and interleukin-1β (IL-1β)^52^, substantiating their anti-inflammatory effect (Supplementary Fig. 31).

After confirming the anti-oxidative effect of CeEV in vitro, we continued to explore its effect for the treatment of ALI. LPS was first administered through intratracheal (*i.t*.) injection, followed by the *i.v*. injection of different NPs after 2 h^53,54^. The bronchoalveolar lavage fluid (BALF) and lung tissues were then collected and analyzed at 24 h post *i.t.* injection of LPS (Fig. 5d). Since the generation of excessive ROS was considered as the primary trigger of ALI^55^, the oxidative stress in lung tissues was first evaluated after LPS challenge. The H_2_O_2_ level of PBS-treated ALI mice was 1.9 times higher than that of healthy control mice (Fig. 5e), confirming the upregulation of ROS level in ALI mice. The administration of CeO_2_ and CeENV, with poor accumulation in lung tissues, did not significantly attenuate the ROS generation (Fig. 5e). In sharp contrast, the injection of lung-targeting CeEV effectively consumed the pulmonary ROS, bringing H_2_O_2_ level to normal values (i.e., 35.5 μM and 41.4 μM for healthy control and CeEV-treated ALI mice, respectively). Moreover, CeEV treatment greatly decreased the level of malondialdehyde (MDA), a product of lipid peroxidation, by 67% compared to that of PBS-treated of ALI mice (Fig. 5f), outperforming its CeO_2_ and CeENV analogues even though the three NPs exhibited similar anti-oxidative effect in vitro (Fig. 5c).

The LPS challenge resulted in over-recruitment and overactivation of immune cells in lung tissues, leading to tissue edema and increased vascular permeability^56^. As expected, ALI mice treated with PBS, CeO_2_, and CeENV showed significantly increased total number of cells in BALF, which was 3.6, 3.3, and 3.2 times higher than that of the control group, respectively (Fig. 5g). The analysis of total proteins in BALF was also much higher than that of normal mice (Fig. 5h), substantiating the compromised integrity of alveolar-capillary barrier. However, with the treatment of lung-targeting CeEV, the BALF cells and proteins returned to normal levels (Fig. 5g, h), suggesting the alleviation of LPS-induced inflammation. Additionally, the administration of CeEV led to the decrease in the level of myeloperoxidase (MPO), an important marker of ALI, by 54% compared to that with PBS-treatment (Fig. 5i), suggesting the inhibited neutrophil infiltration into lung parenchyma or alveolar spaces that ameliorated the inflammatory cascade.

CeEV-mediated anti-oxidative effect in lung tissues resulted in the ameliorated ALI pathology and recovered pulmonary function. The wet/dry weight ratio of lung tissues after the treatment of CeO_2_, CeEV, and CeENV was 5.7, 4.5, and 5.8, respectively (Fig. 5j), indicating the effectively alleviated pulmonary edema mediated by lung-targeting CeEV. Subsequently, the pulmonary function of ALI mice was assessed by measuring partial pressure of oxygen (PaO_2_) and partial pressure of carbon dioxide (PaCO_2_) in the arterial blood. In contrast to the decreased PaO_2_ and increased PaCO_2_ observed in ALI mice, both PaO_2_ and PaCO_2_ were restored to normal levels after treatment with CeEV, outperforming its analogues due to the accumulation in lung tissues (Fig. 5k, l). In support of this observation, hematoxylin-eosin (H&E) staining of lung tissues further revealed that intervention with CeEV ameliorated the pathological symptoms of ALI, including thickening of alveolar wall, hemorrhage in the alveolar and interstitial areas, and infiltration of inflammatory cells that were obvious in other treatment groups (Fig. 5m). While ineffective treatments by PBS, CeO_2_, and CeENV resulted in high clinical scores of lung injury up to 8.5, CeEV reduced the pulmonary damage and promoted the lung function recovery that the injury score was only 2.8 (Fig. 5n).

### CeEV NPs substantially reduced inflammation in diseased mice

**Fig. 6:**
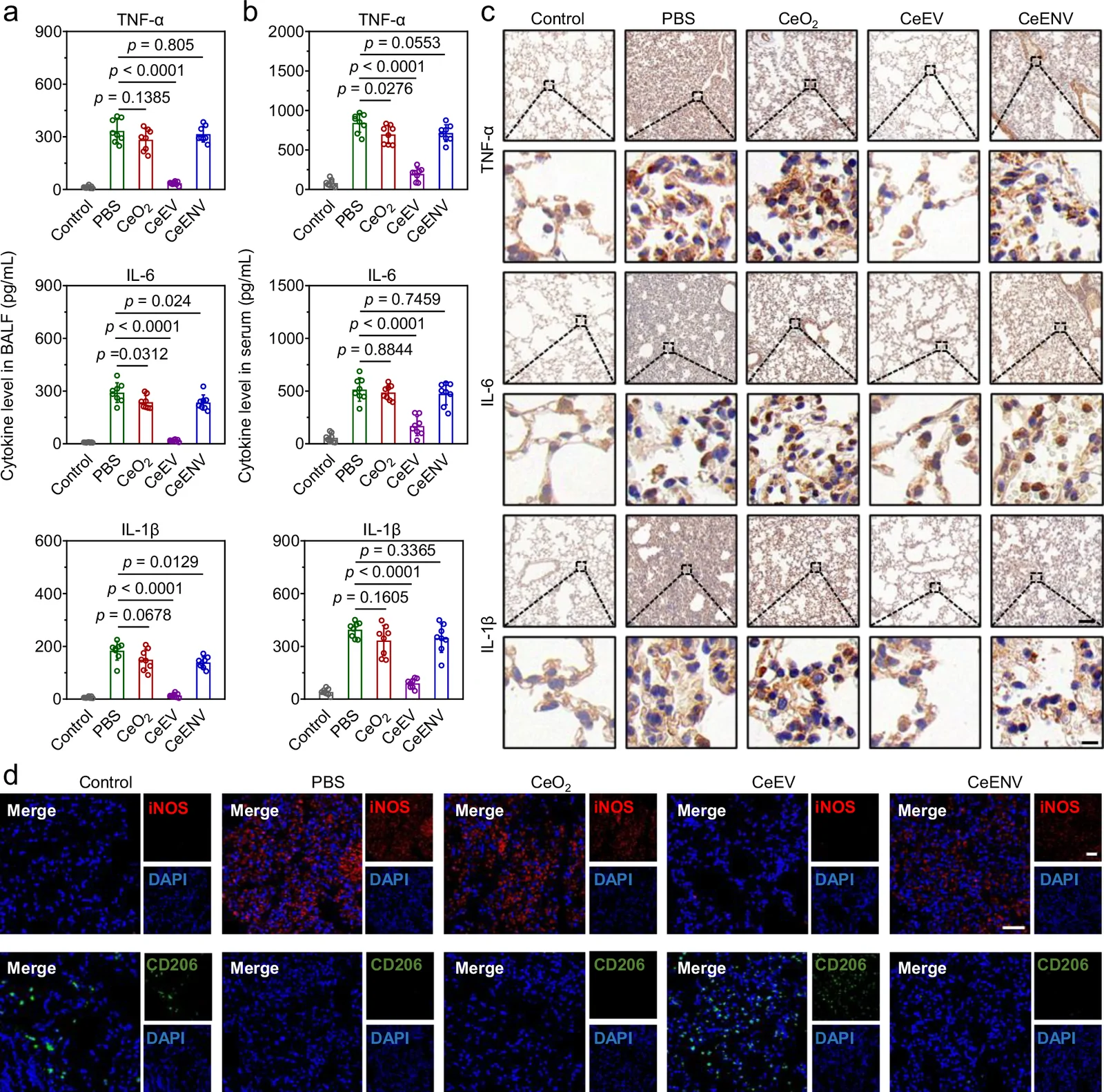
Remission of pro-inflammatory environment of lung tissues in mice with acute lung injury (ALI) through CeEV treatment. **a, b** Levels of pro-inflammatory cytokines, including tumor necrosis factor-α (TNF-α), interleukin-6 (IL-6), and interleukin-1β (IL-1β) in bronchoalveolar lavage fluid (BALF) (**a**) and serum (**b**) after intravenous (*i.v*.) injection of PBS, CeO_2_, CeEV, or CeENV (3 mg/kg). Data are presented as the mean ± S.D. (*n* = 8 male BALB/C mice per group, 6-8 weeks of age). **c** Representative immunohistochemistry (IHC) staining images of lung sections of ALI mice illustrating the expression of cytokines after administration of PBS, CeO_2_, CeEV, or CeENV (3 mg/kg). Scale bar = 500 μm (top row) or 40 μm (bottom row). The experiment was repeated independently for eight times from eight male BALB/C mice with similar results. **d** Representative immunofluorescence images of lung sections of ALI mice revealing the polarization of macrophages to proinflammatory M1 phenotype (red) or anti-inflammatory M2 phenotype (green) after treatment of PBS, CeO_2_, CeEV, or CeENV (3 mg/kg). Scale bar = 50 μm. The experiment was repeated independently for eight times from eight male BALB/C mice with similar results. Normal mice without lipopolysaccharide challenge or NP treatment served as the control. Statistical analysis in **a** and **b** was calculated using one-way ANOVA with Dunnett’s multiple comparisons test. Source data are provided as a Source Data file.

The administration of CeEV also mitigated the inflammatory environment in the lung tissue of ALI mice. To evaluate the expression of pro-inflammatory cytokines, the levels of TNF-α, IL-6, and IL-1β in both BALF and lung homogenate were measured using enzyme linked immunosorbent assay (ELISA) kit. Due to the retention of CeEV in lung tissues, its anti-inflammatory effects resulted in the significant decrease in various pro-inflammatory cytokines. The TNF-α expression, for instance, was reduced by 90% and 79% in BALF and lung homogenate after CeEV administration compared to PBS group, respectively (Fig. 6a and Supplementary Fig. 32). Conversely, the treatment of either CeO_2_ and CeENV led to unsubstantial decrease in TNF-α in both BALF and lung homogenate, which was only reduced by 6%-20% compared to PBS group (Fig. 6a and Supplementary Fig. 32). Similar trends were also observed for IL-6 and IL-1β (Fig. 6a and Supplementary Fig. 32). In addition to the lung tissues, a marked decrease of pro-inflammatory cytokines in the serum was observed after CeEV administration (Fig. 6b), suggesting the attenuation of both local and systemic inflammation in ALI mice. Consistent with the ELISA results, immunohistochemistry (IHC) analysis revealed significantly lower levels of pro-inflammatory cytokines in lung tissues than those with the treatment of PBS, CeO_2_, and CeENV (Fig. 6c and Supplementary Fig. 33).

Polarization of macrophages has also been found in close relationship with the progression of inflammation. Conversion of the macrophage phenotype from pro-inflammatory M1 to anti-inflammatory M2 is essential for the treatment of various inflammatory diseases^57,58^. Therefore, we evaluated the phenotypic transformation of macrophages in the lung of ALI mice with various treatments. Upon the administration of CeO_2_ and CeENV with unappreciated lung-targeting ability, the expression of inducible nitric oxide synthase (iNOS), an M1 marker, was predominant that resembled that of PBS group, suggesting pro-inflammatory pulmonary environments (Fig. 6d and Supplementary Fig. 34). The CeEV treatment, however, led to remarkably decreased level of iNOS and increased level of CD206, an M2 marker, indicating the repolarization from a pro-inflammatory M1 phenotype to an anti-inflammatory M2 phenotype (Fig. 6d and Supplementary Fig. 34). These results collectively suggested that the lung-targeting CeEV could relieve the pulmonary inflammation, aiding the treatment of ALI.

### Biocompatibility of SiEV in vivo

To evaluate the systemic toxicity of SiEV, healthy mice were intravenously injected with a single dose of the NPs and sacrificed. As shown in Supplementary Fig. 35, representative H&E-stained sections of major organs from SiEV-treated mice showed no signs of necrosis, inflammation, or edema compared to the control group. Additionally, given the pulmonary targeting propensity of the SiEV, we specifically investigated the potential for lung fibrosis using Masson’s trichrome staining (Supplementary Fig. 35). The results revealed no abnormal collagen deposition in the SiEV-treated group, indicating no evidence of fibrotic lesions. These histopathological findings demonstrate the satisfactory biocompatibility of SiEV following *i.v*. injection. Furthermore, we will conduct more comprehensive long‑term and repeat‑dose safety evaluations in future work.

In summary, we reported the development of sequence-specific heteropolypeptide NPs for effective lung-targeting for the treatment of pulmonary diseases. Through the incorporation of valine into surface-grafted PLG, the hydrophobic corona mediated reversible RBC complexation in situ, enhancing the lung-targeting of NPs by up to 25 folds. While the changes in side-chain structures of amino acid residues seem trivial at the first sight, it significantly altered the heteropolypeptide sequence that eventually dictated the fate of the NPs. The in situ erythrocyte hitchhiking strategy is based on synthetic polypeptides with higher stability and simplified preparation strategy, serving as another option for antibody-based targeting strategies. Meanwhile, the reversible interactions with RBC membranes of SiEV provided other delivery designs beyond lung-targeting. Therefore, we believe this work highlights the fine adjustments of macromolecular structures to modulate their biomedical performances, paving the way to the design of extrahepatic delivery of nanomedicine.

## Methods

### Ethical statement

All animal procedures were performed according to the NIH guidelines for the care and use of laboratory animals (NIH Publication 85-23 Rev. 1985) and were approved by the Animal Ethics Committee of Soochow University (ref. Nos. 202502A0516, 202503A1038, and 202509A0904).

### Materials, Cells, and Animals

All chemicals were purchased from Shanghai Aladdin Biochemical Technology Co., Ltd. (Shanghai, China) and used as received unless otherwise specified. Deuterated solvents were purchased from Cambridge Isotope Laboratories, Inc. (Tewksbury, USA). Organic solvents were purchased from Sinopharm Chemical Reagent Co., Ltd. (Shanghai, China). QDs were purchased from Beijing Zhongke Keyou Nanotechnology Co., Ltd. (Beijing, China). Fe_3_O_4_ was purchased from Jiangsu Zhichuan Technology Co., Ltd. (Jiangsu, China). CeO_2_ was purchased from Xi’an Ruixi Biological Technology Co., Ltd. (Xi’an, China). ^177^LuCl_3_ and Na^99m^TcO_4_ were obtained from Syncor Pharmaceuticals (Chengdu, China) with a radionuclide purity level of >99.9%. Dialysis bags with a molecular-weight cut-off (MWCO) of 3.5 kDa, 3-(4,5-dimethylthiazol-2-yl)-2,5-diphenyl-tetrazolium bromide (MTT) assay kit, BCA protein assay kit, and 3,3′-dioctadecyloxacarbocyanine perchlorate (DiO) were purchased from Yuanye Bio-Technology (Shanghai, China). FITC anti-mouse CD45 (1:200, catalog no. 147709, clone I3/2.3), phycoerythrin-cyanine7 (PE-Cy7) anti-mouse CD326 (1:100, catalog no. 118215, clone G8.8), PE anti-mouse CD31 (1:100, catalog no. 160203, clone G8.8), FITC anti-mouse TER-119/Erythroid cells (1:100, catalog no. 116205, clone TER-119), PE anti-mouse CD41 (1:200, catalog no. 133905, clone MWReg30), and peridinin-chlorophyll-protein complex (PerCP) anti-mouse CD45 (1:100, catalog no. 103129, clone 30-F11) were purchased from BioLegend (San Diego, USA). Anti-TNF-α rabbit multiclonal primary antibody (1:500, catalog no. ab307164, clone RM1005), anti-IL-6 rabbit monoclonal primary antibody (1:100, catalog no. ab290750, clone EPR23819-103), anti-IL-1β rabbit multiclonal primary antibody (1:500, catalog no. ab283818, clone RM1009), anti-iNOS mouse monoclonal primary antibody (1:1000, catalog no. ab210823, clone EPR16635), and anti-CD206 rabbit polyclonal primary antibody (1:1000, catalog no. ab64693) were purchased from Abcam (Cambridge, Britain). FITC-labeled WGA was purchased from Shanghai Maokang Biotechnology Co., Ltd. (Shanghai, China). Dulbecco’s modified Eagle medium (DMEM) and fetal bovine serum (FBS) were purchased from Gibco (New York, USA). LPS was purchased from Solarbio (catalog no. L8880, Beijing, China). MPO activity assay kit was purchased from Elabscience Biotechnology Co., Ltd. (Wuhan China). Ultrafiltration spin columns (MWCO = 10 kDa and 100 kDa), 4’,6-diamidino-2-phenylindole (DAPI), ROS assay kit, SOD assay kit with WST-8, CAT assay kit, hydrogen peroxide assay kit, and MDA assay kit were purchased from Beyotime Biotcehnology (Shanghai, China). ELISA kits were purchased from Aimeng Youning (Shanghai, China). 2-Amino-2-norbornanecarboxylic acid (BCH) was purchased from MedChemExpress (Monmouth Junction, USA). Anti-PECAM1 was purchased from Shenzhen Dakewe Bio-engineering Co., Ltd. (Shenzhen, China).

Anhydrous tetrahydrofuran (THF) and hexane were dried in a column filled with alumina. Anhydrous dichloromethane (DCM) was stored over 3 Å molecular sieves in a glovebox. Anhydrous *N,N*-dimetthylformamide (DMF) was pre-treated with polymer-bound isocyanates (MilliporeSigma, St. Louis, USA) to remove any amino residues.

The NCA monomers were synthesized following literature procedures^59,60^, including *t*Bu-Glu NCA, *N*^ε^-*tert*-butoxycarbonyl-_L_-lysine NCA (BLL NCA), *N*^ω^-(2,2,4,6,7-pentamethyldihydrobenzofuran-5-sulfonyl)-_L_-arginine NCA (Pbf-Arg NCA), *N*^δ^-trityl-_L_-glutamine NCA (Trt-Gln NCA), *O*-*tert*-butyl-_L_-serine NCA (*t*Bu-Ser NCA), _L_-threonine NCA (Thr NCA), *O*-*tert*-butyl-_L_-tyrosine NCA (*t*Bu-Tyr NCA), _L_-alanine NCA (Ala NCA), _L_-leucine NCA (Leu NCA), _L_-isoleucine NCA (Ile NCA), Val NCA, _L_-phenylalanine NCA (Phe NCA), and Nva NCA. Taking the synthesis of Ala NCA as an example. _L_-alanine (1.0 g, 11.2 mmol, 1.0 equiv) and THF (30 mL) were mixed in a round-bottom flask charged with a stir bar, into which triphosgene (1.66 g, 5.6 mmol, 0.5 equiv) was added in one portion (***Caution***: triphosgene and its degradation product phosgene are corrosive and toxic chemicals. The reaction waste should be quenched with NaHCO_3_ before disposal). The reaction was stirred at 50 °C for ~2 h. Subsequently, the solvent was removed under vacuum. The Ala NCA product was purified through recrystallization in THF/hexane (1:20, v/v) (yield: 78%). Specifically for NCA monomers with acid-labile side chains, including *t*Bu-Glu NCA, BLL NCA, Pbf-Arg NCA, Trt-Gln NCA, *t*Bu-Tyr NCA, and *t*Bu-Ser NCA, the reaction was conducted in the presence of propylene oxide (10.0 equiv)^61^.

RAW 264.7 cells (mouse monocyte macrophages) and iMPMVECs were purchased from the American Type Culture Collection (Rockville, USA). The cells were cultured in DMEM containing 1% nonessential amino acids, 10% FBS, and 1% _L_-glutamine.

Male and female BALB/c mice (6–8 weeks) were obtained from Cavens Model Animal Co., Ltd (Changzhou, China), and housed in a specific pathogen-free (SPF) room with a 12-h light/dark cycle, standard environmental temperature of 25 °C, and humidity of 40–50%.

### Instruments

^1^H NMR spectra were recorded on a Bruker AVANCE NEO-400 MHz spectrometer. The chemical shifts (*δ*) were reported in ppm and referenced to the residual protons in the deuterated solvents. MestReNova software (version 6.1.0, Mestrelab Research, Escondido, USA) was used for all NMR analysis. DLS experiments were performed using a Zeta-sizer Nano ZS90 (Malvern Panalytical Ltd., Malvern, UK) with a He-Ne laser (*λ* = 633 nm) at a scattering angle of 90° at 25 °C. TEM images were recorded using a Tecnai G2 F20 electron microscope operated at 200 kV (FEI Nano Ports, Hillsboro, USA). TEM samples were prepared via the addition of an aliquot of the NP suspension onto the carbon-coated copper grids, followed by the evaporation of the solvent in a fume hood overnight. GPC experiments were performed on a system equipped with an isocratic pump (1260 Infinity II, Agilent, Santa Clara, USA), a multi-angle static light scattering (MALS) detector (DAWN, Wyatt Technology, Santa Barbara, USA), and a differential refractive index (dRI) detector (Optilab, Wyatt Technology, Santa Barbara, USA). The detection wavelength of the MALS detector was set at 658 nm. Separations were performed using serially connected size exclusion columns (KD-803, KD-804, and KD-806, 8 × 300 mm, Shodex, Yokohama, Japan) using DMF containing LiBr (0.1 mol/L) as the mobile phase at a flow rate of 1 mL/min at 60 °C. The MALS detector was calibrated using pure toluene and was used for the determination of the absolute molecular weights (MWs). The MWs of polymers were determined based on the d*n*/d*c* value of each polymer sample calculated offline by using the internal calibration system processed by the ASTRA 8 software (version 8.1.0, Wyatt Technology, Santa Bar-bara, USA). SEM images were collected with a Zeiss Gemini 500 microscope (Carl Zeiss, Oberkochen, Germany). CLSM images were acquired by a ZEISS LSM 800 microscope with an Airyscan detector (Carl Zeiss, Oberkochen, Germany). Cell microplates were analyzed by a BioTek Synergy H1 multifunctional microplate reader (Agilent, Santa Clara, USA). Flow cytometry experiments were performed using a BD Accuri C6 Plus flow cytometer (BD Biosciences, Franklin Lakes, USA) and analyzed using a FlowJo software (version 10.8.1, BD Biosciences, Franklin Lakes, USA). The ex vivo fluorescent imaging of collected organs of mice was performed by the PerkinElmer in vivo imaging systems (IVIS) (PerkinElmer, Shelton, USA). ICP-OES was used to measure the silicon content of NPs in lungs and blood on an Avio 200 ICP-OES spectrometer (PerkinElmer, Shelton, USA). Radioactivity counts were measured with a WIZARD Gamma counter (PerkinElmer, Shelton, USA).

### Synthesis of heteropolypeptide-grafted nanoparticles

Heteropolypeptide-grafted NPs, referred to as SiEX, were prepared through surface-initiated ring-opening polymerization (ROP) of NCA monomers with amine-functionalized silica NPs. The acid-labile side chains of polypeptides were then deprotected by the treatment of trifluoracetic acid (TFA) to render the final heteropolypeptides water-soluble.

Silica NPs were synthesized by the Stöber method^62^, whose surface was then functionalized by (3-aminopropyl)triethoxysilane (APTES) to introduce surface amino groups. Typically, ethanol (100 mL), deionized (DI) water (2.5 mL), and ammonia (25%, 4.0 mL) were mixed for 15 min and transferred into a 250-mL round-bottom flask, into which the mixture of ethanol (25 mL) and tetraethyl orthosilicate (TEOS, 3 mL) was added and gently stirred for 25 min. The resulting mixture was then stirred slowly at 70 °C for 2 h, followed by the addition of ammonia (2.0 mL) and further stirring for 3 h. The final SiO_2_ NPs were purified by centrifugation and washing 3 times with DI water, which were re-dispersed in ethanol to obtain a milky suspension.

To introduce amino groups through surface functionalization, APTES was first pre-hydrolyzed to get silanol, which was then mixed with the obtained SiO_2_ NPs. Typically, ethanol/water (90 mL, 95:5, v/v) was added into a 250-mL round-bottom flask, which was acidified to pH ~5.0 through the addition of glacial acetic acid (60 μL). APTES was then added (4.4 mL, 19 mmol), and the resulting clear solution was stirred at room temperature for 5 min. Meanwhile, the dispersion of SiO_2_ NPs (1.0 g) in ethanol (10 mL) was added into the solution of APTES silanol. The mixture was stirred at room temperature for 2 h. The amine-functionalized SiO_2_ NPs were purified by centrifugation and washing 3 times with ethanol, acetone, and THF, which were then dried overnight in an oven at 50 °C (yield: 84%).

Polypeptides were grafted onto the surface of SiO_2_ NPs through surface initiation. Taking the synthesis of NPs decorated with PEV (i.e., SiEV) as an example. The monomers *t*Bu-Glu NCA (39.9 mg, 0.17 mmol) and Val NCA (2.8 mg, 0.019 mmol) were first dissolved in anhydrous DCM:DMF (1:1, v/v), followed by the addition of a dispersion of amine-functionalized SiO_2_ NPs (14 mg, containing ~18 μmol amino groups) in DCM:DMF. The total volume of solvent was 472 μL ([M]_0_ = 0.4 M). The polymerization was stirred at room temperature until the monomer conversion reached >  99% ( ~ 3 h). The heteropolypeptide-decorated NPs were purified by precipitation with ether/hexane (1:1, v/v) and washing 3 times with ether and THF, dried under vacuum, and suspended in TFA:DCM (500 μL, 1:1, v/v) for side-chain deprotection. The reaction mixture was stirred at 0 °C in an ice bath for 2 h. The final NPs were precipitated with ether/hexane (1:1, v/v), re-dissolved in the aqueous solution of NaHCO_3_ (0.2 M), and then purified by dialysis (MWCO = 3.5 kDa) and lyophilization (yield: 81%).

For the fluorescent labeling of NPs, a small amount of BLL-NCA ( ~ 1 mol%) was added during the polymerization process. After the side-chain deprotection, the *N*-hydroxysuccinimide (NHS) ester of Cy5 was used as a fluorescent probe to react with the exposed lysine residues.

The synthesis of SiEV with different sizes was conducted in a similar way, but with silica NPs bearing different sizes (controlled by the ratio of ethanol and ammonia during Stöber synthesis).

The synthesis of SiEV with different valine percentages was conducted in a similar way, but with different feeding ratios of *t*Bu-Glu NCA and Val NCA.

The synthesis of FeEV, QEV, and CeEV was conducted in a similar way, but with different amine-functionalized inorganic cores including Fe_3_O_4_ NPs, QDs, and CeO_2_ NPs.

Si(E-*b*-NV), Si(E-*b*-L), and Si(E-*b*-F) NPs with block copolypeptide decorations were prepared by the sequential feeding of NCA monomers. Typically, *t*Bu-Glu NCA (39.9 mg, 0.17 mmol) was first dissolved in DCM:DMF (425 μL, 1:1, v/v), followed by the addition of amine-functionalized SiO_2_ NPs (14 mg, containing ~18 μmol amino groups). The polymerization was stirred at room temperature until the monomer conversion reached > 99% ( ~ 3 h). Subsequently, the DCM:DMF solution (47 μL, 1:1, v/v) of Nva NCA (2.8 mg, 0.019 mmol) was added into the solution for chain extension. The synthesis of Si(E-*b*-L) and Si(E-*b*-F) was conducted in a similar way.

### Synthesis of anti-PECAM1-grafted nanoparticles

Anti-PECAM1 was conjugated on NPs through copper-free click chemistry^63^. Typically, the NHS ester of dibenzocyclooctyne (DBCO-NHS) in dimethyl sulfoxide (DMSO, 10 mg/mL, 5 μL) was added into the suspension of amine-functionalized SiO_2_ NPs (7 mg, containing ~9 μmol amino groups) and stirred at 4 °C for 1 h, followed by ultrafiltration (MWCO = 10 kDa, 2400 *× g*) for 20 min and washing 3 times with PBS to remove the unreacted DBCO-NHS. At the same time, to obtain the azido derivative of anti-PECAM1, N_3_-poly(ethlene glycol)-NHS ester (N_3_-PEG-NHS) in DMSO (10 mg/mL, 7 μL) was added into anti-PECAM1 (0.2 mg/mL, 10 μL) and stirred at 4 °C for 1 h, followed by ultrafiltration (MWCO = 10 kDa, 2400 *× g*) for 20 min and washing 3 times with PBS to remove the unreacted N_3_-PEG-NHS. The suspension of DBCO-modified NPs and N_3_-modified anti-PECAM1 (1:1) was mixed, which was stirred at 4 °C for 24 h, followed by ultrafiltration (MWCO = 100 kDa, 2400 *× g*) for 20 min and washing 3 times with PBS.

### Characterization of heteropolypeptide-grafted nanoparticles

The sizes and zeta potentials of NPs were studied by DLS and TEM. Briefly, the PBS suspension of heteropolypeptide-modified NPs (0.1 mg/mL) was filtered through nylon membrane (0.22 μm) twice to remove any dust. The suspension was then characterized with a Zeta-sizer. Additionally, an aliquot of the ethanol dispersion of NPs (10 μg/mL) was placed onto the TEM grid, which was left to dry overnight and then visualized by TEM. To evaluate the stability of SiEV in plasma, blood from healthy mice was collected in anticoagulated blood tubes and centrifuged to isolate plasma (900 *× g*, 10 min), which was incubated with SiEV at 37 °C for different time periods. The changes in size were subsequently measured by DLS.

Meanwhile, NMR was used to determine the copolymerization ratio and the deprotection efficiency of the surface-grafted heteropolypeptides after the etching of silica core by HF. Briefly, SiEV (10 mg) was dissolved in THF (3.0 mL), into which the HF solution (84.3 mg/mL, 0.5 mL) was added (***Caution***: HF is a corrosive and toxic chemical. It should be used in a fume hood and with great care to avoid direct exposure). The resulting mixture was stirred at room temperature for 12 h, and the heteropolypeptides were isolated by precipitation in DI water (5.0 mL). After centrifugation, the heteropolypeptides were further purified through precipitation until the pH of the water was close to neutral.

Finally, GPC was used to determine the MWs and molecular weight distribution (MWD) of the heteropolypeptide-grafted NPs after the silica core was etched by HF. Briefly, poly(γ-benzyl-_L_-glutamate)-*co*-poly(_L_-valine)-modified NPs (10 mg) was dissolved in THF (3.0 mL), into which the HF solution (84.3 mg/mL, 0.5 mL) was added. The benzyl derivatives of poly(glutamic acid)s were used rather than the *tert*-butyl derivatives, mainly due to the poor solubility of the *tert*-butyl-based polypeptide in DMF. The resulting mixture was stirred at room temperature for 12 h, and the isolated polypeptide chains were precipitated with DI water (5.0 mL). After centrifugation, the polypeptides were re-dissolved in THF and precipitated in water, until the pH of the water was close to neutral. The obtained polypeptides were then dried, re-dissolved in DMF containing LiBr (0.1 M), filtered through PTFE membrane (0.22 μm), and analysed by GPC.

### Preparation of ^177^Lu-labeled SiEV

^177^LuCl_3_ (130 MBq) was diluted in sodium acetate buffer (0.1 M, pH = 5.5) and mixed with SiEV (1 mg/mL, 2 mL) at room temperature for 10 min. Following centrifugation (9600 *× g*, 10 min), the supernatant was removed, and the radioactivity associated with the pellet was quantified to determine the labeling yield.

### Preparation of ^99m^Tc-labeled erythrocytes

Freshly isolated blood was incubated with SnCl_2_ (1 mg/mL, 20 μL), glucose (5.5 mg), sodium citrate (3.7 mg), and NaCl (0.11 mg) at room temperature for 5 min. Subsequently, NaClO (0.1%, 200 μL), Na_2_EDTA (4.4%, 500 μL), and Na^99m^TcO_4_ (148 MBq) were added and incubated at room temperature for 20 min. Following centrifugation (900 *× g*, 10 min), the supernatant was removed, and the radioactivity associated with the cell pellet was quantified to determine the labeling yield.

### Biodistribution of heteropolypeptide-grafted nanoparticles

Healthy female BALB/c mice were randomly divided into 12 groups (*n* = 3). The mice were intravenously injected with Cy5-labeled polypeptide-decorated NPs (1.0 mg/mL, 100 μL), which were sacrificed at 4 h post administration, with their major organs (heart, liver, spleen, lung, and kidney) collected. The semi-quantitative Cy5 fluorescent signals in the organs were analyzed using an IVIS optical imaging system (*λ*_ex_ = 620 nm, *λ*_em_ = 670 nm).

To visualize the distribution of Cy5-labeled NPs in lung cells, the collected lung tissue was washed with PBS, embedded in optimum cutting temperature compound (OCT), and cryo-sectioned at 10 μm thickness. After DAPI staining for 10 min, the lung tissue sections were studied by CLSM.

To check the retention time of the SiEV in lung tissues, SiEV were injected into female BALB/c mice (*n* = 3). The mice were sacrificed at pre-determined time intervals post injection, with their major organs (heart, liver, spleen, lung, and kidney) collected, and the semi-quantitative Cy5 fluorescent signals collected using an IVIS optical imaging system. After the homogenization of lung tissues, filtering, and lysis of RBCs, the Cy5 fluorescent signals of the supernatant were quantitatively analyzed according to the standard curve of Cy5-labeled SiEV in lysis buffer. Additionally, ICP-OES was used to measure the silicon content in the lung tissues at different time points. Briefly, SiEV were intravenously injected into female BALB/c mice (*n* = 3), and then the lung tissues of each group of mice at different time points were separately weighed and digested with HNO_3_ to quantify the silicon content by ICP-OES. Meanwhile, ^177^Lu-labeling SiEV was intravenously injected into female BALB/c mice (*n* = 3), the lung tissues were collected at different time points, and the content of ^177^Lu was quantified using the Gamma counter.

For the calculation of the lung-to-liver ratio, the mice were sacrificed at 12 h post *i.v*. injection of SiEV with different sizes, with their major organs (heart, liver, spleen, lung, and kidney) collected, and the semi-quantitative Cy5 fluorescent signals collected using an IVIS optical imaging system. After the homogenization of lung or liver, filtering, and lysis of RBCs, the Cy5 fluorescent signals of the supernatant were quantitatively analyzed according to the standard curve of Cy5-labeled SiEV in lysis buffer.

### In vivo cellular localization

To investigate the cellular distribution and enrichment of SiEV within pulmonary tissues, the NPs were administered via *i.v*. injection. After 12-h circulation period, lung tissues were harvested for subsequent immunofluorescence staining and quantitative analysis using flow cytometry.

For isolation and staining of lung cells, the lung tissues were minced using a sterile blade and digested with trypsin (500 µL, 30 min). The lung homogenates were filtered using a 70-µm filter and washed once with PBS. After centrifugation (900 *× g*, 5 min), the supernatant was removed, and the cell pellets were re-suspended in RBC lysis buffer (5 mL), which was incubated on ice for 5 min. The mixture was then centrifuged (900 × *g*, 5min), and the cells were re-suspended in cell staining buffer. The cell suspensions were then added into flow tubes with antibodies (total volume 100 µL), which were incubated for 30 min in the dark at 4 °C. The stained cells were washed twice with PBS and re-suspended in PBS (100 µL) for flow cytometric analysis. The antibodies used were FITC anti-mouse CD45, PE-Cy7 anti-mouse CD326, and PE anti-mouse CD31.

Meanwhile, to visualize the distribution of NPs in lung cells, the lung tissue sections were stained with antibodies and visualized by CLSM.

### Pharmacokinetics of heteropolypeptide-modified nanoparticles

The mice were intravenously injected with SiEV or SiE (1 mg/mL, 100 μL). Blood samples (100 μL) were collected from the retro-orbital sinus at pre-determined time intervals (1, 3, 6, 12, and 24 h) and were then collected in anticoagulated blood tubes. The silicon content was quantified by ICP-OES.

### The stability of RBC-SiEV complexes

Blood from mice was collected in anticoagulated blood tubes and subsequently centrifuged to remove the top layer of plasma (900 *× g*, 10 min). SiEV was incubated with RBCs at 37 ^o^C under shaking (100 rpm) to mimic the condition within the blood vessels^48^, and the resulting suspensions were centrifugated at various time points to separate the erythrocyte-SiEV complexes. The fluorescence of SiEV in the supernatant was measured and used to calculate the amount of SiEV remaining unbound via a standard curve.

### Studies on the interactions between nanoparticles and erythrocytes

The binding affinity of various surface-modified heteropolypeptide NPs to RBCs was evaluated. Briefly, blood from healthy mice was collected in anticoagulated blood tubes and then centrifuged to separate the plasma (900 *× g*, 10 min), which was then separately incubated with heteropolypeptide-modified NPs at 37 °C for 1 h. The erythrocyte suspension was stained with FITC anti-mouse TER-119/Erythroid cells. Flow cytometry was used to quantitatively assess the binding of NPs to erythrocyte membranes.

Hitchhiking efficiency on erythrocytes was determined by fluorescence measurements. Briefly, blood from healthy mice was collected in anticoagulated blood tubes and then incubated with NPs at the ratio of 1:8000 (RBCs:NPs) at 37 ^o^C for 1 h. After centrifugation (900 *× g*, 10 min), the supernatant was analyzed for fluorescent intensity. The binding efficiency of SiEV to RBCs was calculated according to the standard curve of Cy5-labeled SiEV in lysis buffer. The number of SiEV bound per RBCs was calculated based on the binding efficiency.

For the in vivo analysis of erythrocyte-SiEV complexation, healthy female BALB/c mice were intravenously injected with SiEV and SiENV (1 mg/mL, 100 μL). Blood from healthy mice was collected in anticoagulated blood tubes at 1 h post injection, which was subsequently centrifuged to remove the top layer of plasma (900 *× g*, 10 min). The erythrocyte suspension was stained with FITC-labeled WGA (0.1 μM) for 5 min in the dark, and the colocalization of NPs and erythrocyte membranes was analysed by CLSM and SEM. Flow cytometry was used to quantitatively assess the binding of NPs to erythrocyte membranes.

### Desorption of nanoparticles from erythrocyte membrane

In order to simulate the lung-accumulation of SiEV through shear-induced NP release from erythrocytes in pulmonary capillaries, the formed complexes were extruded through syringes and the desorption of SiEV was quantified according to the decrease in Cy5 fluorescent signals. First, erythrocyte suspension (0.5 mL, 5 × 10^8^ erythrocyte per mL) was incubated with SiEV for 1 h, which was then gently washed twice with PBS to remove the free NPs. The suspension of erythrocyte-SiEV complexes was then washed 3 times with PBS by extrusion using a syringe, which was evaluated quantitatively by flow cytometry. The desorption percentage was determined compared to the complexes without additional extrusion treatments.

FITC-labeled RBCs and Cy5-labeled SiEV were used for desorption studies in vivo. Briefly, FITC-labeled RBCs were pre-incubated with Cy5-labeled SiEV, and the resulting complexes were then intravenously injected into the mice (1 mg/mL, 100 μL). At pre-determined time points post injection (1, 6, and 12 h), blood from mice was collected in anticoagulated blood tubes and then mice were euthanized to collect lungs. Flow cytometry was used to analyze the signals of FITC and Cy5 in the blood and lung tissues, respectively. Mice that were intravenously injected with only FITC-labeled RBCs were used as the control group. Additionally, to visualize the desorption of SiEV, the lung tissues were collected at 3 h or 12 h after *i.v*. injection of complexes, washed with PBS, embedded in OCT, and cryo-sectioned at 10-μm thickness. After DAPI staining for 10 min, the lung tissue sections were studied by CLSM.

The radioisotope labeling and tracing experiments were conducted in a similar way, but using radioisotope ^99m^Tc and ^177^Lu to label RBCs and SiEV, respectively.

### Studies on the interactions between nanoparticles and GUVs

GUVs were first formulated with film rehydration method^64^. Briefly, 1,2-distearoyl-*sn*-glycero-3-phosphocholine (DSPC) and cholesterol (CHOL) were weighed to achieve a molar ratio of DSPC:CHOL = 7:3. The mixture was dissolved in chloroform, condensed to a membrane by evaporation, and then dried under vacuum overnight. Subsequently, the dried film was hydrated with HEPES buffer (pH = 7.0) buffer at 65 °C, leading to film detachment and self-assembly into GUVs. SiEV or SiENV was incubated with liposome at 37 °C for 1 h, stained with DiO (5.0 μM) for 10 min in the dark, and the colocalization of NPs and lipid membranes was analysed by CLSM.

### The effect of protein corona on the interactions between SiEV and erythrocytes

To study the influence of protein corona formation on the interaction between SiEV and RBCs, SiEV was first treated with plasma for protein corona formation. Blood from healthy mice was collected in anticoagulated blood tubes and then centrifuged to separate the plasma (900 *× g*, 10 min). SiEV was incubated with plasma at 37 °C for 1 h to yield SiEV^PC^, where the protein adsorption was confirmed by DLS and zeta potential. Furthermore, BCA assay kit was used to determine the protein concentration of SiEV^PC^. Erythrocyte suspension (0.5 mL, 5 × 10^8^ erythrocyte per mL) was then incubated with SiEV or SiEV^PC^ for 1 h, which was then gently washed twice with PBS to remove the free NPs and stained with FITC anti-mouse TER-119/Erythroid cells. Flow cytometry was used to quantitatively assess the binding of NPs to erythrocyte membranes.

### The effect of LAT1 on the interactions between SiEV and erythrocytes

To study the effect of _L_-type amino acid transporter (LAT1) on the interaction between SiEV and RBCs, RBCs were pre-incubated with BCH, an inhibitor for LAT1. Blood from healthy mice was collected in anticoagulated blood tubes and then centrifuged to separate the plasma (900 × *g*, 10 min). RBC or BCH (30 mM)-treated RBC suspension (0.5 mL, 5 × 10^8^ erythrocyte per mL) was incubated with SiEV for 1 h, which was then gently washed twice with PBS to remove free NPs. Quantitative evaluation of the binding of NPs to RBCs was assessed by flow cytometry.

### Cellular uptake

The iMPMVECs were seeded in a 12-well plate at 2 × 10^5^ cells/well and cultured at 37 °C overnight. The Cy5-labeled SiEV, SiENV, or Si^PECAM1^ (20 μg/mL) were incubated with cells in DMEM at 37 °C for 4 h, and the cells were washed three times with PBS. The cellular uptake level of NPs was determined by flow cytometry with untreated cells as the blank. The Cy5-positive cells were calculated using the FlowJo software.

For the antibody-blocking experiment, anti-PECAM1 (0.2 mg/mL, 3 μL) was pre-employed to block PECAM1 expressed on the surface of iMPMVECs for 2 h. The Cy5-labeled SiEV and Si^PECAM1^ (20 μg/mL) were added in DMEM at 37 °C for 4 h, and the cells were then washed three times with PBS. The cellular uptake level of SiEV and Si^PECAM1^ was determined by flow cytometry with untreated cells used as the blank. The MFI per cell was calculated using the FlowJo software.

### In situ polymerization kinetics

The polymerization kinetics initiated by *n*-hexylamine (Hex-NH_2_) was used to mimic the surface-initiated polymerization, which was monitored by ^1^H NMR in the cosolvent of CD_2_Cl_2_ and DMF-*d*7. Typically, *t*Bu-Glu NCA (66 mg, 0.29 mmol) and Val NCA (4.6 mg, 0.032 mmol) were dissolved in CD_2_Cl_2_:DMF-*d*7 (805 µL, 1:1, v/v), followed by the addition of the solution of Hex-NH_2_ (0.2 M, 32 µL). The polymerization mixture ([M]_0_ = 0.4 M) was vortexed and transferred into an NMR tube, and the ^1^H NMR spectra were collected at different time points. To determine the conversion of NCA, the integral of α-H signal of *t*Bu-Glu NCA (*δ* = 4.30 ppm) and Val NCA (*δ* = 4.11 ppm) was normalized compared to the α-H signal at *t* = 0 (i.e., 100 % remaining NCA).

The polymerization kinetics of *t*Bu-Glu NCA and Nva NCA was monitored in a similar way, but with Nva NCA instead of Val NCA.

### Cytotoxicity and hemocompatibility assay

RAW 264.7 cells were seeded in a 96-well plate at 1 × 10^4^ cells/well and cultured at 37 °C overnight. Various volumes of suspensions of CeO_2_ or heteropolypeptide-modified NPs were then added so that the final concentration was 1, 10, 20, 40, 80, and 100 μg/mL. The cells were then incubated with NPs in DMEM at 37 °C for 4 h. The cell viability was determined through MTT assay. Results were expressed as the percentage viability of control cells that were not treated by any NPs.

Blood from healthy mice was collected in anticoagulated blood tubes and subsequently centrifuged to separate the plasma (900 *× g*, 10 min). The bottom layer was washed several times with PBS until the supernatant was colorless, which was then re-dispersed that the final suspension is 2% erythrocyte. H_2_O (positive control), PBS (negative control), and the suspension of various heteropolypeptide-modified silica NPs (0.1 mg/mL) were then mixed with an equal volume of erythrocyte suspension (2%), which were incubated at 37 °C for 4 h. The resulting mixture was centrifuged, and the supernatant was subjected to absorbance analysis (*λ* = 576 nm). Hemolysis was calculated using Eq. (1):

1 $${{\rm{Hemolysis}}}(\%)=({A}_{{{\rm{S}}}}-{A}_{{{\rm{NC}}}})/({A}_{{{\rm{PC}}}}-{A}_{{{\rm{NC}}}})\times 100$$

Where *A*_S_, *A*_PC_, and *A*_NC_ represent the absorbance at 576 nm of the sample, positive control, and negative control, respectively.

Additionally, agglutination of RBCs was performed following previously reported protocols^12^. Specifically, RBCs and RBC-NP suspensions were placed onto a round-bottom well plate at 37 °C for 1 h, which was then visualized. The RBCs incubated with H_2_O and PBS were used as positive and negative controls, respectively.

### In vitro anti-inflammation efficiency

To determine the ROS-scavenging efficiency of CeO_2_, CeEV, or CeENV, RAW 264.7 cells were seeded on 6-well plates at 2 × 10^5^ cells/well and cultured for 24 h. Cells were pre-treated with LPS (100 ng/mL) for 6 h, and then incubated with CeO_2_, CeEV, or CeENV for 24 h (40 μg/mL). Subsequently, the cells were stained with 2’,7’-dichlorodihydrofluorescein diacetate (DCFH-DA) for 30 min and washed 3 times with PBS. The cellular ROS were quantified using flow cytometry (*λ*_ex_ = 488 nm, *λ*_em_ = 525 nm). Additionally, the levels of various inflammatory factors, including TNF-α, IL-6, and IL-1β were evaluated by ELISA kit.

### SOD-mimetic activity assay

The SOD-mimetic activity was measured using a total SOD assay kit with WST-8, where the presence of SOD-like NPs inhibited the formation of superoxide and the generation of formazan dyes. Briefly, the suspension of CeO_2_, CeEV, or CeENV NPs (40 μg/mL, 20 μL), WST-8/enzyme working solution (160 μL), and reaction start solution (20 μL) were mixed in a 96-well plate. Additionally, control solution 1 without superoxide-consuming components was prepared by mixing WST-8/enzyme working solution (160 μL), SOD buffer (20 μL), and reaction start solution (20 μL). Control solution 2 contains WST-8/enzyme working solution (160 μL) and SOD buffer (40 μL). The mixture was incubated for 30 min at 37 °C and the absorbance of the mixture was quantified (*λ* = 450 nm). SOD-mimetic activity was calculated using Eq. (2):

2 $${{\rm{SOD}}}-{{\rm{mimetic\; activity}}}(\%)=({A}_{{{\rm{C}}}1}-{A}_{{{\rm{S}}}})/({A}_{{{\rm{C}}}1}-{A}_{{{\rm{C}}}2})\times 100$$

Where *A*_C1_, *A*_S_, and *A*_C2_ represent the absorbance at 450 nm of control solution 1, sample, and control solution 2, respectively.

### CAT-mimetic activity assay

The H_2_O_2_ scavenging activities of CeO_2_, CeEV, or CeENV were assessed with a CAT assay kit. Typically, the suspension of CeO_2_, CeEV, or CeENV was incubated with freshly diluted H_2_O_2_ solution (250 mM, 10 μL) for 5 min at 25 °C, followed by the addition of the termination solution (250 μL). The solution of chromogenic reagent (50 μL) was then added to each sample and incubated for 15 min, followed by the measurement of absorbance (*λ* = 520 nm). The remaining H_2_O_2_ was quantified according to the standard curve to calculate the CAT-mimetic activity.

### Mouse models of acute lung injury

Male BALB/c mice were randomly divided into five groups, with four groups receiving *i.t*. injection of LPS (2 mg/kg in saline, 75 μL) to induce ALI. The ALI animals then received *i.v*. injection of PBS, CeO_2_, CeEV, or CeENV NPs (3 mg/kg, 75 μL) after 2 h. The fifth group without LPS stimulation or NP treatment served as the normal control. Mice were euthanized after 22 h, and lung tissues were collected for the various analyses.

### In vivo anti-inflammation efficiency

The lung homogenate was prepared as mentioned above, which was then centrifuged at 4 ^o^C (3000 *× g*, 10 min) and the concentrations of pro-inflammatory cytokines (i.e., TNF-α, IL-6, and IL-1β) and oxidative stress markers (i.e., H_2_O_2_, MPO, and MDA) in the supernatant were determined using ELISA kits or commercial assay kits.

### Collection and analysis of bronchoalveolar lavage fluid

The BALF was collected from healthy mice or ALI mice at 22 h post administration of PBS, CeO_2_, CeEV, or CeENV, which was centrifuged at 4 °C (3000 *× g*, 10 min). The supernatant was collected, which was subjected to the measurement of levels of TNF-α, IL-6, and IL-1β using the ELISA kit or total protein level using the BCA kit. Additionally, the cell pellets were re-suspended in PBS for total cell counting.

### Hematoxylin-eosin staining

The lung tissues were collected from healthy mice or ALI mice at 22 h post administration of PBS, CeO_2_, CeEV, or CeENV, fixed in 10% neutral buffered formalin, embedded in paraffin, sectioned at 8-μm thickness, and stained with H&E before histological observation using CLSM.

### Measurement of wet/dry weight ratios of lung tissues

The pulmonary edema was quantified by calculating the wet/dry weight ratio of lung tissues. At 22 h post injection of PBS, CeO_2_, CeEV, or CeENV, the right lung lobe of ALI mice was excised, washed with PBS, blotted, and weighed to obtain the “wet” weight. The lung tissue was then dried at 50 °C for 24 h and weighed to obtain the “dry” weight, and the wet/dry weight ratio was calculated accordingly. The wet/dry weight ratio of lung tissues of healthy mice was calculated in a similar way as control.

### Blood gas analysis

Blood samples were obtained from the carotid artery at 22 h post administration of PBS, CeO_2_, CeEV, or CeENV, and were directly subjected to the measurement of PaO_2_ and PaCO_2_ by using the blood-gas analyzer (Radiometer, Shanghai, China). PaO_2_ and PaCO_2_ from blood samples of healthy mice was analyzed in a similar way as control.

### Immunohistochemistry analysis

Sections of lung tissues from healthy or ALI mice were incubated with anti-TNF-α rabbit multiclonal primary antibody, anti-IL-6 rabbit monoclonal primary antibody, or anti-IL-1β rabbit multiclonal primary antibody. After the removal of the antibodies, the samples were incubated with horseradish peroxidase (HRP)-conjugated goat anti-rabbit secondary antibodies for 15 min at room temperature. The antibody complexes were visualized by diaminobenzidine (DAB) solution, the samples were counter-stained with Mayer’s hematoxylin for 15 s. Positive cells of each protein were randomly counted at 60 × magnification via ImageJ software.

### In vivo evaluation of macrophage phenotype

The sections were incubated overnight at 4 °C with specific antibodies, including anti-iNOS mouse monoclonal primary antibody and anti-CD206 rabbit polyclonal primary antibody, followed by the staining with Alexa Fluor 555-conjugated goat anti-mouse and Alexa Fluor 488-conjugated goat anti-rabbit secondary antibodies for 15 min at room temperature, respectively. Cell nuclei were stained with DAPI at room temperature for 10 min. Images of the sections were captured using CLSM. Positive cells of macrophage phenotype in eight randomly selected fields of view were analyzed by ImageJ software.

### In vivo safety evaluation

PBS (100 μL) or SiEV (1 mg/mL, 100 μL) was intravenously injected to female BALB/c mice, and major organs (heart, liver, spleen, lung, and kidney) were collected at 7 d post *i.v*. injection. The tissue sections were stained with H&E as descried above and analyzed. Additionally, the collected lung tissues were also stained with Masson’s trichrome to observe the occurrence of fibrosis.

### Statistical Analysis

Statistical analysis was calculated with GraphPad Prism 9. The data were reported as mean ± S.D. Declared group size (*n*) refers to the number of independent, biological values rather than technical replicates, and statistical analysis were undertaken only where group size was at least *n* = 3. The group size is the number of independent values, and statistical analysis was done using these independent values, and there were no outliers in the data analysis. The ordinary one-way analysis of variance (ANOVA) with Dunnet’s multiple comparisons test or two-tailed unpaired *t*-test were employed for statistical difference calculation.

### Reporting summary

Further information on research design is available in the Nature Portfolio Reporting Summary linked to this article.

## Supplementary information


Supplementary Information
Reporting Summary
Transparent Peer Review file


## Source data


Source Data


## Data Availability

All data generated in this study are provided in the Supplementary Information and Source Data file. Source data are provided with this paper.
