# Supplementary material for: Sequence-regulated, lung-targeting heteropolypeptide nanoparticles via in situ erythrocyte hitchhiking
Source: Nat Commun. 2026 Jul 30;17:9251. doi: 10.1038/s41467-026-76188-x (PMC13526850; doi:10.1038/s41467-026-76188-x)
Supplement: Supplementary file 1 [file 41467_2026_76188_MOESM1_ESM.pdf]

Supplementary Information

Sequence-Regulated, Lung-Targeting Heteropolypeptide Nanoparticles via *in situ* Erythrocyte Hitchhiking

Ning Li[a], Jiahe Shen[a], Yuheng Lei[a], Junhong Wang[b], Wei Zhou[a], Aoting Li[a], Hui Liu[a], Shanshan Xiao[a], Jing Zhao[a], Shaobo Feng[a], Guanglin Wang[b], Lichen Yin [a,] *, and Ziyuan Song [a,] *

[a]Institute of Functional Nano & Soft Materials (FUNSOM), Jiangsu Key Laboratory for Carbon-Based Functional Materials and Devices, Soochow University, Suzhou 215123, China.

[b]State Key Laboratory of Radiation Medicine and Protection, School for Radiological and Interdisciplinary Sciences (RAD-X), Collaborative Innovation Center of Radiation Medicine of Jiangsu Higher Education Institutions, Soochow University, Suzhou 215123, China.

*Corresponding email: zysong@suda.edu.cn, lcyin@suda.edu.cn

Supplementary Figures

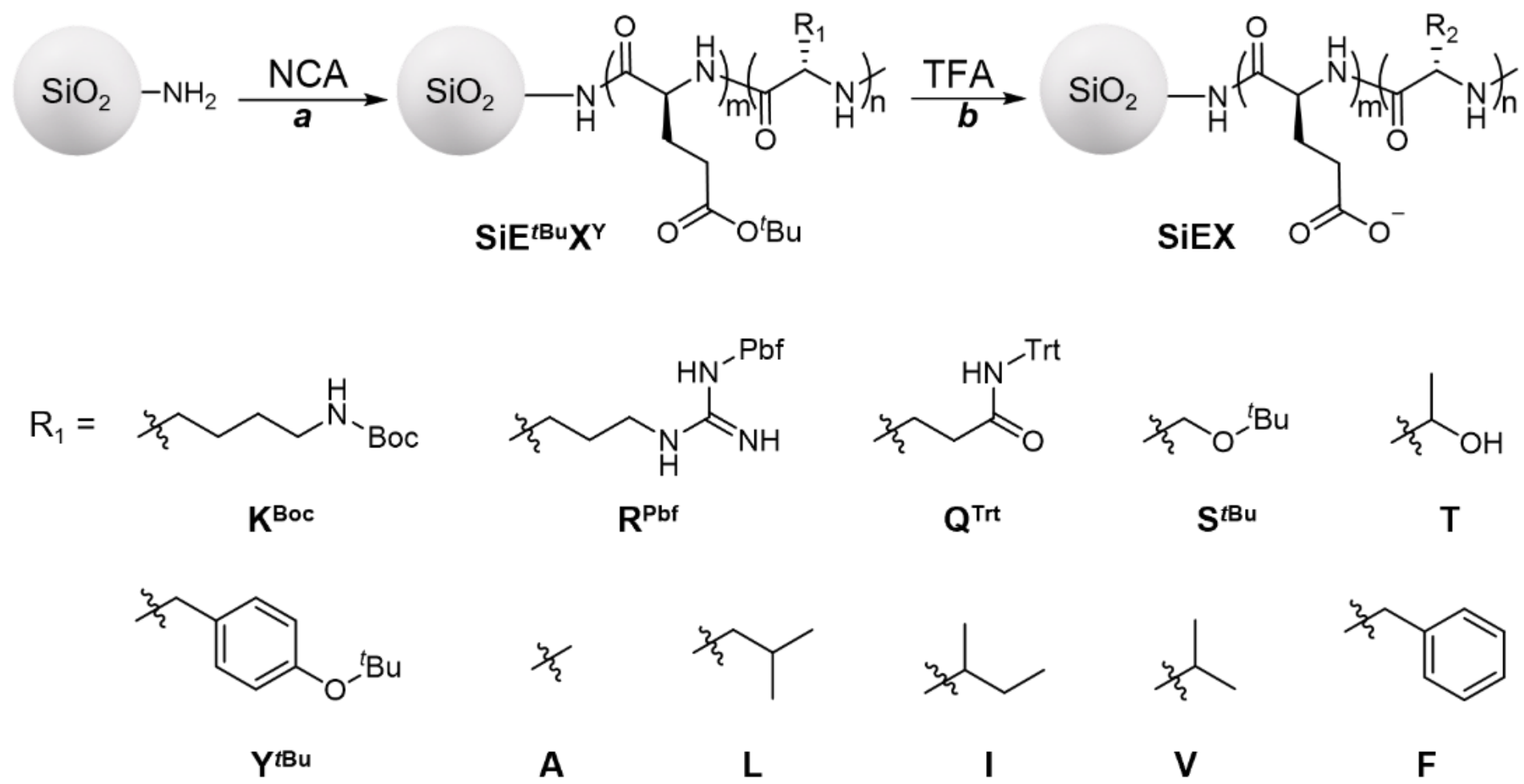

Supplementary Fig. 1. Synthetic route to SiEX NPs. ***a***. DMF/DCM, 1:1, v/v, rt. ***b***. DCM, 0 ºC, 2 h.

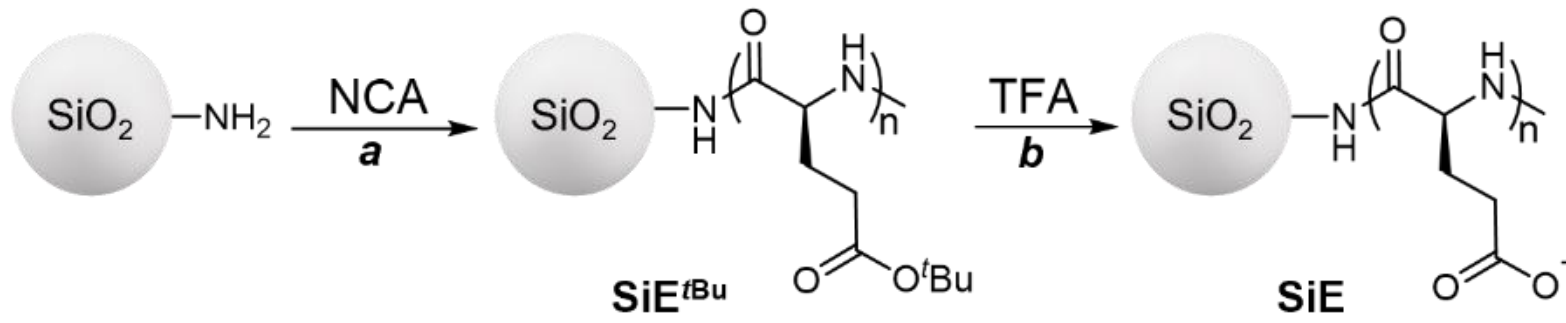

Supplementary Fig. 2. Synthetic route to SiE NPs. ***a***. DMF/DCM, 1:1, v/v, rt. ***b***. DCM, 0 ºC, 2 h.

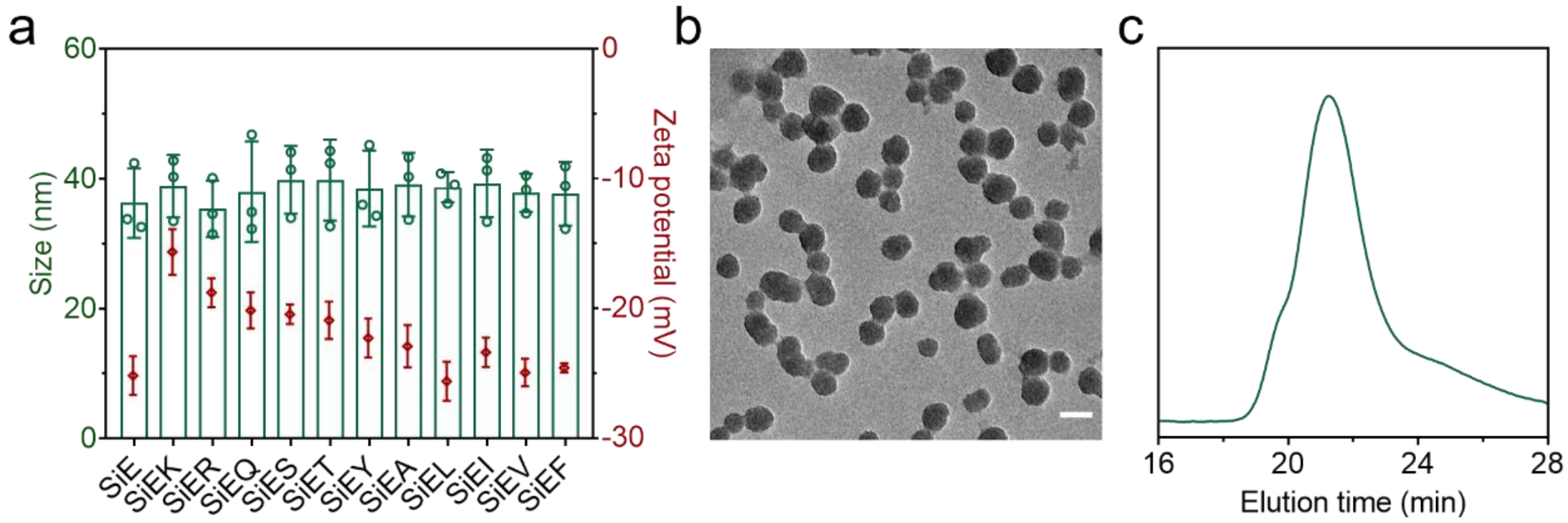

Supplementary Fig. 3. Characterization of heteropolypeptide-grafted nanoparticles (NPs). (a) Sizes and zeta potentials of various heteropolypeptide-modified silica NPs. Data are presented as the mean ± S.D. (n = 3 independent experiments). (b) Representative transmission electron microscopy (TEM) image of SiEV NPs. Scale bar = 50 nm. (c) Gel permeation chromatography-differential refractive index (GPC-dRI) trace of heteropolypeptides cleaved from silica NPs after surface-initiated ring-opening polymerization. PBLG-*co*-PVal was used as a model polypeptide. $M_n = 1.62 \times 10^5$ kDa, $Đ$ = 1.31, dn/dc = 0.087. Source data are provided as a Source Data file.

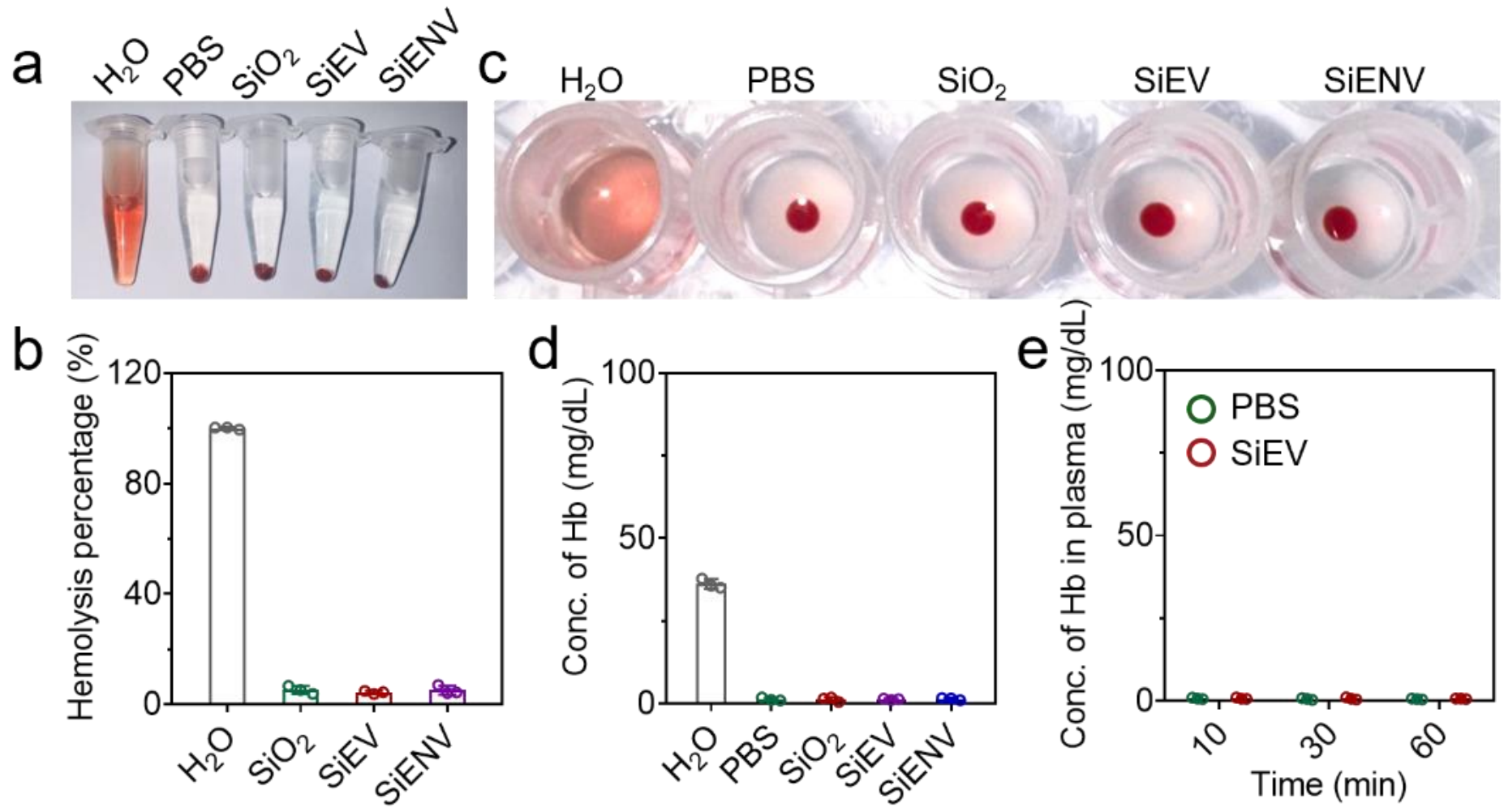

Supplementary Fig. 4. Hemocompatibility studies of SiO_2, SiEV, and SiENV. (a) Photograph showing the appearance of erythrocyte mixtures after treatment with various nanoparticles (NPs). (b) Hemolysis percentage of erythrocytes treated with various NPs. Data are presented as the mean ± S.D. (*n* = 3 biologically independent samples). (c) Red blood cell (RBC) agglutination assay of various NPs using a round-bottom well plate. (d) Release of hemoglobin (Hb) from RBCs after treatment with various NPs. Data are presented as the mean ± S.D. (*n* = 3 biologically independent samples). (e) Quantification of Hb level in plasma at different time post intravenous injection of SiEV. Data are presented as the mean ± S.D. (*n* = 3 female BALB/C mice per group, 6-8 weeks of age). H_2O and PBS were used as positive and negative controls, respectively. Source data are provided as a Source Data file.

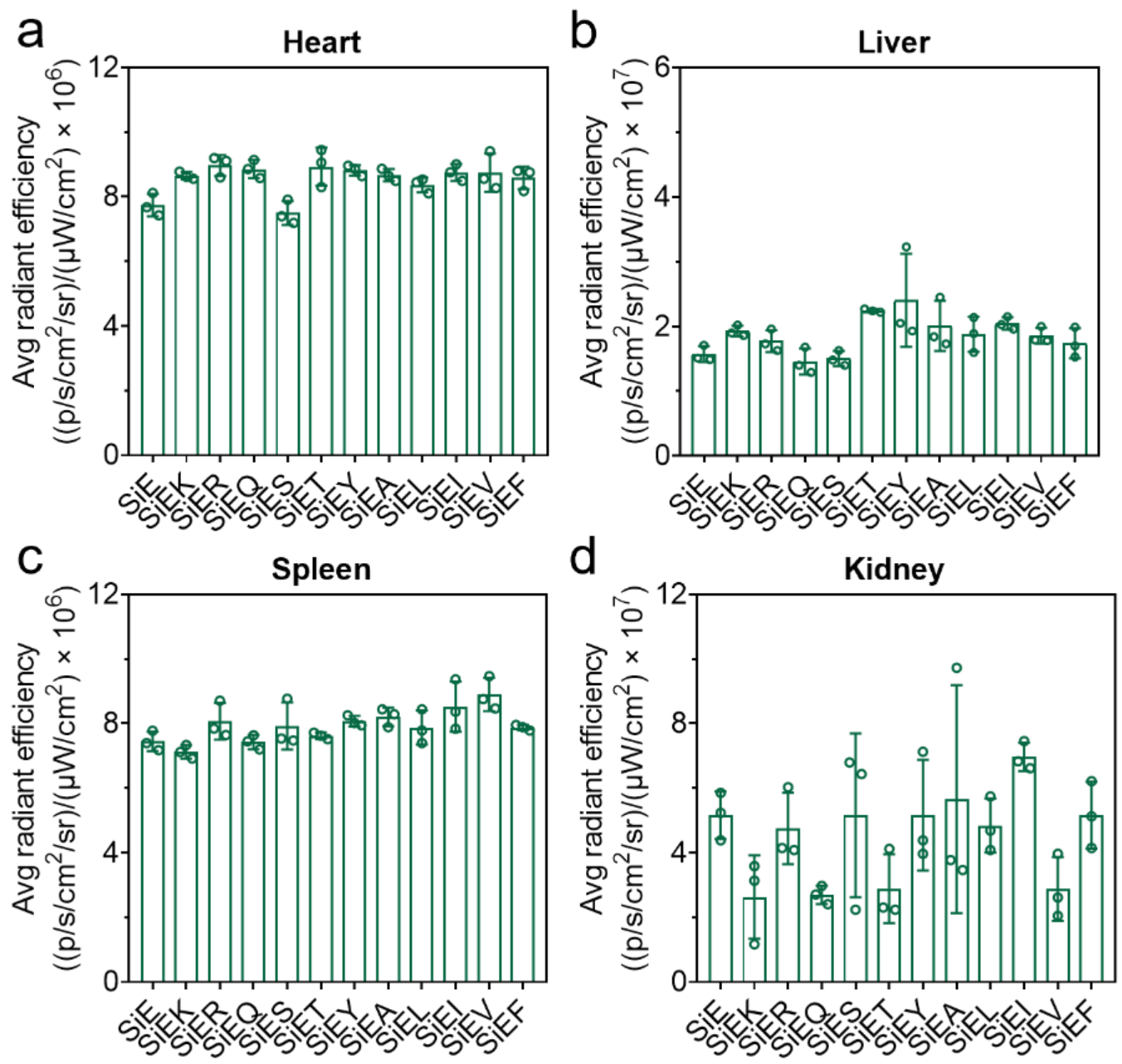

Supplementary Fig. 5. Semi-quantitative analysis of average radiant efficiency in heart (a), liver (b), spleen (c), and kidney (d) of mice treated with different heteropolypeptide-decorated silica nanoparticles at 4 h post intravenous injection. Data are presented as the mean ± S.D. (n = 3 female BALB/C mice per group, 6-8 weeks of age). Source data are provided as a Source Data file.

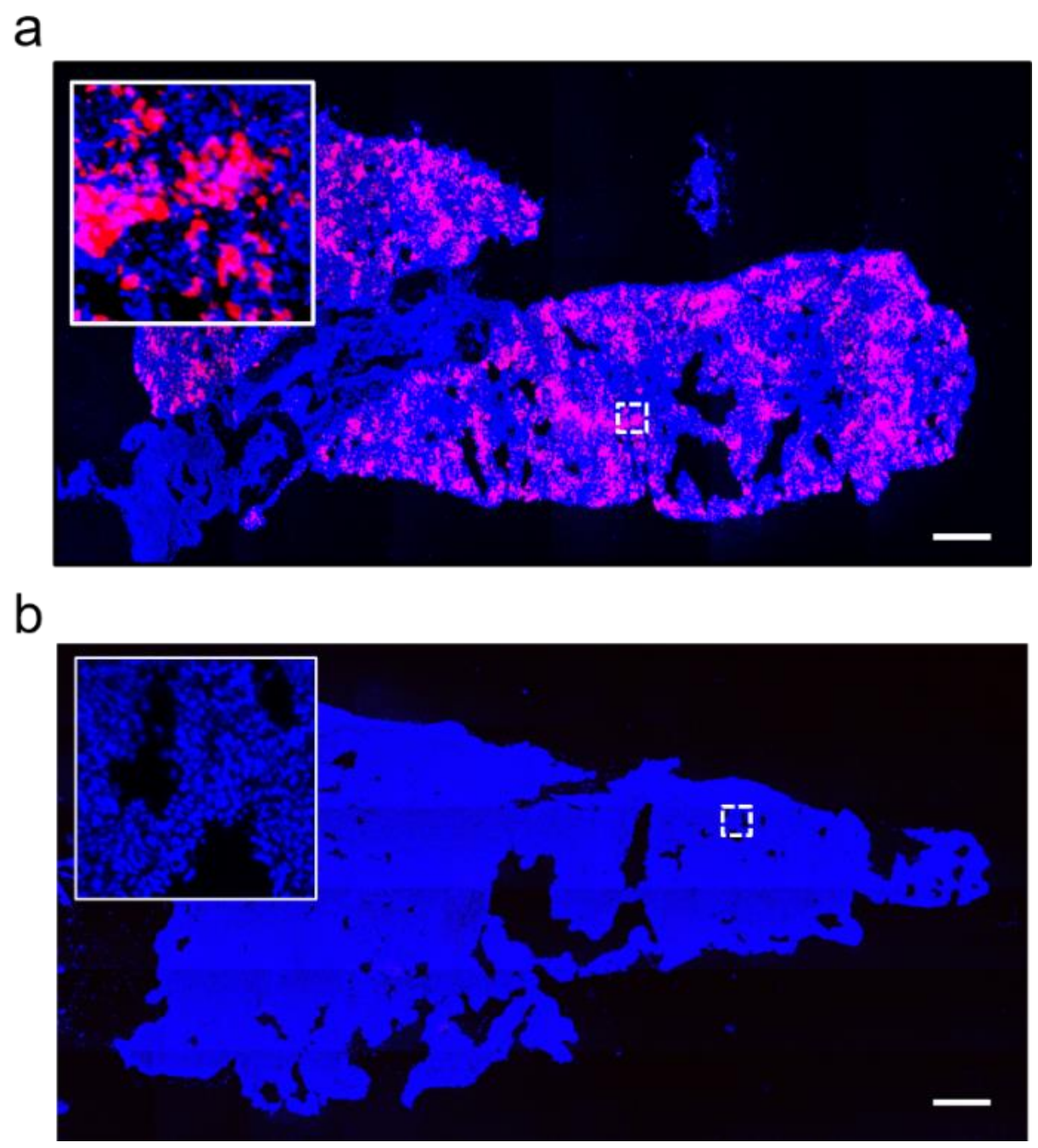

Supplementary Fig. 6. Confocal laser scanning microscopy image visualizing Cy5-labeled SiEV (a) or SiE (b) (red) in pulmonary tissues at 4 h post intravenous injection. The cell nucleus was stained with 4',6-diamidino-2-phenylindole (DAPI, blue). Scale bar = 500 μm.

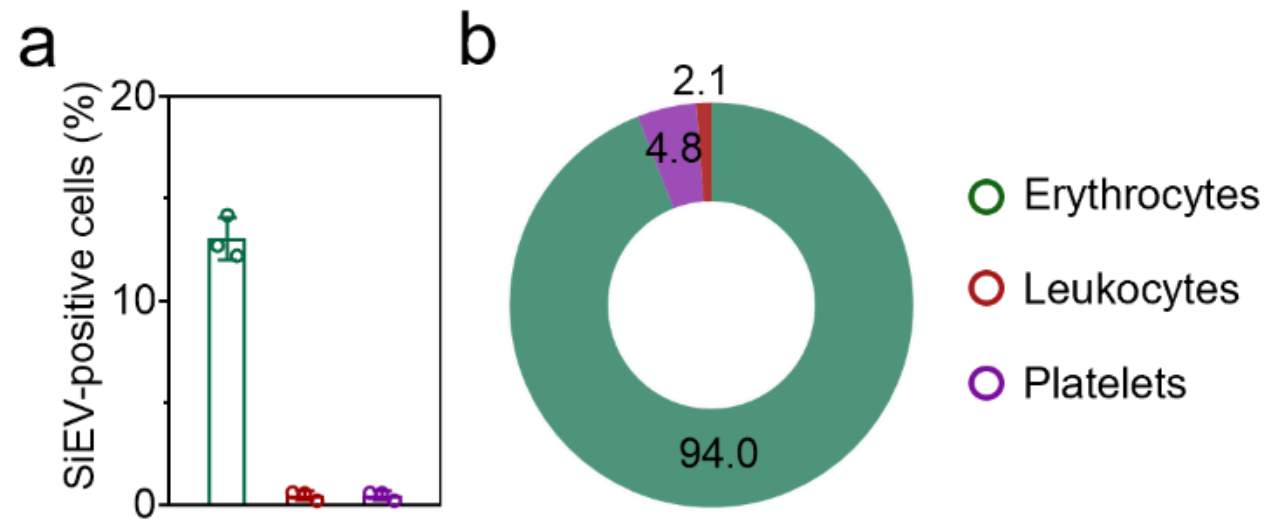

Supplementary Fig. 7. Flow cytometric analysis of the percentage of SiEV-positive cells in various blood cells (a) and the distribution of SiEV in various blood cells (b), including erythrocytes, leukocytes, and platelets. Data are presented as the mean ± S.D. (n = 3 female BALB/C mice per group, 6-8 weeks of age). Source data are provided as a Source Data file.

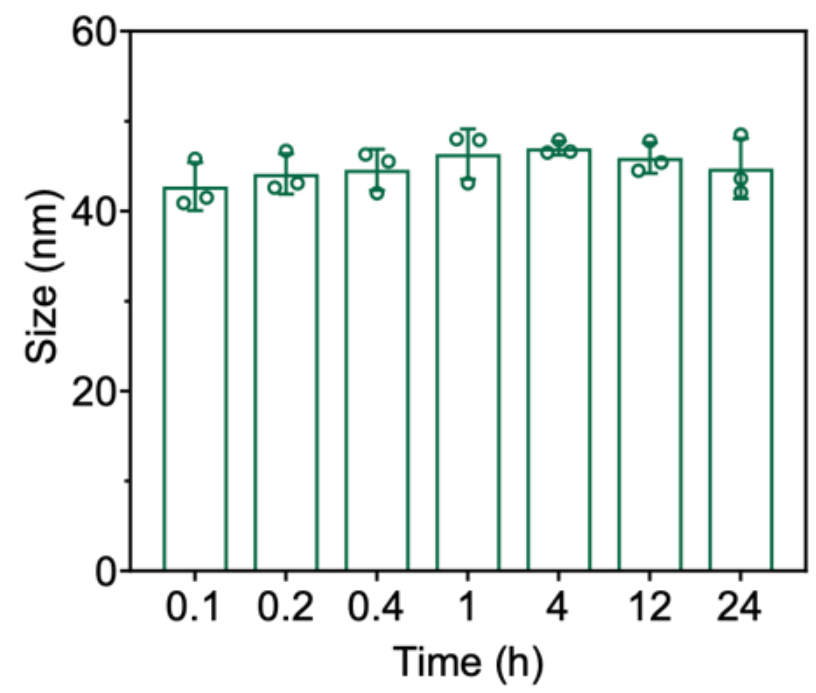

Supplementary Fig. 8. The stability of SiEV in plasma. Data are presented as the mean ± S.D. (n = 3 independent experiments). Source data are provided as a Source Data file.

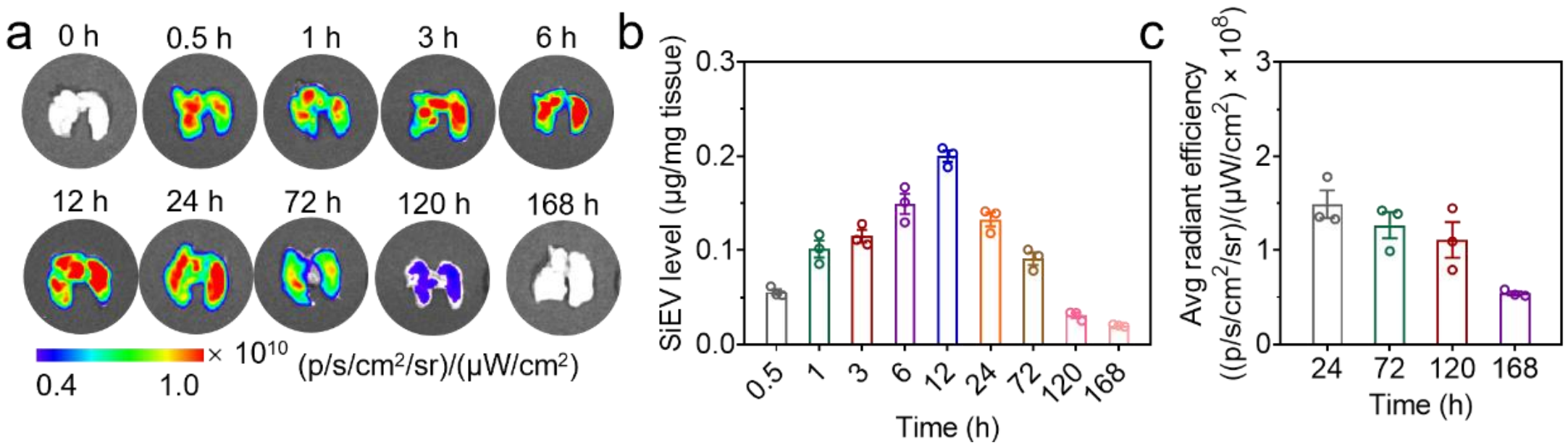

Supplementary Fig. 9. Retention of SiEV in lung tissues by fluorescent analysis. (a, b) *Ex vivo* fluorescence images of excised lungs (a) and corresponding quantitative analysis of SiEV levels in lung tissue homogenates (b) at different time points post intravenous (*i.v.*) administration. (c) Semi-quantitative analysis of SiEV levels in liver tissues at 24, 72, 120, and 168 h post *i.v.* administration. Data are presented as the mean ± S.D. (n = 3 female BALB/C mice per group, 6-8 weeks of age). Source data are provided as a Source Data file.

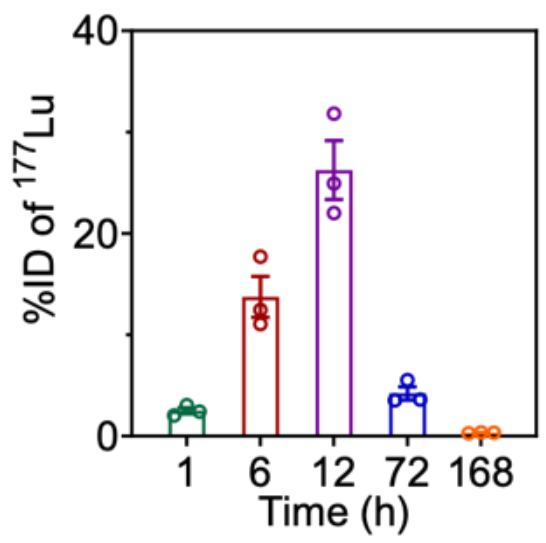

Supplementary Fig. 10. Quantitative analysis of SiEV levels in lung tissues at different time points post intravenous administration using radioisotope tracing. Data are presented as the mean ± S.D. (n = 3 female BALB/C mice per group, 6-8 weeks of age). Source data are provided as a Source Data file.

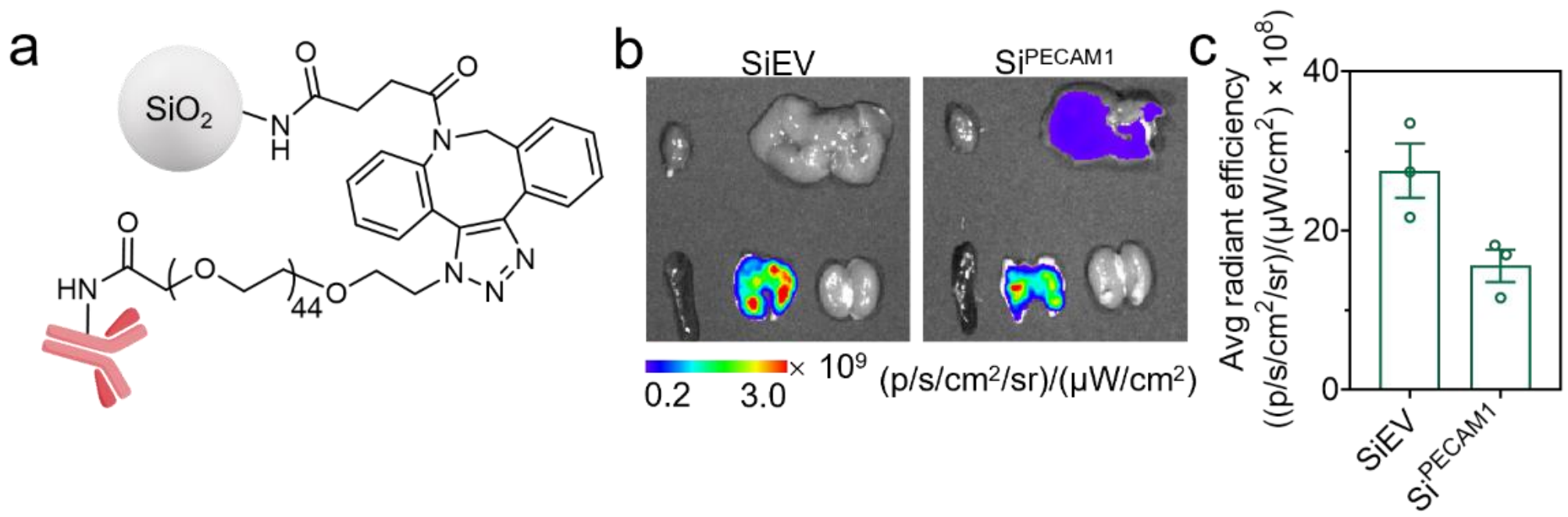

Supplementary Fig. 11. Lung-targeting ability of Si^{PECAM1}, antibody-decorated SiO_2 nanoparticles (NPs). (a) Chemical structure of Si^{PECAM1}. (b, c) *Ex vivo* fluorescence images of major organs (b) and corresponding semi-quantitative analysis of NP levels in lung tissues (c) at 12 h post intravenous injection of Cy5-labeled SiEV and Si$^{PECAM-1}$. Data are presented as the mean ± S.D. (n = 3 female BALB/C mice per group, 6-8 weeks of age). Source data are provided as a Source Data file.

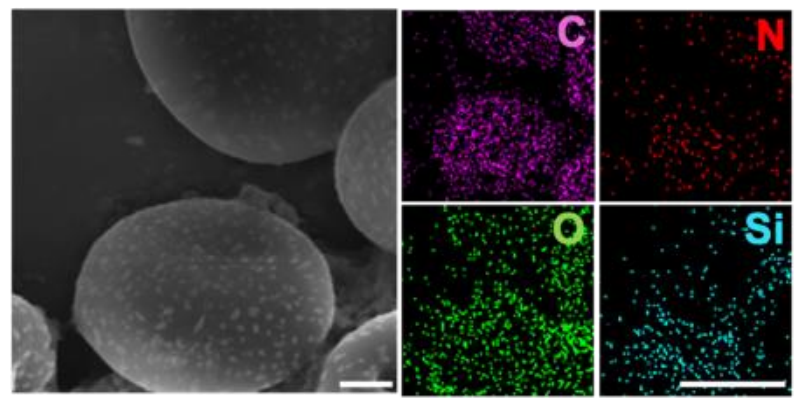

Supplementary Fig. 12. Scanning electron microscopy energy-dispersive X-ray spectroscopy (SEM-EDS) images of erythrocyte-SiEV complexes. Scale bar = 1 μm.

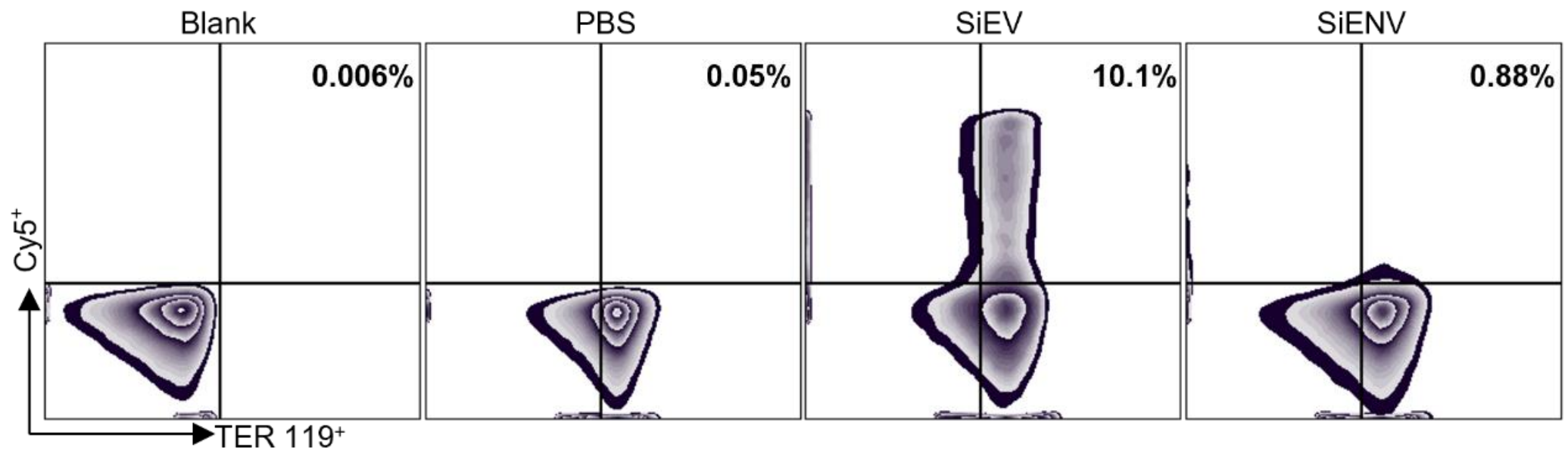

Supplementary Fig. 13. Flow cytometric analysis showing the adsorption of SiEV or SiENV on the membrane of red blood cells at 1 h post intravenous injection of Cy5-labeled NPs.

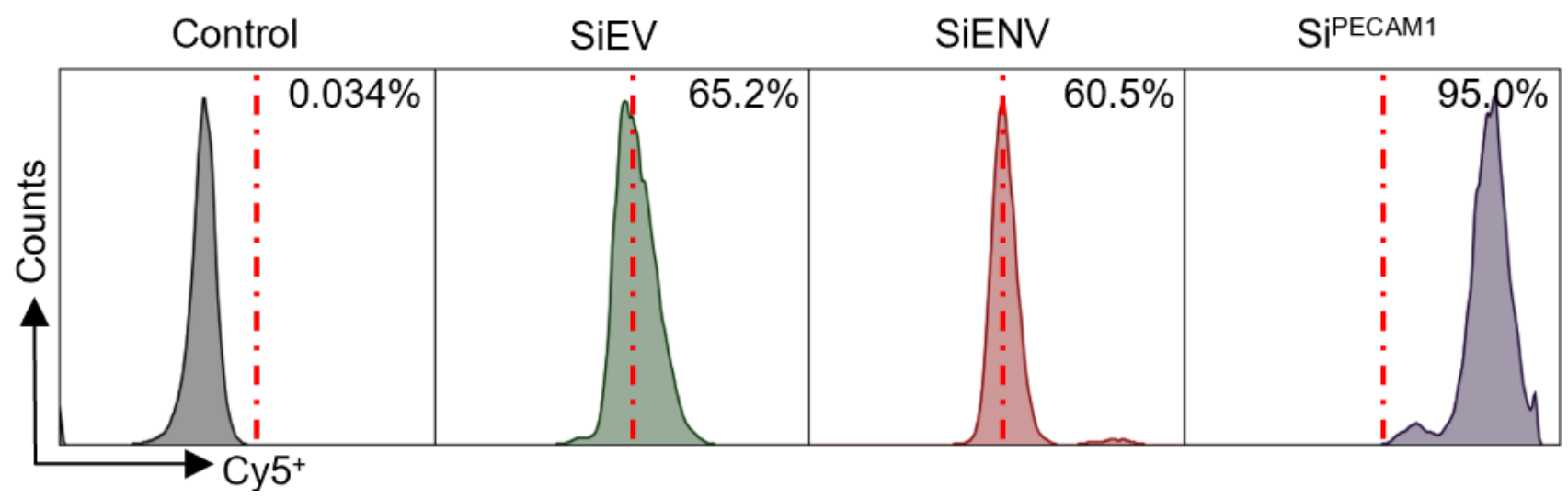

Supplementary Fig. 14. Cellular uptake of Cy5-labeled SiEV, SiENV, or Si^{PECAM1} by immortalized mouse pulmonary microvascular endothelial cells (iMPMVECs) by flow cytometry. The red dashed lines are gates to differentiate Cy5-negative and Cy5-positive cells.

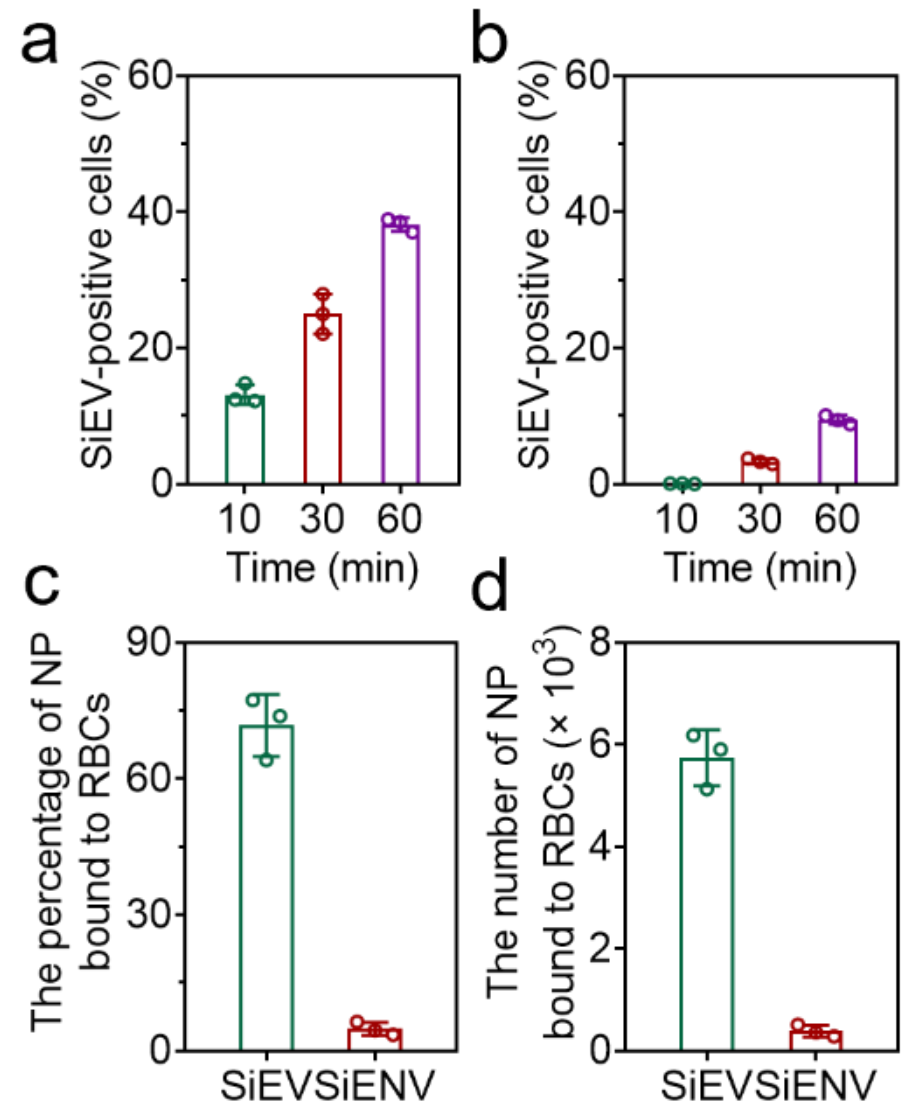

Supplementary Fig. 15. Complexation between red blood cells (RBCs) and SiEV nanoparticles (NPs). (a, b) Flow cytometric analysis showing the *in vitro* (a) (*n* = 3 biologically independent samples) or *in vivo* (b) (*n* = 3 female BALB/C mice per group, 6-8 weeks of age) adsorption kinetics of SiEV on the membrane of RBCs. Data are presented as the mean ± S.D. (c, d) The percentage (c) and the number (d) of NPs bound to RBCs after 1-h incubation with whole blood. Data are presented as the mean ± S.D. (*n* = 3 biologically independent samples). Source data are provided as a Source Data file.

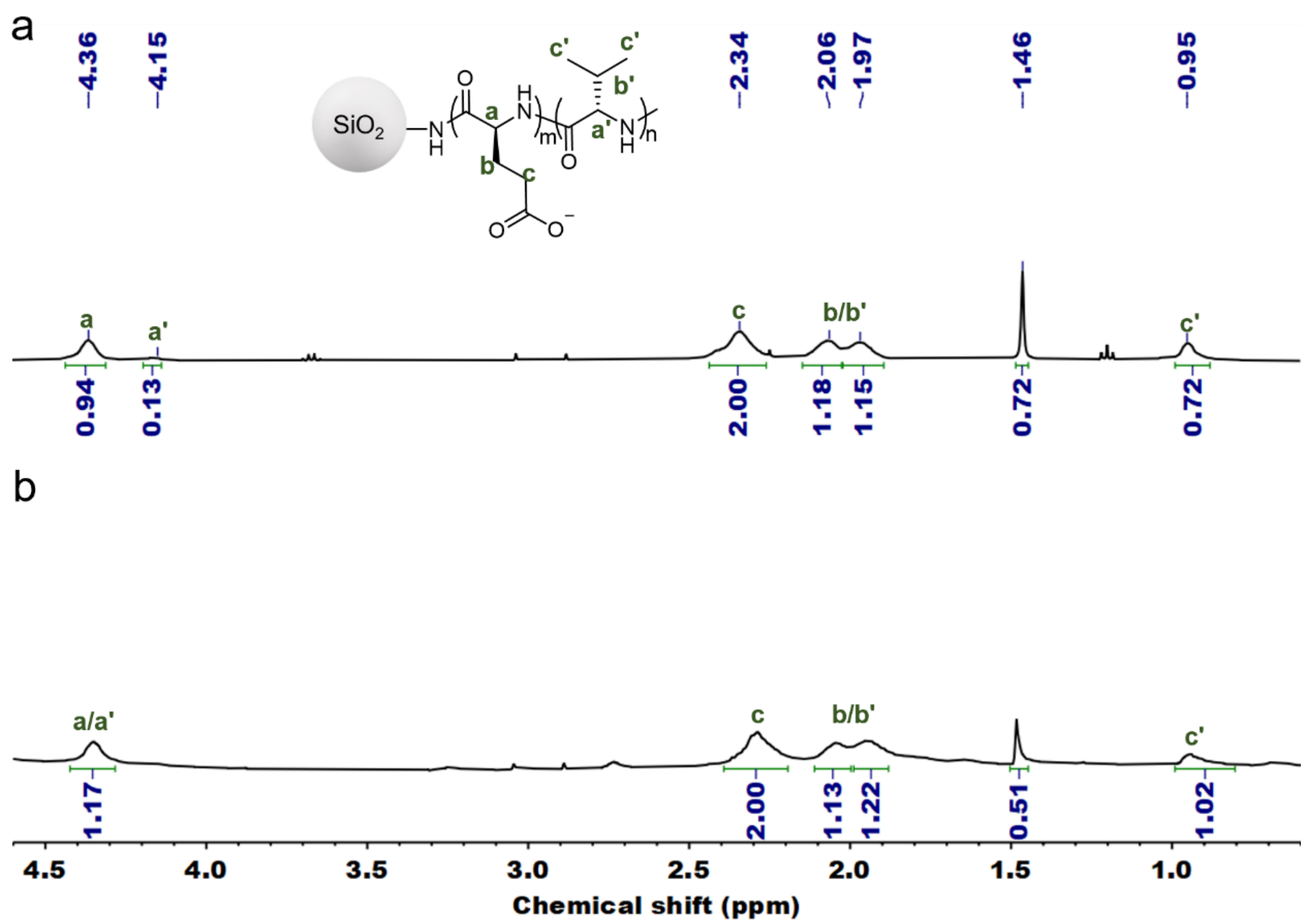

Supplementary Fig. 16. Overlaid ^{1}H NMR spectrum (400 MHz) of SiEV with valine content of 10% (a) or 20% (b) in D_2O.

Compared to the NMR spectra in TFA (assuming complete dissolution), the side-chain valine signals ($\delta = 0.95$ ppm) might be weaker depending on chain length and concentration. The solvent exposure of the hydrophobic segments is disfavored, leading to decreased solvation in aqueous environment.

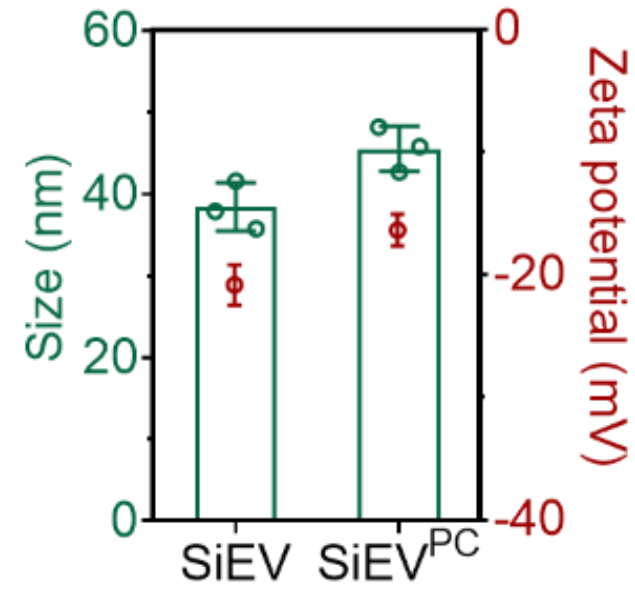

Supplementary Fig. 17. Sizes and zeta potentials of SiEV and $SiEV^{PC}$. Data are presented as the mean ± S.D. (n = 3 independent experiments). Source data are provided as a Source Data file.

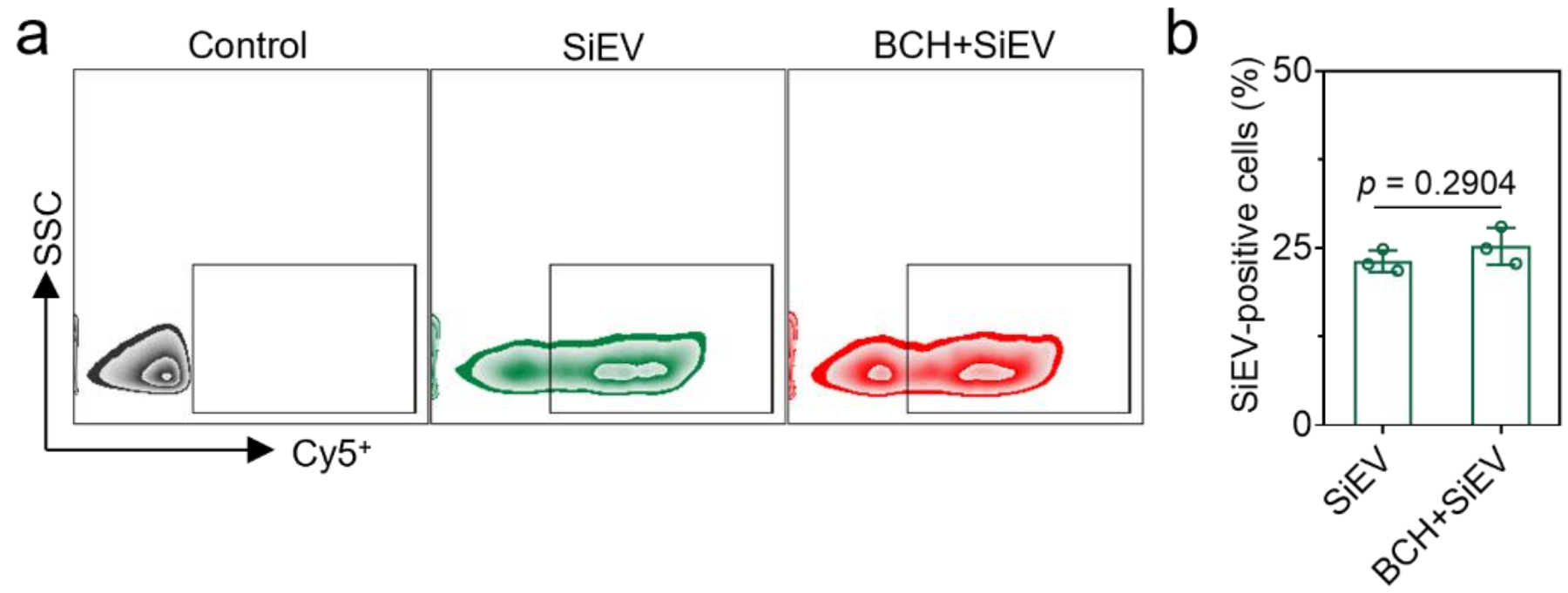

Supplementary Fig. 18. Flow cytometric analysis (a) and the corresponding quantification of SiEV-positive cells (b) of red blood cells after SiEV treatment in the presence or absence of 2-amino-2-norbornanecarboxylic acid (BCH), an inhibitor for L-type amino acid transporter 1 (LAT1). Data are presented as the mean ± S.D. ($n = 3$ biologically independent samples). Statistical analysis was calculated using a two-tailed unpaired *t*-test. Source data are provided as a Source Data file.

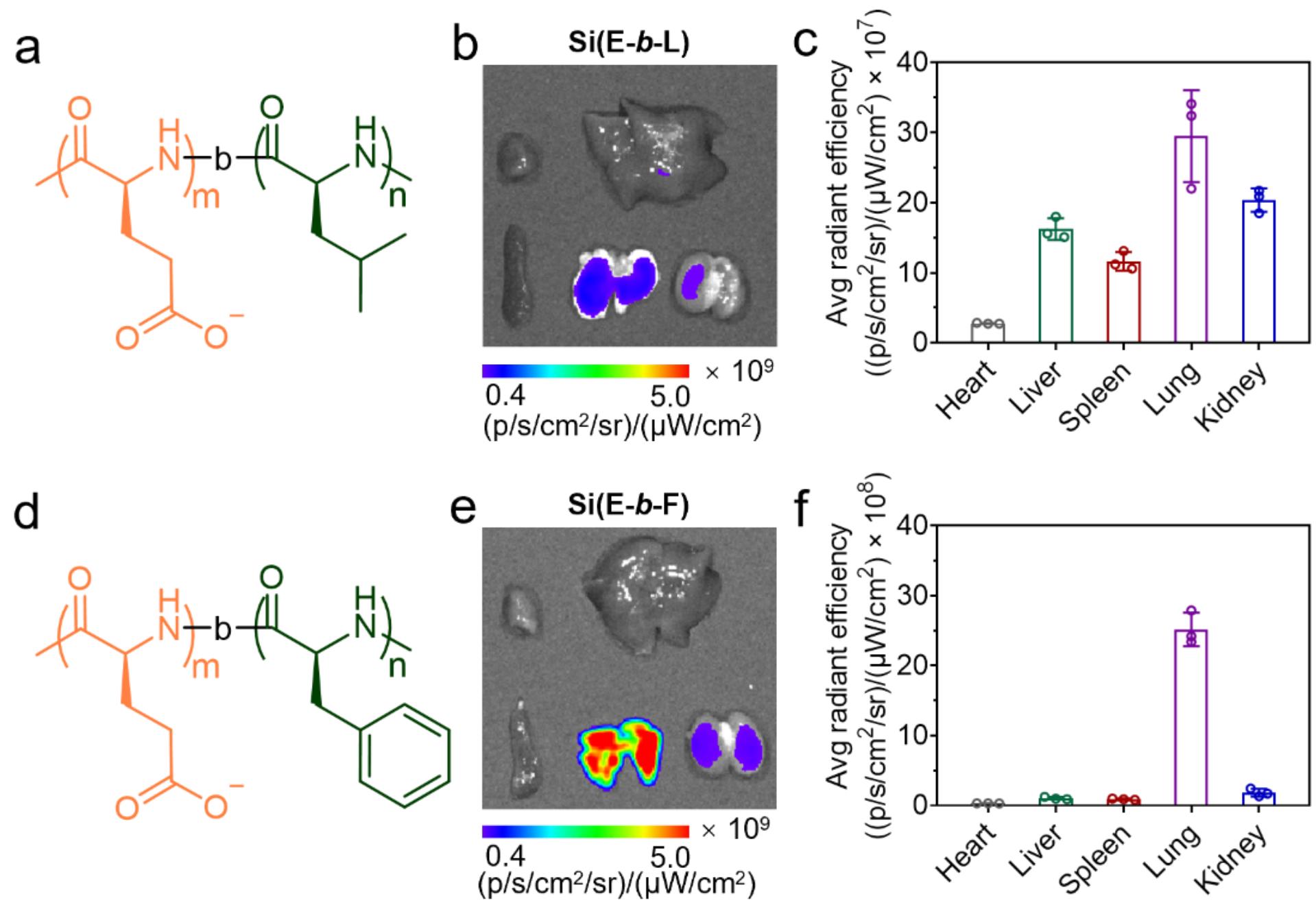

Supplementary Fig. 19. The lung-targeting ability of additional SiO_2 nanoparticles decorated with diblock copolypeptides with hydrophobic outer layers. Chemical structure (a, d), *ex vivo* fluorescence images of major organs (b, e), and corresponding semi-quantitative analysis of accumulation in major organs (c, f) of Si(E-*b*-L) and Si(E-*b*-F) decorated with block copolypeptides. Data are presented as the mean ± S.D. (n = 3 female BALB/C mice per group, 6-8 weeks of age). Source data are provided as a Source Data file.

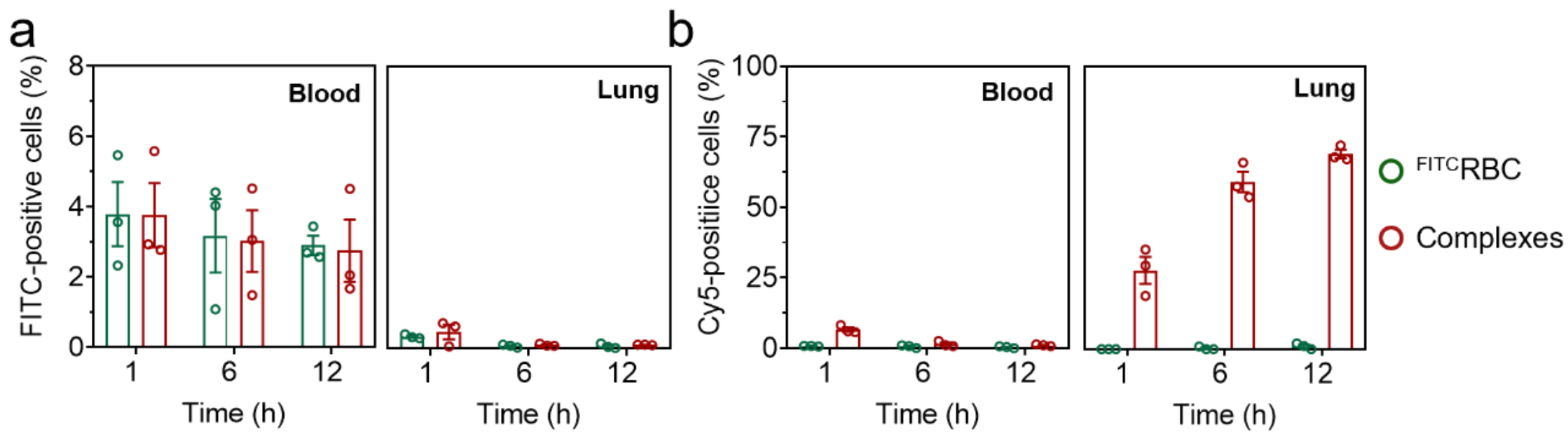

Supplementary Fig. 20. Flow cytometric analysis of the percentage of FITC-positive (a) or Cy5-positive cells (b) in blood and lung tissues at different time points post intravenous injection of fluorescently labeled red blood cell (RBC)-SiEV complexes. RBCs and SiEV were labeled with FITC and Cy5 before *in vitro* complex formation, respectively. Data are presented as the mean ± S.D. (*n* = 3 female BALB/C mice per group, 6-8 weeks of age). Source data are provided as a Source Data file.

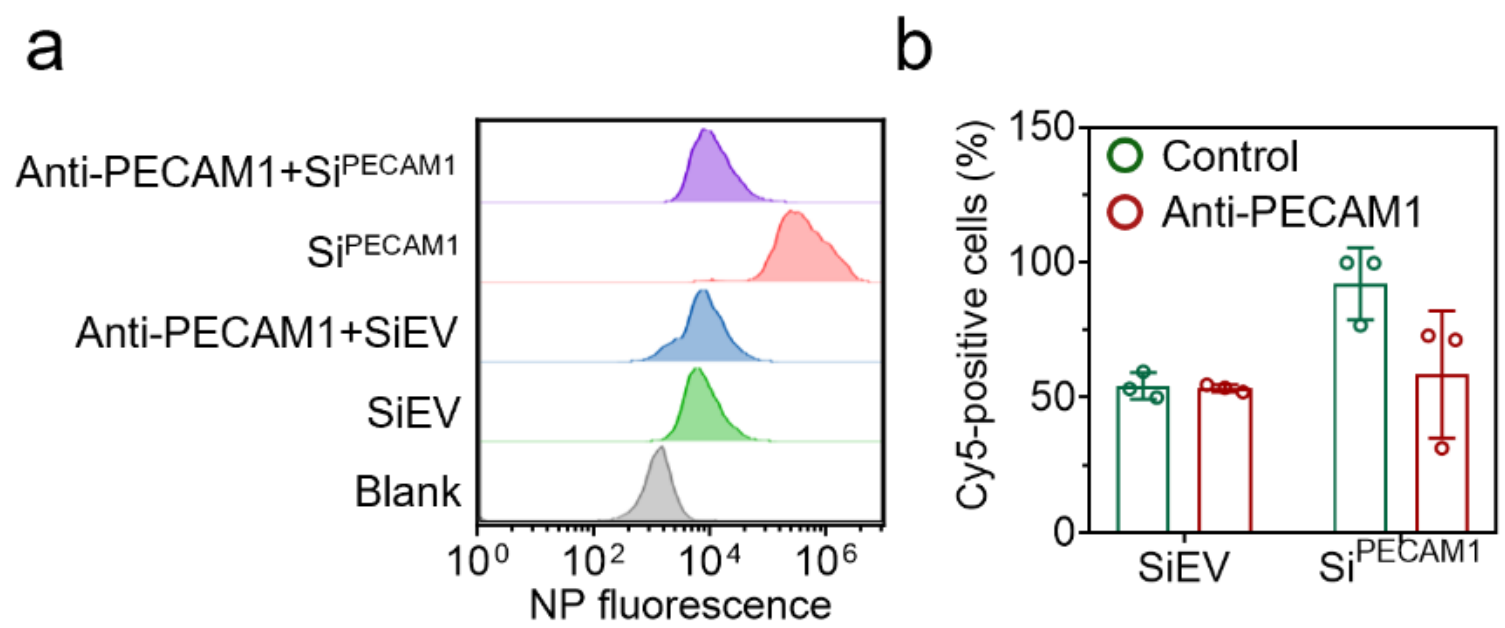

Supplementary Fig. 21. Cellular uptake (a) and the corresponding quantification of Cy5-positive cells (b) of Cy5-labeled SiEV or Si^{PECAM1} nanoparticles (NPs) by immortalized mouse pulmonary microvascular endothelial cells (iMPMVECs) after blocking PECAM1 with corresponding antibodies, as analyzed by flow cytometry. Data are presented as the mean ± S.D. ($n = 3$ biologically independent samples). Source data are provided as a Source Data file.

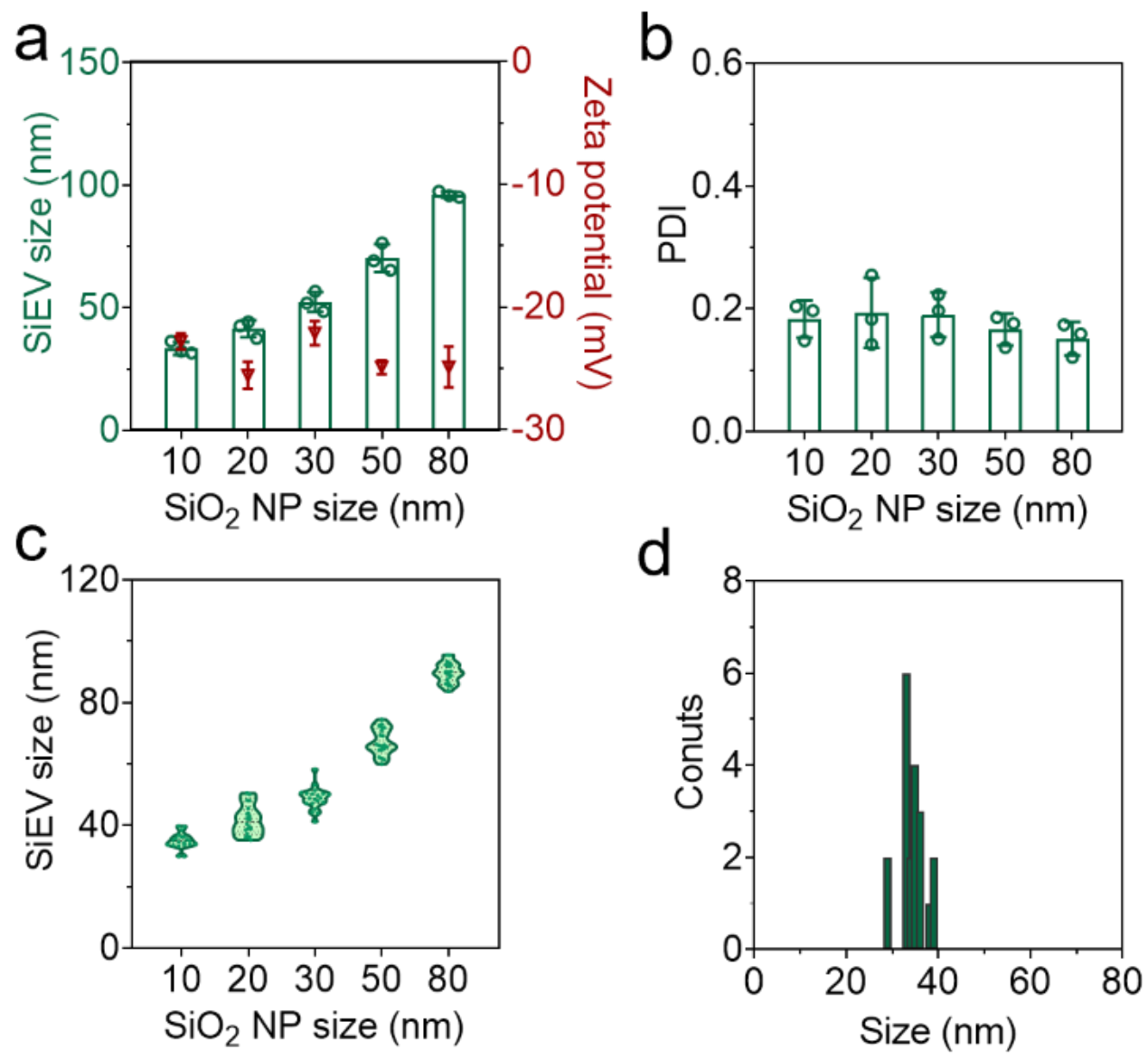

Supplementary Fig. 22. Characterization of various SiEV nanoparticles (NPs) with different sizes of initial silica NPs. (a, b) The sizes, zeta potentials (a) and polydispersity indexes (PDI) (b) of various SiEV NPs. Data are presented as the mean ± S.D. (n = 3 independent experiments). (c) The sizes of various SiEV NPs counted from transmission electron microscopy (TEM) images. Data are presented as the mean ± S.D. (n = 20 independent measurements of individual NPs from five to ten TEM images). (d) The size distribution of SiEV with 10-nm initial silica NPs. Source data are provided as a Source Data file.

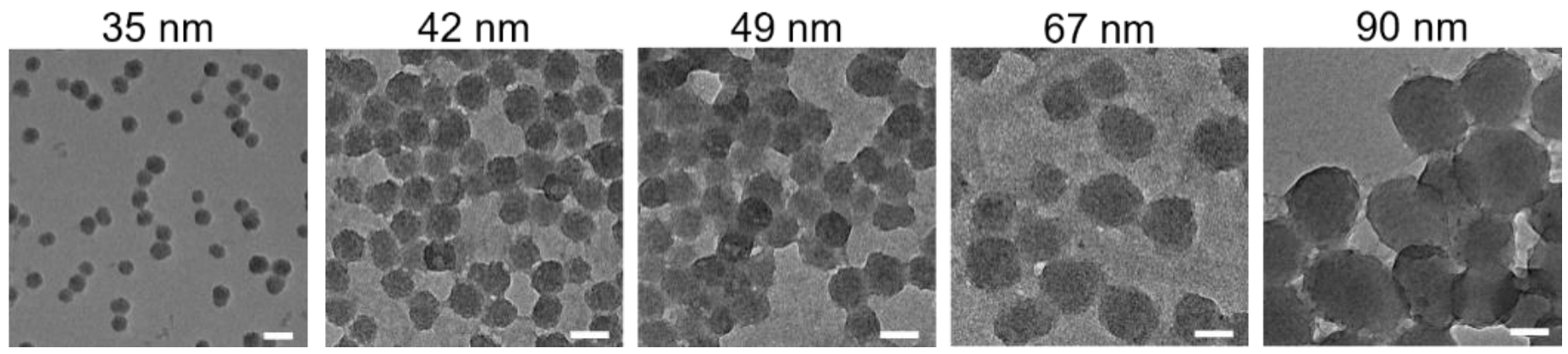

Supplementary Fig. 23. Transmission electron microscopy (TEM) images of various SiEV nanoparticles (NPs) with different sizes of initial silica NPs. Scale bar = 50 nm.

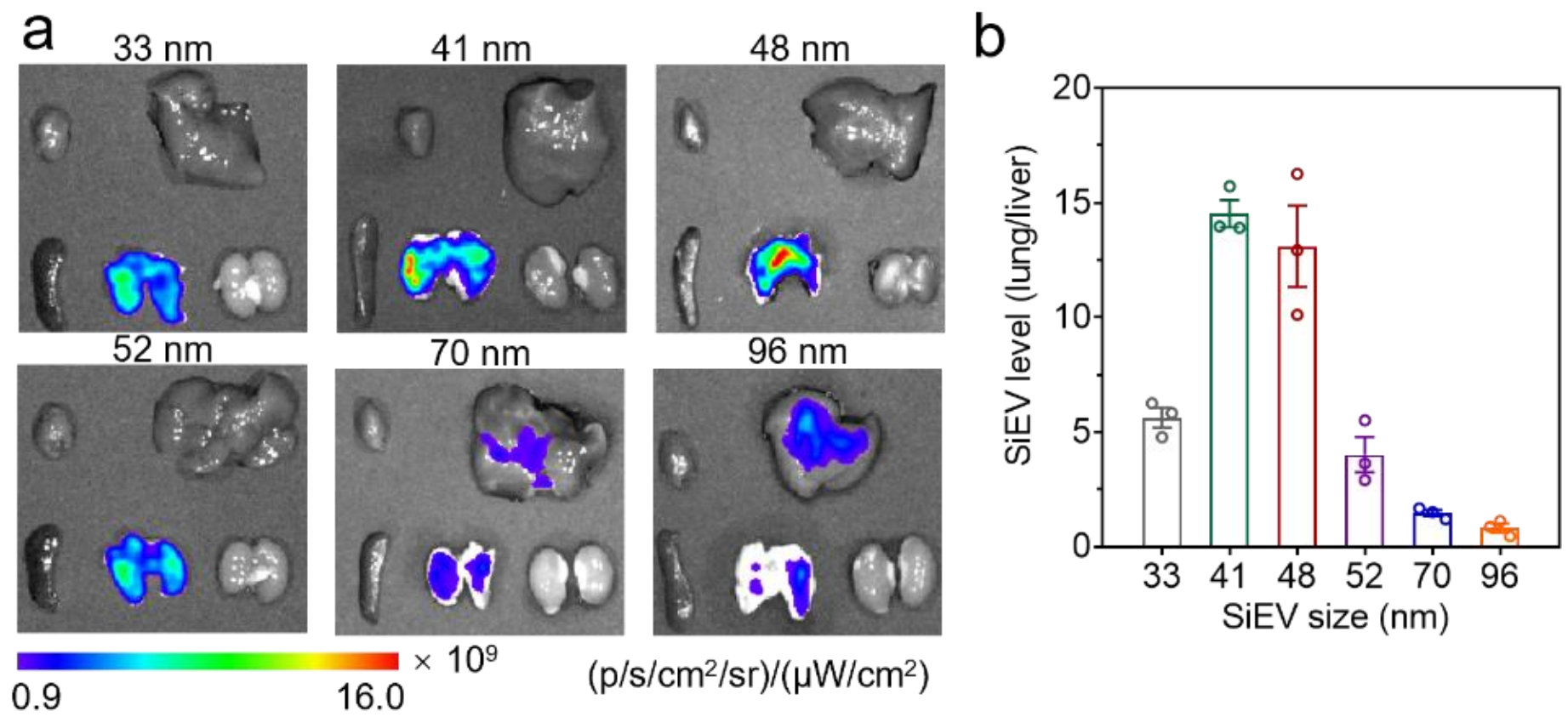

Supplementary Fig. 24. *Ex vivo* fluorescence images of the major organs of mice (a) and the corresponding quantitative analysis of lung-to-liver ratio (b) after tissue homogenization, filtration, and red blood cell lysis at 12 h post intravenous injection of SiEV with different sizes. Data are presented as the mean ± S.D. (n = 3 female BALB/C mice per group, 6-8 weeks of age). Source data are provided as a Source Data file.

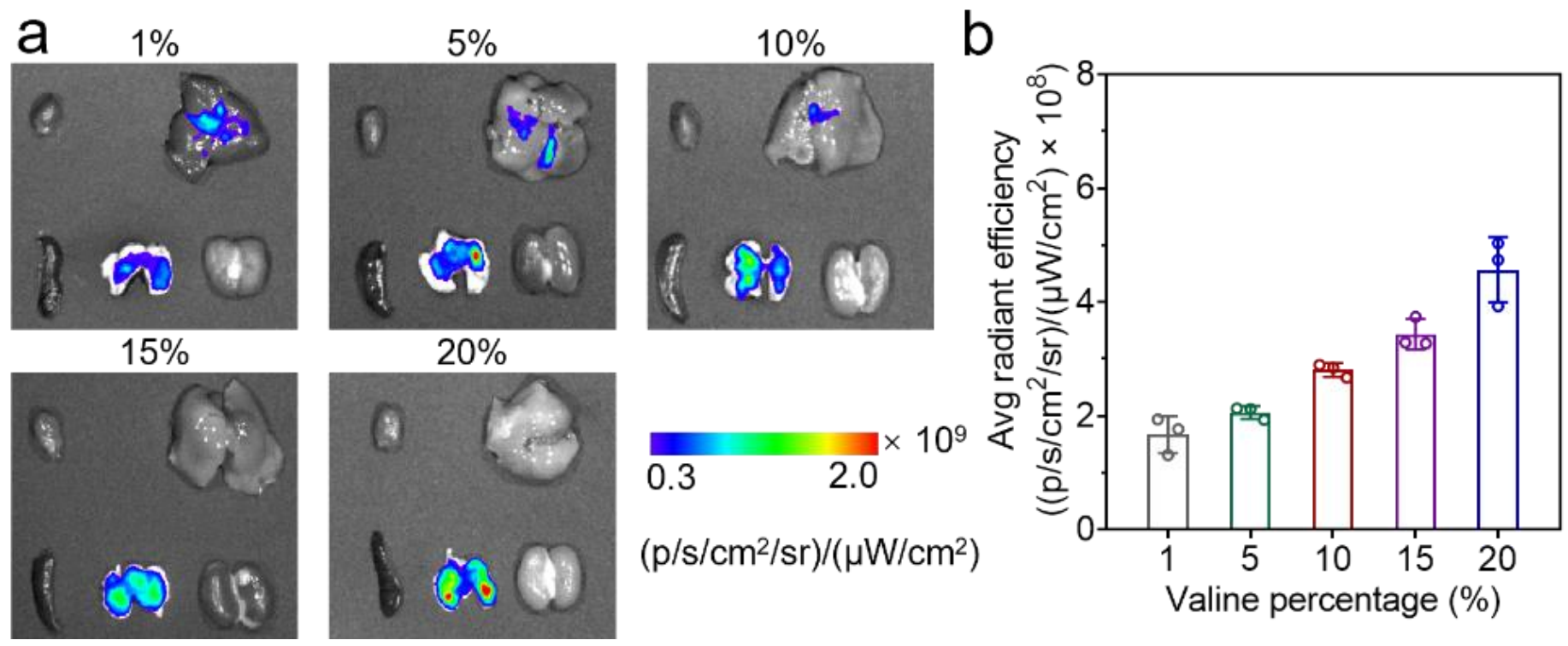

Supplementary Fig. 25. *Ex vivo* fluorescence images of the major organs of mice (a) and the corresponding semi-quantitative analysis of SiEV levels in lung tissues (b) at 12 h post intravenous injection of SiEV with different valine percentages. Data are presented as the mean ± S.D. (n = 3 female BALB/C mice per group, 6-8 weeks of age). Source data are provided as a Source Data file.

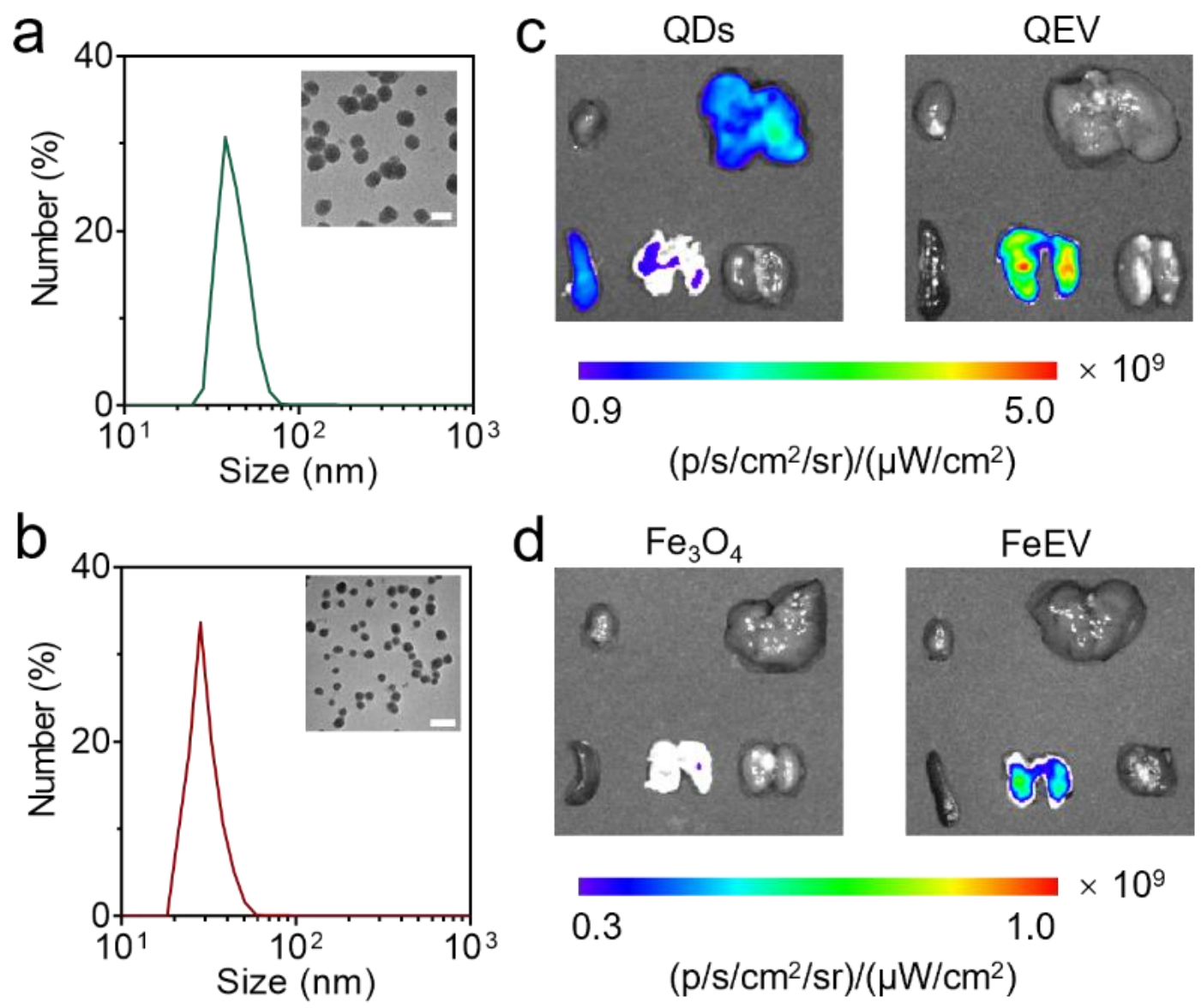

Supplementary Fig. 26. Development of various lung-targeting nanoparticles (NPs) through surface grafting of heteropolypeptides. (a, b) Size distribution and transmission electron microscopy (TEM) images of Cy5-labeled heteropolypeptide-grafted NPs, including PEV-decorated quantum dot (QEV) (a) and ferric oxide (FeEV) (b). Scale bar = 50 nm. (c, d) *Ex vivo* fluorescence images of major organs of mice at 12 h post intravenous injection of Cy5-labeled heteropolypeptide-grafted NPs, including QEV (c) and FeEV (d). Cy5-labeled bare NPs without heteropolypeptide grafting was used as controls in Figs. c and d. Source data are provided as a Source Data file.

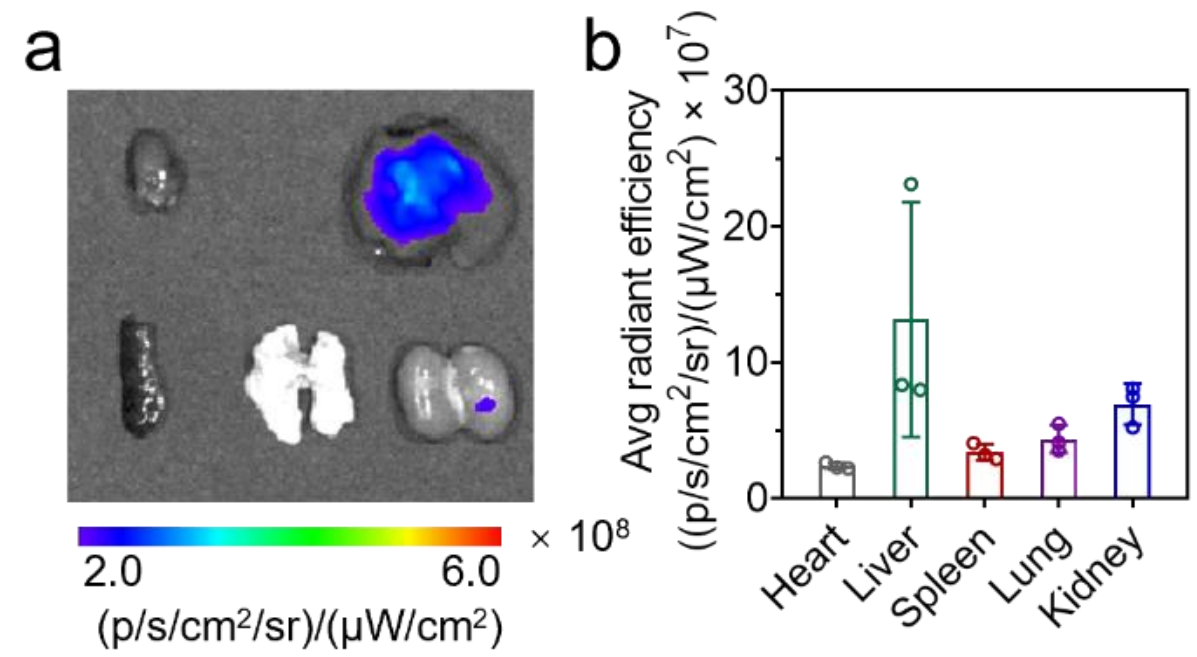

Supplementary Fig. 27. Biodistribution of linear heteropolypeptide PEV. *Ex vivo* fluorescent images of major organs of mice (a) and corresponding semi-quantitative analysis of polymer accumulations in the major organs (b) at 12 h after intravenous injection of Cy5-labeled PEV. Data are presented as the mean ± S.D. ($n = 3$ female BALB/C mice per group, 6-8 weeks of age). Source data are provided as a Source Data file.

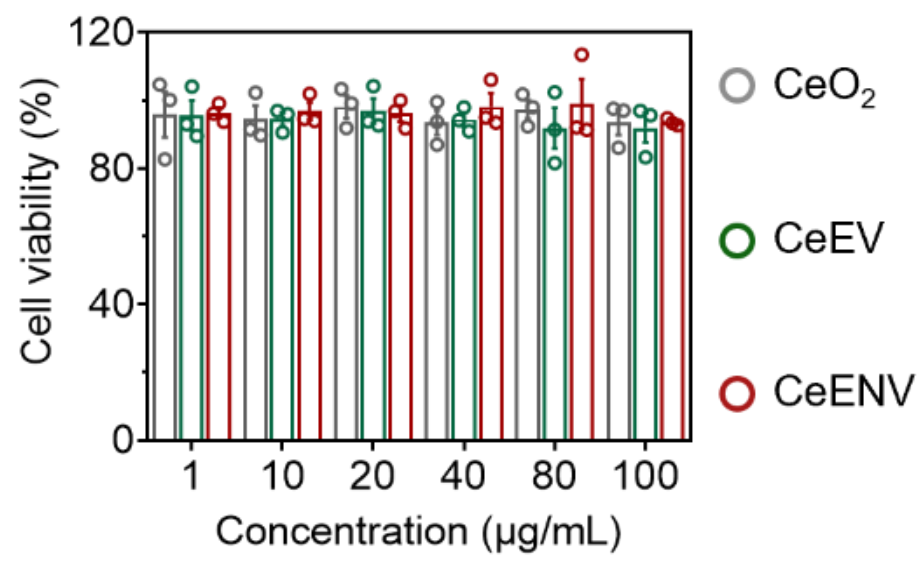

Supplementary Fig. 28. Viability of RAW 264.7 cells after 4-h incubation with CeO_2, CeEV, or CeENV. Data are presented as the mean ± S.D. (*n* = 3 biologically independent samples). Source data are provided as a Source Data file.

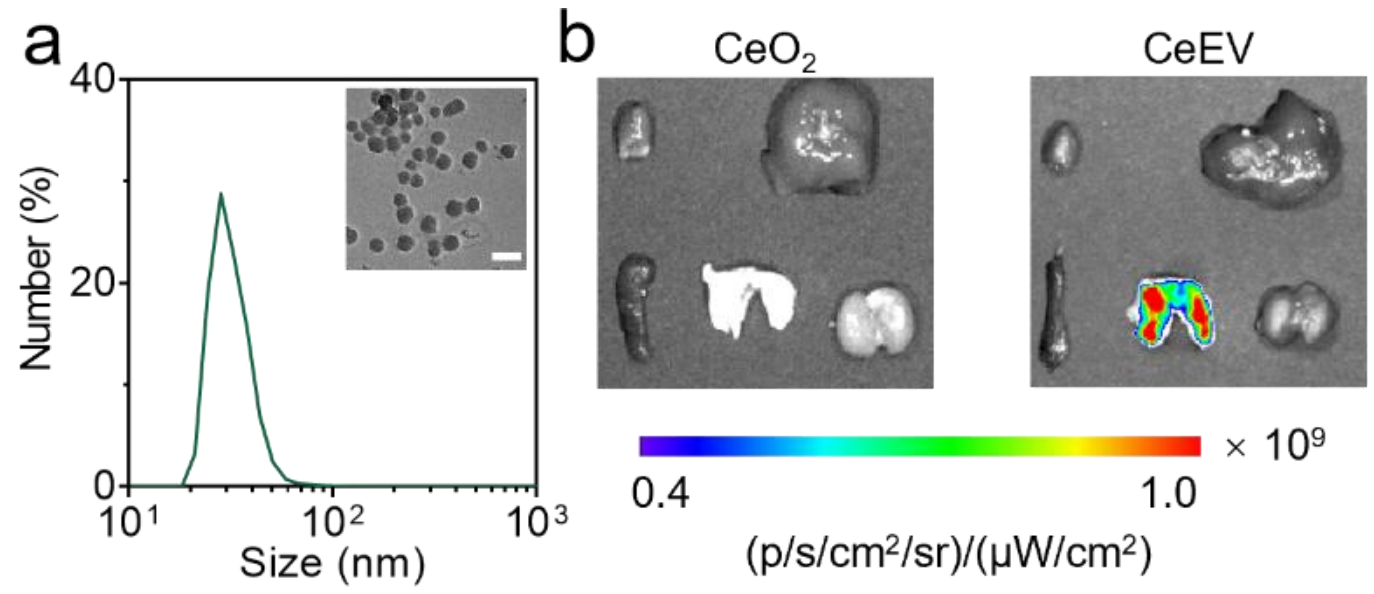

Supplementary Fig. 29. Characterization and lung-targeting of CeEV. (a) Size distribution and transmission electron microscopy (TEM) image of CeEV. Scale bar = 50 nm. (b) *Ex vivo* fluorescence images of major organs of mice at 12 h post intravenous injection Cy5-labeled CeO_2 or CeEV. Source data are provided as a Source Data file.

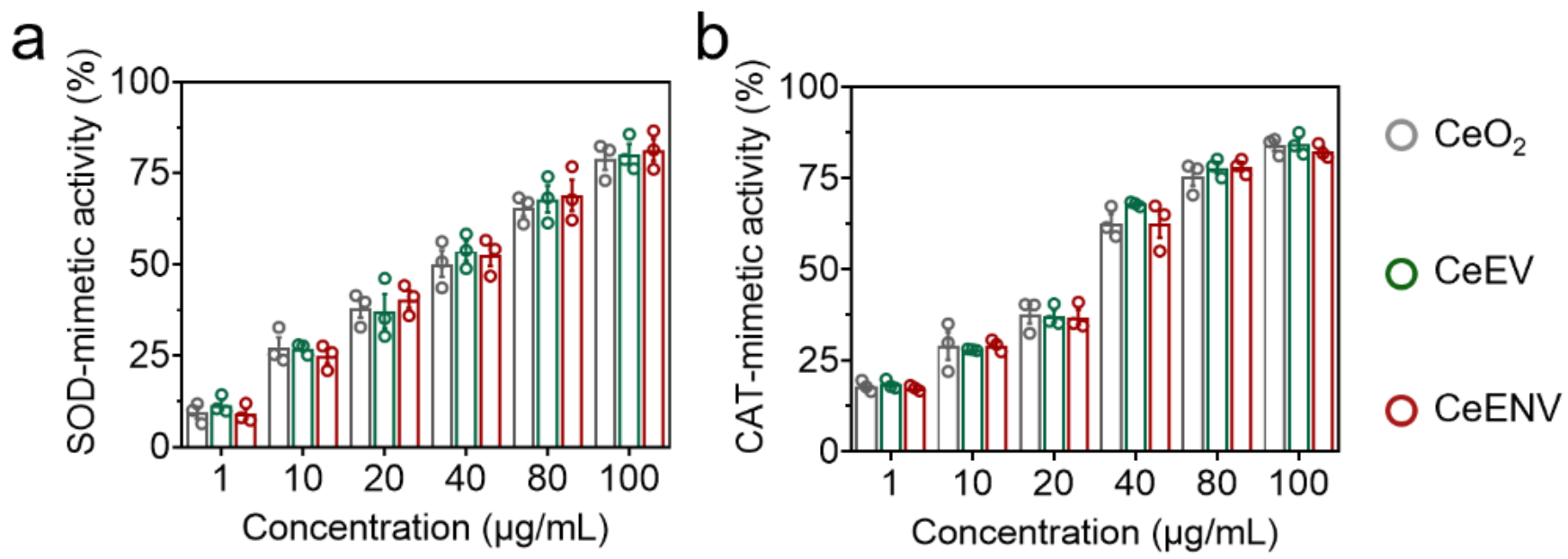

Supplementary Fig. 30. Determination of superoxide dismutase (SOD)-mimetic activity (a) and catalase (CAT)-mimetic activity (b) of CeO_2, CeEV, and CeENV. Data are presented as the mean ± S.D. (n = 3 independent experiments). Source data are provided as a Source Data file.

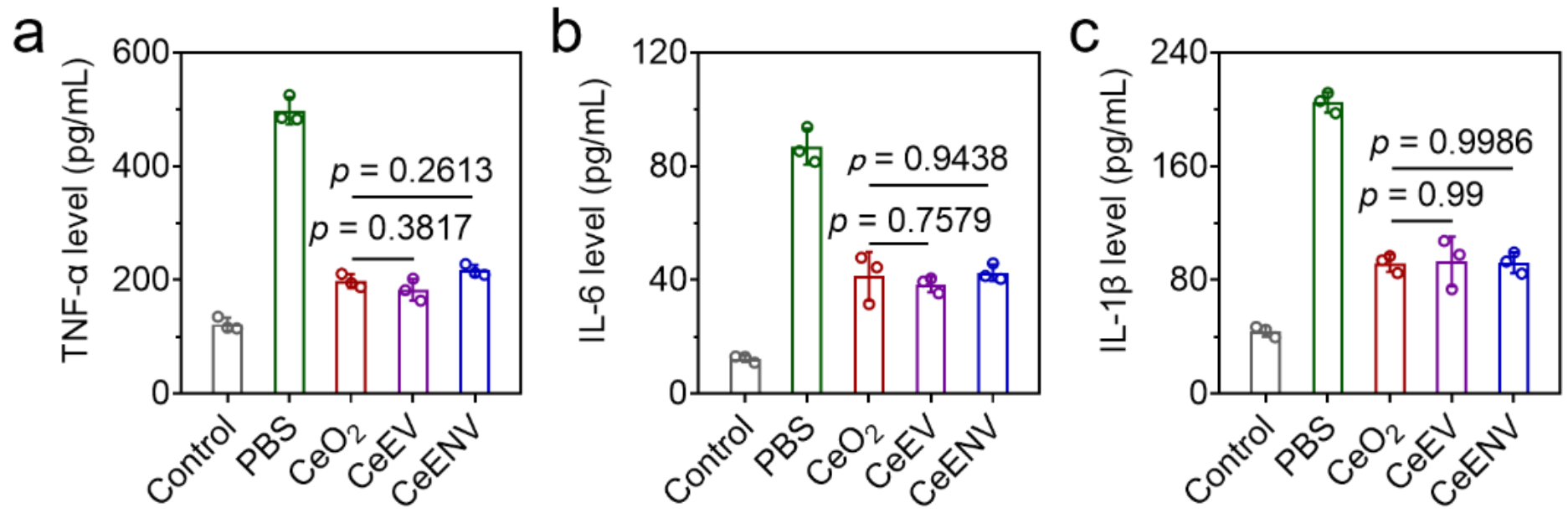

Supplementary Fig. 31. Levels of secreted tumor necrosis factor-α (TNF-α) (a), interleukin-6 (IL-6) (b), and interleukin-1β (IL-1β) (c) from RAW 264.7 cells stimulated with lipopolysaccharide (LPS) followed by the treatment with PBS, CeO_2, CeEV, or CeENV. Data are presented as the mean ± S.D. (n = 3 biologically independent samples). Cells without LPS stimulation or nanoparticle treatment served as the control. Statistical analysis was calculated *via* one-way ANOVA with Dunnett's multiple comparisons test. Source data are provided as a Source Data file.

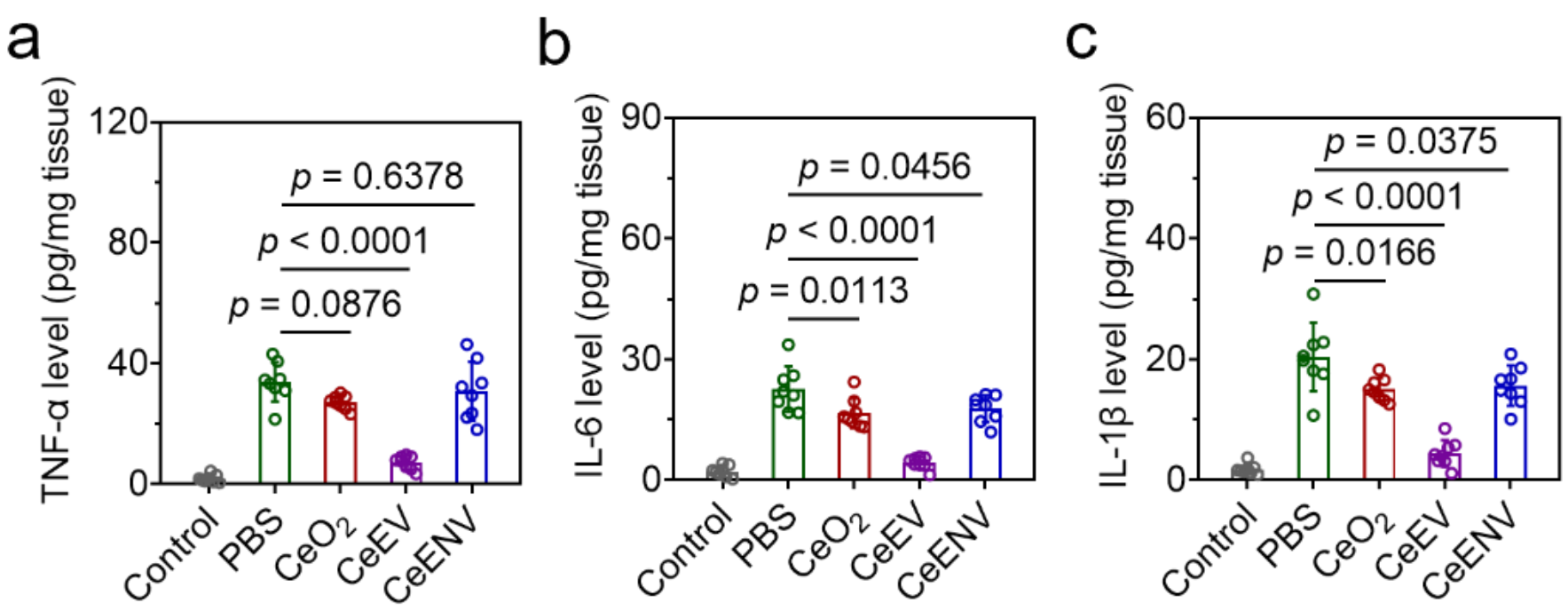

Supplementary Fig. 32. Levels of secreted tumor necrosis factor-α (TNF-α) (a), interleukin-6 (IL-6) (b), and interleukin-1β (IL-1β) (c) in lung tissue homogenates collected from mice with acute lung injury treated with PBS, CeO_2, CeEV, or CeENV (3 mg/kg). Data are presented as the mean ± S.D. (n = 8 male BALB/C mice per group, 6-8 weeks of age). Normal mice without lipopolysaccharide (LPS) challenge or nanoparticle treatment served as the control. Statistical analysis was calculated *via* one-way ANOVA with Dunnett's multiple comparisons test. Source data are provided as a Source Data file.

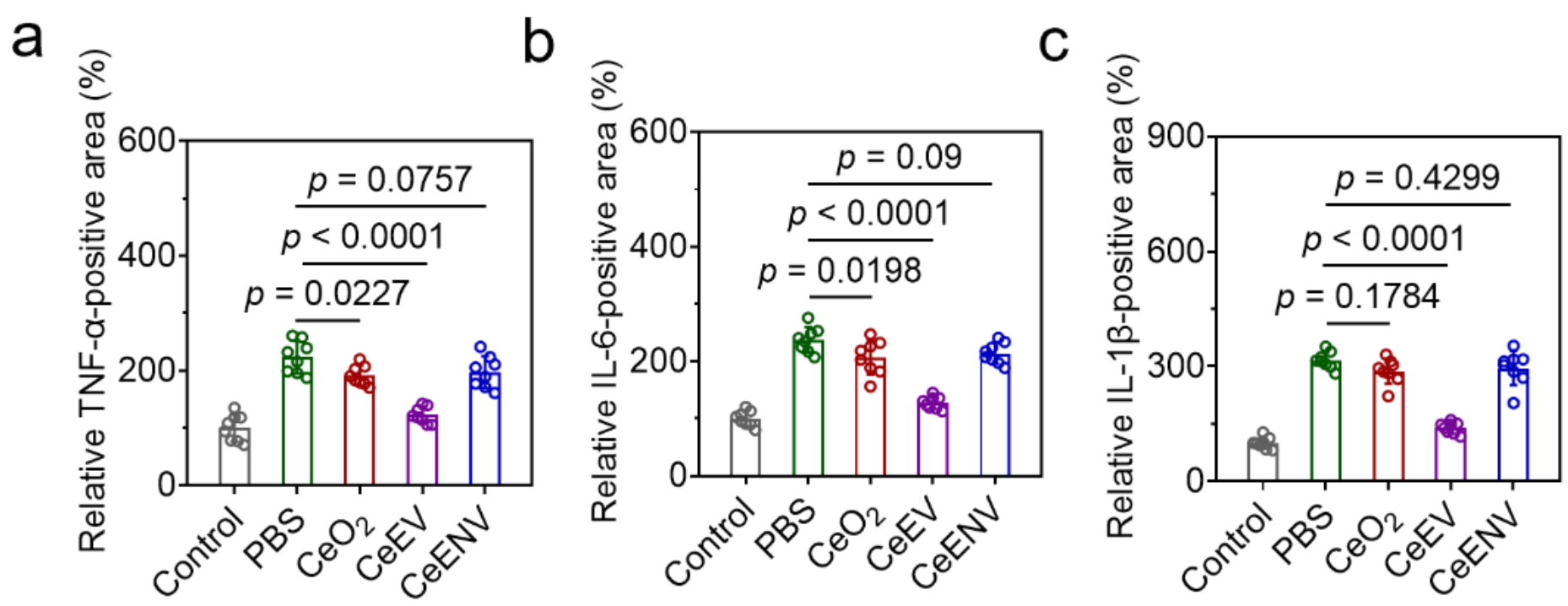

Supplementary Fig. 33. Semi-quantitative analysis of inflammatory factors from immunohistochemistry images of lung sections from mice with acute lung injury treated with PBS, CeO_2, CeEV, or CeENV (3 mg/kg), including tumor necrosis factor-α (TNF-α) (a), interleukin-6 (IL-6) (b), and interleukin-1β (IL-1β) (c). Data are presented as the mean ± S.D. (n = 8 male BALB/C mice per group, 6-8 weeks of age). Normal mice without lipopolysaccharide (LPS) challenge or nanoparticle treatment served as the control. Statistical analysis was calculated *via* one-way ANOVA with Dunnett's multiple comparisons test. Source data are provided as a Source Data file.

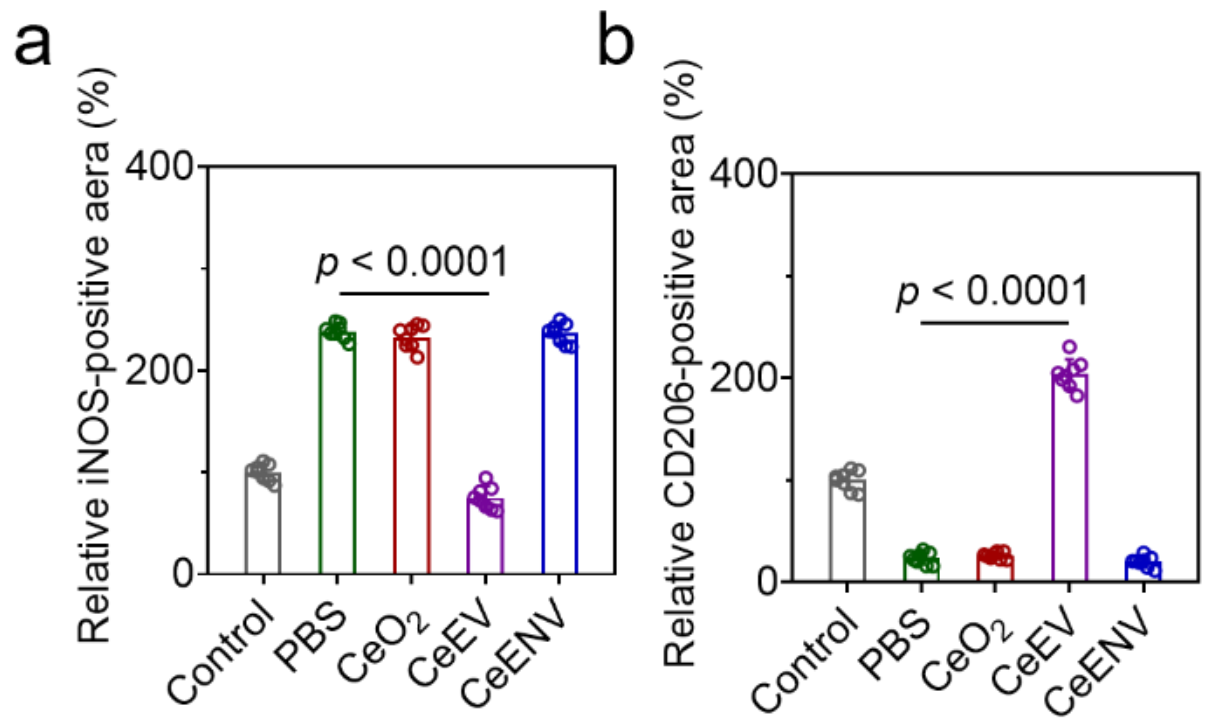

Supplementary Fig. 34. Semi-quantitative analysis of levels of iNOS (a) and CD206 (b) from confocal laser scanning microscopy images of lung sections from mice with acute lung injury treated with PBS, CeO_2, CeEV, or CeENV (3 mg/kg). Data are presented as the mean ± S.D. (n = 8 male BALB/C mice per group, 6-8 weeks of age). Normal mice without lipopolysaccharide (LPS) challenge or nanoparticle treatment served as the control. Statistical analysis was calculated with a two-tailed unpaired t-test. Source data are provided as a Source Data file.

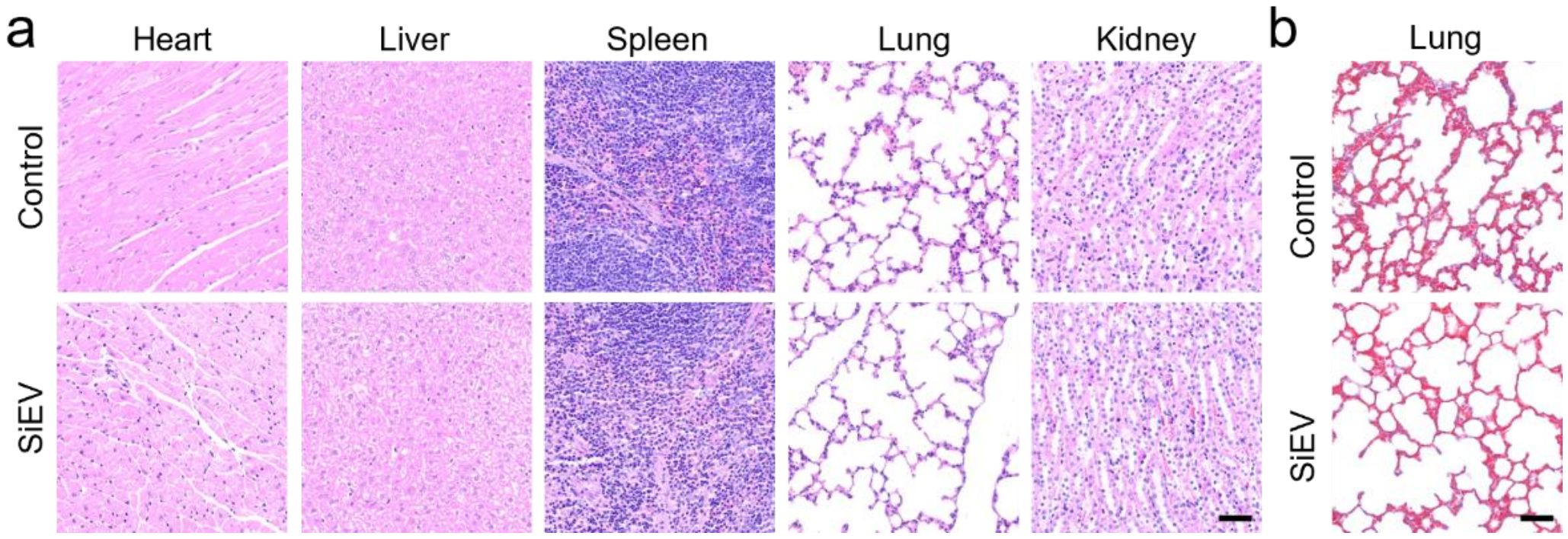

Supplementary Fig. 35. Safety assessment of SiEV *in vivo*. (a) Representative hematoxylin-eosin images of major organs on 7 d post intravenous (*i.v.*) injection of PBS and SiEV. (b) Representative Masson tricolor images of lung tissues on 7 d post *i.v.* injection of PBS and SiEV. Scale bar = 50 μm.

Supplementary Tables

Supplementary Table 1. Characterization of various heteropolypeptide-decorated SiO_2 NPs.[a]

Entry	NPs[b]	Composition[c]	Deprotection efficiency[c, e]	D_h (nm)[d]	ζ (mV)[d]
1	SiE	-	> 95%	36.2	-25.2
2	SiEK	89:11	> 99%	38.8	-15.6
3	SiER	87:13	> 99%, > 94%[f]	35.4	-18.1
4	SiEQ	88:12	> 99%, > 99%[h]	38.0	-20.1
5	SiES	91:9	> 97%	39.8	-20.4
6	SiET	89:11	> 99%	39.8	-20.9
7	SiEY	89:11	> 94%	38.5	-22.3
8	SiEA	87:13	> 95%	39.1	-22.9
9	SiEL	90:10	> 99%	38.7	-25.6
10	SiEI	89:11	> 99%	39.3	-23.4
11	SiEV	89:11	> 99%	37.8	-24.9
12	SiEF	91:9	> 94%	37.7	-24.6
13	SiENV	89:11	> 99%	38.0	-22.6

[a]Heteropolypeptide-decorated SiO_2 NPs were prepared by surface-initiated ROP of NCA monomers followed by side-chain deprotection. [b]E = glutamic acid, K = lysine, R = arginine, Q = glutamine, S = serine, T = theronine, Y = tyrosine, A = alanine, L = leucine, I = isoleucine, V = valine, F = phenylalanine, NV = norvaline. [c]Determined by NMR. [d]Determined by zetasizer. [e]The deprotection efficiency of *t*Bu group from protected glutamic acid residues unless otherwise specified. [f]The deprotection efficiency of Pbf group from protected arginine residues. [h]The deprotection efficiency of Trt group from protected glutamine residues.

Supplementary Table 2. Characterization of various SiEV NPs with different sizes.[a]

Entry	$D_{h,\ \mathrm{silica}}$ (nm)[b]	D_h (nm)[c]	ζ (mV)[c]
1	10	33	-22.8
2	20	48	-25.5
3	30	52	-22.1
4	50	70	-24.9
5	80	96	-24.9

[a]SiEV NPs were prepared by surface-initiated ROP of NCA monomers followed by side-chain deprotection. [b]Designed sizes of silica NPs without heteropolypeptide grafting. [c]Determined by zetasizer.

Supplementary Table 3. Characterization of various SiEV NPs with different valine percentages.[a]

Entry	Composition[b]	Deprotection efficiency[b]
1	99:1	> 99%
2	96:4	> 99%
3	85:15	> 99%
4	81:19	> 99%

[a]SiEV NPs were prepared by surface-initiated ROP of NCA monomers followed by side-chain deprotection. [b]Determined by NMR.

Supplementary NMR Spectra

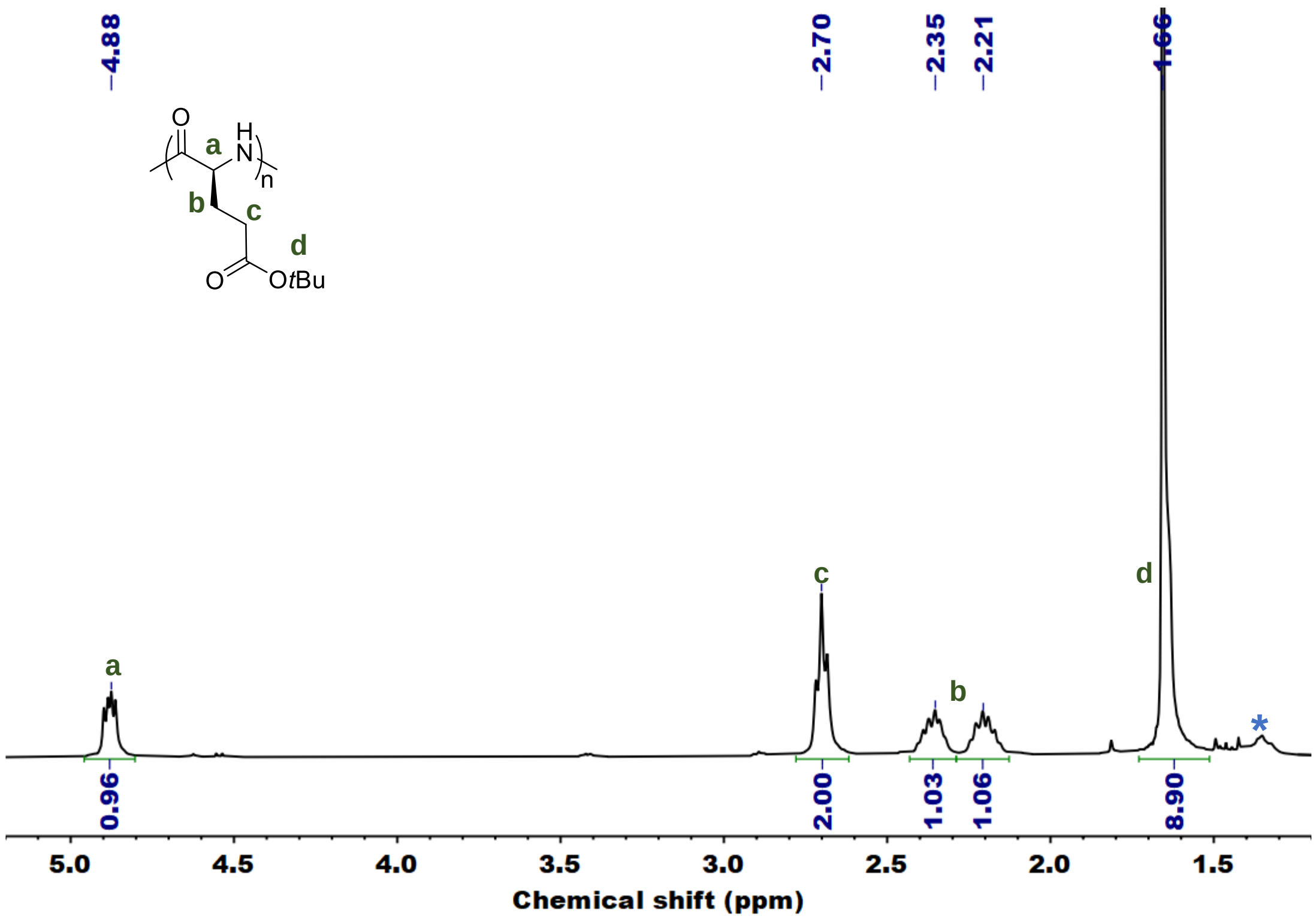

Supplementary Fig. 36. ^{1}H NMR spectrum (400 MHz) of SiE^{tBu} in TFA-*d* after HF etching of silica cores.

The blue star refers to the protons from residual silanes after HF etching for all NMR analysis.

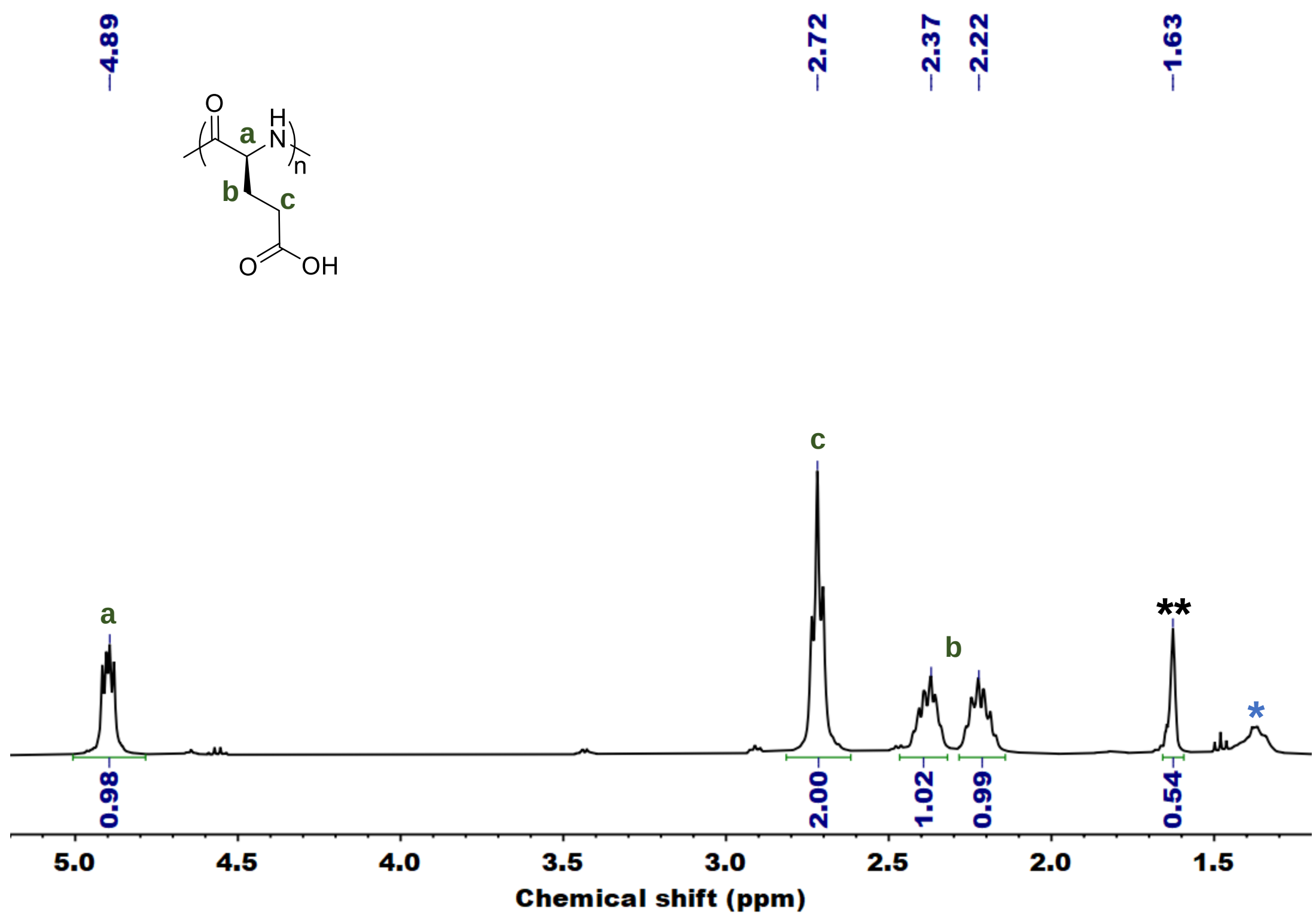

Supplementary Fig. 37. ^{1}H NMR spectrum (400 MHz) of SiE in TFA-*d* after side-chain deprotection and HF etching of silica cores.

The deprotection efficiency was determined by comparing the integral of peaks at 1.7-1.5 ppm (i.e., residual *tert*-butyl protons, highlighted with **) to characteristic peaks at 2.72 ppm (i.e., γ-methylene protons of glutamic acid residues). The deprotection efficiency of all heteropolypeptides was calculated in a similar way (residual *tert*-butyl protons highlighted with **) unless otherwise specified.

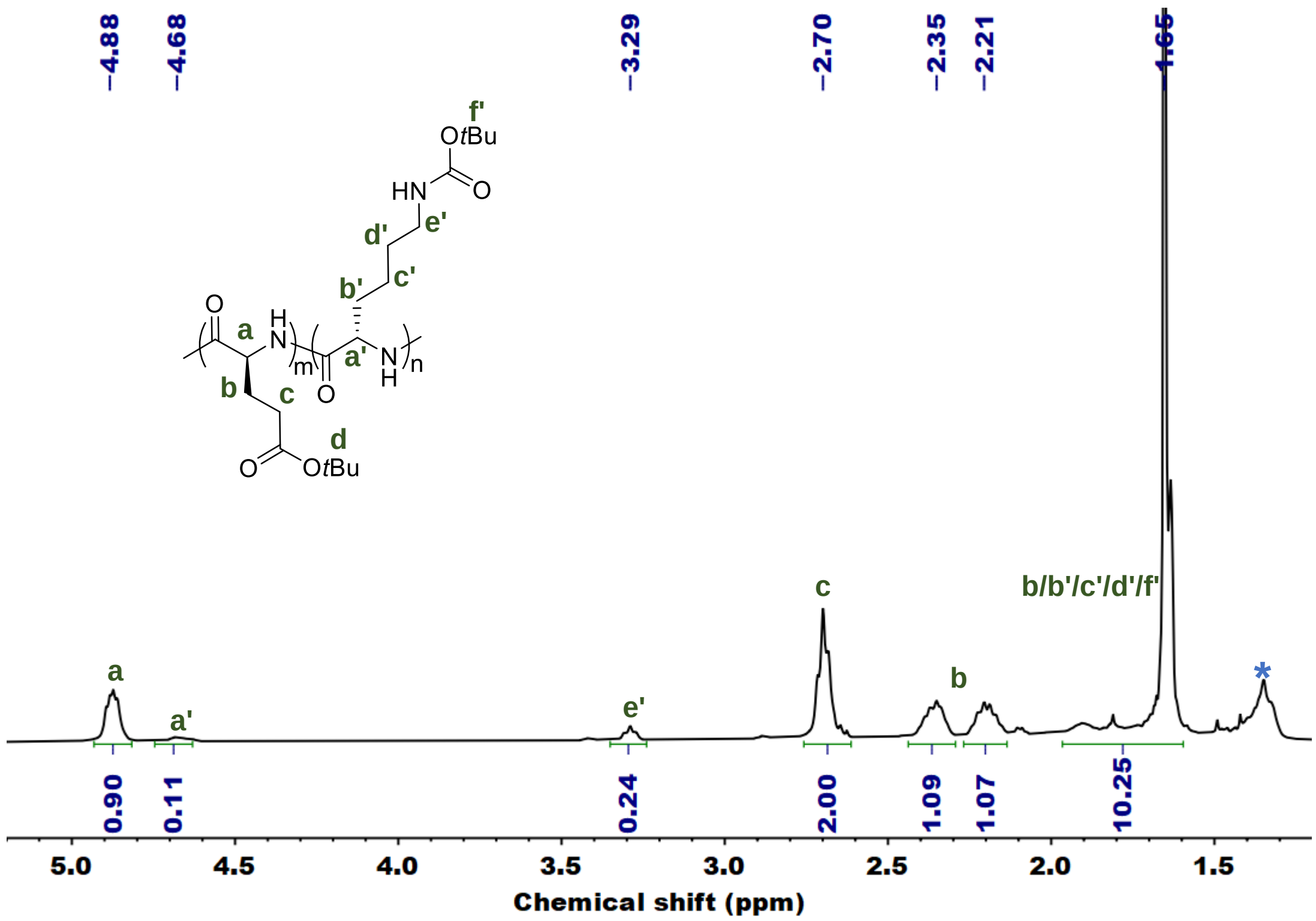

Supplementary Fig. 38. ^{1}H NMR spectrum (400 MHz) of SiE^{tBu}K^{Boc} in TFA-*d* after HF etching of silica cores.

The composition of SiE^{tBu}K^{Boc} was determined by comparing the integral of peaks at 2.70 ppm (i.e., γ-methylene protons of glutamic acid residues) to peaks at 3.29 ppm (i.e., ε-methylene protons of lysine residues).

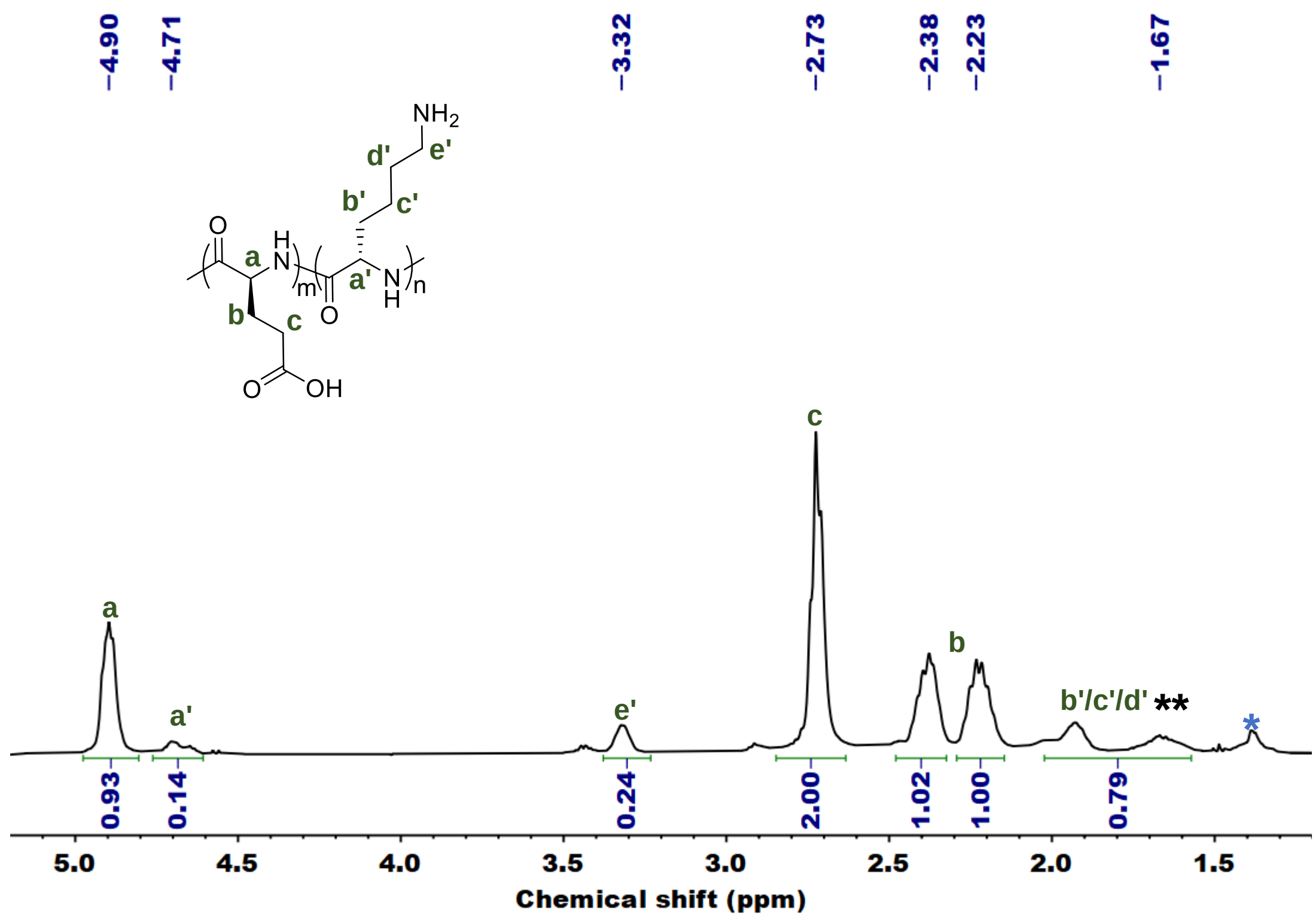

Supplementary Fig. 39. ^{1}H NMR spectrum (400 MHz) of SiEK in TFA-*d* after side-chain deprotection and HF etching of silica cores.

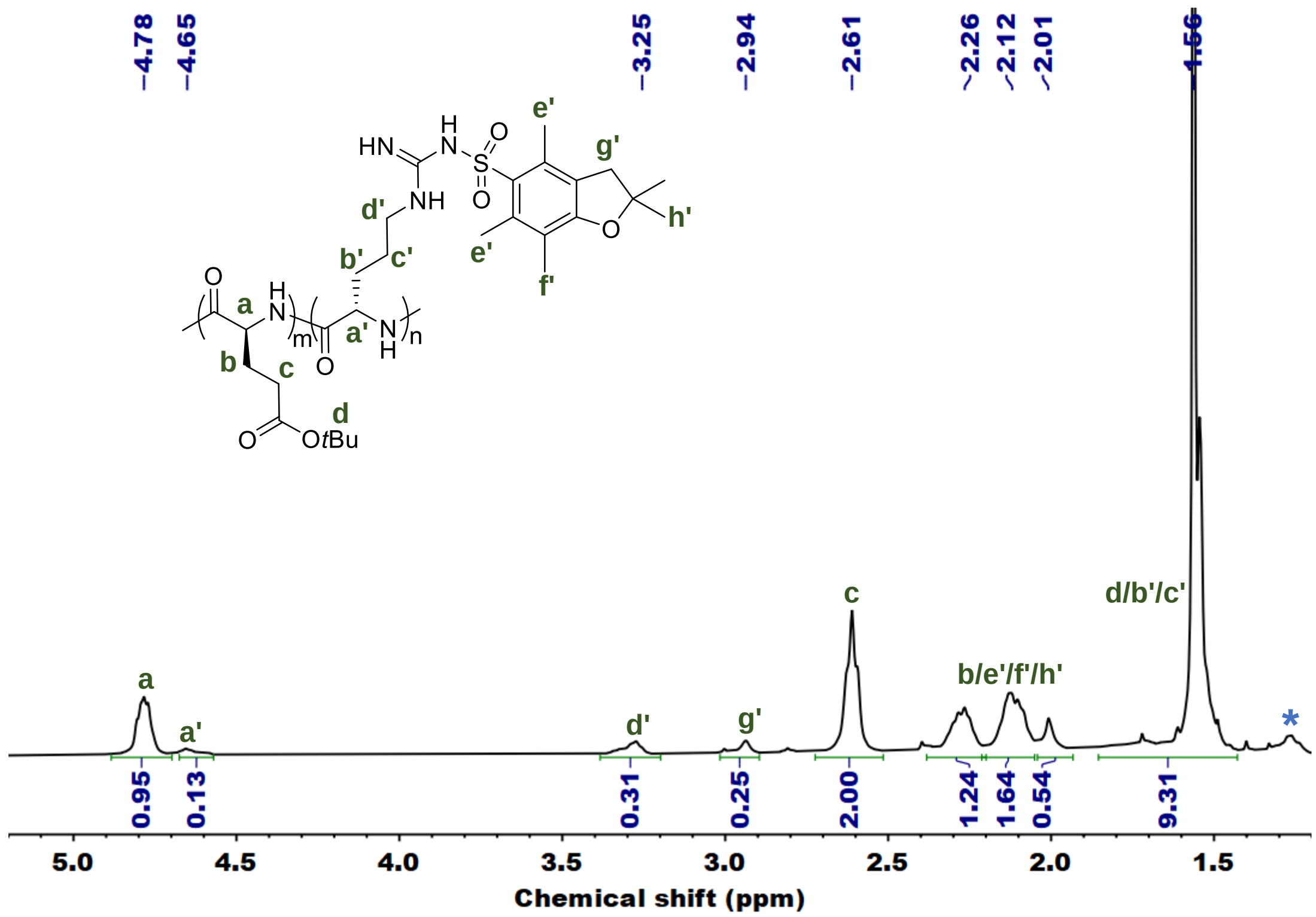

Supplementary Fig. 40. ^{1}H NMR spectrum (400 MHz) of SiE^{tBu}R^{Pbf} in TFA-*d* after HF etching of silica cores.

The composition of SiE^{tBu}R^{Pbf} was determined by comparing the integral of peaks at 2.61 ppm (i.e., γ-methylene protons of glutamic acid residues) to peaks at 3.25 ppm (i.e., δ-methylene protons of arginine residues).

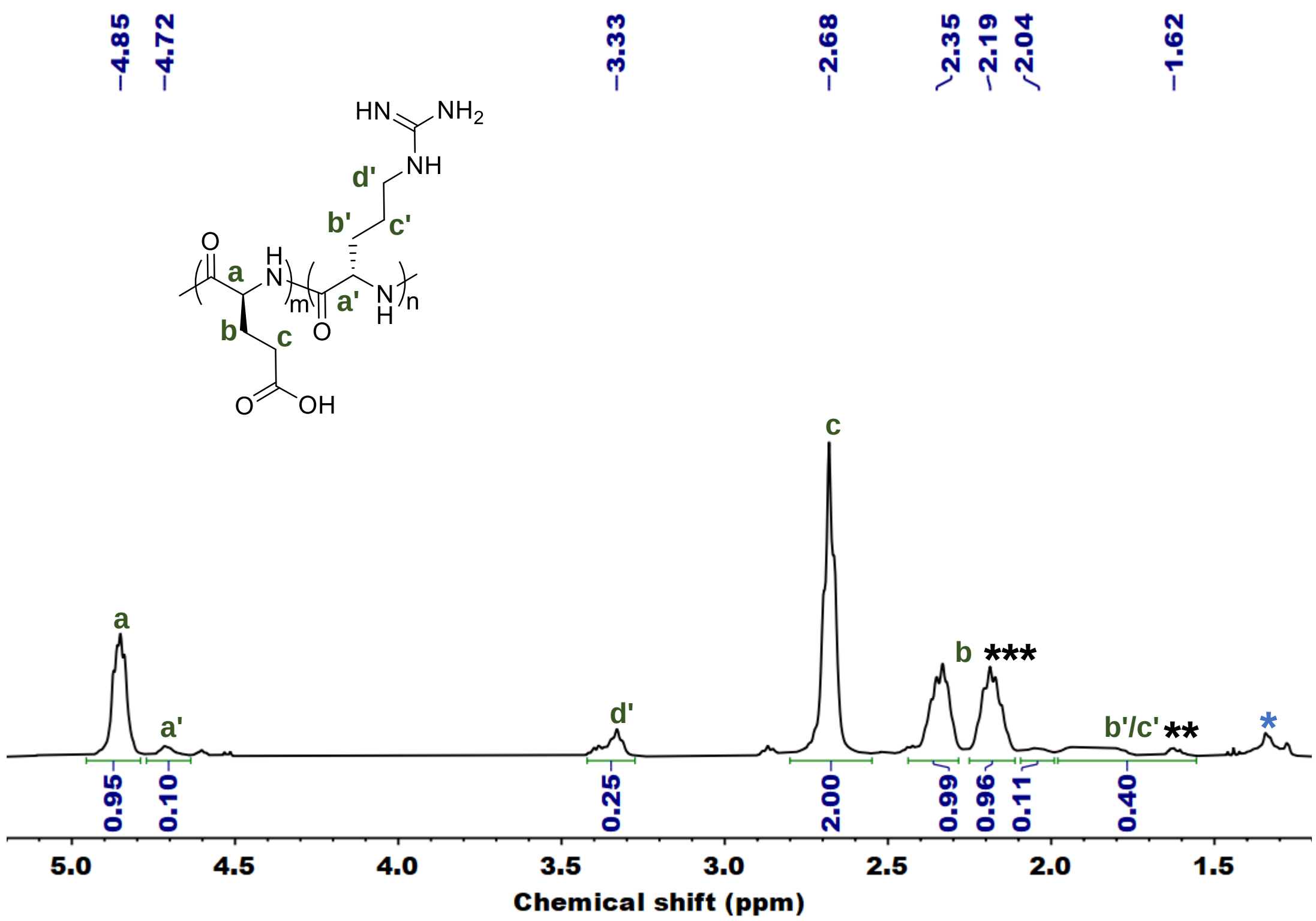

Supplementary Fig. 41. ^{1}H NMR spectrum (400 MHz) of SiER in TFA-*d* after side-chain deprotection and HF etching of silica cores.

The deprotection efficiency of guanidine residues was determined by comparing the integral ratio of peaks of 2.5-2.0 ppm (i.e., residual 2,2,4,6,7-pentamethyldihydrobenzofuran-5-sulfonyl protons, highlighted with ***) to characteristic peaks at 3.33 ppm (i.e., δ-methylene protons of arginine residues).

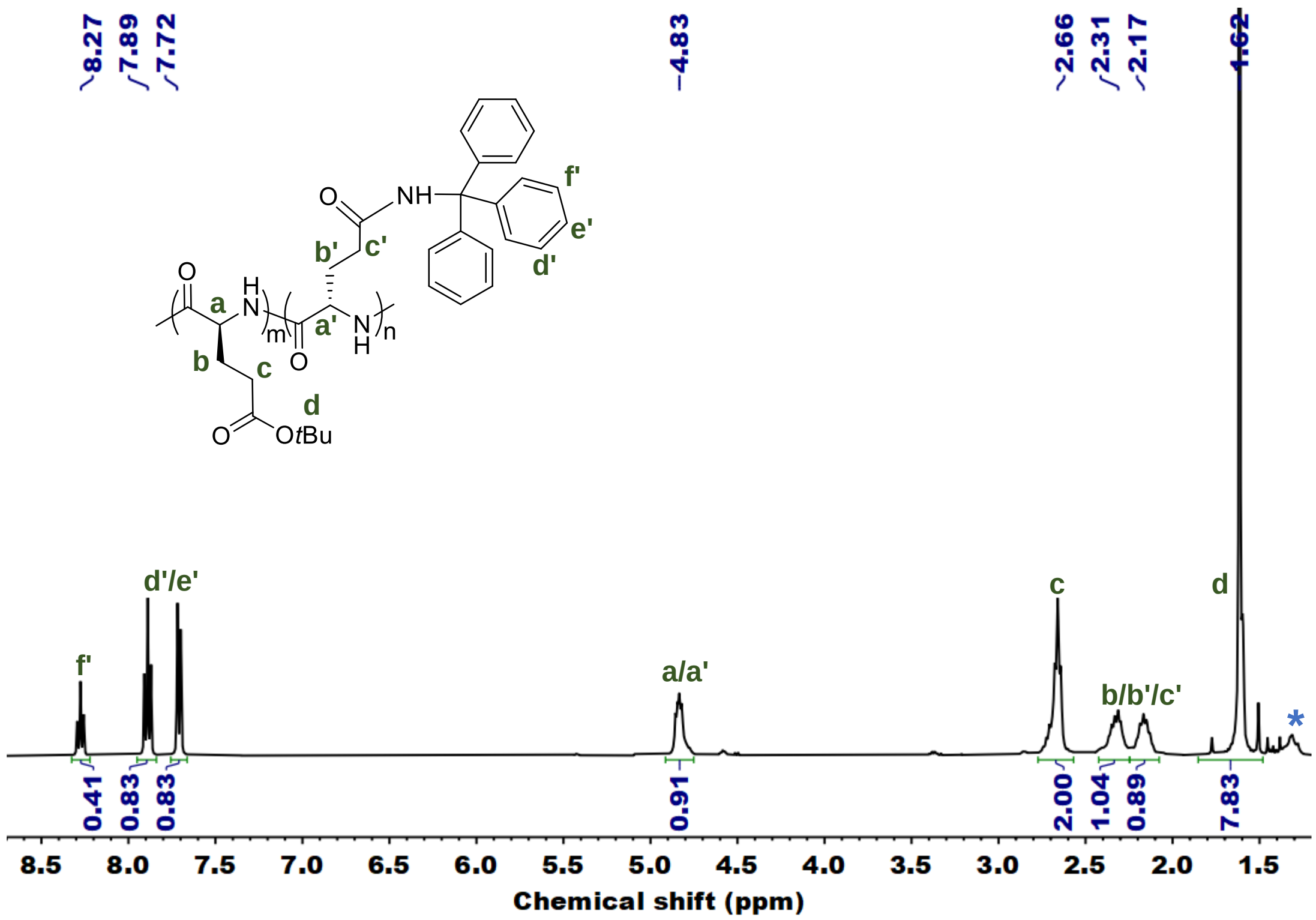

Supplementary Fig. 42. ^{1}H NMR spectrum (400 MHz) of SiE^{tBu}Q^{Trt} in TFA-*d* after HF etching of silica cores.

The composition of SiE^{tBu}Q^{Trt} was determined by comparing the integral of peaks at 2.66 ppm (i.e., γ-methylene protons of glutamic acid residues) to peaks at 8.4-7.6 ppm (i.e., trityl protons of glutamine residues).

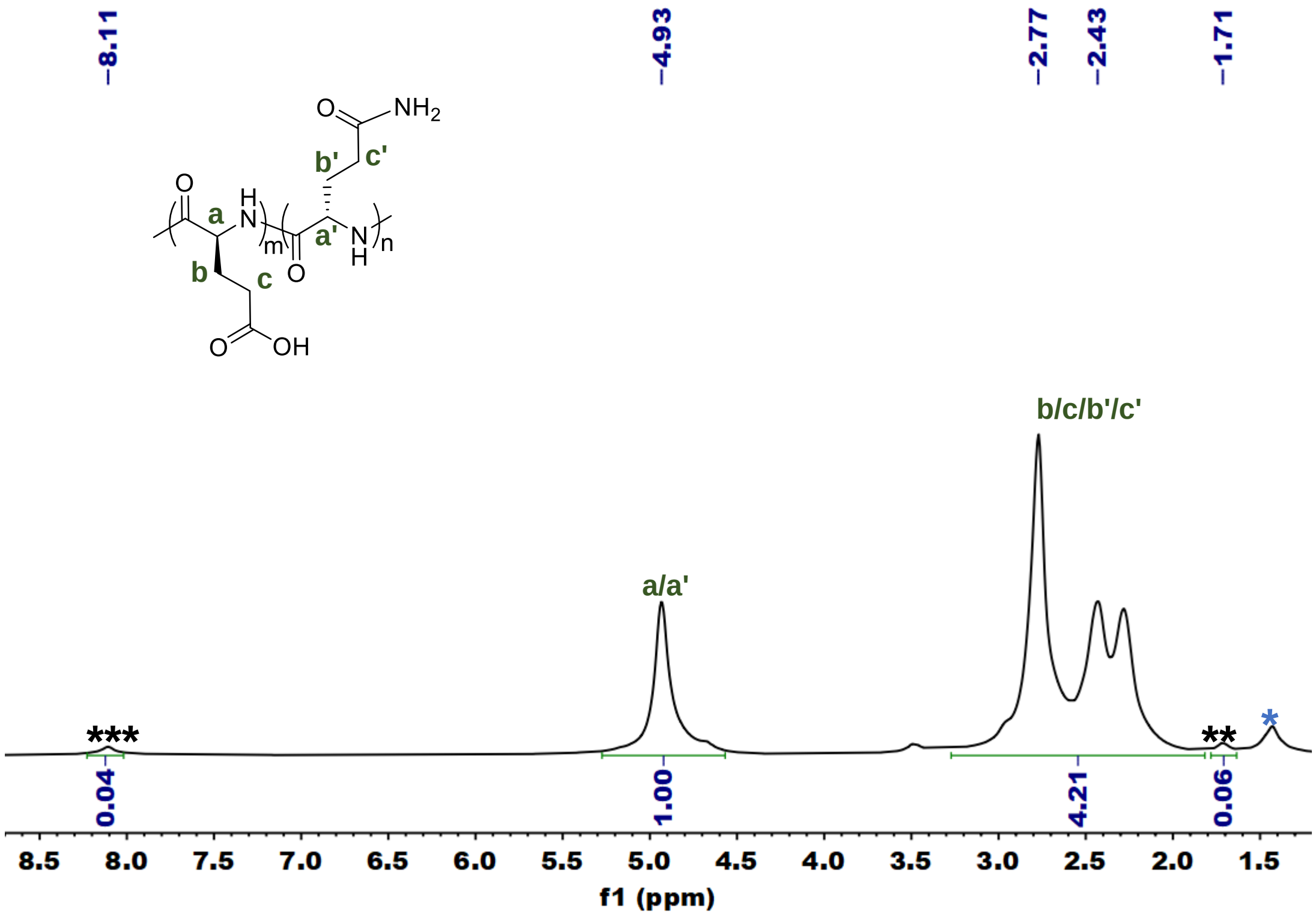

Supplementary Fig. 43. ^{1}H NMR spectrum (400 MHz) of SiEQ in TFA-*d* after side-chain deprotection and HF etching of silica cores.

The deprotection efficiency of glutamine residues was determined by comparing the integral ratio of peaks of 8.3-8.0 ppm (i.e., residual trityl protons, highlighted with ***) to characteristic peaks at 3.3-1.8 ppm (i.e., γ-methylene protons of glutamine residues).

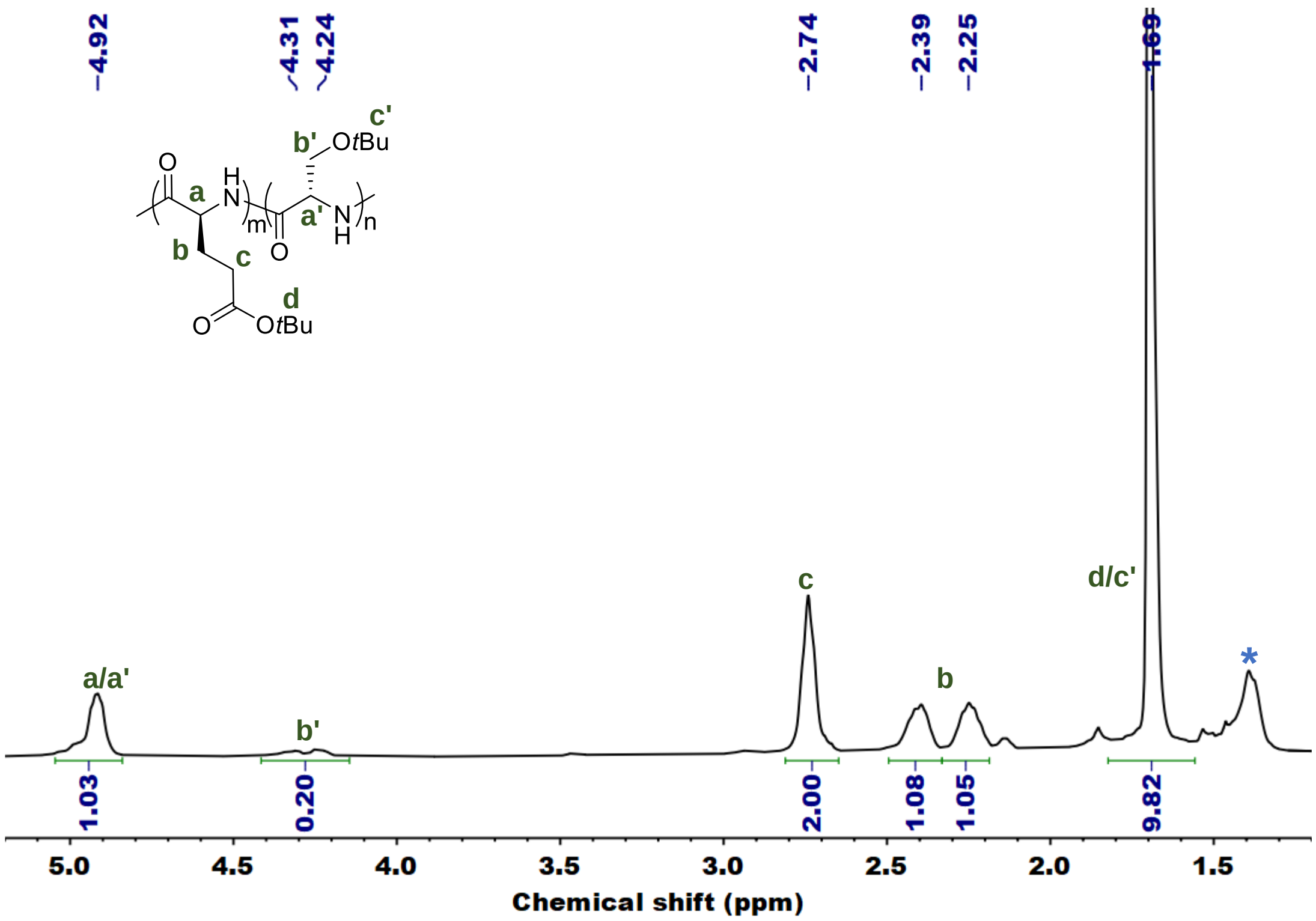

Supplementary Fig. 44. ^{1}H NMR spectrum (400 MHz) of SiE^{tBu}S^{tBu} in TFA-*d* after HF etching of silica cores.

The composition of SiE^{tBu}S^{tBu} was determined by comparing the integral of peaks at 2.74 ppm (i.e., γ-methylene protons of glutamic acid residues) to peaks at 4.4-4.1 ppm (i.e., methylene protons of serine residues).

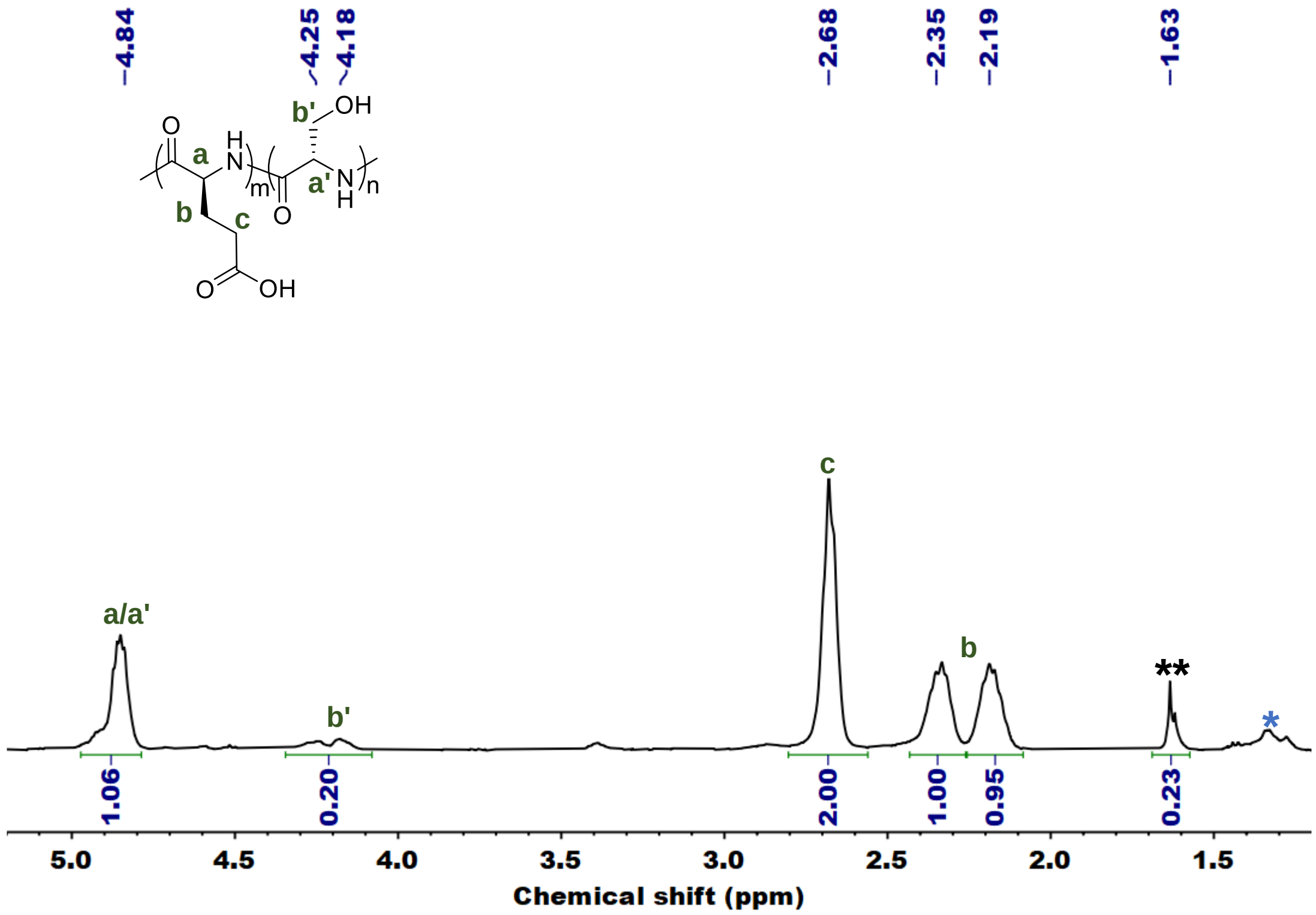

Supplementary Fig. 45. ^{1}H NMR spectrum (400 MHz) of SiES in TFA-*d* after side-chain deprotection and HF etching of silica cores.

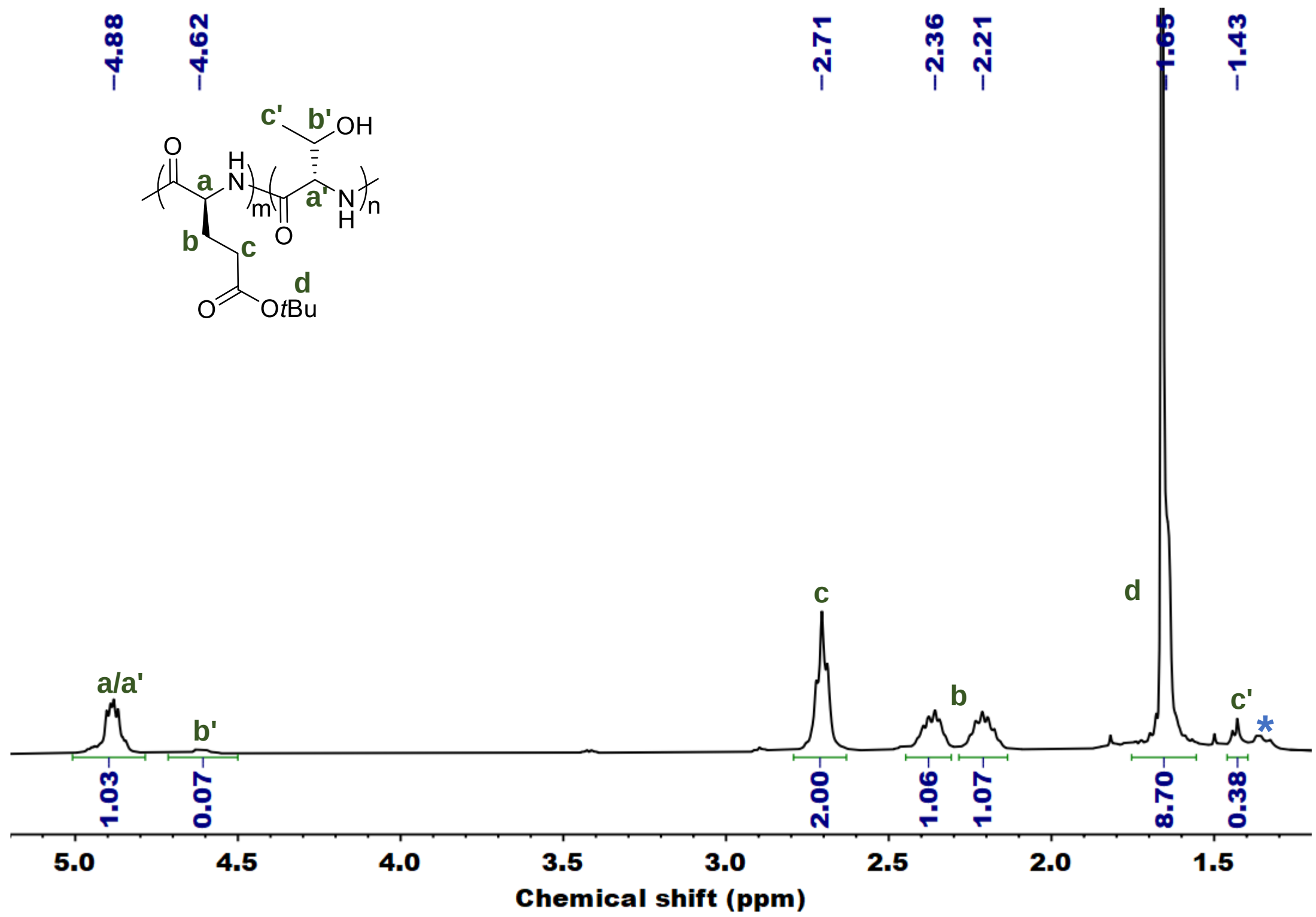

Supplementary Fig. 46. ^{1}H NMR spectrum (400 MHz) of SiE^{tBu}T in TFA-*d* after HF etching of silica cores.

The composition of SiE^{tBu}T was determined by comparing the integral of peaks at 2.71 ppm (i.e., γ-methylene protons of glutamic acid residues) to peaks at 4.62 ppm (i.e., methine protons of threonine residues).

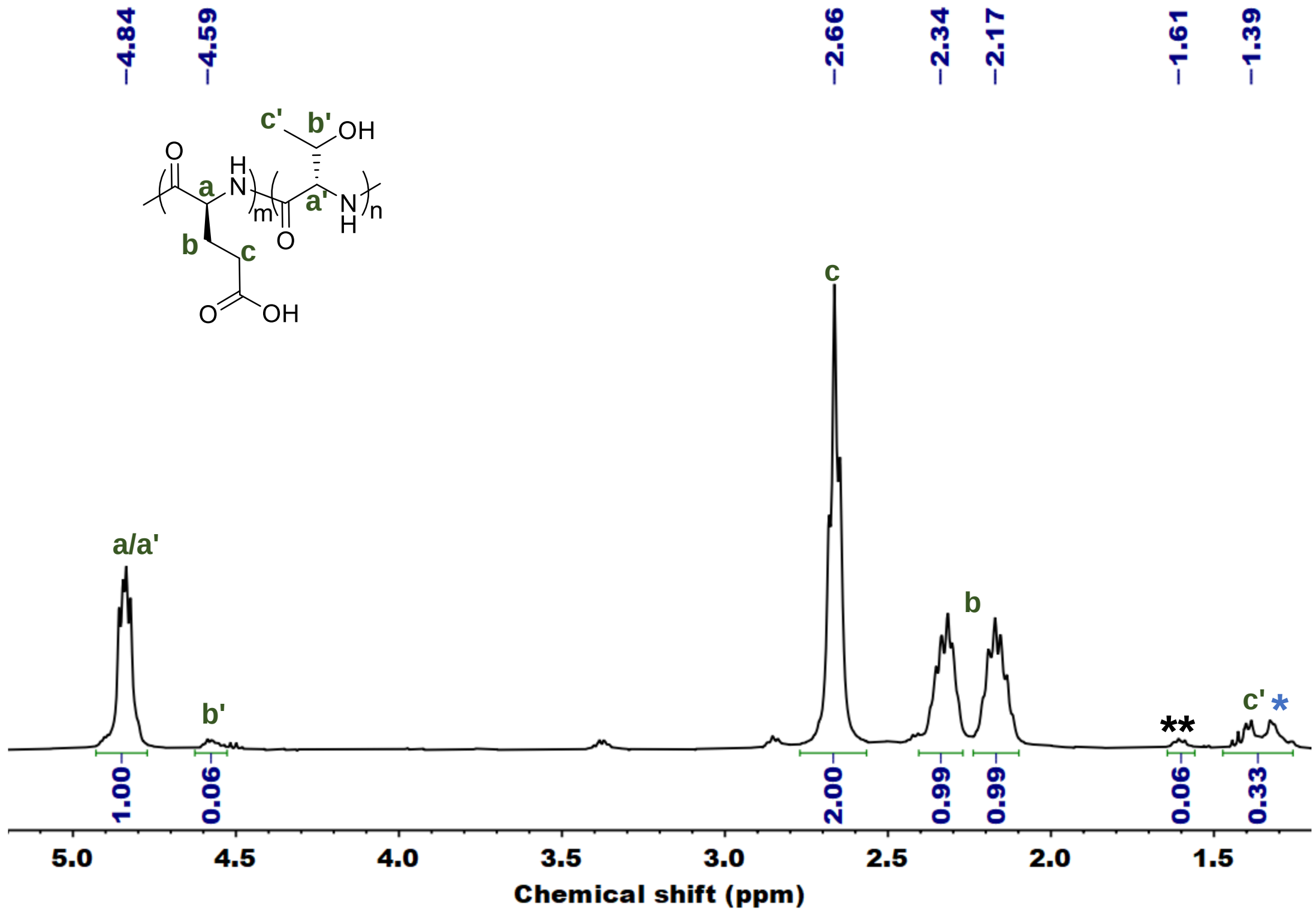

Supplementary Fig. 47. ^{1}H NMR spectrum (400 MHz) of SiET in TFA-*d* after side-chain deprotection and HF etching of silica cores.

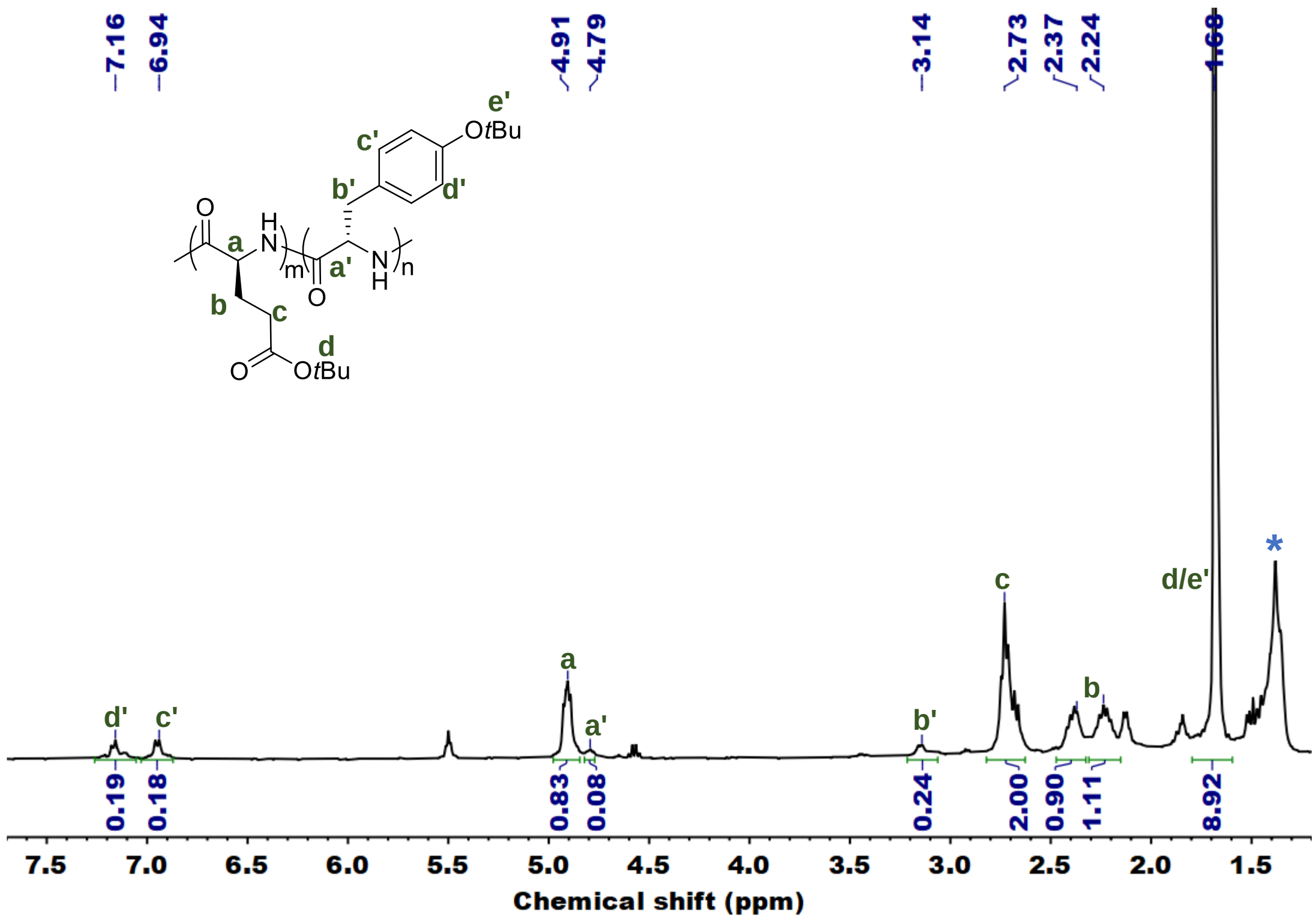

Supplementary Fig. 48. ^{1}H NMR spectrum (400 MHz) of SiE^{tBu}Y^{tBu} in TFA-*d* after HF etching of silica cores.

The composition of SiE^{tBu}Y^{tBu} was determined by comparing the integral of peaks at 2.73 ppm (i.e., γ-methylene protons of glutamic acid residues) to peaks at 7.16 ppm (i.e., aromatic protons of tyrosine residues).

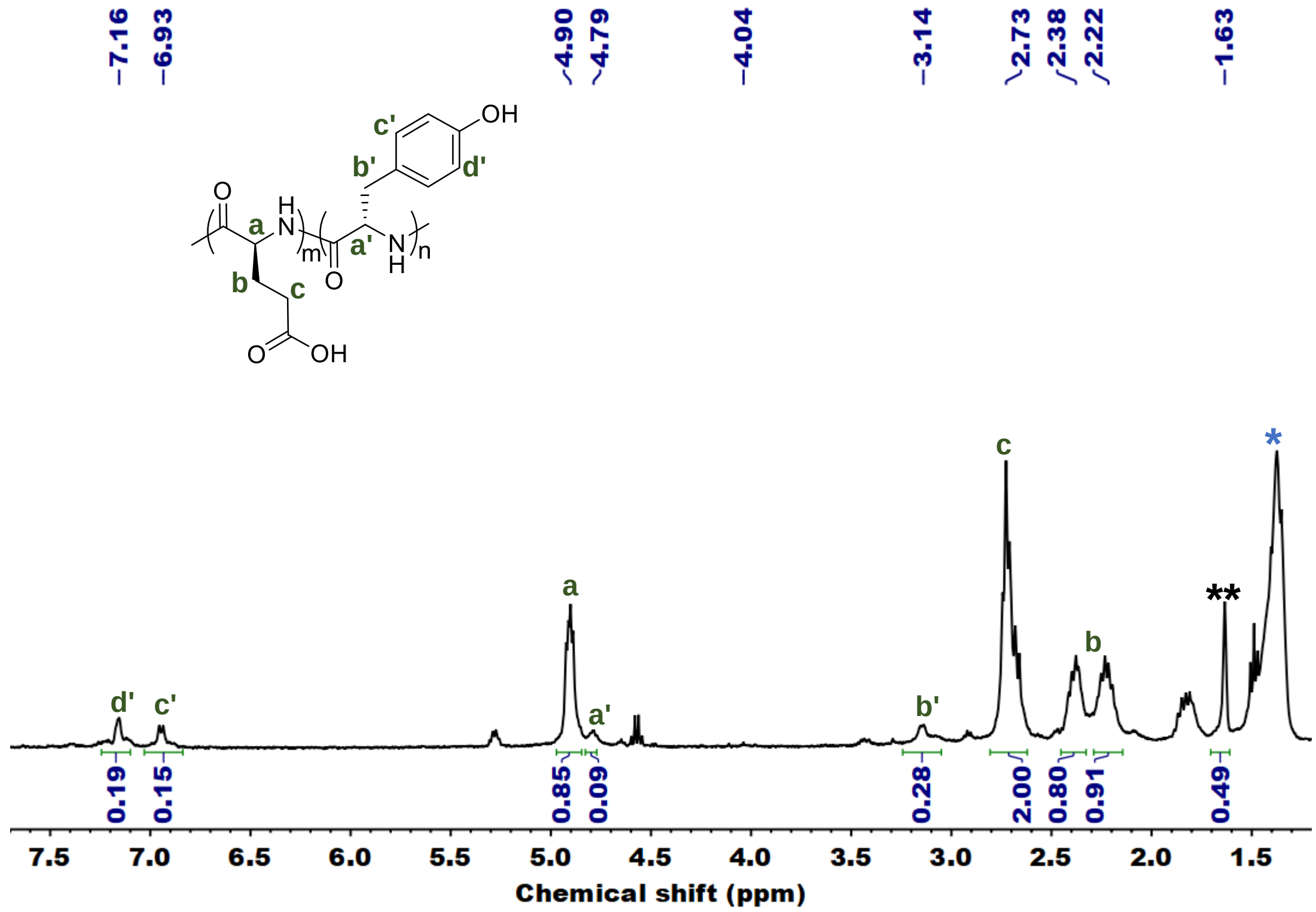

Supplementary Fig. 49. ^{1}H NMR spectrum (400 MHz) of SiEY in TFA-*d* after side-chain deprotection and HF etching of silica cores.

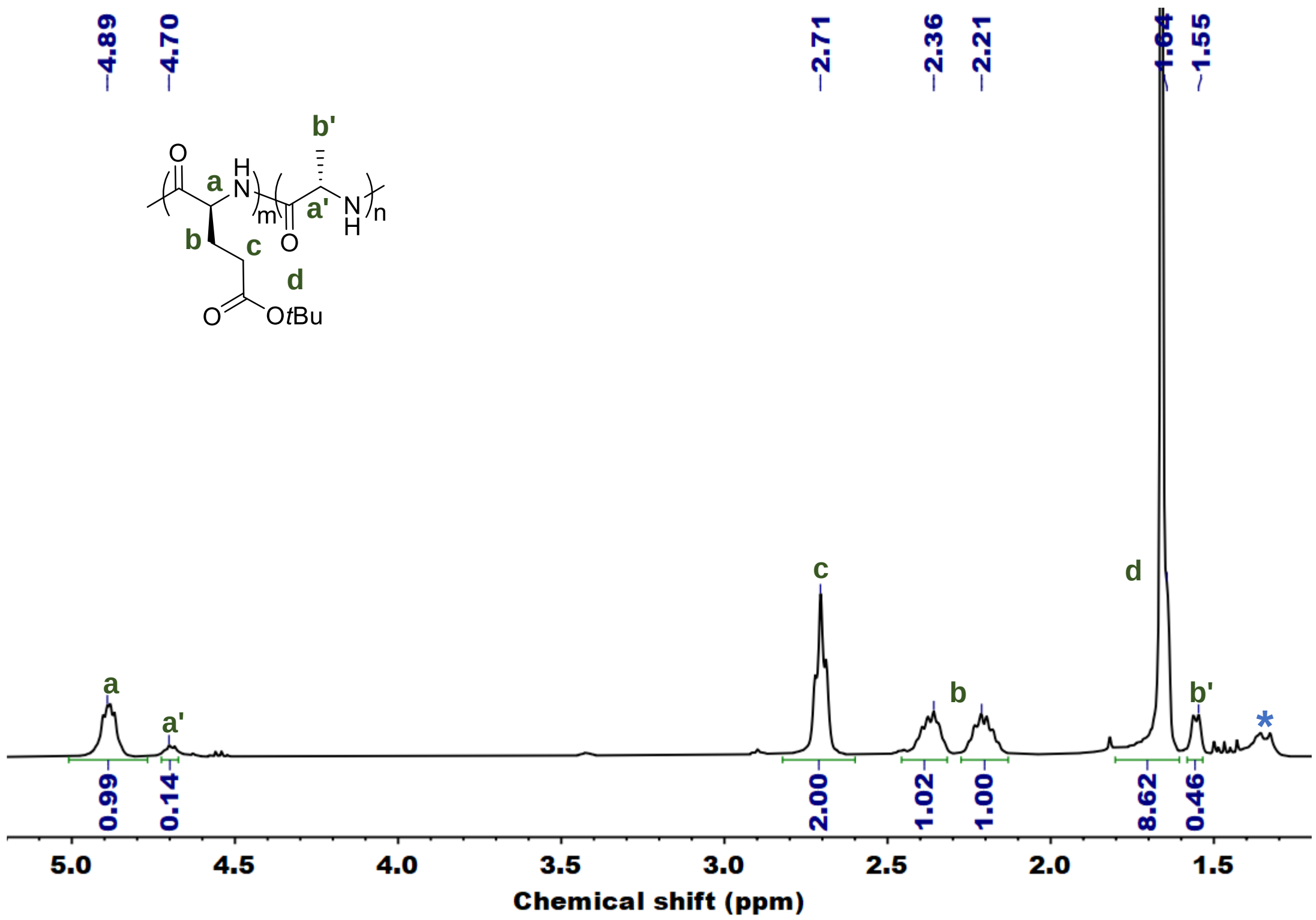

Supplementary Fig. 50. ^{1}H NMR spectrum (400 MHz) of SiE^{tBu}A in TFA-*d* after HF etching of silica cores.

The composition of SiE^{tBu}A was determined by comparing the integral of peaks at 2.71 ppm (i.e., γ-methylene protons of glutamic acid residues) to peaks at 1.55 ppm (i.e., methyl protons of alanine residues).

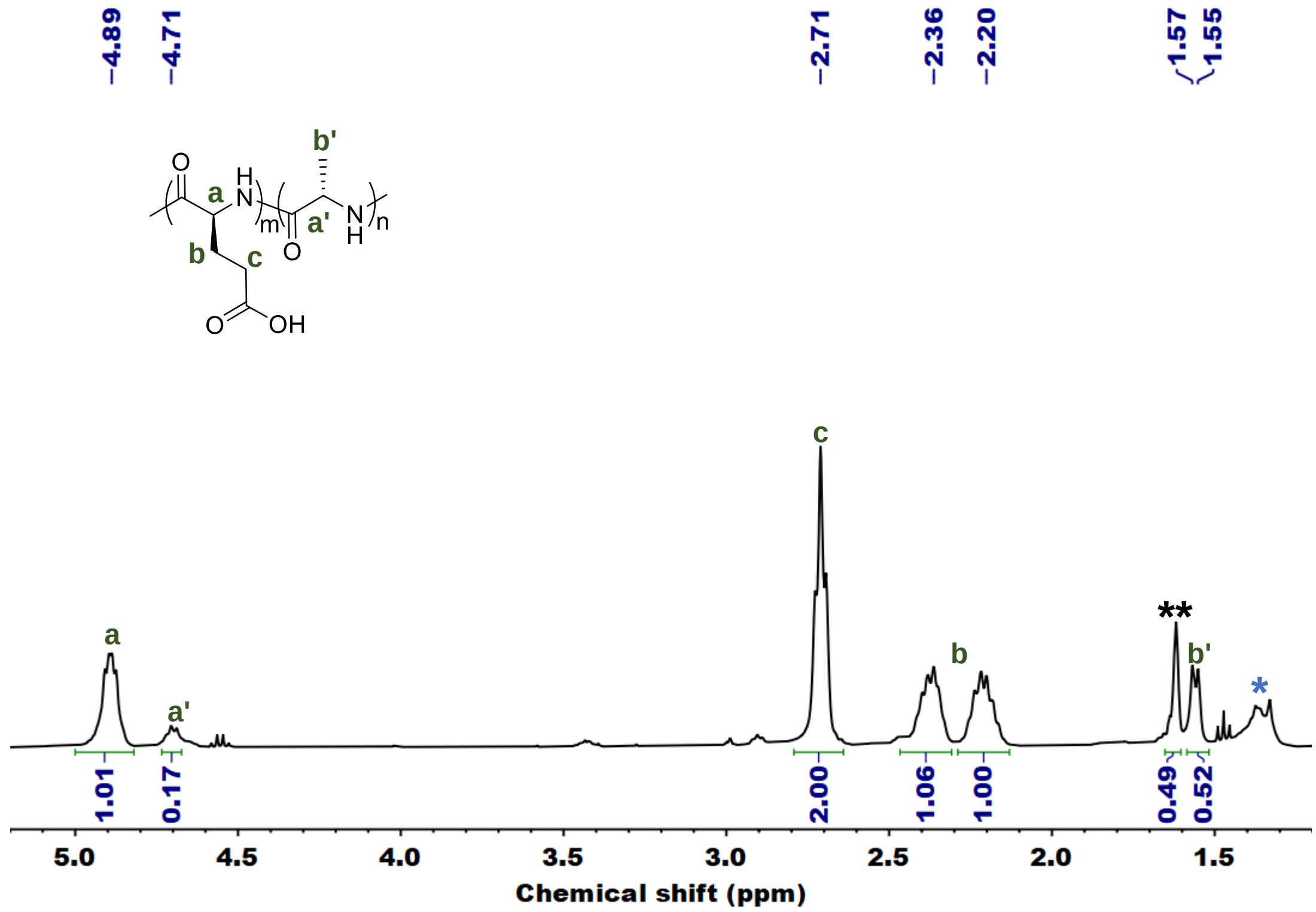

Supplementary Fig. 51. ^{1}H NMR spectrum (400 MHz) of SiEA in TFA-*d* after side-chain deprotection and HF etching of silica cores.

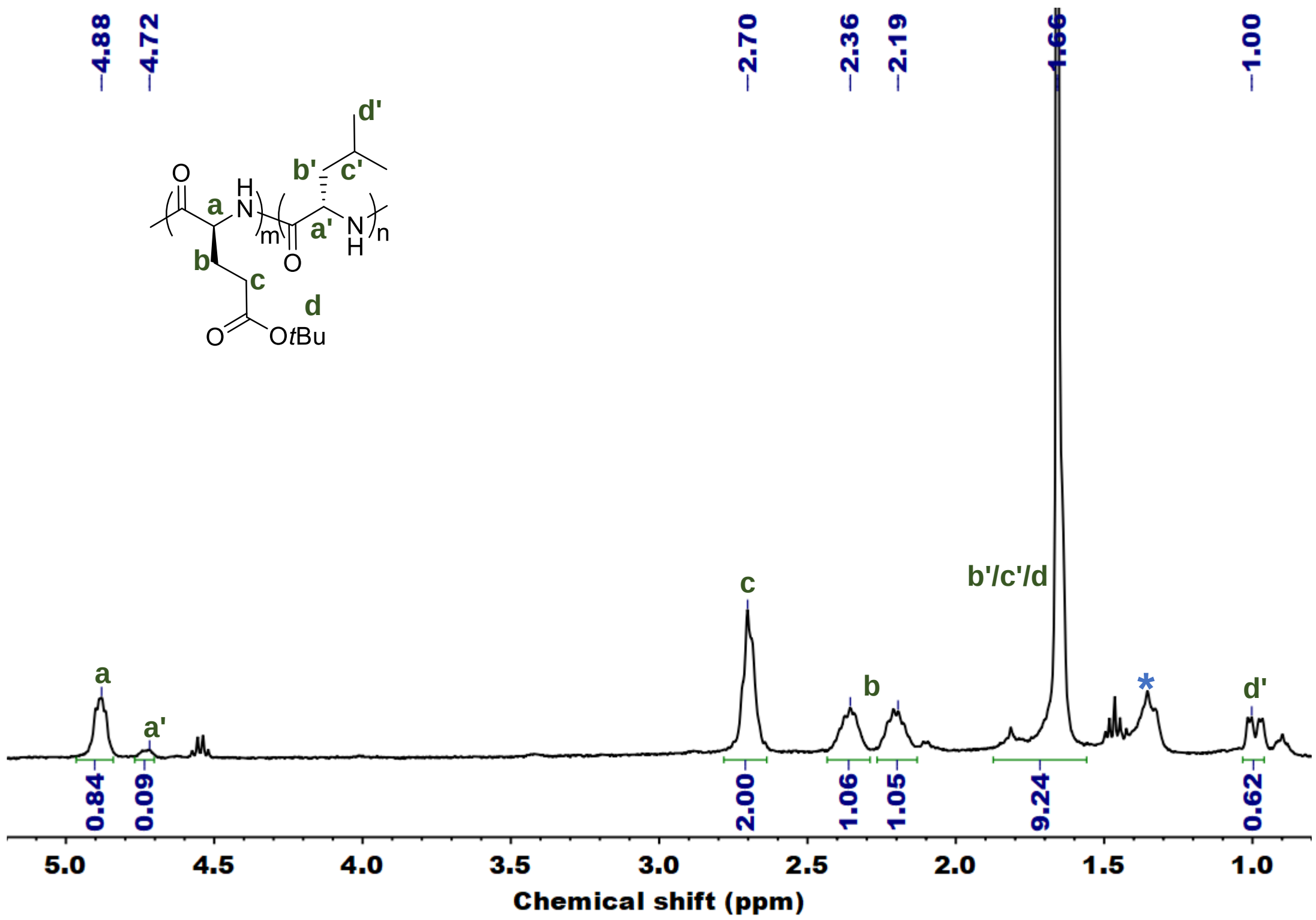

Supplementary Fig. 52. ^{1}H NMR spectrum (400 MHz) of SiE^{tBu}L in TFA-*d* after HF etching of silica cores.

The composition of SiE^{tBu}L was determined by comparing the integral of peaks at 2.70 ppm (i.e., γ-methylene protons of glutamic acid residues) to peaks at 1.0 ppm (i.e., methyl protons of leucine residues).

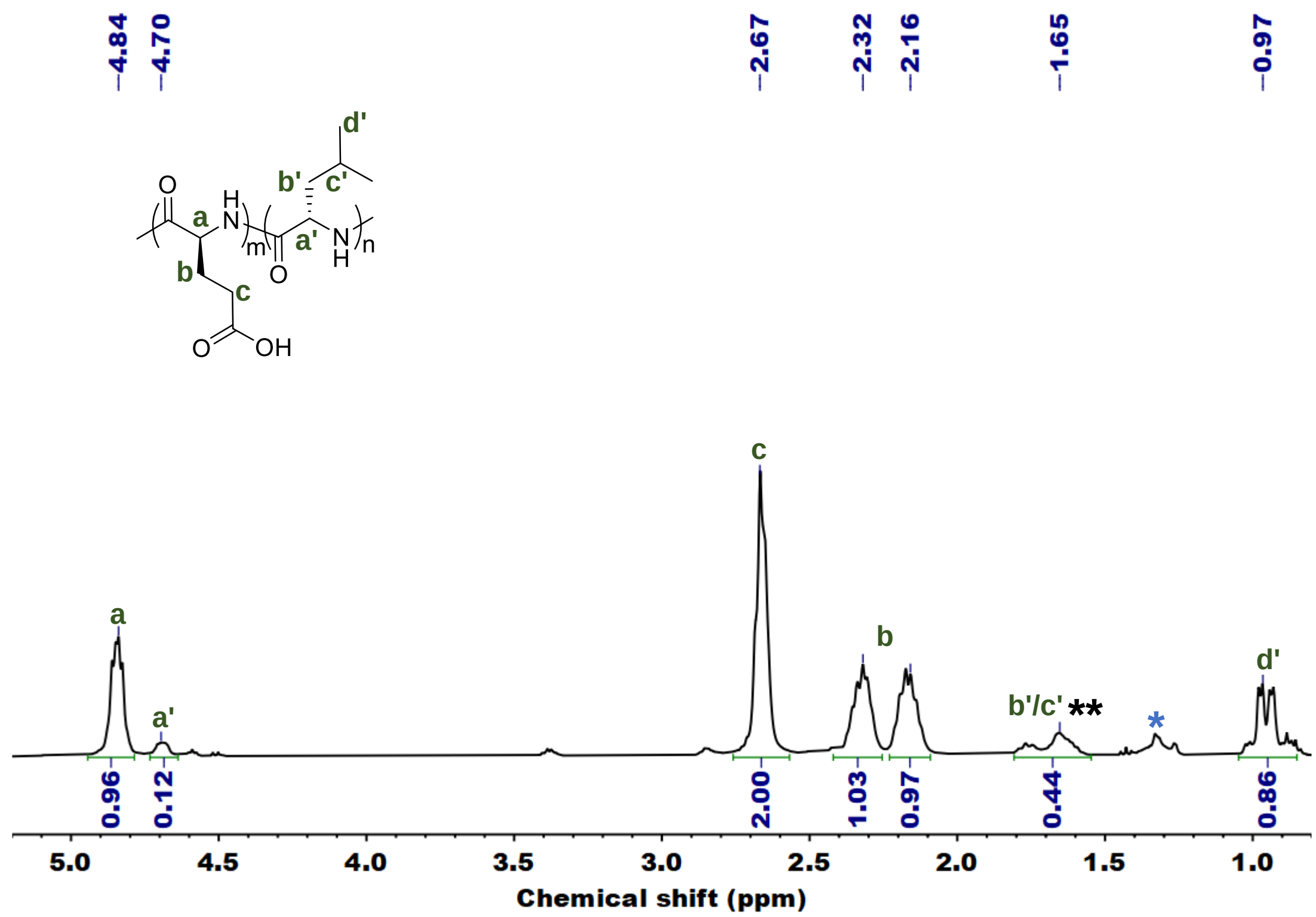

Supplementary Fig. 53. ^{1}H NMR spectrum (400 MHz) of SiEL in TFA-*d* after side-chain deprotection and HF etching of silica cores.

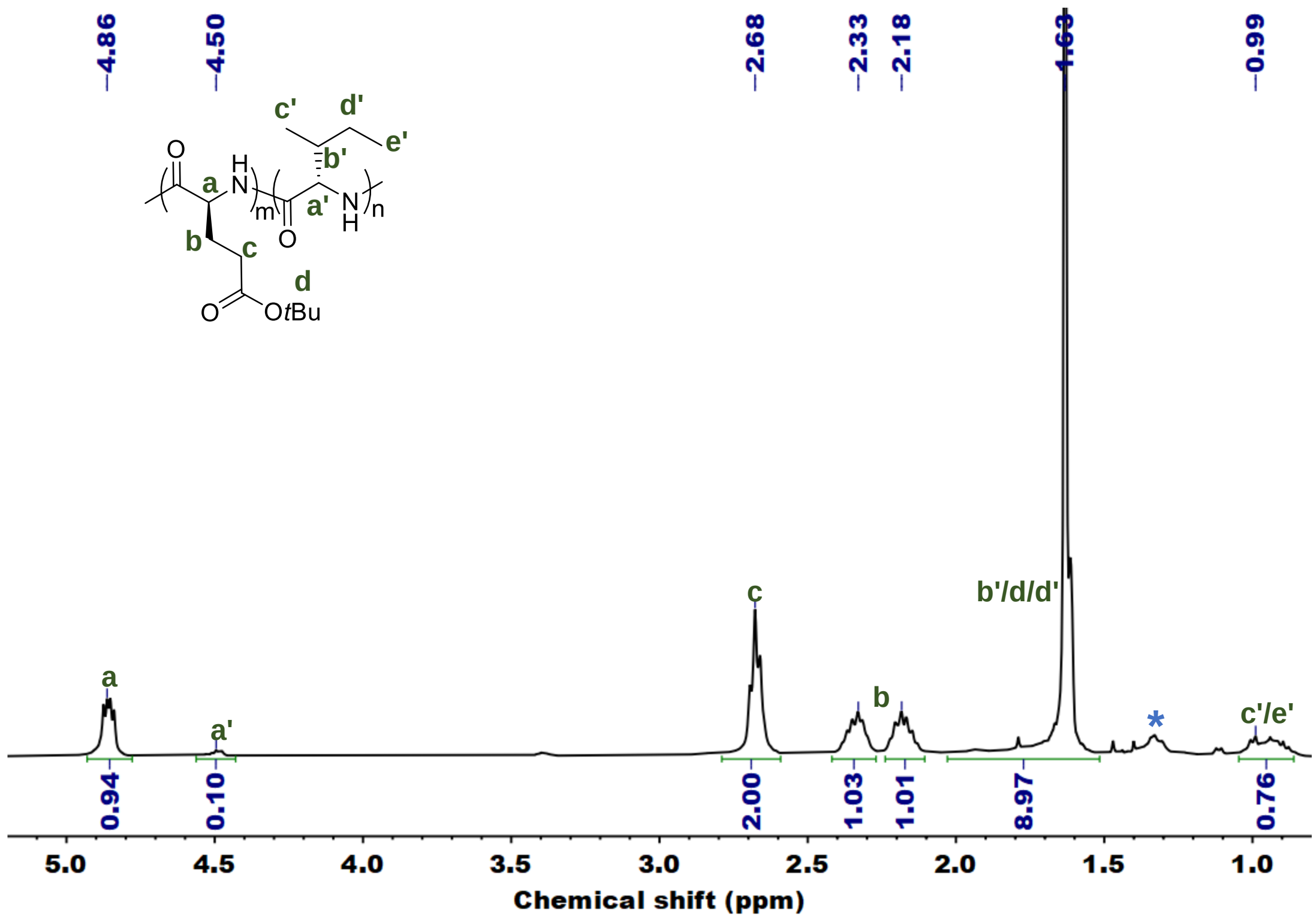

Supplementary Fig. 54. ^{1}H NMR spectrum (400 MHz) of SiE^{tBu}I in TFA-*d* after HF etching of silica cores.

The composition of SiE^{tBu}I was determined by comparing the integral of peaks at 2.68 ppm (i.e., γ-methylene protons of glutamic acid residues) to peaks at 0.99 ppm (i.e., methyl protons of isoleucine residues).

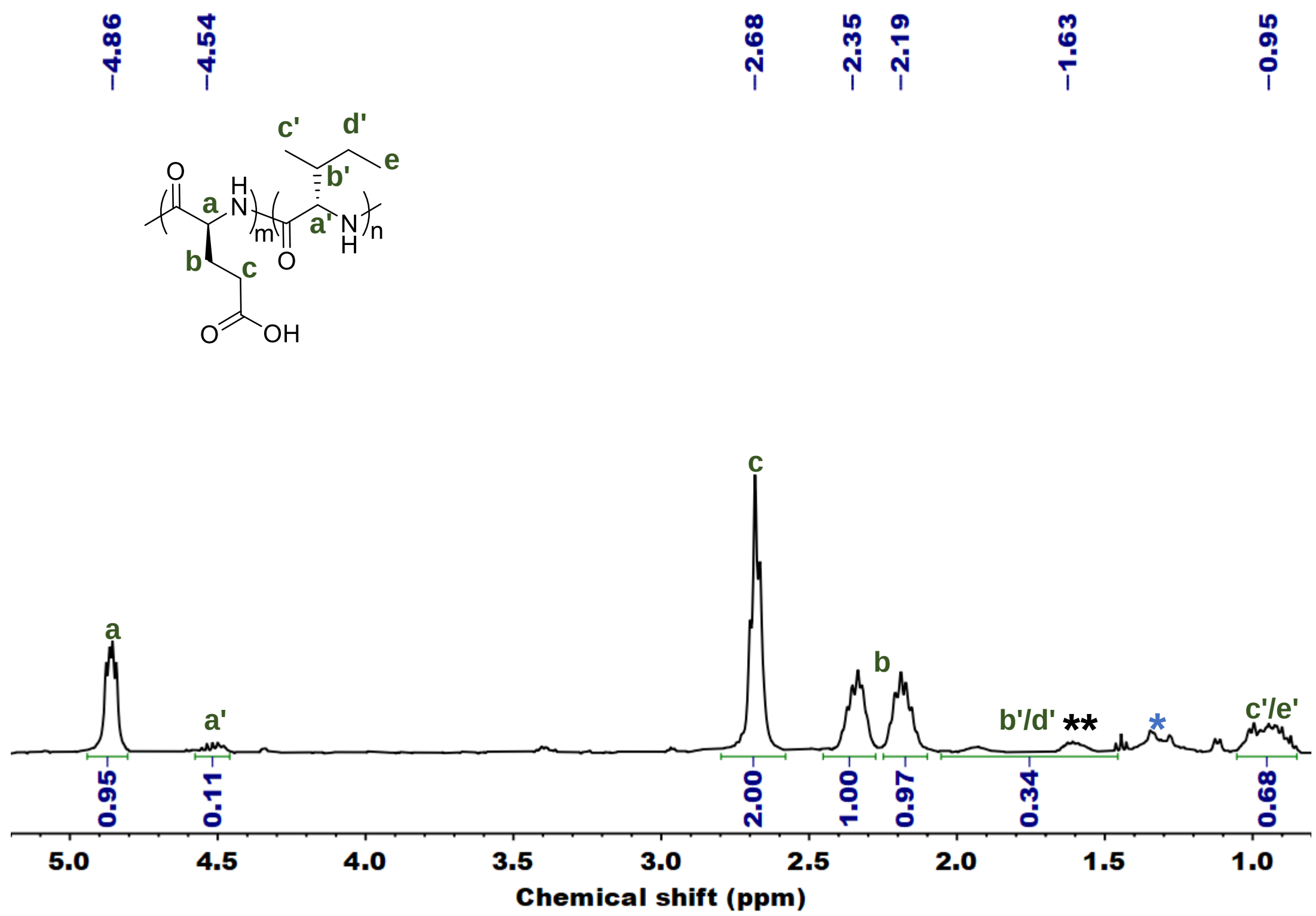

Supplementary Fig. 55. ^{1}H NMR spectrum (400 MHz) of SiEI in TFA-*d* after side-chain deprotection and HF etching of silica cores.

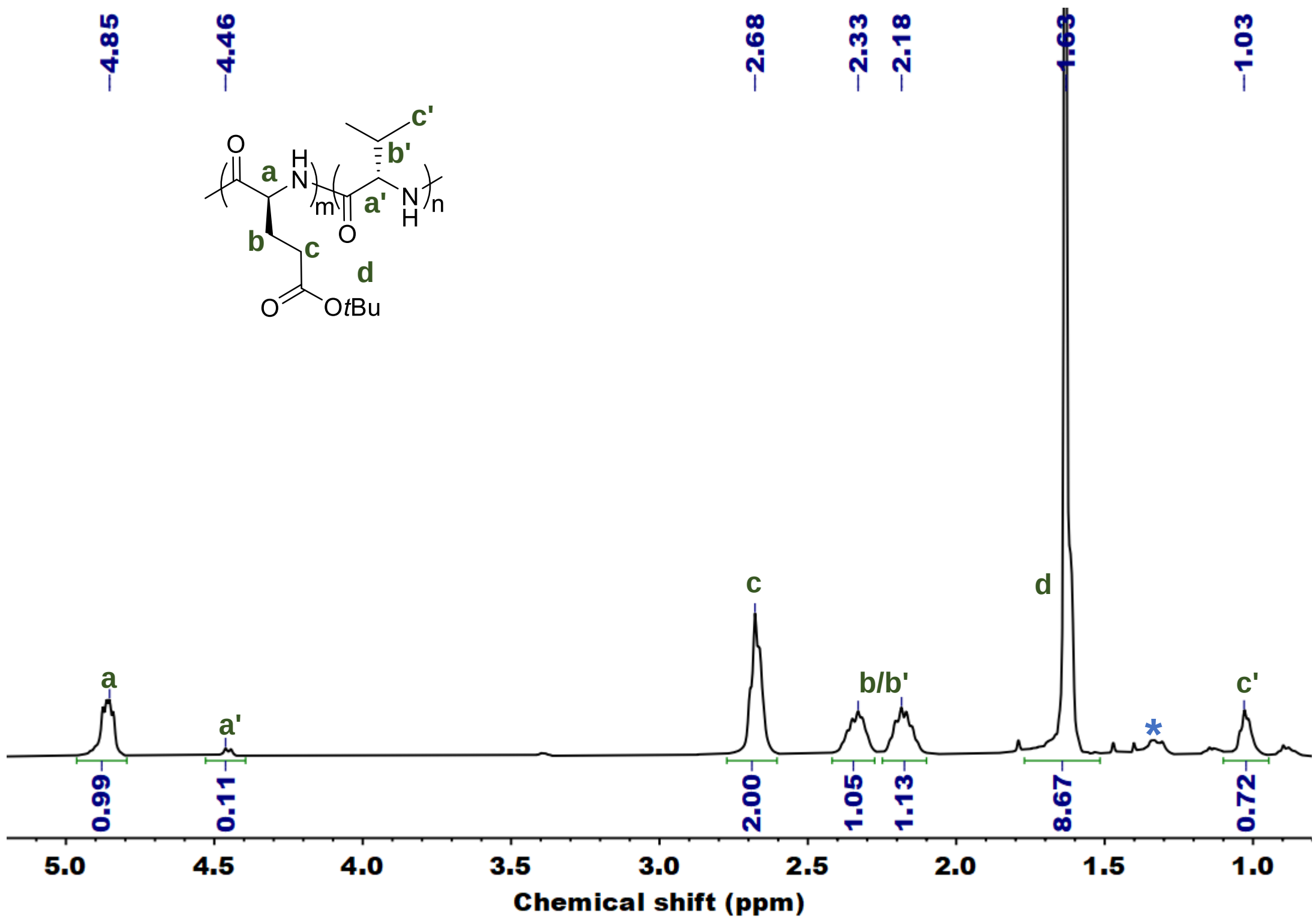

Supplementary Fig. 56. ^{1}H NMR spectrum (400 MHz) of SiE^{tBu}V in TFA-*d* after HF etching of silica cores.

The composition of SiE^{tBu}V was determined by comparing the integral of peaks at 2.68 ppm (i.e., γ-methylene protons of glutamic acid residues) to peaks at 1.03 ppm (i.e., methyl protons of valine residues).

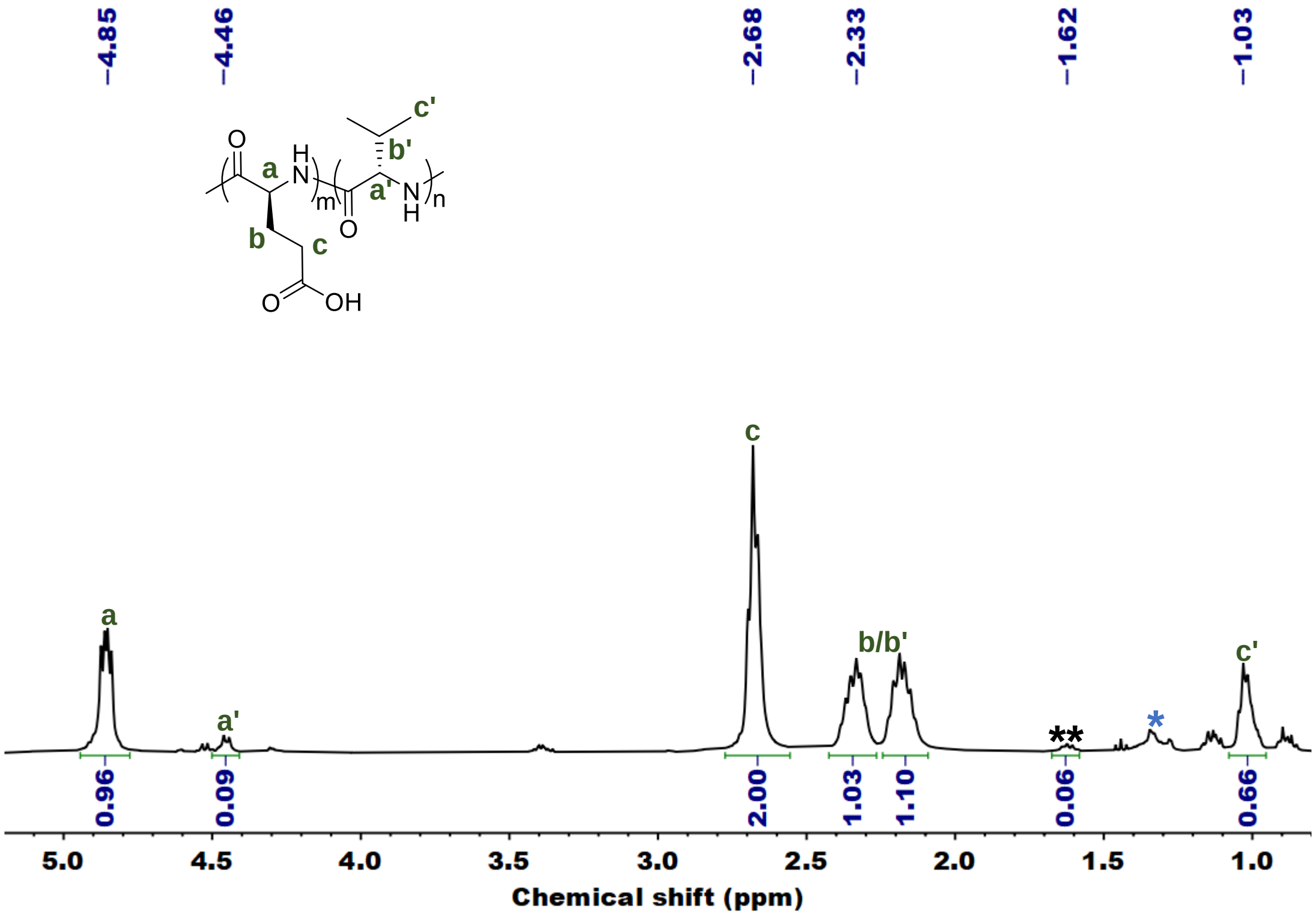

Supplementary Fig. 57. ^{1}H NMR spectrum (400 MHz) of SiEV in TFA-*d* after side-chain deprotection and HF etching of silica cores.

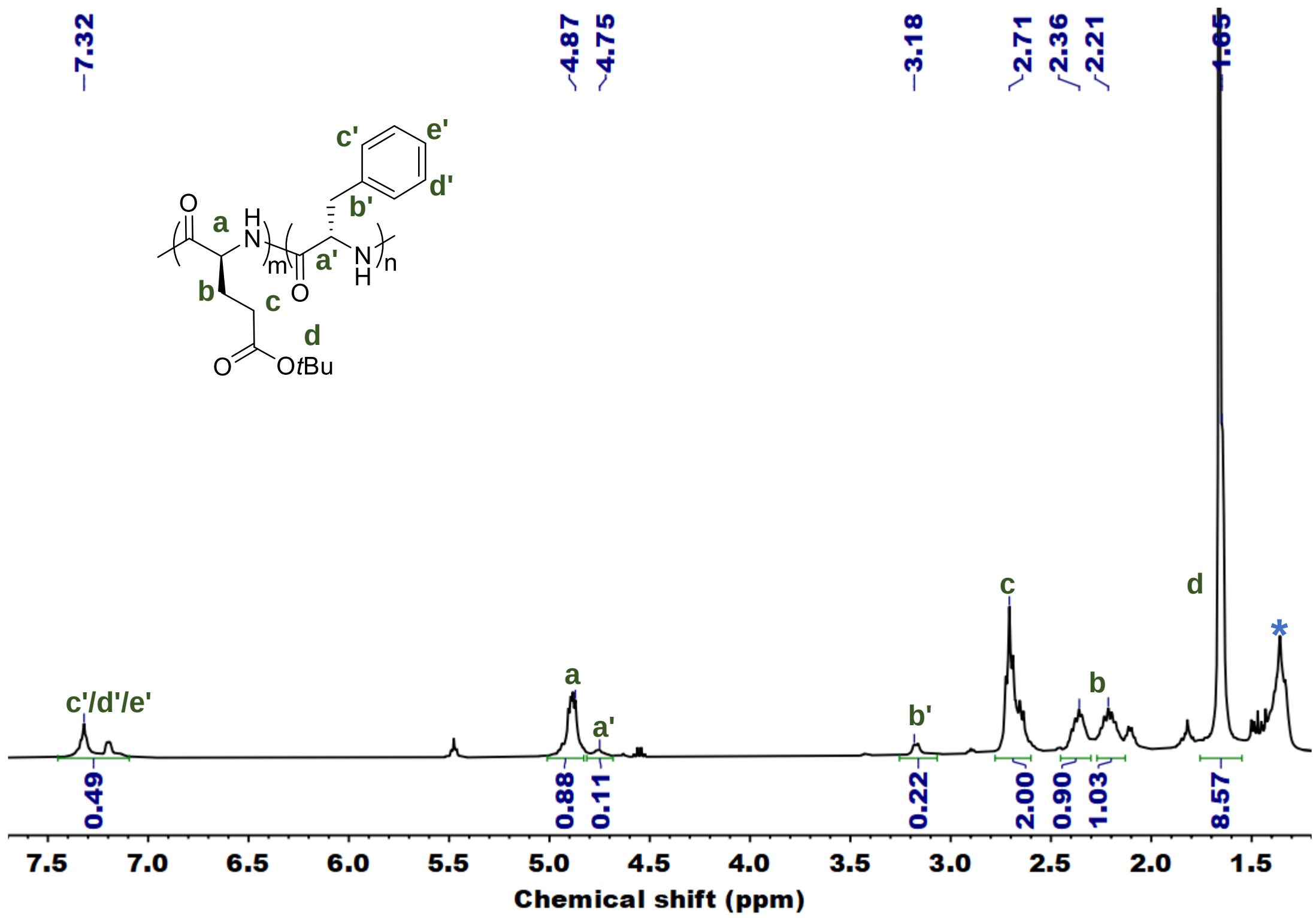

Supplementary Fig. 58. ^{1}H NMR spectrum (400 MHz) of SiE^{tBu}F in TFA-*d* after HF etching of silica cores.

The composition of SiE^{tBu}F was determined by comparing the integral of peaks at 2.71 ppm (i.e., γ-methylene protons of glutamic acid residues) to peaks at 7.32 ppm (i.e., aromatic protons of phenylalanine residues).

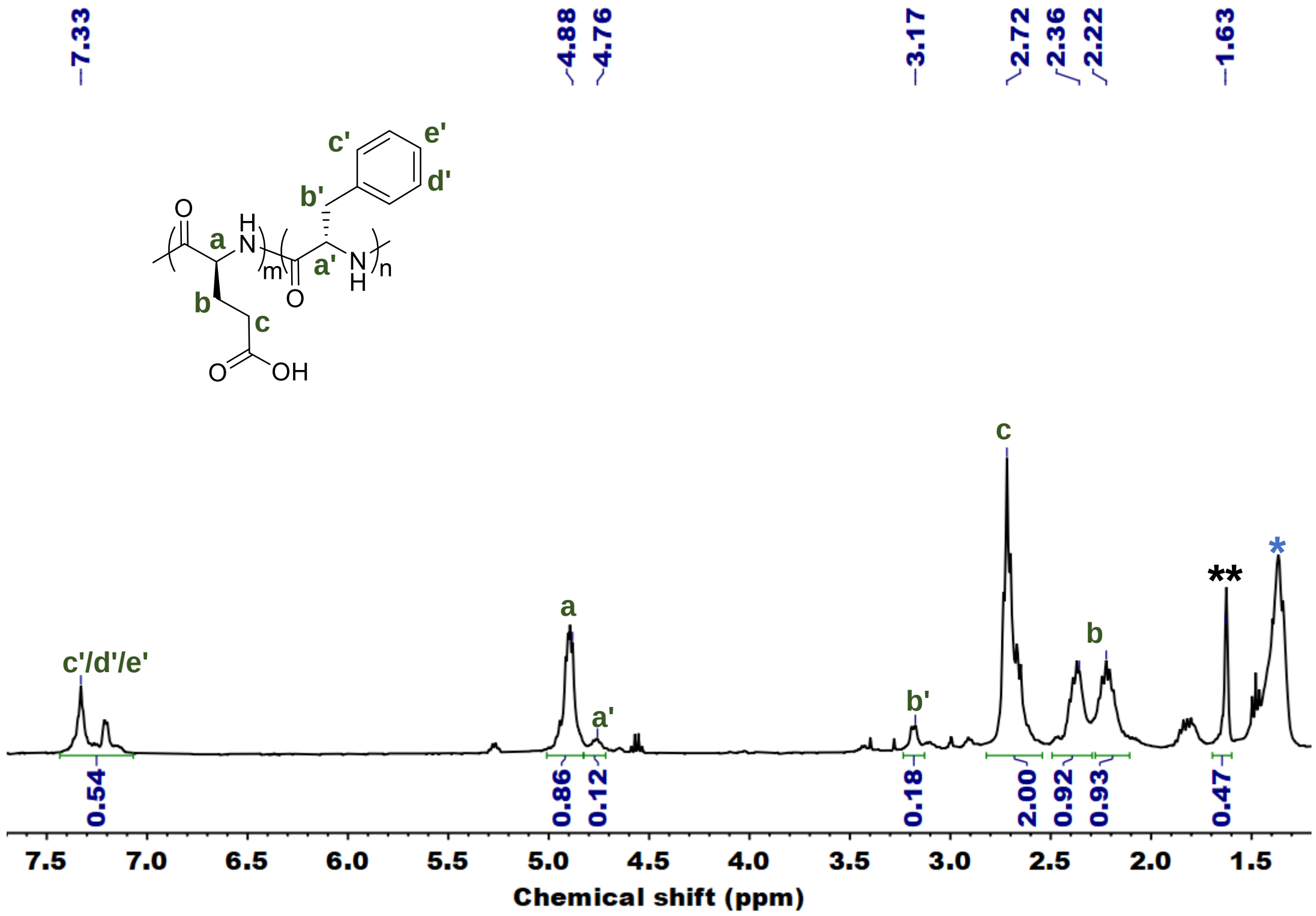

Supplementary Fig. 59. ^{1}H NMR spectrum (400 MHz) of SiEF in TFA-*d* after side-chain deprotection and HF etching of silica cores.

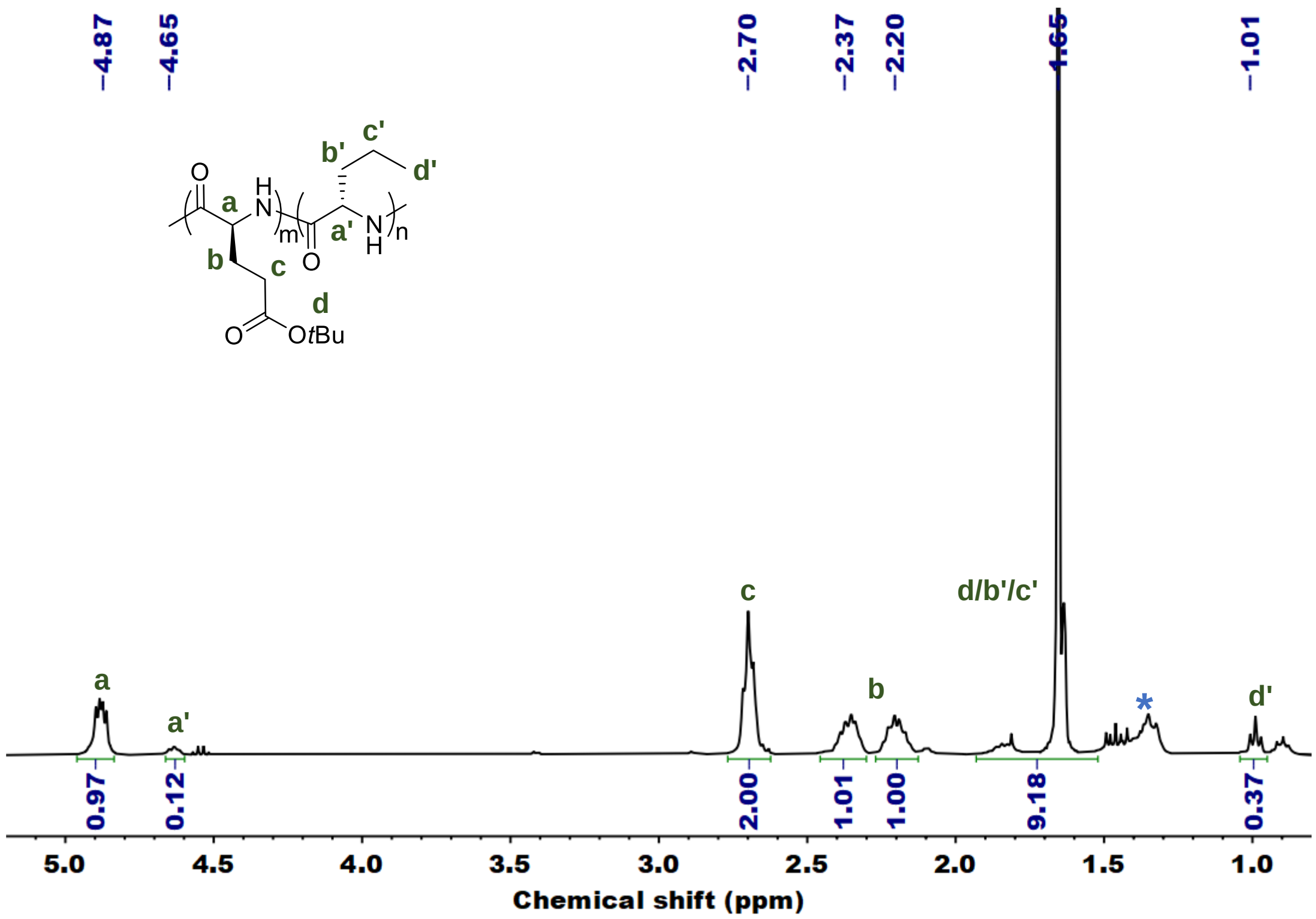

Supplementary Fig. 60. ^{1}H NMR spectrum (400 MHz) of SiE$^{t\mathrm{Bu}}$NV in TFA-*d* after HF etching of silica cores.

The composition of SiE$^{t\mathrm{Bu}}$NV was determined by comparing the integral of peaks at 2.70 ppm (i.e., γ-methylene protons of glutamic acid residues) to peaks at 1.01 ppm (i.e., methyl protons of norvaline residues).

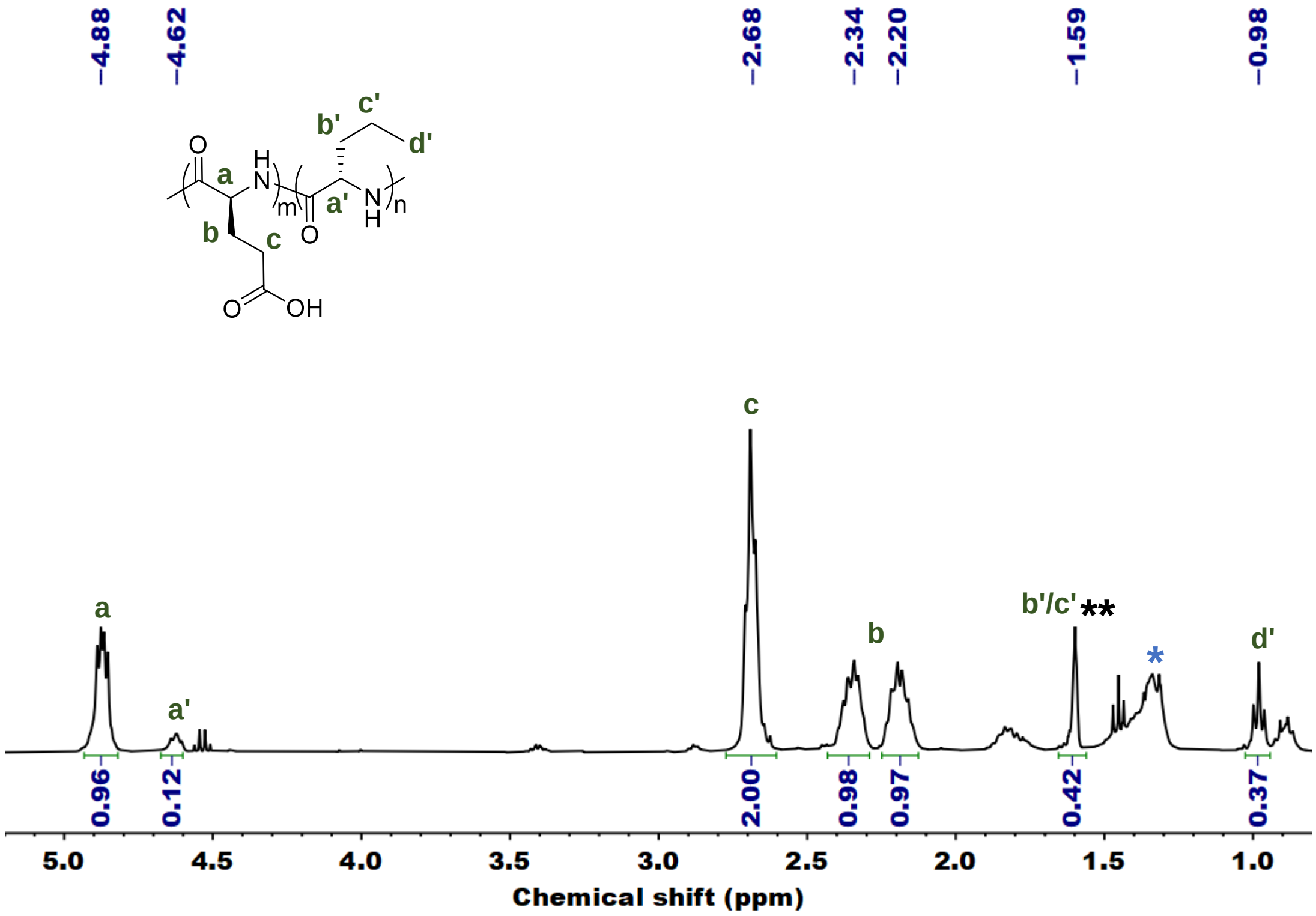

Supplementary Fig. 61. ^{1}H NMR spectrum (400 MHz) of SiENV in TFA-*d* after side-chain deprotection and HF etching of silica cores.

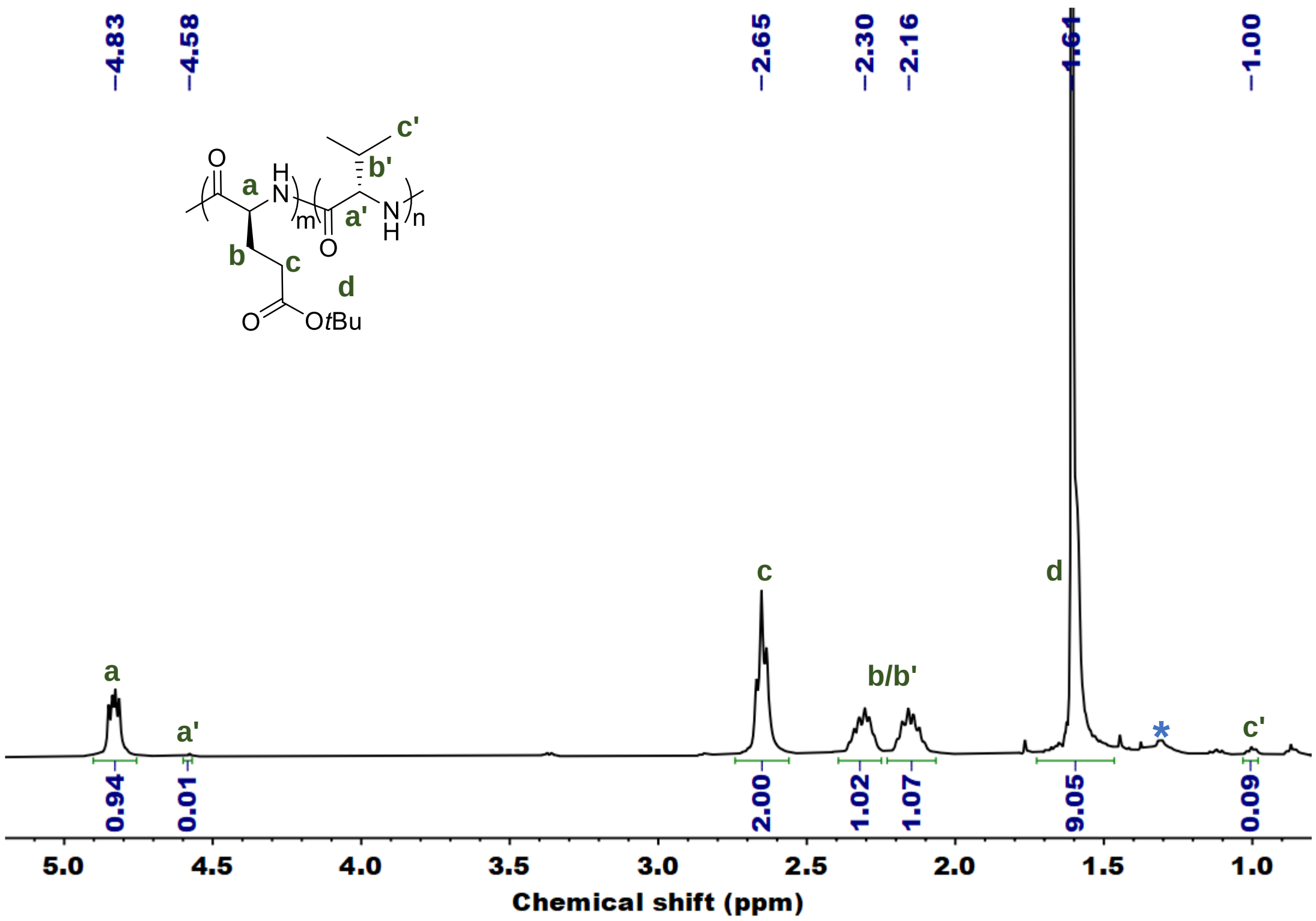

Supplementary Fig. 62. ^{1}H NMR spectrum (400 MHz) of SiE$^{t\mathrm{Bu}}$V with 1% valine in TFA-*d* after HF etching of silica cores.

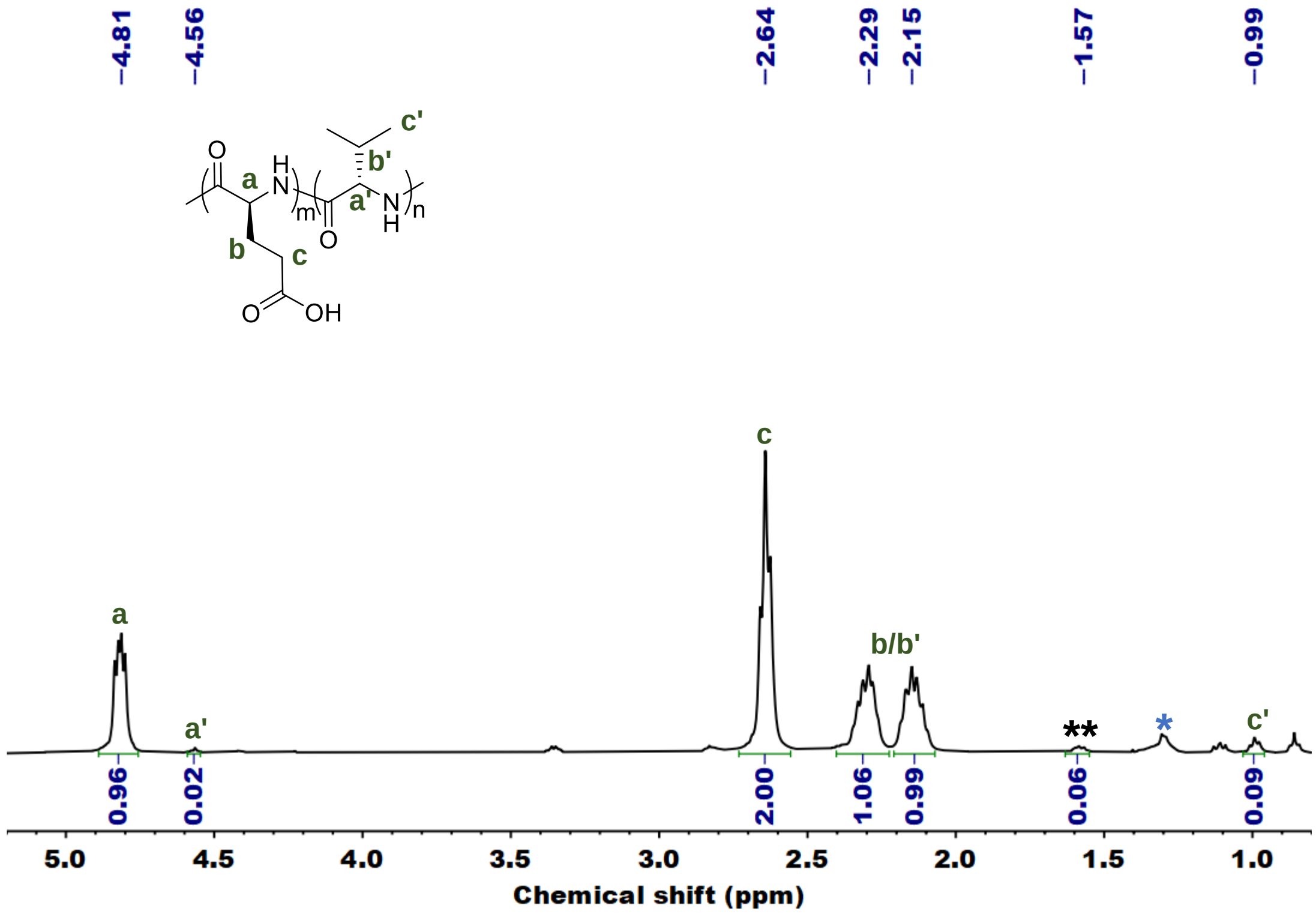

Supplementary Fig. 63. ^{1}H NMR spectrum (400 MHz) of SiEV with 1% valine in TFA-*d* after side-chain deprotection and HF etching of silica cores.

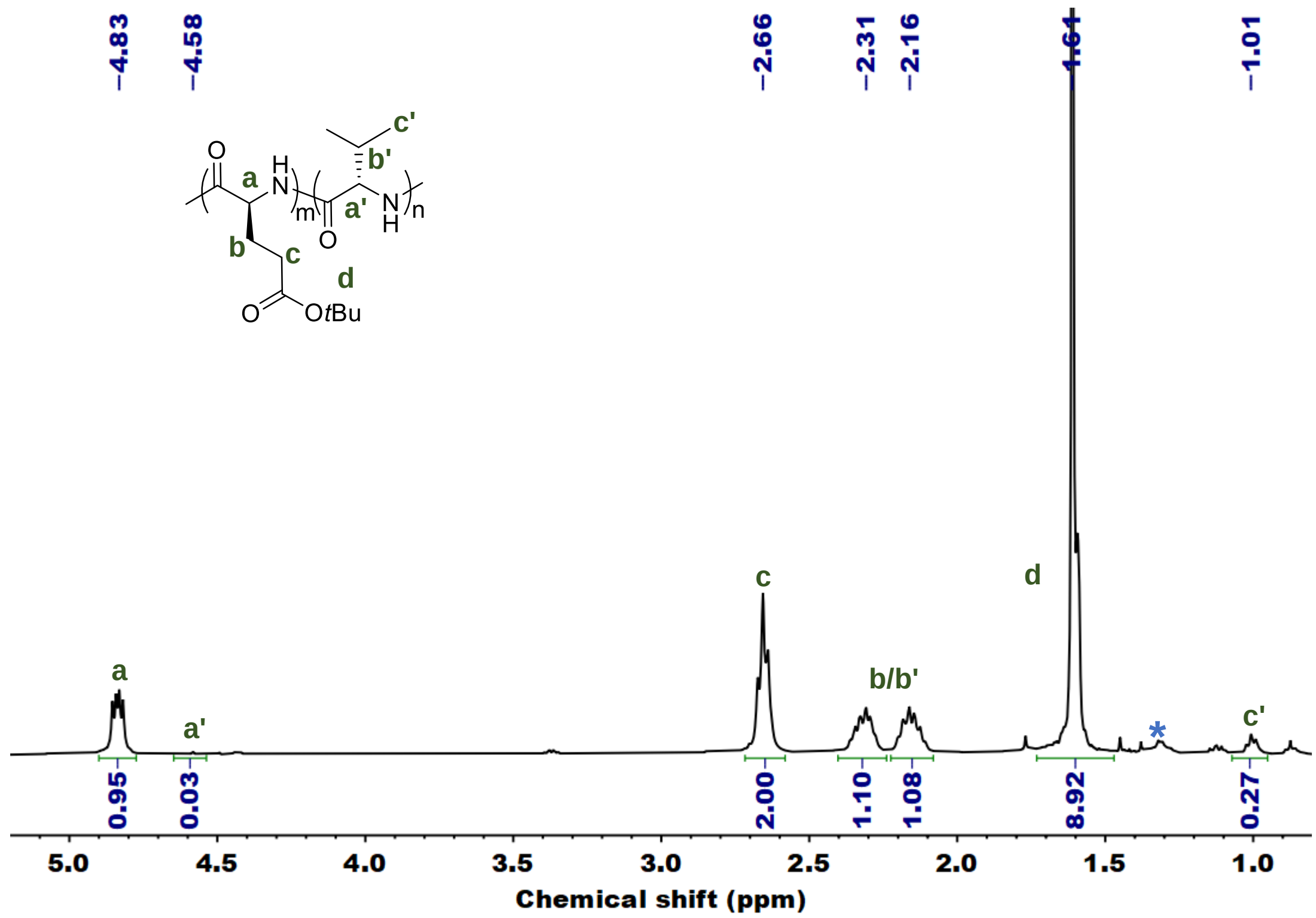

Supplementary Fig. 64. ^{1}H NMR spectrum (400 MHz) of SiE^{tBu}V with 5% valine in TFA-*d* after HF etching of silica cores.

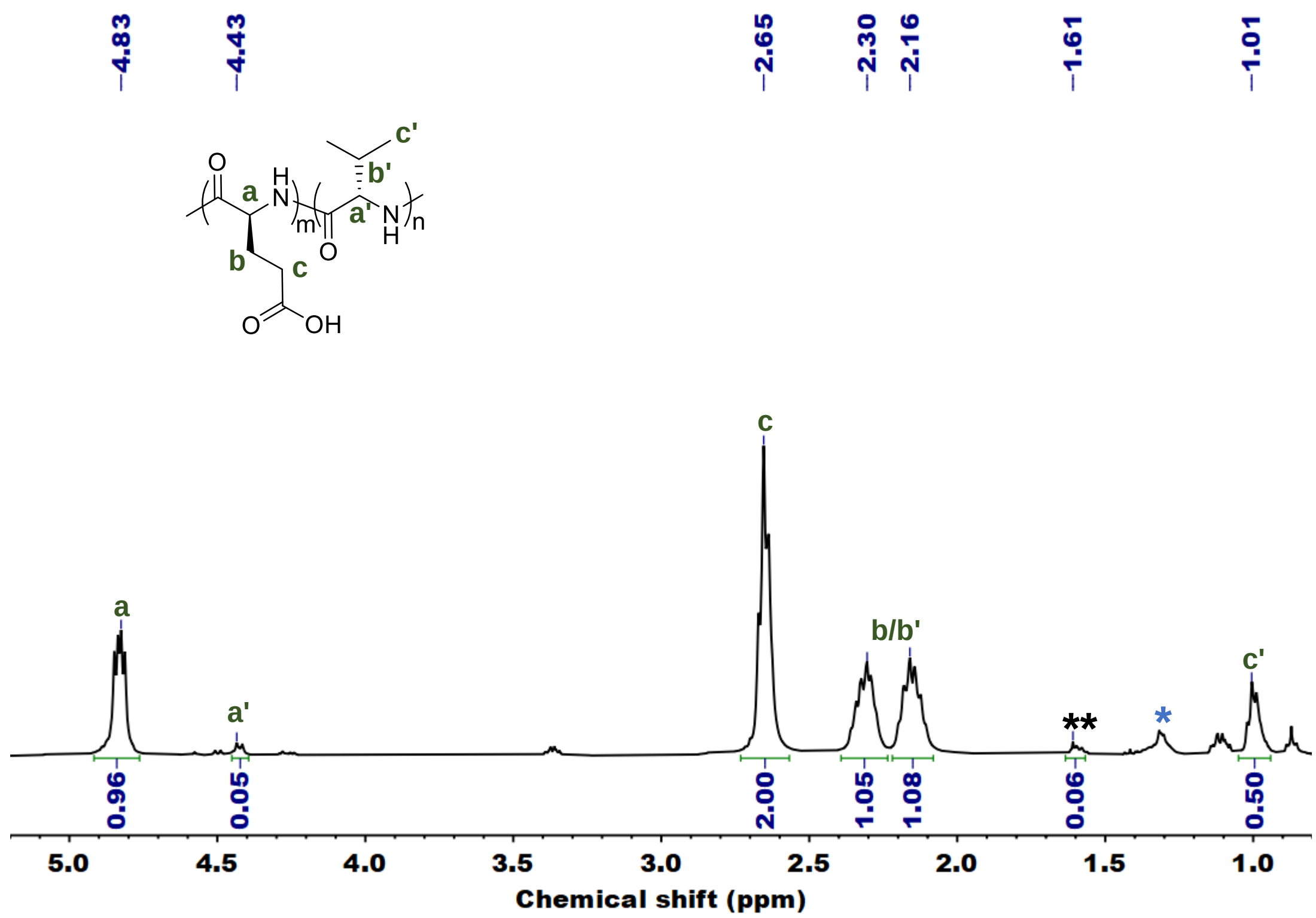

Supplementary Fig. 65. ^{1}H NMR spectrum (400 MHz) of SiEV with 5% valine in TFA-*d* after side-chain deprotection and HF etching of silica cores.

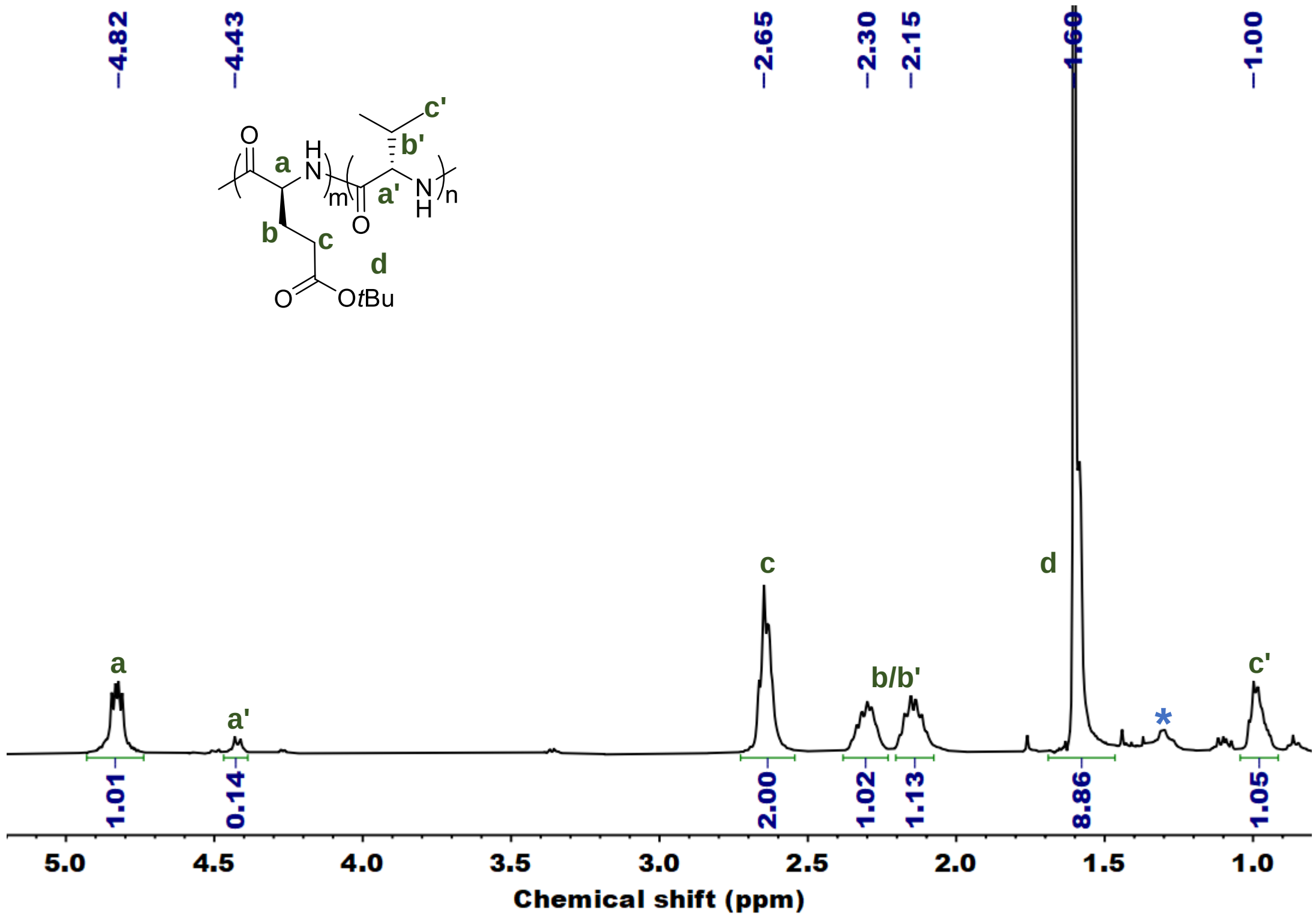

Supplementary Fig. 66. ^{1}H NMR spectrum (400 MHz) of SiE^{tBu}V with 15% valine in TFA-*d* after HF etching of silica cores.

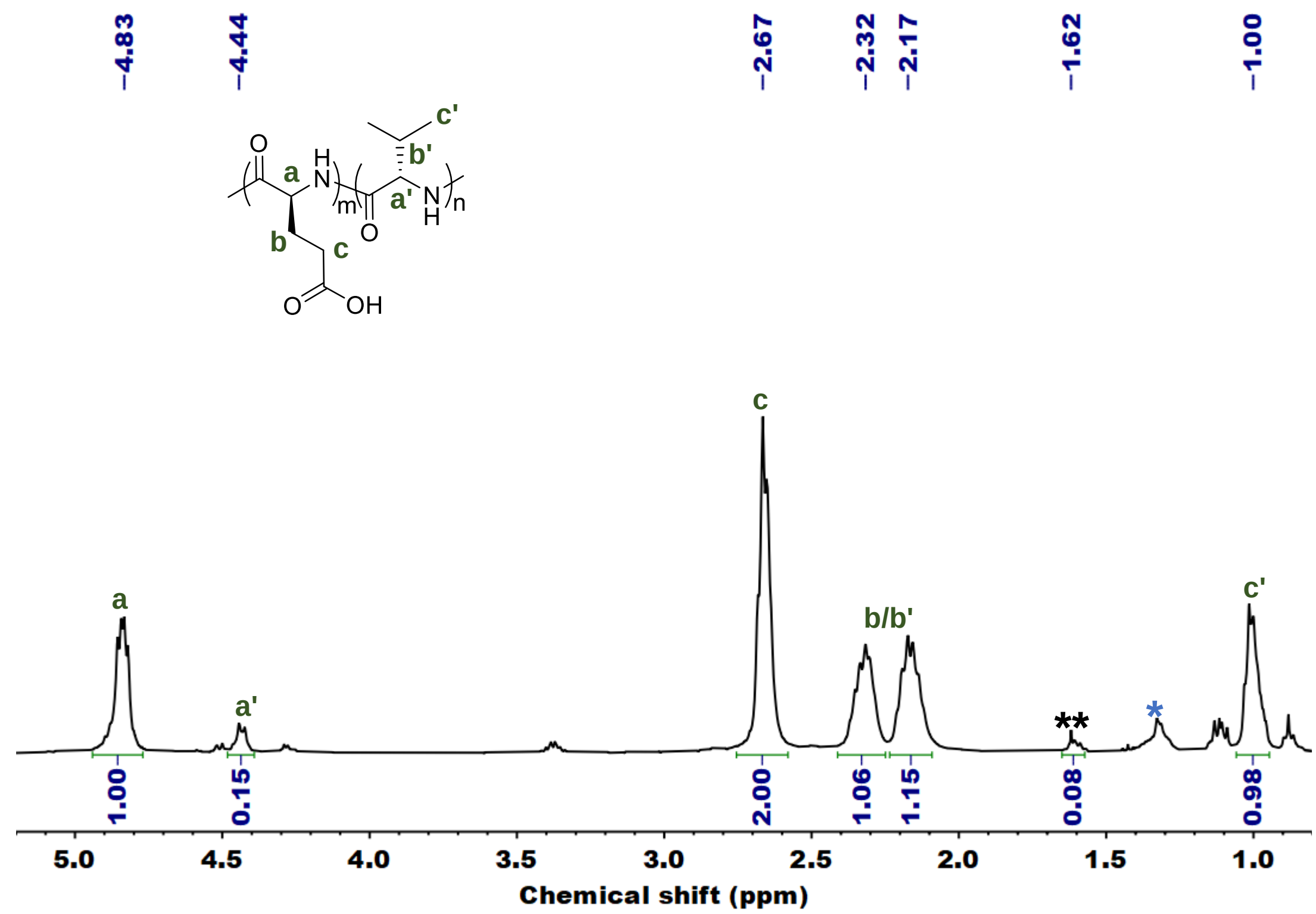

Supplementary Fig. 67. ^{1}H NMR spectrum (400 MHz) of SiEV with 15% valine in TFA-*d* after side-chain deprotection and HF etching of silica cores.

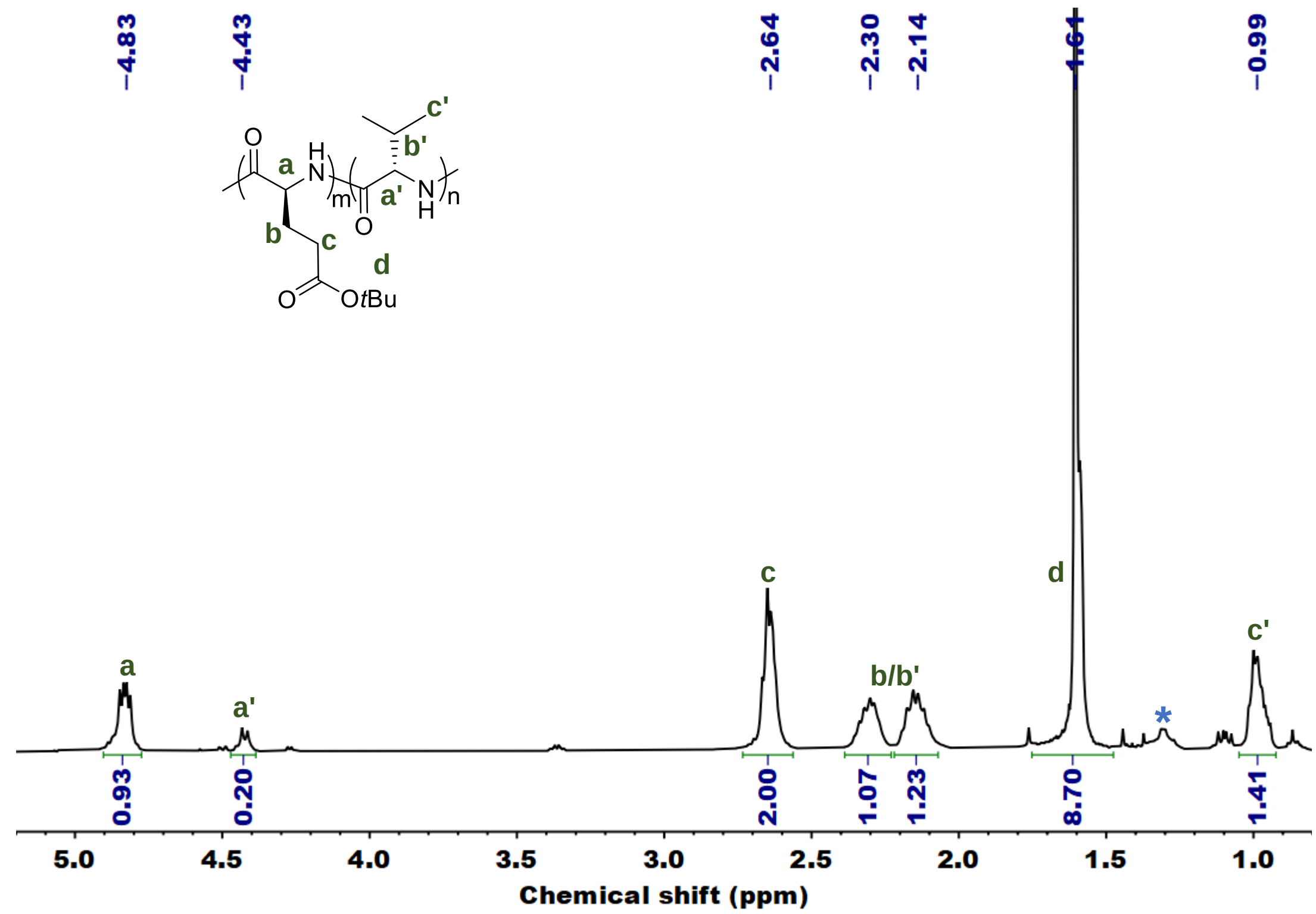

Supplementary Fig. 68. ^{1}H NMR spectrum (400 MHz) of SiE^{tBu}V with 20% valine in TFA-*d* after HF etching of silica cores.

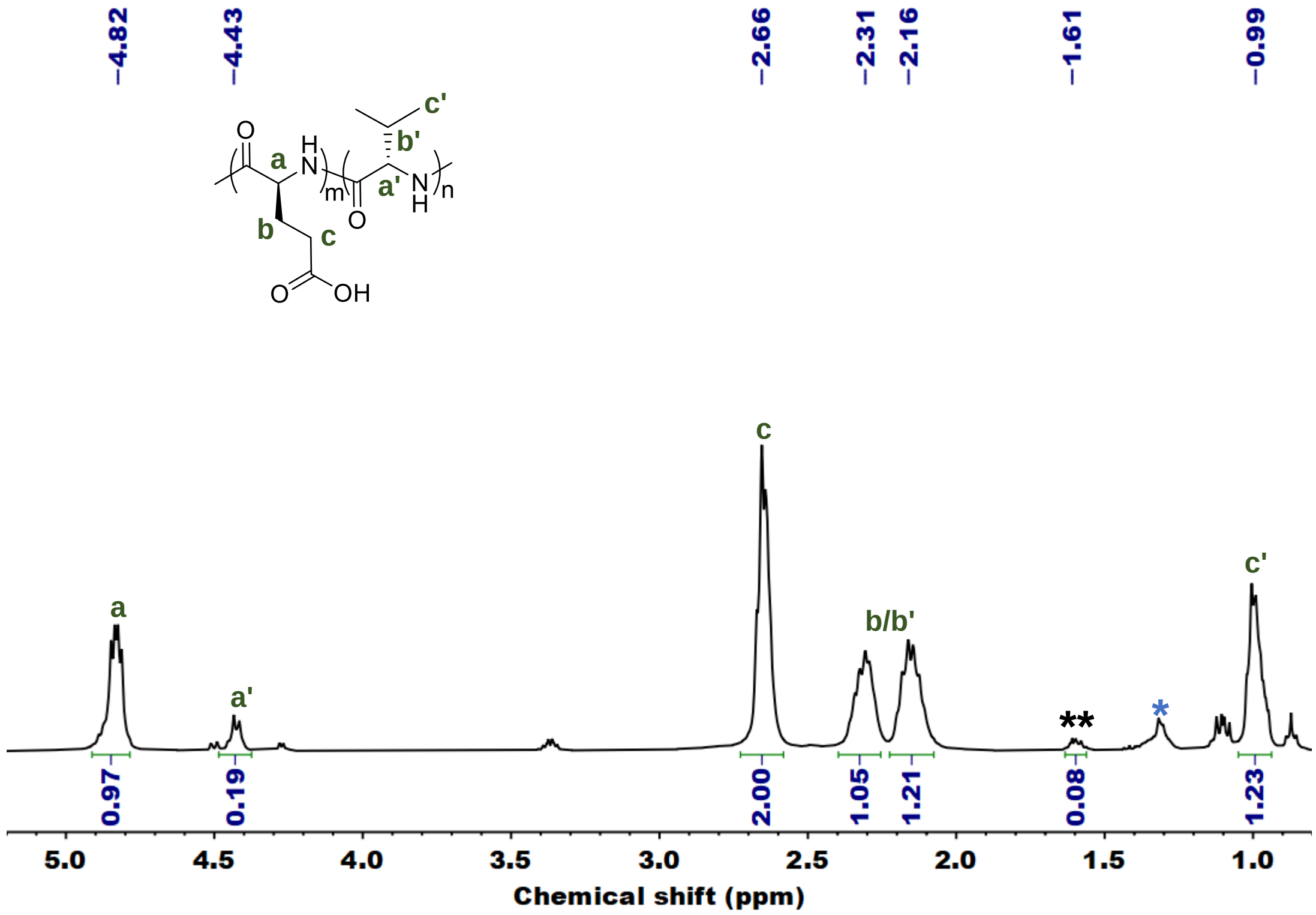

Supplementary Fig. 69. ^{1}H NMR spectrum (400 MHz) of SiEV with 20% valine in TFA-*d* after side-chain deprotection and HF etching of silica cores.

Supplementary Flowing Gating Strategies

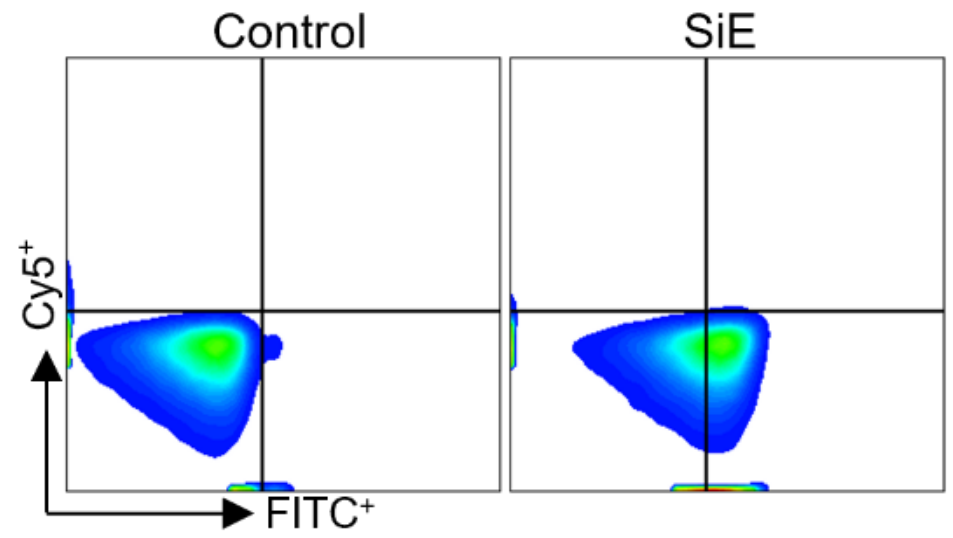

Supplementary Fig. 70. The FACS gating strategy for the analysis of Cy5+ expression in red blood cells (RBCs). FITC+ was used to define RBCs. Gates for Cy5+ were drawn based on RBCs incubated with PBS (n = 3 biologically independent samples). See Methods Section for the description of the antibodies used.

Figs. 2b, 3i, and Supplementary Figs. 15a, b all employed this strategy for the analysis of Cy5+ expression in RBCs.

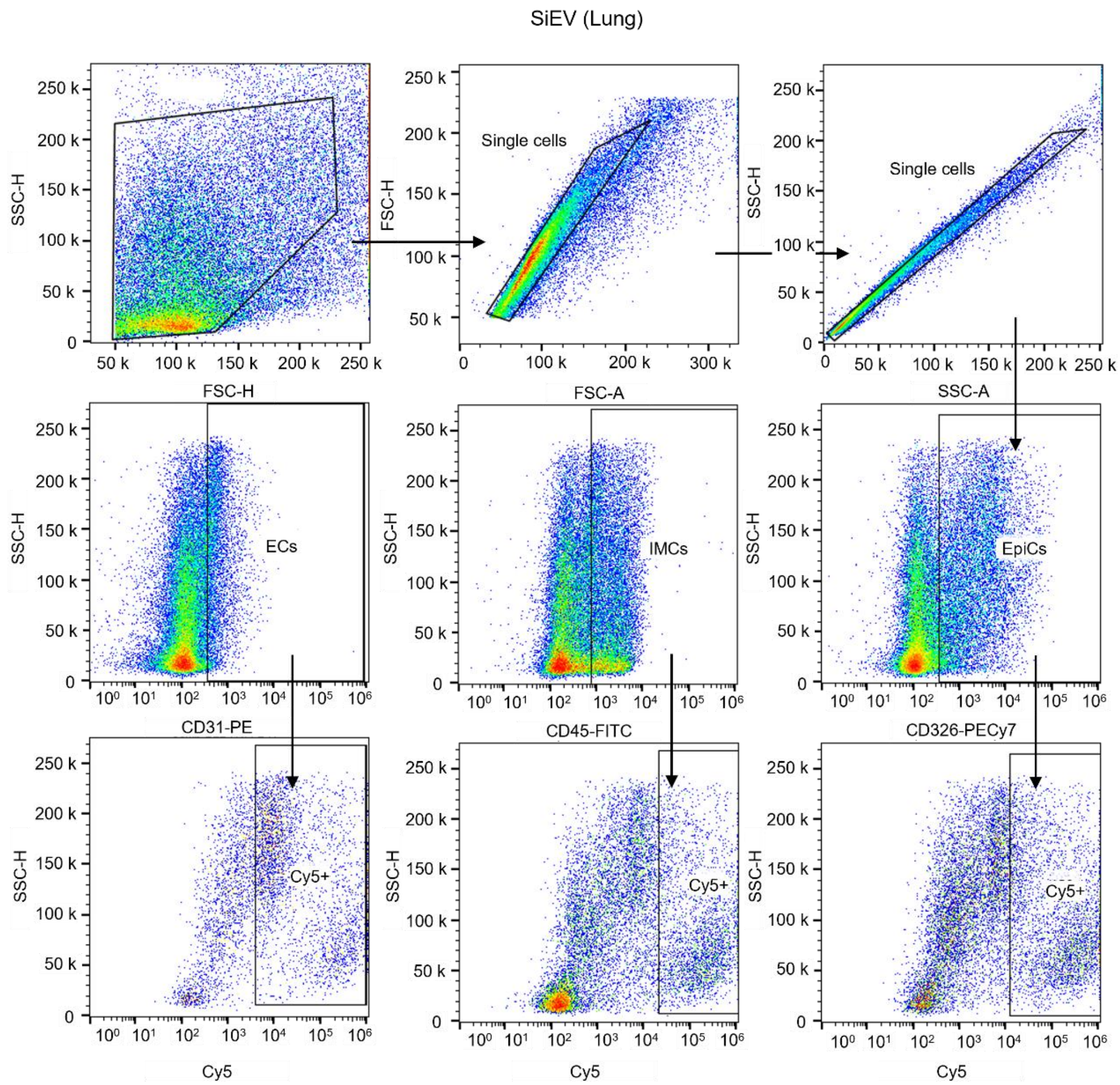

Supplementary Fig. 71. The FACS gating strategy for the analysis of Cy5+ expression in lung cells. CD45+ was used to define immune cells, CD31+ was used to define endothelial cells, and CD326+ was used to define epithelial cells. Gates for Cy5+ in cell types were drawn based on PBS-injected control mice (*n* = 3 female BALB/C mice per group, 6-8 weeks of age). See Methods Section for the description of the antibodies used.

Fig. 2f employed this strategy for the analysis of Cy5+ expression in lung cells.

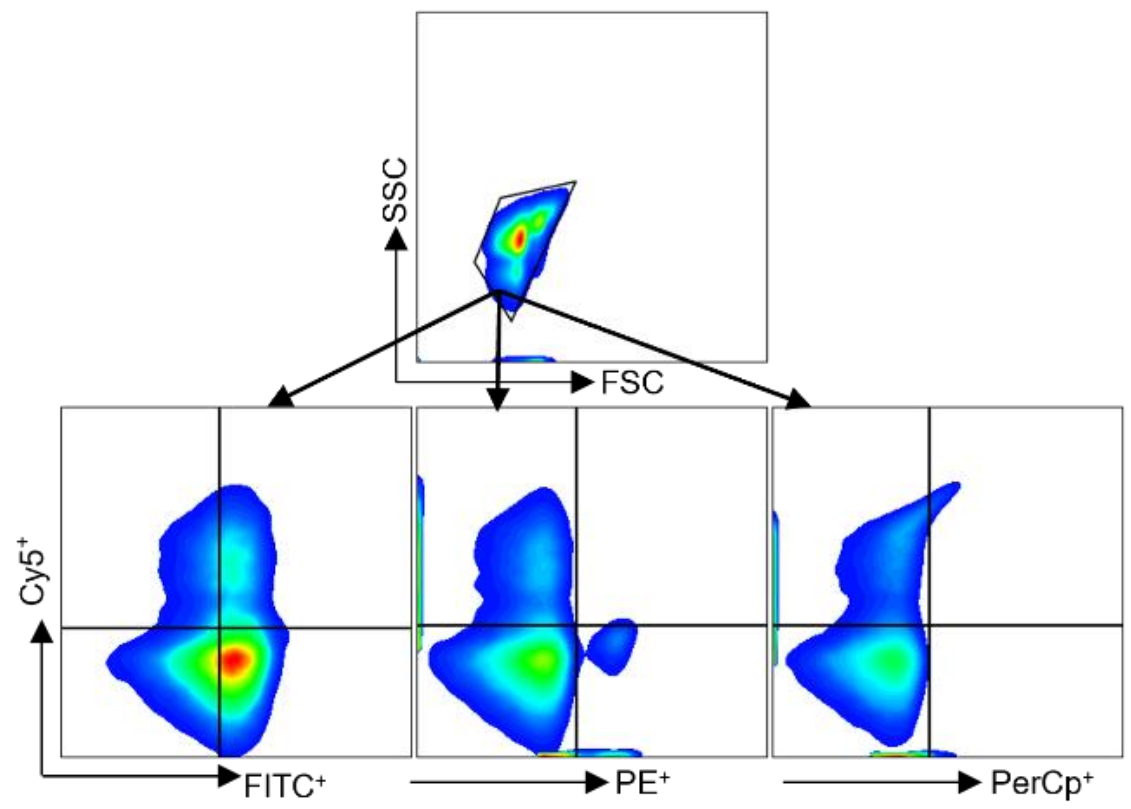

Supplementary Fig. 72. The FACS gating strategy for the analysis of Cy5+ expression in blood cells. CD45+ was used to define leukocytes, CD41+ was used to define platelets, and TER-119+ was used to define erythrocytes. Gates for Cy5+ in cell types were drawn based on PBS-injected control mice (*n* = 3 female BALB/C mice per group, 6-8 weeks of age). See Methods Section for the description of the antibodies used.

Supplementary Fig. 7a employed this strategy for the analysis of Cy5+ expression in blood cells.

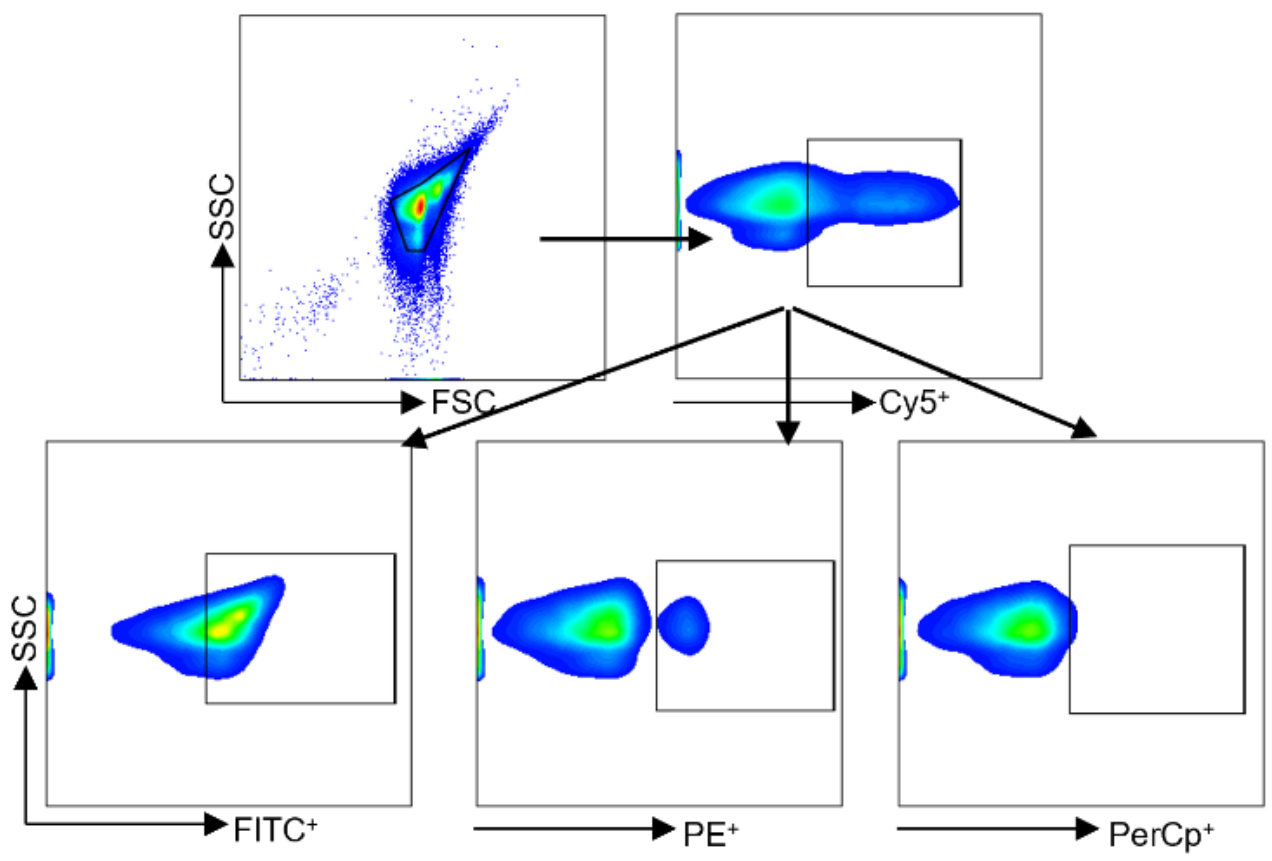

Supplementary Fig. 73. The FACS gating strategy for the analysis of the distribution of SiEV in various blood cells. CD45+ was used to define leukocytes, CD41+ was used to define platelets, and TER-119+ was used to define erythrocytes. Gates for Cy5+ in cell types were drawn based on PBS-injected control mice (*n* = 3 female BALB/C mice per group, 6-8 weeks of age). See Methods Section for the description of the antibodies used.

Supplementary Fig. 7b employed this strategy for the analysis of the distribution of SiEV in various blood cells.

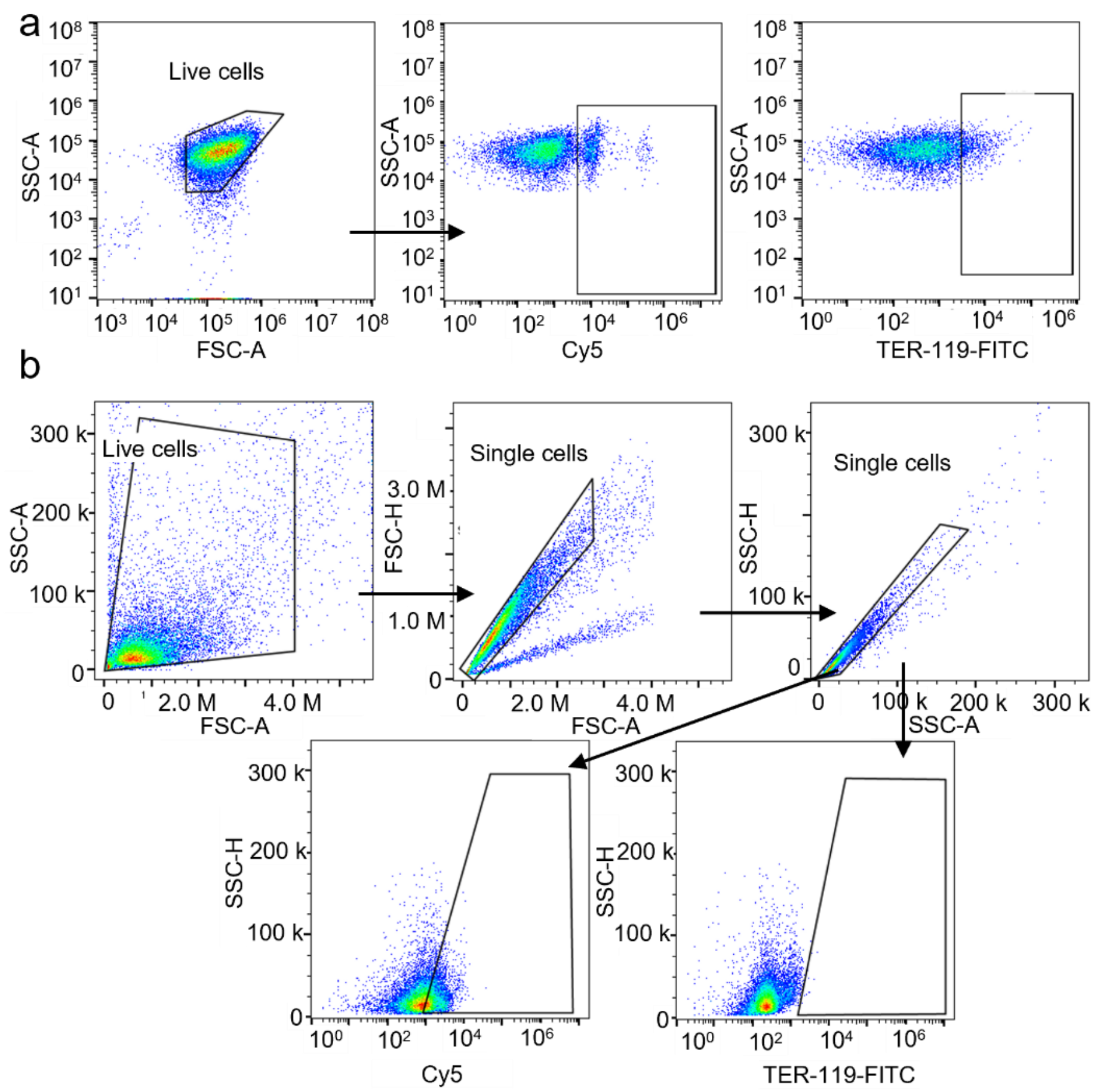

Supplementary Fig. 74. The FACS gating strategies for analysis of Cy5+ or FITC+ expression in blood cells (a) and lung cells (b). TER-119+ was used to define erythrocytes. Gates for Cy5+ in cell types were drawn based on PBS-injected control mice (*n* = 3 female BALB/C mice per group, 6-8 weeks of age). See Methods Section for the description of the antibodies used.

Supplementary Fig. 20 employed this strategy for the analysis of Cy5+ or FITC+ expression in blood cells and lung cells.
