# Supplementary material for: Sequence-regulated, lung-targeting heteropolypeptide nanoparticles via in situ erythrocyte hitchhiking
Source: Nat Commun. 2026 Jul 30;17:9251. doi: 10.1038/s41467-026-76188-x (PMC13526850; doi:10.1038/s41467-026-76188-x)
Supplement: Supplementary file 2 [file 41467_2026_76188_MOESM2_ESM.pdf]

nature portfolio

Corresponding author(s): Ziyuan Song, Lichen Yin

Last updated by author(s): Jun 29, 2026

Reporting Summary

Nature Portfolio wishes to improve the reproducibility of the work that we publish. This form provides structure for consistency and transparency in reporting. For further information on Nature Portfolio policies, see our Editorial Policies and the Editorial Policy Checklist.

Statistics

For all statistical analyses, confirm that the following items are present in the figure legend, table legend, main text, or Methods section.

n/a	Confirmed	
[ ]	[x]	The exact sample size (*n*) for each experimental group/condition, given as a discrete number and unit of measurement
[ ]	[x]	A statement on whether measurements were taken from distinct samples or whether the same sample was measured repeatedly
[ ]	[x]	The statistical test(s) used AND whether they are one- or two-sided<br>*Only common tests should be described solely by name; describe more complex techniques in the Methods section.*
[x]	[ ]	A description of all covariates tested
[x]	[ ]	A description of any assumptions or corrections, such as tests of normality and adjustment for multiple comparisons
[ ]	[x]	A full description of the statistical parameters including central tendency (e.g. means) or other basic estimates (e.g. regression coefficient) AND variation (e.g. standard deviation) or associated estimates of uncertainty (e.g. confidence intervals)
[ ]	[x]	For null hypothesis testing, the test statistic (e.g. *F*, *t*, *r*) with confidence intervals, effect sizes, degrees of freedom and *P* value noted<br>*Give P values as exact values whenever suitable.*
[x]	[ ]	For Bayesian analysis, information on the choice of priors and Markov chain Monte Carlo settings
[x]	[ ]	For hierarchical and complex designs, identification of the appropriate level for tests and full reporting of outcomes
[x]	[ ]	Estimates of effect sizes (e.g. Cohen's *d*, Pearson's *r*), indicating how they were calculated

Our web collection on statistics for biologists contains articles on many of the points above.

Software and code

Policy information about availability of computer code

Data collection

Burker AVANCE NEO-400 MHZ.
Zeta-sizer Nano ZS90 (Malvern) .
Tecnai G2 F20 electron microscope (FEI Nano Ports).
Zeiss Gemini 500 microscope (Carl Zeiss).
Gel permeation chromatography (Agilent).
ZEISS LSM 800 microscope (Carl Zeiss).
BioTek Synergy H1 multifunctional microplate reader (Agilent).
BD Accuri C6 Plus flow cytometer (BD Biosciences).
PerkinElmer in vivo imaging systems (PerkinElmer).
Inductively coupled plasma-optical emission spectrometer (PerkinElmer).
WIZARD Gamma counter (PerkinElmer).

Data analysis	MestReNova software version 6.1.0.<br>FlowJo software version 10.8.1.<br>GraphPad Prism software version 9.4.1.<br>ZEN software.<br>LivingImage verison 4.4.<br>ImageJ software.<br>CaseViewer 2.3 software.

For manuscripts utilizing custom algorithms or software that are central to the research but not yet described in published literature, software must be made available to editors and reviewers. We strongly encourage code deposition in a community repository (e.g. GitHub). See the Nature Portfolio guidelines for submitting code & software for further information.

Data

Policy information about availability of data

All manuscripts must include a data availability statement. This statement should provide the following information, where applicable:

- Accession codes, unique identifiers, or web links for publicly available datasets
- A description of any restrictions on data availability
- For clinical datasets or third party data, please ensure that the statement adheres to our policy

All data generated in this study are provided in the Supplementary Information and Source Data file. Source data are provided with this paper.

Research involving human participants, their data, or biological material

Policy information about studies with human participants or human data. See also policy information about sex, gender (identity/presentation), and sexual orientation and race, ethnicity and racism.

Reporting on sex and gender	N/A
Reporting on race, ethnicity, or other socially relevant groupings	N/A
Population characteristics	N/A
Recruitment	N/A
Ethics oversight	N/A

Note that full information on the approval of the study protocol must also be provided in the manuscript.

Field-specific reporting

Please select the one below that is the best fit for your research. If you are not sure, read the appropriate sections before making your selection.

☒ Life sciences ☐ Behavioural & social sciences ☐ Ecological, evolutionary & environmental sciences

For a reference copy of the document with all sections, see nature.com/documents/nr-reporting-summary-flat.pdf

Life sciences study design

All studies must disclose on these points even when the disclosure is negative.

Sample size	Sample sizes were determined using statistical power calculations. For various assays, we performed at least three replicates to yield statistically significant differences.
Data exclusions	No data were excluded from the analysis.
Replication	We confirm that all results were replicated successfully in multiple experiments. The statistical tests used in this manuscript have been described in both the Statistics section and the Figure captions.
Randomization	All samples/organisms in our experiments were randomly divided into each group.
Blinding	Due to the proof-of-concept developmental nature of this study, true blinding of experiments was not performed. However, data collection and analysis for some experiments were performed by separate individuals. In some cases, these collectors/analyzers were not aware which samples corresponded to which experimental groups at the time of data collection and analysis.

Reporting for specific materials, systems and methods

We require information from authors about some types of materials, experimental systems and methods used in many studies. Here, indicate whether each material, system or method listed is relevant to your study. If you are not sure if a list item applies to your research, read the appropriate section before selecting a response.

Materials & experimental systems

n/a	Involved in the study
[ ]	[x] Antibodies
[ ]	[x] Eukaryotic cell lines
[x]	[ ] Palaeontology and archaeology
[ ]	[x] Animals and other organisms
[x]	[ ] Clinical data
[x]	[ ] Dual use research of concern
[x]	[ ] Plants

Methods

n/a	Involved in the study
[x]	[ ] ChIP-seq
[ ]	[x] Flow cytometry
[x]	[ ] MRI-based neuroimaging

Antibodies

Antibodies used	FITC anti-mouse CD45 (1:200, catalog no. 147709, clone I3/2.3), PE-Cy7 anti-mouse CD326 (1:100, catalog no. 118215, clone G8.8), PE anti-mouse CD31 (1:100, catalog no. 160203, clone G8.8), FITC anti-mouse TER-119/Erythroid cells (1:100, catalog no. 116205, clone TER-119), PE anti-mouse CD41 (1:200, catalog no. 133905, clone MWReg30), and PerCP anti-mouse CD45 (1:100, catalog no. 103129, clone 30-F11) were purchased from BioLegend (San Diego, USA). Anti-TNF-α rabbit multiclonal primary antibody (1:500, catalog no. ab307164, clone RM1005), anti-IL-6 rabbit monoclonal primary antibody (1:100, catalog no. ab290750, clone EPR23819-103), anti-IL-1β rabbit multiclonal primary antibody (1:500, catalog no. ab283818, clone RM1009), anti-iNOS mouse monoclonal primary antibody (1:1000, catalog no. ab210823, clone EPR16635), and anti-CD206 rabbit polyclonal primary antibody (1:1000, catalog no. ab64693) were purchased from Abcam (Cambridge, Britain).
Validation	All antibodies are quality control tested by commercial supplier for the application.<br>FITC anti-mouse CD45 antibody: https://www.biolegend.com/en-gb/products/fitc-anti-mouse-cd45-antibody-9796<br>PE-Cy7 anti-mouse CD326 antibody: https://www.biolegend.com/en-gb/products/pe-cyanine7-anti-mouse-cd326-ep-cam-antibody-5303<br>PE anti-mouse CD31 antibody: https://www.biolegend.com/en-gb/products/pe-anti-mouse-cd31-pecam-1-antibody-19418<br>PE anti-mouse CD41 antibody: https://www.biolegend.com/en-gb/products/pe-anti-mouse-cd41-antibody-5897<br>PerCP anti-mouse CD45 antibody: https://www.biolegend.com/en-gb/products/percp-anti-mouse-cd45-antibody-4265<br>FITC anti-mouse TER-119/Erythroid cells: https://www.biolegend.com/en-us/search-results?Keywords=ter+119<br>Anti-TNF-α rabbit multiclonal primary antibody: https://www.abcam.cn/products/primary-antibodies/tnf-alpha-antibody-rm1005-ab307164<br>Anti-IL-6 rabbit monoclonal primary antibody: https://www.abcam.cn/products/primary-antibodies/il-6-antibody-epr23819-103-bsa-and-azide-free-ab290750<br>Anti-IL-1β rabbit multiclonal primary antibody: https://www.abcam.cn/products/primary-antibodies/il-1-beta-antibody-rm1009-ab283818<br>Anti-iNOS mouse monoclonal primary antibody: https://www.abcam.cn/products/primary-antibodies/inos-antibody-epr16635-mouse-igg2b-chimeric-ab210823<br>Anti-CD206 rabbit polyclonal primary antibody: https://www.abcam.cn/products/primary-antibodies/mannose-receptor-antibody-ab64693

Eukaryotic cell lines

Policy information about cell lines and Sex and Gender in Research

Cell line source(s)	RAW 264.7 (mouse monocyte macrophages) and iMPMVEC cells (Immortalized mouse pulmonary microvascular endothelial cells) were obtained from American Type Culture Collection.
Authentication	Cell lines have been authentication using STR.
Mycoplasma contamination	Cell lines were not detected for mycoplasma contamination.
Commonly misidentified lines (See ICLAC register)	No commonly misidentified lines were used.

Animals and other research organisms

Policy information about studies involving animals; ARRIVE guidelines recommended for reporting animal research, and Sex and Gender in Research

Laboratory animals	Male and female BALB/c mice (6-8 weeks) were obtained from Cavens Model Animal Co., Ltd (Changzhou, China), and housed in a specific pathogen-free (SPF) room with a 12-h light/dark cycle, standard environmental temperature of 25 °C, and humidity of 40-50%. All animal procedures were performed according to the NIH guidelines for the care and use of laboratory animals (NIH Publication 85-23 Rev. 1985) and were approved by the Animal Ethics Committee of Soochow University.

Wild animals	No wild animals were used.
Reporting on sex	Female and male mice were used. Biological distribution studies typically focus on universal pharmacokinetic principles, where sex is often not considered a primary variable. In contrast, in the ALI model, male mice were specifically selected for two principal reasons: (1) To eliminate the potential confounding effects of the estrous cycle, as fluctuating levels of female sex hormones are known to modulate immune and inflammatory pathways; (2) To maintain consistency with the predominant use of male animals in existing literature, thereby ensuring our data are comparable and can be contextualized within an established research framework.
Field-collected samples	No filed-collected samples were used.
Ethics oversight	All animal experiments in this study are approved by the Animal Ethics Committee of Soochow University and performed in accordance with the guidelines of the Animal Ethics Committee of Soochow University.

Note that full information on the approval of the study protocol must also be provided in the manuscript.

Plants

Seed stocks	N/A
Novel plant genotypes	N/A
Authentication	N/A

Flow Cytometry

Plots

Confirm that:

- [x] The axis labels state the marker and fluorochrome used (e.g. CD4-FITC).
- [x] The axis scales are clearly visible. Include numbers along axes only for bottom left plot of group (a 'group' is an analysis of identical markers).
- [x] All plots are contour plots with outliers or pseudocolor plots.
- [x] A numerical value for number of cells or percentage (with statistics) is provided.

Methodology

Sample preparation

For isolation and staining of lung cells, the lung tissues were minced using a sterile blade and digested with trypsin (500 µL, 30 min). The lung homogenates were filtered using a 70-µm filter and washed once with PBS. After centrifugation (900 × g, 5 min), the supernatant was removed and the cell pellets were re-suspended in RBC lysis buffer (5 mL), which was incubated on ice for 5 min. The mixture was then centrifuged (900 × g, 5 min) and the cells were re-suspended in cell staining buffer. The cell suspensions were then added into flow tubes with antibodies (total volume 100 µL), which were incubated for 30 min in the dark at 4 oC. The stained cells were washed twice with PBS and re-suspended in PBS (100 µL) for flow cytometric analysis. The antibodies used were FITC anti-mouse CD45, PE-Cy7 anti-mouse CD326, and PE anti-mouse CD31.

For the in vivo analysis of erythrocyte-SiEV complexation, healthy female BALB/c mice were injected intravenously with SiEV and SiENV (1 mg/mL, 100 µL). Blood from healthy mice was collected in anticoagulated blood tubes at 1 h post injection, which was subsequently centrifuged to remove the top layer of plasma (900 × g, 10 min). The erythrocyte suspension was stained with FITC-labeled WGA (0.1 µM) for 5 min in the dark, and flow cytometry was used to quantitatively assess the binding of NPs to erythrocyte membranes. Moreover, in order to simulate the lung-accumulation of SiEV through shear-induced NP release from erythrocytes in pulmonary capillaries, the formed complexes were extruded through syringes and the desorption of SiEV was quantified according to the decrease in Cy5 fluorescent signals. First, erythrocyte suspension was incubated with SiEV for 1 h, which was then gently washed twice with PBS to remove the free NPs. The suspension of SiEV-erythrocyte complexes was then washed 3 times with PBS by extrusion using a syringe, which was evaluated quantitatively by flow cytometry. The desorption percentage was determined compared to the complexes without additional extrusion treatment.

To investigate the interactions between SiEV and blood cells (erythrocytes, leukocytes, and platelets), healthy BALB/c mice were intravenously injected with SiEV (1 mg/mL, 100 µL). Blood from healthy mice was collected in anticoagulated blood tubes at 1 h post i.v. injection, which was subsequently centrifuged to remove the top layer of plasma (900 × g, 10 min). The suspension was stained with FITC anti-mouse TER-119/Erythroid cells, PE anti-mouse CD41, and PerCP anti-mouse CD45. SiEV-positive cells were then quantified using flow cytometry.

FITC-labeled RBCs were pre-incubated with Cy5-labeled SiEV, and the resulting complexes were then intravenously injected into the mice (1 mg/mL, 100 µL). At pre-determined time points post injection (1, 6, and 12 h), blood from mice was collected in anticoagulated blood tubes and then mice were euthanized to collect lungs. Flow cytometry was used to analyze the signals of FITC and Cy5 in the blood and lung tissues, respectively. Mice that were intravenously injected with only FITC-labeled RBCs were used as the control group.

Instrument	BD Accuri C6 Plus flow cytometer (BD Biosciences).
Software	FlowJo software version 10.8.1.
Cell population abundance	N/A
Gating strategy	For isolation and staining of lung cells, FSC and SSC plots were firstly used to gate the cells. Then, the intensity in the FITC, PE, and PE-Cy7 channels to analyze the uptake of NPs by cells.<br>For the analysis of NP-erythrocyte complexation, TER119-FITC and NPs-Cy5 were used to gate the cells.<br>For the interactions between SiEV and blood cells, FSC and SSC plots were firstly used to gate the cells. Then, the intensities in the FITC, PE, and PerCP channels to analyze the uptake of NPs by cells.<br>For the assessment of SiEV percentage distribution in various blood cells, cells were first gated using the Cy5 plot. The intensities recorded in the FITC, PE, and PerCP channels were then analyzed to determine the distribution profile of SiEV.<br>To analyze the levels of SiEV and RBC in blood or lungs at different time points, cells were gated using FSC and SSC plots, followed by analysis of the intensities in the FITC and Cy5 channels.

☒ Tick this box to confirm that a figure exemplifying the gating strategy is provided in the Supplementary Information.
