# Supplementary material for: Sequence-regulated, lung-targeting heteropolypeptide nanoparticles via in situ erythrocyte hitchhiking
Source: Nat Commun. 2026 Jul 30;17:9251. doi: 10.1038/s41467-026-76188-x (PMC13526850; doi:10.1038/s41467-026-76188-x)
Supplement: Supplementary file 3 [file 41467_2026_76188_MOESM3_ESM.pdf]

nature portfolio

Peer Review File

Sequence-Regulated, Lung-Targeting Heteropolypeptide Nanoparticles via *in situ* Erythrocyte Hitchhiking

Corresponding Author: Professor Ziyuan Song

This file contains all reviewer reports in order by version, followed by all author rebuttals in order by version.

Version 0:

Reviewer comments:

Reviewer #1

(Remarks to the Author)
This is an interesting study that deserves constructive analysis and collegial encouragement. I hope that authors will take my comments in this context. There are several methodological aspects that MUST be addressed before the manuscript may be reconsidered for publication. The main issues on hand are the following.

The biomedical aspects of this study need more attention and work. The authors propose a new type of lung-targeted NP formulation, which has not been studied like liposomes or LNPs. This is the first publication on this NP (unless the authors insist otherwise), nothing is known about their fate and activities in the body. This question begs the detailed characterization of the fate of NP after injection, performed using adequate means (see below). The more inclusive molecular and cellular schemas of targeting and effect are needed. What the side effect of accumulation of silica NP in the lungs is? Memento Asbestosis?

Methods for characterization of the pharmacokinetics (PK) in blood and tissues based on using optical probes DOES NOT characterize the blood level, and uptake in the organs (including the lungs). Optical data cannot adequately prove the quantitative parameters. These data are distorted by uncontrolled rate of self-quenching, tissue transparency in given segments of the spectrum, tissue background in healthy vs pathological conditions. The daunting challenges of interpretation optical signal is clearly illustrated by problem of blood: RBC have so strong signal of Hb that it dwarfs or distorts any optical probe. Only radioisotopes afford the needed quality of objective, precise and accurate data.

Studies of in vivo injection of the particles described in this and other papers MUST include a simple control on selectivity of the uptake in the lungs, i.e., injection into an artery feeding perfusion of organs of interest. If the pulmonary uptake of your particles measured by isotope labels of particle and RBC remain exactly the same as after IV injection (by isotope tracing), congratulations: apparently your approach does afford some tissue-specific targeting to the lungs. This result will warrant investigations of the mechanism underlying such an unusual and important result.

Interaction with RBC must include effects of NP on RBC in vitro and in vivo (hemolysis by complement and osmotic stress, agglutination, persistence in the circulation and uptake by spleen and other organs). Have you measured Hb in the medium after filtering RBC-NP? The super of control RBCs in Supplement f4 look reddish; why it looks blue in the third tube? F5 shows full lysis with water – please show the calibration curve of Hb A and describe the incubation conditions. To study hitchhiking you must have RBC labeled by 51Cr and cargo NP labeled by 125Iodine, 111In or other appropriate isotope that can de clearly and accurately discerned from RBC label. Only this dual-labeling approach, described in RBC-HH studies allows to distinguish the transfer from RBC to the vascular cells from mechanical, charge or other non-specific RETENTION or UPTAKE of the whole RBC-cargo complex in the microvasculature downstream injection site. This is especially important in the RBC-HH studies, because this mechanism of delivery is prone to these non-specific features.

Particles may engage diverse cells in blood and organs, including marginated leukocytes in the pulmonary vasculature, RES and other sites. It became known recently that interaction of particles with leukocytes and other reactive components of host defense may lead to unexpected consequences, such as hitchhiking particles by blood cells headed to the CNS.

The nanoparticles devised in this study are very interesting. If (it is an important “if”) the phenomenon and its specificity will be proven as requested, the question of what the tentative mechanism of the reported “specific precise delivery into the

pulmonary endothelium is needed. Which component of nanoparticles recognize specifically and precisely pulmonary endothelium? In which vascular segment? Which surface marker on endothelium represents the binding site for these NP provides the precision and selectivity of the delivery?

A singular interpretation of the interesting results pertaining to the functional effects as the result of specific and precise delivery of the described particles to the pulmonary endothelium is NOT established. The observed results may represent the effects of interactions of the injected materials with numerous migratory and resident pulmonary constituencies.

There are issues with literature. The second sentence in the abstract says: "the rational design of lung-targeted nanocarriers through fine-tuning of molecular structures remains challenging'. This reviewer prefers to take this as a human error rather than either intentional misleading attempt to self-inflate, or lack of relevant knowledge. Lots of studies established specific targeted delivery to the pulmonary endothelium of a plethora of cargoes, carriers and conjugates using ligands of PECAM, ACE, thrombomodulin, caveolar determinants, etc., published in PNAS, Nature Series, ACS Nano, Blood, etc.. Based on the literature and direct isotope tracing nanoparticles, the pulmonary endothelium is very precisely served by particles binding to ACE, TM and PV1, whereas PECAM ligands work as pan-endothelial binding sites. Even cargo for these truly pulmonary targeting studies have been similar to the current study, including quencher of superoxide and hydrogen peroxide. Authors need to compare their approach and results obtained with previous studies focused on specific targeting of agents to the pulmonary endothelium. What exactly is better in the submitted manuscript in terms of targeting or protective effects of targeted delivery to EC of SOD and catalase in animal models of ALI, pulmonary embolism, radiation injury, inflammation, edema – all of which were responding to these targeted therapeutics with clear and compelling improvement?

Authors embarked on the testing protective effects of this drug delivery system in animal models of human lung diseases. Scientifically speaking, this direction of the continuation of this research project needs to answer the questions above first. This is especially relevant to the studies of such sort based on measuring totally non-specific markers of non-specific pathological conditions such as oxidative burst driven and manifested by ROS, MDA, etc.

Reviewer #2

(Remarks to the Author)
The manuscript presents an interesting and potentially impactful strategy for lung endothelial enrichment that links heteropolypeptide sequence effects to RBC hitchhiking and in vivo targeting. The work is innovative, systematically executed, and major conclusions are supported by extensive in vitro and in vivo data. The data are promising and the findings are exciting, I'm willing to support its publication in Nature Communications after a few clarifications and additional experiments could be made.
1. The proposed hydrophobic block at the outer surface of nanoparticles is somehow unfavored from an energy point of view. Would these hydrophobic domains folded or aggregated inside in order to avoid solvent exposure? Some microscopy or simulation works would help to clarify this point.
2. What kind of interactions on the surface of RBC are responsible for the hitchhiking? Would protein corona of the NP play any role in this special lung-selective biodistribution? These are important questions to answer. Although I don't know whether it is feasible to the authors to find the answer, some attempts or discussions would be highly appreciated.
3. At the beginning of the post–Fig. 3 discussion, the transition from "β-branched amino acids perturb protein folding" to "β-branched residues alter copolymer sequence" is not obvious.
4. The copolymerization kinetics data provided (for Val and Nva NCAs) are informative but limited. To better support the claim that β-branching leads to gradient/blocky sequences, please include additional copolymerization kinetics curves for other NCAs (e.g., Leu NCA, Phe NCA) with Glu NCA. Alternatively, provide biodistribution data for other block copolymer nanoparticles (e.g., SiE-b-Leu, SiE-b-Phe) to demonstrate the generalizability of sequence-dependent targeting.
5. Please provide DP values for representative heteropolypeptides (e.g., PEV, PENV) calculated from 1H NMR after deprotection. If feasible, size exclusion chromatography (SEC) data would further strengthen the characterization.
6. Correct a small typo in Abstract, "heteropolyepeptide" should be "heteropolypeptide".
With these clarifications and modest additions, the manuscript will be suitable for publication.

Reviewer #3

(Remarks to the Author)
I co-reviewed this manuscript with one of the reviewers who provided the listed reports. This is part of the Nature Communications initiative to facilitate training in peer review and to provide appropriate recognition for Early Career Researchers who co-review manuscripts.

Reviewer #4

(Remarks to the Author)
This is an interesting and innovative study in which the authors have designed and synthesized a series of sequence-regulated heteropolypeptide nanoparticles (NPs), successfully identifying a lead candidate (SiEV) with highly efficient lung-targeting capabilities. The authors propose that this superior lung targeting is achieved through in situ erythrocyte hitchhiking, a mechanism mediated by the unique surface chemistry of the NPs. This strategy achieves a remarkable lung accumulation of up to 32% of the injected dose and demonstrates significant therapeutic potential in an acute lung injury

(ALI) model. However, the manuscript could be further strengthened by addressing several points related to the logical flow of the argument, mechanistic elucidation, and experimental details.

Major Comments:
Regarding the narrative logic and screening strategy for the “erythrocyte hitchhiking” mechanism:
The central claim of sequence-dependent lung targeting via erythrocyte hitchhiking is compelling. However, the narrative connection between the initial screening and the proposed mechanism feels abrupt. The introduction provides a thorough background on engineering the physicochemical properties of nanocarriers for organ targeting but does not introduce or foreshadow the erythrocyte hitchhiking hypothesis. Consequently, the mechanism is presented as a post-facto explanation for the observed lung targeting of SiEV (identified from the macroscopic organ distribution screen in Fig. 2b), rather than a pre-established hypothesis driving the experimental design.
The authors might consider adjusting the narrative to create a stronger logical thread. For instance, the introduction could be revised to foreshadow the hypothesis that modulating polypeptide hydrophobicity to enhance interactions with blood cells, particularly erythrocytes, could be a viable strategy for lung targeting.
Furthermore, if in situ erythrocyte hitchhiking is the central proposed mechanism, a more direct screening strategy might have involved assessing the in vitro binding efficiency of the NP library to erythrocytes, rather than proceeding directly to an in vivo screen based on the macroscopic outcome of lung accumulation. The current approach cannot fully exclude the possibility that other mechanisms may contribute to the observed lung enrichment. A discussion of this point would strengthen the authors' conclusions.

Regarding off-Target effects and biocompatibility analysis:
The study convincingly demonstrates the high affinity of SiEV NPs for erythrocytes. However, as the NPs are administered systemically, their interaction with other circulating blood components is a critical aspect of their biological fate and potential off-target effects. The manuscript would be significantly strengthened by investigating the uptake or binding of SiEV NPs to other key blood cell populations, such as neutrophils, monocytes, and lymphocytes. Non-specific uptake by these immune cells could potentially trigger adverse immune responses or lead to clearance in non-target organs. Including this analysis would provide a more complete picture of the NPs’ in vivo specificity and safety profile.
Furthermore, a point of safety consideration is whether the high accumulation of SiEV NPs within the pulmonary capillaries could potentially impede local blood flow. Given the historical risk of microparticle embolization leading to adverse effects such as pulmonary hypertension, it would be beneficial for the authors to discuss this potential safety concern and, if possible, provide evidence to rule out such effects.

Minor Comments
Definition of "lung/liver ratio":
This ratio is used as a key metric for targeting efficiency. As there are several ways to calculate this ratio in the literature (e.g., based on the total radiant efficiency of the entire organ vs. the average radiant efficiency per unit mass/area), the authors should explicitly define their calculation method in the Methods section or figure captions for clarity and reproducibility.

Missing information in Fig. 2d caption:
The caption for Fig. 2d, which presents flow cytometry data on NP uptake by different lung cell types, does not specify the time point at which the lung tissue was harvested post-injection. Please add this crucial information to the caption.

Use of "quantitative analysis" for IVIS data and unit clarification:
The term "quantitative analysis" is used in relation to IVIS imaging data (e.g., Fig. 2g caption). It is generally accepted that in vivo fluorescence imaging provides semi-quantitative data due to factors like signal attenuation in tissue. It is recommended to revise this wording to "semi-quantitative analysis" throughout the manuscript for greater scientific accuracy.
Specifically, the y-axis in Fig. 2g is labeled in units of "μg/mg tissue". The direct output from an IVIS system is typically radiant efficiency. The Methods section should be updated to clearly describe how this fluorescence signal was converted into a mass-based concentration unit (μg/mg), which presumably required the use of a standard curve correlating fluorescence intensity to known NP concentrations.

Discussion of in vivo NP-erythrocyte complex stability:
The study provides excellent in vitro data on the NP-erythrocyte interaction. To further substantiate the in situ hitchhiking mechanism, it would be beneficial to include a discussion on the stability of the NP-erythrocyte complexes in circulation over time in vivo. For how long do the NPs remain attached to the erythrocytes before reaching the lung capillaries?

Shear stress simulation:
In Fig. 3j, shear stress is simulated by manual extrusion through a syringe to demonstrate shear-induced release. While this provides proof-of-concept, it would be helpful if the authors could comment on how this model relates to the physiological shear forces within pulmonary capillaries and acknowledge any potential limitations.

Long-term clearance of nanoparticles:
The biodistribution study impressively tracks the NPs for up to 120 hours. A brief discussion in the manuscript regarding the expected long-term clearance pathway and degradation of these silica-based NPs from the lung tissue would be a valuable addition for a comprehensive toxicological and pharmacokinetic profile.

Version 1:

Reviewer comments:

Reviewer #1

(Remarks to the Author)
This reviewer appreciates considerable writing efforts invested by the authors in their attempt to tangentially obviate or mute points made by this reviewer in the first round. I have clearly indicated that the ultimate test of whether uptake in the lungs is roughly the same after IV vs alternative injection routes, or non-IV routings direct injected materials to other organs at cost of inhibiting the lung uptake. The first outcome may be interpreted as an indirect evidence consistent with (although not finally scientifically proving) the preferable uptake in the lungs (NOT TARGETING!). The second outcome establishes that authors deal with totally non-specific retention of the particles in the first significant microvascular networks downside the injection. The mechanism of this trivial outcome might combine mechanical and electrostatic retention of the injected particles or result of their fast enlargement post-injection due to deposition of blood components, or by aggregation of particles, or other processes. This study, therefore, unfortunately unlikely yield substantial and compelling justification for continuing efforts. This reviewer noted that most of requested methodologies, first of all, isotope tracing of RBC and NP labeled by different isotopes and quality of other approaches employed in this study. While these methodological aspects would be very important to discuss in the scenario of lung-preferred uptake after various routings, failure of the routing study provides a strong justification for termination of this publishing affair.

Reviewer #2

(Remarks to the Author)
All of my previous questions have been adequately addressed, and I am satisfied with the revisions made. I have no further concerns and recommend the manuscript for publication in its current form.

Reviewer #3

(Remarks to the Author)
I co-reviewed this manuscript with one of the reviewers who provided the listed reports. This is part of the Nature Communications initiative to facilitate training in peer review and to provide appropriate recognition for Early Career Researchers who co-review manuscripts.

Reviewer #4

(Remarks to the Author)
The authors have answered all my questions. Given the novelty and quality of this work, I support its publication in *Nature Communications*. I have no further comments.

Version 2:

Reviewer comments:

Reviewer #1

(Remarks to the Author)
I am glad that authors addressed many of my previous requests. These changes in general greatly elevated the quality and potential impact of their study. In particular, the tracing of isotope-labeled particles injected via IC vs IV routes is very important. This result indicates that particles injected IV accumulate in the lungs whereas those injected IC accumulate in other organs at the cost of dramatic reduction of the pulmonary uptake. Ergo, these particles do not target specifically any organ or, more precisely, any vascular area. Similar outcome has been described in multiple papers of radiolabeled imaging probes. Occam Razor logics dictates that the most simple and direct explanation of this outcome would likely include retention/occlusion/entrapment/uptake of the particles due to their charge, size and rapid aggregation in blood.

This result was expected. Numerous papers from Langer's/Anderson team have reported similar results. The authors must not be disappointed, because as they state astutely, most likely they have discovered an important and novel phenomenon. They have a good chance to decode physicochemical parameters of their novel tentative drug delivery system including the role of hemodynamics, functional and morphological status of endothelium, glycocalyx, blood and its clotting and other pathological and physiological characteristics.

However, it is categorically unadvisable to call this phenomenon in the way offered in the manuscript. Authors must abandon terms distorting and misrepresenting the nature of their discovery peppering the manuscript by flashy words such as “targeting”, “specific” “elucidation of targeting mechanism” and alike. Using these “terminological bragging points” has no scientific basis and emanates an aroma of self-promotion. It is unfair to criticize antibody-mediated targeting for delicate nature and high price tag while hailing your own preliminary findings by reciting tentative but not proven advantages, some of which most likely will not bear the fruit. The field of pulmonary vascular targeting yielded scores of reproducible, controlled and scientifically sound studies. The literature on PECAM targeting unequivocally proves this point. In fact, authors support this statement by including PECAM targeted NP studies in their work.

This is difficult to explain, though, why authors use as the reference to PECAM-directed targeting ref.39 that deals with totally different aspects of immunological signaling instead of the pubs that address the question of interest directly and beyond any doubts [PMID 10051650; 12851209; 12652312,]. This reviewer made a specific comment in the previous iteration of the manuscript, which authors preferred to ignore, and which I request to address directly and using appropriate literature: “There are issues with literature. The second sentence in the abstract says: “the rational design of lung-targeted nanocarriers through fine-tuning of molecular structures remains challenging’. This reviewer prefers to take this as a human error rather than either intentional misleading attempt to self-inflate, or lack of relevant knowledge. Lots of studies established specific targeted delivery to the pulmonary endothelium of a plethora of cargoes, carriers and conjugates using ligands of PECAM, ACE, thrombomodulin, caveolar determinants, etc., published in PNAS, Nature Series, ACS Nano, Blood, etc.. Based on the literature and direct isotope tracing nanoparticles, the pulmonary endothelium is very precisely served by particles binding to ACE, TM and PV1, whereas PECAM ligands work as pan-endothelial binding sites. Even cargo for these truly pulmonary targeting studies have been similar to the current study, including quencher of superoxide and hydrogen peroxide. Authors need to compare their approach and results obtained with previous studies focused on specific targeting of agents to the pulmonary endothelium. What exactly is better in the submitted manuscript in terms of targeting or protective effects of targeted delivery to EC of SOD and catalase in animal models of ALI, pulmonary embolism, radiation injury, inflammation, edema – all of which were responding to these targeted therapeutics with clear and compelling improvement?

In this context, the conclusion section offered in the current version looks as an attempt to sell data presented in the manuscript, despite the fact that the results are preliminary and incomplete. This desire to embellish the impact is problematic in biomedical aspects. For example, the NP rigidity may be cause for vascular abnormalities in the microvasculature in the lungs. The morphology of the RBC/NP complexes in figure 3d looks like upper panel RBC is coated by nearly the monolayer of small particles. Is this impression correct? If so, this reviewer believes that this complex will be problematic.
Further emphasizing this point: Fig.S4 shows patchy deposits of fluorescent materials in the lung sections which looks exactly as microemboli, size of particles in suppl fig.17 does not fit with size bar, PDI is not systematically shown and size of panels and axes make them illegible.

The hypothetical scenario how this delivery system may work is hanging in the air. Measuring plasma effects on NP must be compared with whole blood, under static conditions and hydrodynamic stress in terms of agglutination, stability, damage and dissociation. How fast does NP bind to RBC? How many particles added and bound per RBC? How the kinetics of attachment and detachment look like? All these questions, that can be at least initially addressed in vitro, are vitally relevant to the real attempts to figure out what does take place in vivo, namely how fast and where NP stick to and detach from RBCs after injection. The glimpses of kinetic data provided in the current version, to a great surprise imply that NP loading/detachment to/from RBCs takes a relatively long time, namely hours. Where does this happen? No convincing data prove the transfer in the lungs or any other organ. Authors repetitively state that NP detachment in the pulmonary capillaries is caused by high shear stress. However, a pulmonologist would not agree, because blood flow in the pulmonary vasculature is the slowest and least shear stressed in the body. Peripheral, splenic, cerebral and especially myocardial vasculature represent sites of much higher shear stress.

From the previous review: The daunting challenges of interpretation optical signal is clearly illustrated by problem of blood: RBC have so strong signal of Hb that it dwarfs or distorts any optical probe. Only radioisotopes afford the needed quality of objective, precise and accurate data. To study hitchhiking you must have RBC labeled by 51Cr and cargo NP labeled by 125Iodine, 111In or other appropriate isotope that can de clearly and accurately discerned from RBC label. Only dual-labeling approach, described in RBC-HH studies allows to distinguish the transfer from RBC to the vascular cells from mechanical, charge or other non-specific RETENTION or UPTAKE of the whole RBC-cargo complex in the microvasculature downstream injection site. This is especially important in the RBC-HH studies, because this mechanism of delivery is prone to these non-specific features. Authors mention in passing that they failed to carry experiments using dual isotope labeled RBC/NP. This is too bad, because optical probes fail the authors to prove their hypotheses of local in vivo hitchhiking on RBC, followed by swing from RBC to endothelium. The third sentence in second paragraph on page R5 is astonishingly revealing: In response to the comment that supernatant in figure 4 looks reddish while control is yellow, authors state that this optical artefact “most likely originated from the color distortion when we took photo” (Sic!).

From the previous review: “Interaction with RBC must include effects of NP on RBC in vitro and in vivo (hemolysis by complement and osmotic stress, agglutination, persistence in the circulation and uptake by spleen and other organs). Have you measured Hb in the medium after filtering RBC-NP? F5 shows full lysis with water – please show the calibration curve of Hb A and describe the incubation conditions”. The super of control RBCs in Supplement Fig. 4 look reddish; why it looks blue in the third tube? Authors responded to this by admitting in passing that these are likely artefacts of color distortion inflicted by photography, which makes the whole issue of inadequacy of optical methods vs precision of isotope detection even more important.

In response, authors showed nearly ideally linear calibration for optical density of Hb measured in a buffer, which is appropriate basal level of this part of the study. Now you must reproduce this calibration using serial dilutions of Hb in plasma, whole blood and in the homogenates of organs after IV injection of NP and compare with the graph of Hb dilutions of Hb doses added directly to these biological specimen.

The comments cited below have not been addressed fully and adequately. This reviewer invites authors and editors to revisit these points if the main issues are solved. .

The nanoparticles devised in this study are very interesting. If (it is an important "if") the phenomenon and its specificity will be proven as requested, the question of what the tentative mechanism of the reported "specific precise delivery into the pulmonary endothelium is needed. Which component of nanoparticles recognize specifically and precisely pulmonary endothelium? In which vascular segment? Which surface marker on endothelium represents the binding site for these NP provides the precision and selectivity of the delivery?

A singular interpretation of the interesting results pertaining to the functional effects as the result of specific and precise delivery of the described particles to the pulmonary endothelium is NOT established. The observed results may represent the effects of interactions of the injected materials with numerous migratory and resident pulmonary constituencies.

Authors embarked on the testing protective effects of this drug delivery system in animal models of human lung diseases. Scientifically speaking, this direction of the continuation of this research project needs to answer the questions above first. This is especially relevant to the studies of such sort based on measuring totally non-specific markers of non-specific pathological conditions such as oxidative burst driven and manifested by ROS, MDA, etc.

Particles may engage diverse cells in blood and organs, including marginated leukocytes in the pulmonary vasculature, RES and other sites. It became known recently that interaction of particles with leukocytes and other reactive components of host defense may lead to unexpected consequences, such as hitchhiking particles by blood cells headed to the CNS.

Version 3:

Reviewer comments:

Reviewer #1

(Remarks to the Author)
In the rebuttal, authors admit that PMID 40668829 presents a highly promising and innovative approach. Indeed, the level of the heart uptake of the cardiotropic LNP/RNA determined quantitatively using isotopes and optical probes dwarfs known attempts of systemic (IV) delivery to the CMC in the myocardium. Further, this approach yields measuring blood level and distribution between blood plasma and cellular elements. PK and biodistribution data are missing in most publications including present paper. Instead of appraisal of the informative value of methodologies, authors seem concerned that "Incorporating this system would introduce a confounding variable". This reviewer categorically disagrees. Authors should discuss and cite PMID 40668829, missing in the manuscript. In the rebuttal it is mistakenly attributed to Kenjo. The glitch reveals inconsistencies in the way of engagement of "careful considerations".

Reviewer #5

(Remarks to the Author)
The authors of this manuscript have addressed a number of the comments made by Reviewer #1, and their data is consistent with lung delivery via in situ RBC hitchhiking. I agree with the reviewer that this phenomenon is to be expected based on prior published work, but see the value in the description of a new chemical approach to achieve this and agree with the prior reviewer that the real contribution of this work lies in understanding the NP-RBC interactions and how these influence the in vivo performance of the materials.

1. The authors assert (correctly) that in situ RBC hitchhiking offers advantages over antibody-mediated RBC (or pulmonary) targeted NPs and pre-incubated RBC-hitchhikers. The authors currently do not contextualize this work within the state-of-the-art of chemical-based (particularly ionic liquid-based) in situ RBC hitchhiking. Therefore, the most exciting claim of this manuscript is not the discovery of a chemical-based in situ RBC hitchhiker but the more delicate structural findings.
2. I am not convinced that the mechanism is as straight-forward as hydrophobic interactions. I do think it's likely that hydrophobic interactions play a role, but based on the structural specificity of the author's findings, I wonder if other transporters within the RBC membrane are also implicated (like LAT1).
3. I am curious about the decision to evaluate the RBC hitchhiking in vitro with isolated RBCs vs whole blood. Do the authors in vitro data better match the in vivo data when the NPs are first evaluated with whole blood instead of RBCs? This to me seems more rigorous than pre-incubating with plasma since it captures the competing dynamics. What percentage of NPs are ending up on the RBCs? (vs the number of RBCs with NPs on them) Is this consistent with the ~37% lung delivery they see?
4. I am convinced by the isotope experiments that the NPs are leaving the RBCs at the lungs, but the slow adsorption and desorption kinetics is inconsistent and confusing with this phenomenon and is still unresolved. The SEM image of the RBC is also inconsistent with this, and I'm not sure whether I'm looking at a red blood cell that is post-hitchhiking or not. Could the authors use elemental mapping to establish if the features on the RBCs are NPs? An additional 50 nm scale bar in the zoomed in images would also help with this.
5. I would recommend couching the biocompatibility experiments as early stage and preliminary. This is the first report of the material, so I do think it is unnecessary to include long term, repeat dose safety studies, but these results should be presented as they are - a first run, single dose study that indicates promising potential biocompatibility.

Open Access This Peer Review File is licensed under a Creative Commons Attribution 4.0 International License, which permits use, sharing, adaptation, distribution and reproduction in any medium or format, as long as you give appropriate credit to the original author(s) and the source, provide a link to the Creative Commons license, and indicate if changes were made.
In cases where reviewers are anonymous, credit should be given to 'Anonymous Referee' and the source.
The images or other third party material in this Peer Review File are included in the article's Creative Commons license, unless indicated otherwise in a credit line to the material. If material is not included in the article's Creative Commons license and your intended use is not permitted by statutory regulation or exceeds the permitted use, you will need to obtain permission directly from the copyright holder.
To view a copy of this license, visit https://creativecommons.org/licenses/by/4.0/

RESPONSES TO REFEREES
(Reviewer's comments in black, our response in blue, and text added to the paper in magenta)

Reviewer #1 (Remarks to the Author):

This is an interesting study that deserves constructive analysis and collegial encouragement. I hope that authors will take my comments in this context. There are several methodological aspects that MUST be addressed before the manuscript may be reconsidered for publication.

We thank the Reviewer for the positive comments and appreciate all the detailed and insightful comments and suggestions from the Reviewer. We have conducted additional experiments and revised the manuscript accordingly, especially on the molecular mechanism of the lung-targeting and the safety evaluation of the NPs, which certainly strengthened our arguments and improved the understanding of the biofate of the system.

The main issues on hand are the following.

1. The biomedical aspects of this study need more attention and work. The authors propose a new type of lung-targeted NP formulation, which has not been studied like liposomes or LNPs. This is the first publication on this NP (unless the authors insist otherwise), nothing is known about their fate and activities in the body. This question begs the detailed characterization of the fate of NP after injection, performed using adequate means (see below). The more inclusive molecular and cellular schemas of targeting and effect are needed. What the side effect of accumulation of silica NP in the lungs is? Memento Asbestosis?

We appreciate the detailed and helpful suggestions from the Reviewer. We concur with the Reviewer that the comprehensive assessment of the biofate, including biodistribution, clearance mechanisms, and long-term safety of lung-targeted NPs, is crucial for their biomedical application. While we have detailed answers to the specific questions from the Reviewer as shown below (especially Questions 3, 4, 6, and 7), we would like to briefly summarize our understanding on the biofate of the NPs: (1) both the formation of erythrocyte-SiEV complexes and the shear stress-facilitated unloading are critical for the pulmonary accumulation of the NPs; (2) Unlike antibody-based targeting that relies on specific markers/receptors in the target tissue/cell, the targeting of our NPs is mainly based on the different shear stress applied in capillary beds and normal blood vessels; (3) The administration route is actually quite important, as the pulmonary capillary is the first downstream capillary bed when NPs are *i.v.* injected; (4) RBCs serve as only carriers, which are neither entrapped nor internalized in pulmonary tissues, but unloading the NPs when they squeeze through the capillaries.

On the other hand, the pathogenicity of non-biodegradable, biopersistent, and fibrous asbestos stems from its pulmonary accumulation and the difficulty for clearance, leading to chronic inflammation and fibrosis. In contrast, the silica NPs have been widely used as biomaterials with no or negligible biocompatibility issues, including those used in pulmonary delivery (e.g., *Sci. Adv.*, **2024**, *10,* eabd7904 and *J. Control. Release*, **2025**, *385*, 114020). In addition to the hemolytic experiments in our initial submission (i.e., NPs did not induce RBC rupture), we have performed additional experiments to directly assess the safety of NPs. Hematoxylin-eosin

(H&E) staining and Masson tricolor staining on the major organs, including lungs, revealed that the use of SiEV did not cause significant inflammatory infiltration, tissue damage, or abnormal collagen deposition. Meanwhile, the NPs were gradually cleared from the lungs, with 0.76% remaining on 7 d post *i.v.* injection, in sharp contrast to the retention of asbestos.

The new staining results of major organs were added as Supplementary Fig. 26 (Page S48), which demonstrated the excellent short-term safety profile of our NPs. A dedicated section discussing biocompatibility and safety has been incorporated into the revised manuscript (Page 9 and Page 23, highlighted):

> "Finally, the Si signal gradually decreased and became nearly undetectable on 7 d after administration, suggesting the gradual clearance of the NPs from the body without long-term safety concerns (Supplementary Fig. 6)."
>
> "To evaluate the systemic toxicity of SiEV, healthy mice were *i.v.* injected with a single dose and sacrificed. As shown in Supplementary Fig. 26, representative H&E-stained sections of major organs from SiEV-treated mice showed no signs of necrosis, inflammation, or edema compared to the control group. Additionally, given the pulmonary targeting propensity of the SiEV, we specifically investigated the potential for lung fibrosis using Masson's trichrome staining (Supplementary Fig. 26). The results revealed no abnormal collagen deposition in the SiEV-treated group, indicating no evidence of fibrotic lesions. These histopathological findings demonstrate the satisfactory biocompatibility of SiEV following *i.v.* injection."

2. Methods for characterization of the pharmacokinetics (PK) in blood and tissues based on using optical probes DOES NOT characterize the blood level, and uptake in the organs (including the lungs). Optical data cannot adequately prove the quantitative parameters. These data are distorted by uncontrolled rate of self-quenching, tissue transparency in given segments of the spectrum, tissue background in healthy vs pathological conditions. The daunting challenges of interpretation optical signal is clearly illustrated by problem of blood: RBC have so strong signal of Hb that it dwarfs or distorts any optical probe. Only radioisotopes afford the needed quality of objective, precise and accurate data.

We thank the Reviewer for the insightful comments. Fluorescence labeling was a widely used method for the preliminary visualization of tissue distribution during the early screening process to study the biodistribution of nanocarriers (for instance, *Nat. Mater.*, **2025**, *24*, 637-648 and *Nat. Commun.*, **2025**, *16*, 899). On the other hand, we fully acknowledge the limitations raised by the Reviewer, where the optical data was easily distorted by interference, quenching and numerous other reasons, especially for the *ex vivo* fluorescent imaging of the organs. In fact, the original fluorescent quantification results in our initial submission (i.e., Fig. 2g in our initially submitted manuscript) was not from live animal imaging, but rather based on the *fluorescent signals after tissue homogenization, filtration, and RBC lysis*, which was based on reliable standard curves and already minimized the interference of tissue background, RBCs, and other factors. Meanwhile, the targeting of PEV-based NPs was also validated when the inorganic core was changed to QDs, which offer superior photostability compared to the organic fluorophores on the polymer side chains. The strong pulmonary fluorescent signal confirmed the lung-targeting of QEV (i.e., Supplementary Fig. 10 in our initially submitted manuscript), ruling out the possibility that the targeting signal originated from the degraded

polymer chains from NPs.

Following the suggestions from the Reviewer, we have performed additional studies to quantify the PK and tissue accumulation of NPs. While the radioisotope experiments are widely accepted to quantify the NP distribution, the limited access to radioactive compounds and instruments makes it difficult for us to finish the experiments within the revision period. Instead, *we conducted independent and quantitative analysis using inductively coupled plasma optical emission spectroscopy (ICP-OES)*. The quantification of silicon content *via* ICP-OES provided a direct and interference-free approach to assess biodistribution in blood and the lung tissues. Interestingly, the measurements of pulmonary accumulation of SiEV at 12 h post injection, which showed the highest retention, were quite consistent between the tissue homogenization-fluorescent method and the ICP-OES method (*i.e.*, 32% ID vs 37% ID). The ICP-OES data thus serve as an objective and quantitative validation of the fluorescence findings, free from optical artifacts, which have significantly improved the rigor of our study.

We have revised the *Methods* section in maintext as well as Supplementary Information to clarify and highlight the quantification of fluorescent signals with tissue homogenization. New experiments with ICP-OES quantification were incorporated as Fig. 2e and Fig. 2h (Page 6). Additionally, the related arguments on the quantification of fluorescent signals and the further use of quantitative ICP-OES were modified (Page 8, highlighted):

> "Additionally, pharmacokinetic analysis by inductively coupled plasma-optical emission spectroscopy (ICP-OES) revealed that SiEV, unlike SiE that was cleared within 3 h, exhibited prolonged blood circulation up to 12 h (i.e., 0.11 μg/mL in plasma) (Fig. 2e). The extended circulation of SiEV was attributable to its capacity of *in situ* RBC hitchhiking following *i.v.* injection, which enabled effective NP delivery to the lung tissues."

> "Additionally, the pulmonary concentration of SiEV were first quantified through the homogenization of the lung tissues, which revealed highest Cy5 intensity at 12 h post injection, corresponding to 0.2 μg of SiEV per mg of the tissue (i.e., corresponding to 32% injected dose (ID) (Supplementary Fig. 6). Considering the possible optical artifacts with the fluorescent method, the Si content was further measured by ICP-OES (Fig. 2h), which revealed ~4.9 μg of Si in the entire lung tissue (i.e., 32.7 μg of Si per g of the tissue), which accounted for 37% ID."

3. Studies of in vivo injection of the particles described in this and other papers MUST include a simple control on selectivity of the uptake in the lungs, i.e., injection into an artery feeding perfusion of organs of interest. If the pulmonary uptake of your particles measured by isotope labels of particle and RBC remain exactly the same as after IV injection (by isotope tracing), congratulations: apparently your approach does afford some tissue-specific targeting to the lungs. This result will warrant investigations of the mechanism underlying such an unusual and important result.

We appreciate but respectfully disagree with the comments from the Reviewer on the lung-targeting mechanism of our NP system. Specifically, the pulmonary accumulation of RBC hitchhiking NPs was attributed to the small pulmonary capillaries, which forced the translocation of the surface-adsorbed NPs when RBCs passed through the narrow blood vessels.

The mechanism is thus completely different with the antibody-based targeting, which relies on specific interactions between targeting ligands and certain tissue-specific markers/receptors. Since pulmonary capillaries is the first downstream capillary bed after the entry of NPs through a vein, the *i.v.* injection led to lung-specific targeting of RBC hitchhiking NPs. In fact, the existing erythrocyte hitchhiking literatures already discussed the abovementioned mechanism, which was supported by the fact that intra-arterially injected NPs accumulated in the immediately downstream organs (*Nat. Commun.*, **2018**, *9*, 2684).

Following the suggestion from the Reviewer, we conducted additional biodistribution experiments, where SiEV was administered through intracardiac (*i.c.*) injection. Cy5 fluorescence was observed in several major organs like liver, lung, and kidney, in sharp contrast to the high pulmonary accumulation of SiEV through *i.v.* injection. The *i.c.* injected SiEV NPs directly flow through multiple capillary beds like hepatic capillaries and peritubular capillaries, leading to the translocation and accumulation of SiEV in these organs. Therefore, we believe the targeting of SiEV was not based on the selective binding or uptake by lung tissues.

The results of the new biodistribution studies through *i.c.* administration were added as Fig. 4f and Fig. 4g (Page 14). The following discussion was incorporated to highlight the crucial role of *i.v.* injection in mediating lung-targeting of SiEV NPs (Page 15, highlighted):

> "Both the formation of erythrocyte-SiEV complexes and the *i.v.* injection were critical for the lung-targeting of SiEV, as the pulmonary capillaries is the first capillary bed encountered by the complexes administered through veins, which mediated efficient translocation of NPs to ECs. Therefore, the change in injection route would likely alter the biodistribution of the NPs, especially when pulmonary capillaries is not the only downstream capillary bed. To verify our hypothesis, Cy5-labeled SiEV was administered through intracardiac (*i.c.*) injection, where multiple downstream capillaries exist. The Cy5 fluorescent signals were observed in several organs including lungs, livers, and kidneys (Fig. 4f, g), substantiating that the lung-targeting of SiEV through *i.v.* injection was not correlated to specific receptors."

4. Interaction with RBC must include effects of NP on RBC in vitro and in vivo (hemolysis by complement and osmotic stress, agglutination, persistence in the circulation and uptake by spleen and other organs). Have you measured Hb in the medium after filtering RBC-NP? The super of control RBCs in Supplement f4 look reddish; why it looks blue in the third tube? F5 shows full lysis with water – please show the calibration curve of Hb A and describe the incubation conditions. To study hitchhiking you must have RBC labeled by 51Cr and cargo NP labeled by 125Iodine, 111In or other appropriate isotope that can de clearly and accurately discerned from RBC label. Only this dual-labeling approach, described in RBC-HH studies allows to distinguish the transfer from RBC to the vascular cells from mechanical, charge or other non-specific RETENTION or UPTAKE of the whole RBC-cargo complex in the microvasculature downstream injection site. This is especially important in the RBC-HH studies, because this mechanism of delivery is prone to these non-specific features.

We appreciate the comments from the Reviewer about the RBC complexation and the biofate of RBC-SiEV complexes. First of all, we have conducted the hemagglutination assay besides the hemolysis experiments. The visualization of a tight dot of SiEV-treated, non-aggregated RBCs substantiated the hemocompatibility of the heteropolypeptide-decorated NPs.

Additionally, the RBC-SiEV complexes were prepared and dual-labeled as the Reviewer suggested, which revealed the persistence of RBCs in circulation after unloading SiEV NPs in pulmonary capillaries. These new experimental results, together with the hemolysis studies in our initial manuscript, suggested the excellent biocompatibility of heteropolypeptide-modified NPs with RBCs.

Specifically for the RBC-SiEV interactions (i.e., Supplementary Fig. 4 in the initially submitted manuscript), the control RBC actually looks yellowish, which is completely different with the osmotic imbalance-induced hemolysis. The reddish supernatant in the figure likely originated from the color distortion when we took the photograph. On the other hand, the blue color of the supernatant in SiENV tube resulted from the Cy5 fluorescent dye attached on the heteropolypeptide side chains. Due to the weak affinity between SiENV and RBCs, the centrifugation separated the NPs and the RBCs, leaving the Cy5-labeled SiENV suspended in the supernatant. Moreover, we have measured the Hb in the supernatant after the formation and filtering of the RBC-SiEV complexes to quantify the hemolysis, which showed negligible Hb release and RBC disruption compared to those treated with water (i.e., 1.3 and 36.1 mg/dL after treatment with SiEV and water, respectively). The Hb release study further supported that the formation of erythrocyte-SiEV complexes showed little disruption of RBCs.

Finally, we have conducted the dual-labeling experiments as the Reviewer suggested. Specifically, the RBCs and SiEVs were labeled with organic fluorescent dyes, FITC and Cy5, respectively, which were then used for the formation of complexes. Again, the radioisotope experiment suggested by the Reviewer was not feasible due to instrumental/reagent issues. Nevertheless, the cellular-level results were measured by flow cytometric experiments, which were well-accepted with few concerns on optical artifacts. The results suggested the maintained circulation of RBCs in plasma, with negligible fluorescent signals detected in pulmonary tissues. Conversely, the Cy5-labeled SiEV exhibited gradual accumulation in lungs, coinciding with the decrease of NP concentration in blood. Therefore, we have offered solid evidences that the lung-targeting of SiEV stemmed from the unloading of the NPs from the complexes in pulmonary capillaries, rather than the entrapment/internalization of the entire complexes. Combining with the abovementioned studies on hemocompatibility, the RBCs only served as carriers to deliver SiEV to lung tissues, which remained intact and circulating in our system.

The new hemocompatibility experiments, including hemagglutination assay and Hb release, were incorporated as Supplementary Fig 2c and 2d (Page S24) (the calibration curve of Hb was shown here as Fig. R1). Due to the change in paper flow, the initial Supplementary Fig. 4 was removed for clarification. Meanwhile, the dual-labeling experiments were added as Fig. 4d and 4e (Page 14). The underlying mechanism for the lung-targeting of SiEV was discussed in the third section of the *Results and Discussion* (Page 14-15, highlighted):

> "To further investigate the biofate of erythrocyte-SiEV, the complexes were pre-formed with FITC-labeled RBCs (referred to as ^{FITC}RBC) and Cy5-labeled SiEV, which were *i.v.* injected into the mice. The percentage of FITC-positive RBCs in plasma were maintained at a constant level within 12 h, with negligible FITC signals observed in the lung tissues that resembled that from mice receiving only ^{FITC}RBC injection (Fig. 4d, e). In stark contrast, the Cy5-positive cells exhibited a gradual decrease in plasma over time, coinciding with the increase in fluorescent signals in lung tissues (Fig. 4d, e), suggesting the translocation of SiEV NPs from the complexes

to pulmonary tissues. The result excluded the possibility that the erythrocyte-SiEV complexes were entirely trapped in lung tissues or internalized by ECs. In other words, the RBC cells served as carriers, which stayed in the blood flow to consistently deliver SiEV into lung tissues."

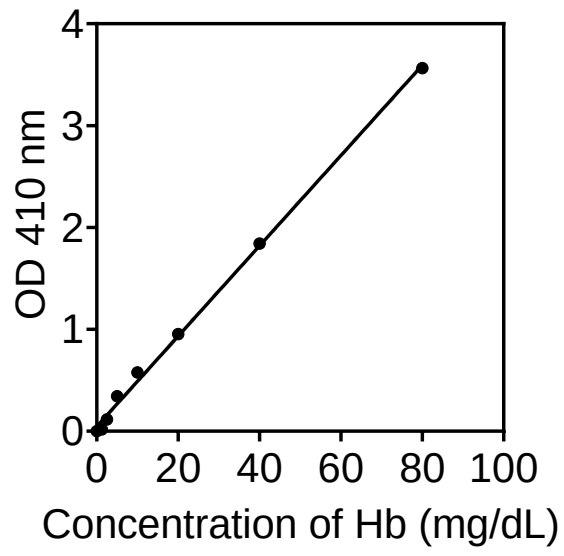

Fig. R1 The standard curve of Hb.

5. Particles may engage diverse cells in blood and organs, including marginated leukocytes in the pulmonary vasculature, RES and other sites. It became known recently that interaction of particles with leukocytes and other reactive components of host defense may lead to unexpected consequences, such as hitchhiking particles by blood cells headed to the CNS.

We appreciate the comments from the Reviewer. We performed additional experiments to explore the interactions between SiEV NPs and other blood cells. Flow cytometric result showed that the proportion of Cy5-positive RBCs was 28-fold higher than those with platelet and leukocytes, indicating that RBCs were the primary carriers of SiEV in circulation. The low uptake by leukocytes (less than 1%) also indicated the low incidence of unexpected consequences like triggering immune responses.

The results of adsorption/internalization of SiEV by other blood cells were incorporated as Supplementary Fig. 5 (Page S27). The following sentences were added to discuss the major binding with RBC and the minimized interactions with leukocytes (Page 7-8, highlighted):

"To verify that the lung-targeting of SiEV is mediated by *in situ* RBC hitchhiking, blood samples were collected for visualization at 1 h after *i.v.* injection of SiEV. As expected, the RBCs exhibited much stronger Cy5 fluorescence than other blood cells (Supplementary Fig. 5), confirming the *in vivo* complexation of SiEV with RBCs that mediated effective lung-targeting. On the other hand, the low uptake by leukocytes minimized the triggering of immune responses that validated the biocompatibility of SiEV."

6. The nanoparticles devised in this study are very interesting. If (it is an important "if") the phenomenon and its specificity will be proven as requested, the question of what the tentative mechanism of the reported "specific precise delivery into the pulmonary endothelium is needed. Which component of nanoparticles recognize specifically and precisely pulmonary endothelium? In which vascular segment? Which surface marker on endothelium represents the binding site for these NP provides the precision and selectivity of the delivery?

A singular interpretation of the interesting results pertaining to the functional effects as the

result of specific and precise delivery of the described particles to the pulmonary endothelium is NOT established. The observed results may represent the effects of interactions of the injected materials with numerous migratory and resident pulmonary constituencies.

Thank you for the comments on the lung-targeting mechanism. We respectfully disagree with the Reviewer's opinion that the targeting of SiEV originated from "specific and precise" recognition of certain receptors/components in pulmonary endothelium. We believe that the *in situ* RBC hitchhiking of SiEV to lung tissues requires *the hydrophobic interactions between erythrocytes and NPs to form complexes* as well as *sufficient shear forces in pulmonary capillaries to unload the NPs*. In fact, the lung-targeting was directly associated with the *i.v.* injection, where the pulmonary capillaries served as the first capillary bed when NPs were administered through a vein. The targeting mechanism is thus completely different with those specific delivery systems like antibody-decorated NPs. We mainly have three evidences to support our arguments:

(1) The lung-targeting SiEV and non-targeting SiENV exhibited similar internalization by iMPMVECs (i.e., mouse ECs). If the antibody-like targeting receptor/marker exists, one cannot expect to see such huge differences in the biodistribution of two NPs.

(2) As shown in our answers to Question 3 from the Reviewer, the *i.c.* injection of SiEV exhibited completely different biodistribution results compared to that through *i.v.* injection. Once the SiEV was administered through *i.c.* injection, it directly encountered multiple capillary beds in various organs like liver and kidney, leading to the accumulation of NPs in these organs. Similarly, previous literature reports also suggested the altered distribution of RBC-hitchhiking NPs to brains through intra-arterial injection (*Nat. Commun.*, **2018**, *9*, 2684).

(3) We have conducted additional experiment to check the internalization of SiEV and antibody-modified Si^{PECAM1} NPs by iMPMVECs pre-treated with anti-PECAM1, one of the most common pulmonary endothelial antibodies. As expected, the antibody pre-treatment blocked the uptake of Si^{PECAM1}, but showed little impact on SiEV internalization.

The uptake experiments of SiEV, SiENV, and Si^{PECAM1} by ECs and the antibody-blocking experiments were added as Supplementary Fig. 9 (Page S31) and Supplementary Fig. 13 (Page S35), respectively. We have incorporated additional discussion in the revised manuscript to clarify the lung-targeting mechanism (Page 10 and Page 15, highlighted):

> "At the same time, the internalization study by iMPMVECs revealed similar uptake of SiEV and SiENV (Supplementary Fig. 9), ruling out the possibility that the different lung-targeting of the two NPs originated from specific interactions with markers in lung tissues."
>
> "Both the formation of erythrocyte-SiEV complexes and the *i.v.* injection were critical for the lung-targeting of SiEV, as the pulmonary capillaries is the first capillary bed encountered by the complexes administered through veins, which mediated efficient translocation of NPs to ECs. Therefore, the change in injection route would likely alter the biodistribution of the NPs, especially when pulmonary capillaries is not the only downstream capillary bed. To verify our hypothesis, Cy5-labeled SiEV was administered through intracardiac (*i.c.*) injection, where multiple downstream capillaries exist. The Cy5 fluorescent signals were observed in several organs including lungs, livers, and kidneys (Fig. 4f, g), substantiating that the lung-targeting

of SiEV through *i.v.* injection was not correlated to specific receptors."

"As expected, the pre-treatment of iMPMVECs with anti-PECAM1 exhibited negligible impact on the internalization of SiEV, but significantly blocked the cellular entrance of Si^{PECAM1} (Supplementary Fig. 13). In summary, the *in situ* RBC hitchhiking of SiEV to lung tissues requires the hydrophobic interactions between erythrocytes and NPs to form complexes as well as sufficient shear forces in pulmonary capillaries to unload the NPs."

7. There are issues with literature. The second sentence in the abstract says: "the rational design of lung-targeted nanocarriers through fine-tuning of molecular structures remains challenging'. This reviewer prefers to take this as a human error rather than either intentional misleading attempt to self-inflate, or lack of relevant knowledge. Lots of studies established specific targeted delivery to the pulmonary endothelium of a plethora of cargoes, carriers and conjugates using ligands of PECAM, ACE, thrombomodulin, caveolar determinants, etc., published in PNAS, Nature Series, ACS Nano, Blood, etc. Based on the literature and direct isotope tracing nanoparticles, the pulmonary endothelium is very precisely served by particles binding to ACE, TM and PV1, whereas PECAM ligands work as pan-endothelial binding sites. Even cargo for these truly pulmonary targeting studies have been similar to the current study, including quencher of superoxide and hydrogen peroxide. Authors need to compare their approach and results obtained with previous studies focused on specific targeting of agents to the pulmonary endothelium. What exactly is better in the submitted manuscript in terms of targeting or protective effects of targeted delivery to EC of SOD and catalase in animal models of ALI, pulmonary embolism, radiation injury, inflammation, edema – all of which were responding to these targeted therapeutics with clear and compelling improvement?

We thank the Reviewer for the comments. As we already mentioned in the answers to Questions 3 and 6 from the Reviewer, the pulmonary targeting of SiEV was distinctly different from the antibody-decorated NPs. To be more specific, it is challenging to fine tune the molecular structures of *synthetic polymers* to achieve efficient lung-targeting, without the use of delicate targeting ligands like antibodies or aptamers. Therefore, we believe our targeting system is superior than the reported antibody-based systems in the following aspects:

(1) Scientifically, the SiEV system is the first reported example to achieve lung-targeting through *in situ erythrocyte hitchhiking*, rather than the pre-formation of complexes outside the body (e.g., *ACS Nano*, **2013**, *7*, 11129-11173 and *Sci. Adv.*, **2019**, *5*, eaax9250). Moreover, there are few synthetic polymer systems that the heteropolymer sequence makes a significant impact on their functions/properties.

(2) From an application aspect, the targeting of SiEV based on synthetic polypeptides *circumvents the use of delicate molecules* like antibodies, achieving comparable or even better targeting effect (see Supplementary Fig. 7 in the revised manuscript). With the recent advances in simplified preparation of polypeptide materials, the stability and synthetic scale of the polymer outperformed the natural proteins, offering an opportunity to lower the cost to prepare lung-targeting nanomedicines.

(3) The *in situ* complexation of SiEV with erythrocytes offers promising new designs of nanomedicine beyond lung-targeting. In fact, we are currently working on the development of

other RBC-associated NP designs in our lab.

The abstract was revised accordingly for clarification (Page 2, highlighted). The following sentence was added in the *Conclusion* section to briefly discuss the differences and advantages of our system compared to antibody-mediated designs (Page 23, highlighted):

> "Unlike the antibody-based targeting design, the *in situ* erythrocyte hitchhiking strategy is based on synthetic polypeptides that circumvents the use of delicate ligand molecules."

8. Authors embarked on the testing protective effects of this drug delivery system in animal models of human lung diseases. Scientifically speaking, this direction of the continuation of this research project needs to answer the questions above first. This is especially relevant to the studies of such sort based on measuring totally non-specific markers of non-specific pathological conditions such as oxidative burst driven and manifested by ROS, MDA, etc.

We appreciate the comments from the Reviewer on the future direction of the current work. The selection of ALI model is a fair demonstration to show the significantly enhanced therapeutic outcomes with potentially lower side effects of lung-targeting NPs, compared to their non-targeting analogues. We believe that the extension of the targeting NPs in biomedical applications and the in-depth understanding of the targeting mechanism are equally important. In fact, the ALI model is only one possible direction, and there are several other directions/questions requiring further exploration, including the fine-tuning of erythrocyte-NP interactions and other design based on RBC-NP complexes.

At the same time, while there are several animal models for lung-related diseases, the ALI model was selected with specific reasons: (1) The overwhelming distribution of SiEV in lung tissues, especially in endothelial cells, validate the treatment of ALI, which occurs primarily due to endothelial damage and uncontrolled inflammation. (2) The inorganic core of SiEV was easily changed to functional CeO_2 NPs for anti-inflammation effect. (3) Considering the acuteness of ALI disease, the *i.v.* injection was more feasible than other administrations like nebulization.

Reviewer #2 (Remarks to the Author):

The manuscript presents an interesting and potentially impactful strategy for lung endothelial enrichment that links heteropolypeptide sequence effects to RBC hitchhiking and in vivo targeting. The work is innovative, systematically executed, and major conclusions are supported by extensive in vitro and in vivo data. The data are promising and the findings are exciting, I'm willing to support its publication in Nature Communications after a few clarifications and additional experiments could be made.

We appreciate the positive comments from the Reviewer.

1. The proposed hydrophobic block at the outer surface of nanoparticles is somehow unfavored from an energy point of view. Would these hydrophobic domains folded or aggregated inside

in order to avoid solvent exposure? Some microscopy or simulation works would help to clarify this point.

Thank you for the comments. We concur with the Reviewer that the outer hydrophobic segments are unfavored when the NPs were dissolved in aqueous solution alone, which tended to fold within the NP or form inter-particle aggregations. Since the DLS results suggested negligible aggregation (normal TEM characterization is not suitable to reflect the NP status in solution), we believe that *the hydrophobic PVal segments may fold within the NP or became solubilized*, mainly depending on the hydrophobic chain length and NP concentration. Therefore, we have conducted ^{1}H NMR characterization to evaluate the solvation of the hydrophobic domain in D_2O. Compared to the NMR in TFA-*d* (i.e., assuming the heteropolypeptide chains are fully solvated), SiEV with 20% valine residues exhibited 17% decrease in the proton signals of valine side chains (δ = 0.95 ppm), suggesting the aggregation of the PVal segments to avoid solvent exposure. In contrast, the decrease in valine protons was negligible for SiEV with 10% valine residues, indicating the almost full solvation of the PVal segments with short chain length. Meanwhile, we are aware that the precise spatial arrangement of these hydrophobic domains is crucial to further elucidate the adsorption with RBCs and subsequent lung-targeting, which is currently under investigation and will be included in our future publication.

The NMR characterization was incorporated as Supplementary Fig. 10 (Page S32). The following sentence was included in the main text to discuss the solution status of hydrophobic segments (Page 12, highlighted):

> "While the existence of a hydrophobic outer layer of a NP in aqueous solution was disfavored, NMR study suggested rather good solvation of the hydrophobic segments of SiEV in D_2O at 10 mol% valine (Supplementary Fig. 10). Further increase in valine proportion to 20 mol%, nevertheless, led to the decrease in valine signals that suggested the assembly or folding of the hydrophobic segments to avoid excessive solvent exposure."

2. What kind of interactions on the surface of RBC are responsible for the hitchhiking? Would protein corona of the NP play any role in this special lung-selective biodistribution? These are important questions to answer. Although I don't know whether it is feasible to the authors to find the answer, some attempts or discussions would be highly appreciated.

We appreciate the insightful comments from the Reviewer. Our initial structure-property relationship studies suggested that the exposure of hydrophobic chains on the NP surface is critical for the interactions with RBCs. Therefore, we believe that the lipid bilayer of RBC plays an important role in mediating the interactions. In other words, the hydrophobic tails from SiEV insert into the bilayer in a similar way as the lipid tethers (e.g., *Nat. Commun.*, **2014**, *5*, 4482 and *Chem. Mater.*, **2025**, *37*, 5621-5635). To validate this hypothesis, we checked the interaction of SiEV with GUV, a synthetic analogue of RBC with only lipids. As expected, SiEV exhibited significantly stronger co-localization with the GUV membrane compared to SiENV, even in the absence of membrane proteins, indicating the important role of lipid bilayer in mediating the formation of erythrocyte-SiEV complexes.

For the impact of protein corona, we initially thought that the simultaneous formation of

erythrocyte-SiEV complexes and the adsorption of serum proteins were competing pathways upon the injection of NPs. To better answer the question from the Reviewer, we manually separate these two processes by first prepared a protein corona-coated SiEV ($SiEV^{PC}$) *via* plasma incubation. Interestingly, the *in vitro* RBC affinity assay revealed comparable binding capacity of $SiEV^{PC}$ and SiEV, suggesting that the protein corona does not impair the RBC hitchhiking of SiEV. Therefore, we would expect even less impact of protein corona on the lung-targeting, when protein adsorption and RBC complexation occur concurrently. Nevertheless, we believe that the formation of protein corona will somehow change the properties of SiEV or its RBC complexes. Protein adsorption is still a key process to consider for the *in situ* RBC hitchhiking design.

The new results on SiEV-GUV complexation and the impact of protein corona were incorporated as Fig. 3h and Fig. 3i (Page 11), respectively. The following sentences were included in the manuscript to briefly discuss the erythrocyte-SiEV complexes and the impact of protein corona (Page 12, highlighted):

> "Once injected into the blood vessels, the complexation of SiEV with RBC likely originated from the insertion of the hydrophobic segments into the bilayer of RBC membrane like lipid tethers. To further support our hypothesis, giant unilamellar vesicles (GUVs) were prepared, which served as a synthetic analogue of RBC with only lipids on the membrane. The pronounced co-localization of SiEV with the GUV membrane (Fig. 3h), in stark contrast to SiENV, provided visual evidence that the exposed hydrophobic segments drove the complexation with lipid membranes through hydrophobic interactions. Meanwhile, the exposed hydrophobic segments may also result in the adsorption of serum proteins. To check the impact of protein corona, SiEV was first pre-treated with plasma for the formation of protein corona, which was evidenced by the increase in size and change in zeta potential (Supplementary Fig. 11). Interestingly, the resulting protein-coated $SiEV^{PC}$, with the adsorption of 37 μg protein/mg NPs, exhibited similar RBC affinity compared to bare SiEV (Fig. 3i). Therefore, even with the pre-formed protein corona, SiEV still complexed with the erythrocytes in situ, suggesting robust lung-targeting driven by the hydrophobic interactions between hydrophobic segments and membrane bilayers."

3. At the beginning of the post-Fig. 3 discussion, the transition from "β-branched amino acids perturb protein folding" to "β-branched residues alter copolymer sequence" is not obvious.

We thank the Reviewer for the comments. We have revised the transition to discuss from a structural aspect, specifically the steric hinderance, to make a smooth transition to the polymerization kinetic studies.

4. The copolymerization kinetics data provided (for Val and Nva NCAs) are informative but limited. To better support the claim that β-branching leads to gradient/blocky sequences, please include additional copolymerization kinetics curves for other NCAs (e.g., Leu NCA, Phe NCA) with Glu NCA. Alternatively, provide biodistribution data for other block copolymer nanoparticles (e.g., SiE-*b*-Leu, SiE-*b*-Phe) to demonstrate the generalizability of sequence-dependent targeting.

We thank the Reviewer for the comments. Following the suggestion from the Reviewer, we examined the biodistribution of block copolypeptide-based NPs, Si(E-*b*-L) and Si(E-*b*-F), both exhibiting significantly higher accumulation in lungs compared to their analogue decorated with random heteropolypeptides. This result substantiated that the lung-targeting effect is a general phenomenon for heteropolypeptide-based NPs, as long as the hydrophobic segments were placed at the outer layer.

The new biodistribution results were added as Supplementary Fig. 12 (Page S34). The following discussion was incorporated in the revised manuscript to highlight the general strategy to design polypeptide-based lung-targeting NPs (Page 13, highlighted):

> "Meanwhile, the block copolymerization strategy was also used to boost the pulmonary enrichment of the analogues of SiEL and SiEF, referred to as Si(E-*b*-L) and Si(E-*b*-F) (Supplementary Fig. 12), demonstrating the general lung-targeting design of heteropolypeptide-modified NPs by introducing hydrophobic outer layers."

5. Please provide DP values for representative heteropolypeptides (e.g., PEV, PENV) calculated from ^{1}H NMR after deprotection. If feasible, size exclusion chromatography (SEC) data would further strengthen the characterization.

Thank you for the suggestion. With limited access of aqueous-phase GPC, we provided the organic-phase GPC results of the polypeptides before deprotection. Due to the solubility issue of PE^{tBu}, the benzyl derivative of poly(glutamic acid) was used instead. The $PE^{tBu}V$ analogue, poly(γ-benzyl-L-glutamate)-*co*-poly(valine), was cleaved from silica NPs through HF etching and subjected to GPC analysis. The results indicated the generation of monomodal GPC peak from the surface-initiated polymerization, with $M_n = 1.62 \times 10^5$ kDa and $Đ = 1.31$ (DP = 680).

6. Correct a small typo in Abstract, "heteropolyepeptide" should be "heteropolypeptide".

We appreciate the reviewer for the comment. The typo has been corrected, and the manuscript has been thoroughly checked to avoid similar mistakes.

With these clarifications and modest additions, the manuscript will be suitable for publication.

Thank you again for the insightful comments. The new experiments following the suggestions from the Reviewer, especially the solution status of SiEV and the impact of protein corona, significantly strengthen our manuscript.

Reviewer #3 (Remarks to the Author):

I co-reviewed this manuscript with one of the reviewers who provided the listed reports. This is part of the Nature Communications initiative to facilitate training in peer review and to provide appropriate recognition for Early Career Researchers who co-review manuscripts.

We appreciate the comments and suggestions from the Reviewer.

Reviewer #4 (Remarks to the Author):

This is an interesting and innovative study in which the authors have designed and synthesized a series of sequence-regulated heteropolypeptide nanoparticles (NPs), successfully identifying a lead candidate (SiEV) with highly efficient lung-targeting capabilities. The authors propose that this superior lung targeting is achieved through in situ erythrocyte hitchhiking, a mechanism mediated by the unique surface chemistry of the NPs. This strategy achieves a remarkable lung accumulation of up to 32% of the injected dose and demonstrates significant therapeutic potential in an acute lung injury (ALI) model.

We thank the Reviewer for the positive comments.

However, the manuscript could be further strengthened by addressing several points related to the logical flow of the argument, mechanistic elucidation, and experimental details.

We appreciate all the detailed and insightful comments and suggestions from the Reviewer. The suggestion on logic flow is really helpful, which indeed helps us to deliver our designs in a more smooth and logical manner. We also performed new experiments to address the concerns from the Reviewer on the targeting mechanism and the experimental details.

Major Comments:

1. Regarding the narrative logic and screening strategy for the “erythrocyte hitchhiking” mechanism:

The central claim of sequence-dependent lung targeting via erythrocyte hitchhiking is compelling. However, the narrative connection between the initial screening and the proposed mechanism feels abrupt. The introduction provides a thorough background on engineering the physicochemical properties of nanocarriers for organ targeting but does not introduce or foreshadow the erythrocyte hitchhiking hypothesis. Consequently, the mechanism is presented as a post-facto explanation for the observed lung targeting of SiEV (identified from the macroscopic organ distribution screen in Fig. 2b), rather than a pre-established hypothesis driving the experimental design.

The authors might consider adjusting the narrative to create a stronger logical thread. For instance, the introduction could be revised to foreshadow the hypothesis that modulating polypeptide hydrophobicity to enhance interactions with blood cells, particularly erythrocytes, could be a viable strategy for lung targeting.

Furthermore, if in situ erythrocyte hitchhiking is the central proposed mechanism, a more direct screening strategy might have involved assessing the in vitro binding efficiency of the NP library to erythrocytes, rather than proceeding directly to an in vivo screen based on the macroscopic outcome of lung accumulation. The current approach cannot fully exclude the possibility that other mechanisms may contribute to the observed lung enrichment. A discussion of this point would strengthen the authors' conclusions.

Thank you for the suggestions. We agree that introducing the “erythrocyte hitchhiking” hypothesis earlier would create a stronger narrative. The *Introduction* section was revised accordingly to first introduce the concept of RBC hitchhiking, followed by our hypothesis and

design of heteropolypeptide NPs for *in situ* RBC complexation and lung-targeting. Meanwhile, we followed the suggestion from the Reviewer to conduct the *in vitro* complexation of various NPs with RBCs, which indeed showed distinct differences in binding. SiEV and SiEI NPs with significant lung-targeting behaviors exhibited strong affinity with RBC *in vitro*, which was consistent with our erythrocyte hitchhiking hypothesis.

We believe the changes reframe our manuscript based on a clear hypothesis, significantly enhancing the logical coherence and impact of our work. The new *in vitro* adhesion assay data with RBCs were included in our revised manuscript as Fig. 2b (Page 6). The narrative logic was changed based on the suggestion of the Reviewer, with some representative key sentences shown below (Page 3 and Page 6-7, highlighted):

> "Among various lung-targeting strategies, the red blood cell (RBC) hitchhiking serves as a promising method. Considering the biocompatibility and prior clinical successes, RBCs are attractive intravascular carriers that overcome various *in vivo* barriers like mononuclear phagocyte clearance, exhibiting enhanced accumulation of adsorbed nanoparticles (NPs) in the lungs by more than 10-fold through intravenous (*i.v.*) injection. However, the isolation of RBCs from donor blood and the *ex vivo* handling of RBCs are inevitable in conventional RBC hitchhiking design, which significantly complicates the procedures and increases the risk of contamination. It is therefore of great interest to design NPs with stronger interactions with RBCs, capable of *in situ* RBC hitchhiking for efficient lung-targeting."

> "Therefore, we reasoned that the introduction of heteropolymers onto the NP surface might allow for the fine-tuning of RBC-NP interactions, as the diverse side-chain design of heteropolymers offers rich non-covalent interactions that mimics natural proteins."

> "Unlike the traditional RBC hitchhiking strategy, we developed a streamlined approach to achieve lung-targeting *via* the direct *i.v.* injection of NPs capable of *in situ* association with circulating RBCs, rather than pre-incubation of NPs and RBCs outside the body……As a critical first step, Cy5-labeled SiE and SiEX with similar sizes and surface charges but different heteropolypeptide compositions were screened to identify candidates with strong RBC affinity *in vitro*. While most SiEX NPs showed similar binding with RBCs compared to SiE after 1-h *in vitro* incubation with RBCs, RBCs treated with SiEV and SiEI exhibited markedly stronger fluorescence signals than others (Fig. 2b). The mean fluorescent intensity (MFI) of SiEV- and SiEI-treated RBCs was 43- and 27-fold higher than that of SiE, respectively (Fig. 2b), highlighting the critical role of specific polypeptide composition in mediating RBC affinity."

2. Regarding off-Target effects and biocompatibility analysis:

The study convincingly demonstrates the high affinity of SiEV NPs for erythrocytes. However, as the NPs are administered systemically, their interaction with other circulating blood components is a critical aspect of their biological fate and potential off-target effects. The manuscript would be significantly strengthened by investigating the uptake or binding of SiEV NPs to other key blood cell populations, such as neutrophils, monocytes, and lymphocytes. Non-specific uptake by these immune cells could potentially trigger adverse immune responses

or lead to clearance in non-target organs. Including this analysis would provide a more complete picture of the NPs' in vivo specificity and safety profile.

We appreciate the comments from the Reviewer. We conducted additional experiments to examine the interactions of SiEV with other circulating blood cells. Flow cytometric analysis revealed that the proportion of Cy5-positive RBCs was approximately 28-fold higher than those of platelet and leukocytes, underscoring that RBCs serve as the primary carriers of SiEV NPs in the bloodstream. Meanwhile, the markedly lower association of NPs with leukocytes avoided the triggering of adverse immune responses. Additionally, given the potential influence of protein adsorption, we sought to determine whether a pre-formed protein corona affects the association of SiEV with RBCs through *in vitro* incubation with plasma. Our findings revealed that the binding ability of $SiEV^{PC}$ to RBCs was comparable to that of SiEV, suggesting that the protein corona does not prevent the complexation between RBCs and SiEV.

The results of adsorption/internalization of SiEV by other blood cells and the impact of protein corona were incorporated as Supplementary Fig. 5 (Page S27) and Fig. 3i (Page 11), respectively. The relevant discussion was incorporated in the revised manuscript (Page 7-8 and Page 12, highlighted)

> "To verify that the lung-targeting of SiEV is mediated by *in situ* RBC hitchhiking, blood samples were collected for visualization at 1 h after *i.v.* injection of SiEV. As expected, the RBCs exhibited much stronger Cy5 fluorescence than other blood cells (Supplementary Fig. 5), confirming the *in vivo* complexation of SiEV with RBCs that mediated effective lung-targeting. On the other hand, the low uptake by leukocytes minimized the triggering of immune responses that validated the biocompatibility of SiEV."

> "Meanwhile, the exposed hydrophobic segments may also result in the adsorption of serum proteins. To check the impact of protein corona, SiEV was first pre-treated with plasma for the formation of protein corona, which was evidenced by the increase in size and change in zeta potential (Supplementary Fig. 11). Interestingly, the resulting protein-coated $SiEV^{PC}$, with the adsorption of 37 μg protein/mg NPs, exhibited similar RBC affinity compared to bare SiEV (Fig. 3i). Therefore, even with the pre-formed protein corona, SiEV still complexed with the erythrocytes *in situ*, suggesting robust lung-targeting driven by the hydrophobic interactions between hydrophobic segments and membrane bilayers."

3. Furthermore, a point of safety consideration is whether the high accumulation of SiEV NPs within the pulmonary capillaries could potentially impede local blood flow. Given the historical risk of microparticle embolization leading to adverse effects such as pulmonary hypertension, it would be beneficial for the authors to discuss this potential safety concern and, if possible, provide evidence to rule out such effects.

Thank you for the insightful comments. We have conducted additional experiments, which revealed that SiEV NPs demonstrated excellent biocompatibility and clearance profiles. Specifically, the staining of major organs (including lungs) with H&E and Masson tricolor staining showed no significant signs of inflammation, tissue damage, or pathological collagen deposition. Furthermore, pharmacokinetic studies suggested that the NPs were gradually

cleared from the lungs after extended time post *i.v.* injection, with only 0.76% of the injected dose on 7 d.

The new staining results of major organs were added as Supplementary Fig. 26 (Page S48), demonstrating the excellent short-term safety profile of our NPs. The corresponding discussion on biocompatibility and safety has been incorporated into the revised manuscript (Page 9 and Page 23, highlighted)

> "Finally, the Si signal gradually decreased and became nearly undetectable on 7 d after administration, suggesting the gradual clearance of the NPs from the body without long-term safety concerns (Supplementary Fig. 6)."

> "To evaluate the systemic toxicity of SiEV, healthy mice were *i.v.* injected with a single dose and sacrificed. As shown in Supplementary Fig. 26, representative H&E-stained sections of major organs from SiEV-treated mice showed no signs of necrosis, inflammation, or edema compared to the control group. Additionally, given the pulmonary targeting propensity of the SiEV, we specifically investigated the potential for lung fibrosis using Masson's trichrome staining (Supplementary Fig. 26). The results revealed no abnormal collagen deposition in the SiEV-treated group, indicating no evidence of fibrotic lesions. These histopathological findings demonstrate the satisfactory biocompatibility of SiEV following *i.v.* injection."

Minor Comments

4. Definition of "lung/liver ratio":

This ratio is used as a key metric for targeting efficiency. As there are several ways to calculate this ratio in the literature (e.g., based on the total radiant efficiency of the entire organ vs. the average radiant efficiency per unit mass/area), the authors should explicitly define their calculation method in the Methods section or figure captions for clarity and reproducibility.

We appreciate the comment from the Reviewer. In our initially submitted manuscript, the lung/liver ratio (except for Fig. 2c) was determined from a protocol involving tissue homogenization, filtration, and RBC lysis, followed by the detection of fluorescence signals. We would like to emphasize that this established protocol was specifically designed to minimize potential interference from tissue autofluorescence, hemoglobin from RBCs, and other factors, thereby ensuring the accuracy and reliability of the quantitative distribution data. On the other hand, Fig. 2c in our initial manuscript was based on the quantification of *ex vivo* imaging fluorescent signals (i.e., average radiant efficiency), which was only semi-quantitative that was removed in our revised manuscript. To further confirm the fluorescent results from tissue homogenization, we actually performed additional quantification with ICP-OES (i.e., the content of Si), which revealed similar results with the fluorescent method.

We have clearly indicated the calculation method for the ratio of lungs to liver in the *Methods* section in Supplementary Information (Page S10, highlighted).

5. Missing information in Fig. 2d caption:

The caption for Fig. 2d, which presents flow cytometry data on NP uptake by different lung

cell types, does not specify the time point at which the lung tissue was harvested post-injection. Please add this crucial information to the caption.

We thank the Reviewer for the comments. The caption for Fig. 2d (now designated as Fig. 2f in our revised manuscript) has been updated to specify that lung tissues were harvested at 4 h post-injection (Page 6, highlighted).

6. Use of "quantitative analysis" for IVIS data and unit clarification:

The term "quantitative analysis" is used in relation to IVIS imaging data (e.g., Fig. 2g caption). It is generally accepted that in vivo fluorescence imaging provides semi-quantitative data due to factors like signal attenuation in tissue. It is recommended to revise this wording to "semi-quantitative analysis" throughout the manuscript for greater scientific accuracy.

Specifically, the y-axis in Fig. 2g is labeled in units of "μg/mg tissue". The direct output from an IVIS system is typically radiant efficiency. The Methods section should be updated to clearly describe how this fluorescence signal was converted into a mass-based concentration unit (μg/mg), which presumably required the use of a standard curve correlating fluorescence intensity to known NP concentrations.

We appreciate the comments from the Reviewer. Indeed, fluorescence imaging using IVIS, while widely used to provide biodistribution data, is inherently semi-quantitative. The signal intensity is influenced by several factors, including tissue thickness, opacity, and intrinsic autofluorescence, which distort the absolute quantity of fluorescent probes. As we already indicated in our answer to Question 4 of the Reviewer, we employed a protocol of tissue homogenization followed by fluorescence measurement in a transparent lysate, which offered reliable results coupled with a standard curve, effectively eliminating the optical artifacts. Furthermore, we have performed ICP-OES to track Si content in our delivery system. The results were in good agreement with those from our tissue homogenization fluorescence assay.

All terms "quantitative analysis" regarding the IVIS imaging data were revised to "semi-quantitative analysis". The calculation method of the y-axis in Fig. 2g (now included as Supplementary Fig. 6 in our revised manuscript) has been updated in the *Methods* section in Supplementary Information (Page S10, highlighted).

7. Discussion of in vivo NP-erythrocyte complex stability:

The study provides excellent in vitro data on the NP-erythrocyte interaction. To further substantiate the in situ hitchhiking mechanism, it would be beneficial to include a discussion on the stability of the NP-erythrocyte complexes in circulation over time in vivo. For how long do the NPs remain attached to the erythrocytes before reaching the lung capillaries?

Thank you for the suggestion. To evaluate the stability of RBC-SiEV complexes under simulated physiological flow conditions, the mixture of SiEV and RBCs was subjected to continuous agitation on a shaker operating at 100 rpm (*Nat. Commun.*, **2019**, *10*, 5476). Samples were then collected at designated time points (1, 3, 6, and 12 h), followed by centrifugation to separate the RBC-SiEV complexes from the supernatant. The fluorescence intensity of Cy5 in the supernatant was measured to quantify unbound SiEV. The amount of unabsorbed SiEV in the supernatant was negligible (< 1%) after 12-h incubation, indicating

that the RBC-SiEV complexes exhibited remarkable stability under simulated circulation conditions.

Furthermore, we have performed dual-labeling experiments as suggested by the Reviewer 1. The fluorescent signal of Cy5-SiEV in the lungs exhibited a substantial increase, from an initial 27.9% at 1 h to a peak of 69.2% at 12 h post injection, indicating that the release of SiEV from complexes and their pulmonary accumulation is a gradual, time-dependent process. The release of SiEV appears to require appropriate shear forces, and the risk of off-target effects due to premature dissociation of the complexes was effectively minimized.

The stability of RBC-SiEV and the dual-labeling experiments were added as Fig. 4b and Fig. 4d, e (Page 14). The third section of the *Results and Discussion* was revised to report the in-depth evaluation of the biofate of the complexes (Page 13-15, highlighted):

> "To evaluate the impact of shear stress on the stability of complexes, the suspension of erythrocyte-SiEV was first placed on a shaker with gentle agitation (i.e., 100 rpm) to simulate the hemodynamic shear stress during normal circulation. As shown in Fig. 4b, the RBC-SiEV complexes were quite stable under circulation-mimetic conditions, with negligible desorbed SiEV in the supernatant (< 1%) after 12-h incubation."

> "To further investigate the biofate of erythrocyte-SiEV, the complexes were pre-formed with FITC-labeled RBCs (referred to as ^{FITC}RBC) and Cy5-labeled SiEV, which were *i.v.* injected into the mice. The percentage of FITC-positive RBCs in plasma were maintained at a constant level within 12 h, with negligible FITC signals observed in the lung tissues that resembled that from mice receiving only ^{FITC}RBC injection (Fig. 4d, e). In stark contrast, the Cy5-positive cells exhibited a gradual decrease in plasma over time, coinciding with the increase in fluorescent signals in lung tissues (Fig. 4d, e), suggesting the translocation of SiEV NPs from the complexes to pulmonary tissues. The result excluded the possibility that the erythrocyte-SiEV complexes were entirely trapped in lung tissues or internalized by ECs. In other words, the RBC cells served as carriers, which stayed in the blood flow to consistently deliver SiEV into lung tissues."

8. Shear stress simulation:

In Fig. 3j, shear stress is simulated by manual extrusion through a syringe to demonstrate shear-induced release. While this provides proof-of-concept, it would be helpful if the authors could comment on how this model relates to the physiological shear forces within pulmonary capillaries and acknowledge any potential limitations.

We appreciate the comment from the Reviewer. Our hypothesis is that a threshold of shear forces exists, which is sufficient to trigger the release of SiEV from the complexes. The shear stress in pulmonary capillaries is higher than that in normal blood vessels, circumventing the risk of premature dissociation of complexes. This hypothesis is supported by comparing our *in vitro* agitation experiments, which were conducted either under gentle shaking (100 rpm, *Nat. Commun.*, **2019**, *10*, 5476) or manual extrusion from syringes (*Nat. Biomed. Eng.*, **2021**, *5*, 1019-1037). Nevertheless, the precise quantification of the shear threshold warrants further systematic investigation and will be included in our future publications.

The relevant discussions about this experiment have been updated in the revised manuscript (Page 13, highlighted):

> "While the quantification of the shear forces requires further studies, the moderate interactions between SiEV and RBC guarantees the dissociation in capillaries while maintaining the complex stability under normal blood flow conditions. To evaluate the impact of shear stress on the stability of complexes, the suspension of erythrocyte-SiEV was first placed on a shaker with gentle agitation (i.e., 100 rpm) to simulate the hemodynamic shear stress during normal circulation. As shown in Fig. 4b, the RBC-SiEV complexes were quite stable under circulation-mimetic conditions, with negligible desorbed SiEV in the supernatant ($< 1\%$) after 12-h incubation. As a result, the off-target effect resulting from the premature release of SiEV from the complexes was minimized. In sharp contrast, a considerable amount of SiEV fell off from RBC when the complexes were loaded in a syringe and the shear stress was applied through manual extrusion. Flow cytometric analysis revealed significantly decreased Cy5 signals of the complexes with stronger shear stress that mimics the flow in pulmonary capillaries (Fig. 4c)."

9. Long-term clearance of nanoparticles:

The biodistribution study impressively tracks the NPs for up to 120 hours. A brief discussion in the manuscript regarding the expected long-term clearance pathway and degradation of these silica-based NPs from the lung tissue would be a valuable addition for a comprehensive toxicological and pharmacokinetic profile.

We appreciate the comment from the Reviewer. Previous literature showed that silica NPs were primarily cleared by the mononuclear phagocyte system, leading to significant accumulation in liver (*Nat. Commun.*, **2018**, *9*, 4551). Our data indicated that even after the clearance of NPs in plasma (i.e., at 12 h post injection), the fluorescent signal of SiEV is still detectable in the liver. This result suggested that a portion of the SiEV were ultimately metabolized and cleared from the body *via* the hepatobiliary route.

The *ex vivo* semi-quantitative analysis of liver after 24 h were incorporated as Supplementary Fig. 6c (Page S28). The related arguments on the metabolism and clearance of SiEV were added (Page 9, highlighted):

> "Finally, the Si signal gradually decreased and became nearly undetectable on 7 d after administration, suggesting the gradual clearance of the NPs from the body without long-term safety concerns (Supplementary Fig. 6)."

RESPONSES TO REFEREES

(Reviewer's comments in black, our response in blue, and text added to the paper in magenta)

Reviewer #1:

1. This reviewer appreciates considerable writing efforts invested by the authors in their attempt to tangentially obviate or mute points made by this reviewer in the first round.

We appreciate but respectfully disagree with the comments from the Reviewer. We believe that all comments by the Reviewer in the first round has been properly addressed, including the targeting mechanism, safety evaluation, and comparison with antibody-modified strategy (please check the table below). In fact, we feel that some of the comments, such as the dual-labeling experiments, are critical for us to elucidate the targeting mechanism and further strengthen our manuscript.

Purpose	Experiments	Position in revised manuscript in the second round
Safety evaluation	H&E and Masson tricolor staining<br>RBC agglutination and Hb release	Supplementary Fig. 29<br>Supplementary Figs. 2c and 2d
Confirmation of biodistribution and pharmacokinetics	Si quantification by ICP-OES	Figs. 2h and 2e
Comparison with antibody-targeting systems	*i.v.* injection of SiEV and Si^{PECAM1}	Supplementary Figs. 9, 13, and 16
Targeting mechanism	Intracardiac injection<br>Dual-labeling experiments	Figs. 4h and 4i<br>Supplementary Fig. 15
Specificity of RBC complexation	Blood cell adsorption	Supplementary Fig. 5

2. I have clearly indicated that the ultimate test of whether uptake in the lungs is roughly the same after IV vs alternative injection routes, or non-IV routings direct injected materials to other organs at cost of inhibiting the lung uptake. The first outcome may be interpreted as an indirect evidence consistent with (although not finally scientifically proving) the preferable uptake in the lungs (NOT TARGETING!). The second outcome establishes that authors deal with totally non-specific retention of the particles in the first significant microvascular networks downside the injection.

We appreciate the opinions and comments from the Reviewer. We are afraid it is oversimplified to summarize our targeting mechanism studies as “totally non-specific retention of particles”. The heteropolypeptide nanoparticles (NPs) were carefully designed and optimized, which relied on two critical processes to achieve effective lung-targeting: (1) the *in-situ* adsorption of NPs with hydrophobic corona onto erythrocytes; (2) desorption from RBC membrane in response to appropriate shear forces in lung tissues. *The materials design and mechanism study are novel compared with reported RBC hitchhiking system, which circumvents the in vitro handling of RBCs.*

Meanwhile, we believe our lung-targeting NPs exhibited obvious advantages over conventional antibody-like systems with “specific uptake and retention”. Compared with the fragile and expensive antibodies, the synthetic polypeptides are relatively stable and easy to prepare, but achieving similar or even better lung-targeting at least in our studies (lung-to-liver ratio up to 14). Additionally, *the dependence on administration route actually allows for more versatile designs of drug delivery beyond lung-targeting*, as already demonstrated in previous literatures (e.g., *Nat. Commun.*, **2018**, *9*, 2684). Therefore, we believe that the targeting mechanism of our NP is an advantage rather than a disadvantage compared to conventional antibody-based targeting systems.

The following discussion was incorporated to emphasize the advantages of our lung-targeting NPs over antibody modification (Page 24, highlighted):

> “Unlike the antibody-based targeting design that relied on the use of delicate and expensive ligand molecules, the *in situ* erythrocyte hitchhiking strategy is based on synthetic polypeptides with higher stability and simplified preparation strategy. Meanwhile, the reversible interactions with RBC membranes of SiEV provided other delivery designs beyond lung targeting.”

3. The mechanism of this trivial outcome might combine mechanical and electrostatic retention of the injected particles or result of their fast enlargement post-injection due to deposition of blood components, or by aggregation of particles, or other processes. This study, therefore, unfortunately unlikely yield substantial and compelling justification for continuing efforts.

Thank you for the comments. It is really unfortunate that the Reviewer regard our work as “trivial outcome”. RBC hitchhiking is a recently developed strategy with promising applications to treat pulmonary diseases or even beyond lung-targeted delivery (e.g., *Nat. Biomed. Eng.*, **2021**, *5*, 441-454, *Adv. Mater.* **2024**, *36*, 2310306, and *Sci. Adv.*, **2025**, *11*, 49). Compared with conventional RBC hitchhiking system, the reported heteropolypeptide NP in our manuscript, SiEV, exhibited *in situ* complexation with RBC that circumvented the *in vitro* handling of RBC, thus *not only simplifying the overall procedure but also offering new structural insights to inspire the future design of RBC hitchhiking methods*. In fact, the *in situ* complexation between RBCs and NPs was only recently reported by Mitragotri, Singh, et al. (after the initial submission of our manuscript), which was also published in high profile journal (*Nat. Commun.*, **2025**, *16*, 10280). Nevertheless, the targeting ability (lung-to-liver ratio of 2) was lower than our designed system (lung-to-liver ratio of 14). Additionally, from a material perspective, *our work represents one of the few examples to demonstrate the significant impact of copolymer sequence on their functions and properties*. It will therefore draw broad interests from scientists from the field of polymer chemistry, biomaterials, and nanomedicine.

Moreover, we have already provided solid evidence that the lung-targeting originated from the *in situ* adsorption on erythrocytes and release from erythrocytes in lung tissues in response to shear forces. Therefore, either the "mechanical and electrostatic retention" or the "fast enlargement post-injection" hypothesis by the Reviewer was incorrect. Specifically, (1) we have conducted additional experiments to monitor the stability of SiEV in plasma, which showed negligible aggregation or even significant changes in size. Meanwhile, the dual-labeling of RBC-SiEV complexes, no matter through fluorescent or radioisotope labeling (*vide infra*), suggested the desorption of SiEV from RBC in lung tissues. To further confirm the desorption process, the disassembly of RBC-SiEV complexes was also visualized by the fluorescent analysis of pulmonary sections. These results collectively ruled out the possibility of mechanical retention caused by the increase in size. (2) all our NPs were designed to have similar surface charges, excluding the possibility that electrostatic interactions are the main factor for pulmonary accumulation.

The results of the fluorescent analysis of tissues sections and the stability study of SiEV was added as Fig. 4d/4e and Supplementary Fig. 6, respectively (Page 14 and S29). The following discussion was incorporated to highlight RBC-mediated lung-targeting of SiEV NPs (Page 15 and 8, highlighted):

> "Notably, the excellent stability of SiEV in plasma ruled out any significant size changes (Supplementary Fig. 6), thereby excluding the possibility of passive lung-targeting through mechanical filtration."

> "To further investigate the biofate of erythrocyte-SiEV in lung tissue, the complexes were pre-formed *in vitro* with FITC-labeled RBCs (referred to as ^{FITC}RBC) and Cy5-labeled SiEV, which were intravenously injected into the mice. CLSM characterization revealed that the RBC-SiEV complexes were still observed with good co-localization (Pearson coefficient ~ 0.54) at 3 h after *i.v.* injection. However, negligible FITC signal of RBCs was detected in lung tissues with low fluorescent co-localization (Pearson coefficient ~ 0.13) at 12 h, suggesting the translocation of SiEV from the complexes to pulmonary tissues (Fig. 4d, e)."

4. This reviewer noted that most of requested methodologies, first of all, isotope tracing of RBC and NP labeled by different isotopes and quality of other approaches employed in this study. While these methodological aspects would be very important to discuss in the scenario of lung-preferred uptake after various routings, failure of the routing study provides a strong justification for termination of this publishing affair.

Thank you for the comments. While we are aware of the importance of radioisotope labeling in biodistribution studies, we have provided solid evidence in the first round of revision that the fluorescent analysis of tissue homogenates and the ICP-OES experiments also generate reliable and quantitative results with negligible interference from tissues/cells. In fact, these two methods have been used in various biodistribution studies (e.g., *Nat. Biomed. Eng.*, **2020**, 4, 1102-1116, *Nat. Commun.*, **2021**, 12, 3393, *Nat. Mater.*, **2025**, *24*, 637-648, and *Nat. Commun.*, **2025**, *16*, 899).

Meanwhile, we have conducted radioisotope labeling and tracing experiments in this round of revision, as suggested by the Reviewer. The results of time-dependent pulmonary retention studies were consistent with that from previous fluorescent or ICP-OES methods, suggesting

the peak accumulation at ~12 h with 26% ID pulmonary attention. In addition, the dual-labeling experiments also indicated the disassembly of RBC-SiEV complexes in lung tissues, with continuous circulation of RBCs that left the SiEV in pulmonary tissues. The latter dual-labeling results agreed well with the fluorescent labeling results in our first round of revision.

The results of the new radioisotope labeling and tracing studies were added as Fig. 4f/4g and Supplementary Fig. 8 (Page 14 and S31). Additionally, the related arguments of quantitative analysis of radioisotope labeling were modified (Page 8 and 15, highlighted):

> "Moreover, the pulmonary level of SiEV was also confirmed by fluorescent analysis of tissue homogenates or radioisotope tracing, which was consistent with the ICP-OES results that the pulmonary accumulation of SiEV reached a peak at 12 h post injection (Supplementary Fig. 7, 8)."

> "Meanwhile, different radioisotopes (i.e., ^{99m}Tc and ^{177}Lu) were used to separately label RBC and SiEV in the complex, which provided quantitative desorption results. While most ^{99m}Tc-labeled RBCs were constantly found in circulating plasma, ^{177}Lu-labeled SiEV gradually transferred from the blood to the lung tissues (Fig. 4f, g). Specifically, the blood level of SiEV decreased from 60.3% ID to 1.5% ID from 1 h to 12 h post injection. The pulmonary concentration of SiEV, on the other hand, increased from 2.8% ID to 25.3% ID."

Reviewer #2:

All of my previous questions have been adequately addressed, and I am satisfied with the revisions made. I have no further concerns and recommend the manuscript for publication in its current form.

We appreciate the insightful comments from the Reviewer. The suggestions helped us to further elucidate the targeting mechanism, and we are pleased with the positive final decision.

Reviewer #3:

I co-reviewed this manuscript with one of the reviewers who provided the listed reports. This is part of the Nature Communications initiative to facilitate training in peer review and to provide appropriate recognition for Early Career Researchers who co-review manuscripts.

Thank you for the valuable comments and suggestions. The comments indeed helped us to improve our arguments.

Reviewer #4:

The authors have answered all my questions. Given the novelty and quality of this work, I support its publication in *Nature Communications*. I have no further comments.

We thank the Reviewer for the constructive comments. The points raised by the Reviewer have helped us to further strengthened our manuscript, and we are pleased with the positive final assessment.

RESPONSES TO REFEREES

(Reviewer's comments in black, our response in blue, and text added to the paper in magenta)

Reviewer #1 (Remarks to the Author)

I am glad that authors addressed many of my previous requests. These changes in general greatly elevated the quality and potential impact of their study.

We thank the Reviewer for the positive comments on our revised manuscript. Your previous suggestions, especially the dual-labeling experiments to study the biofate of SiEV-erythrocyte complexes, have greatly improved our arguments and manuscript.

1. In particular, the tracing of isotope-labeled particles injected via IC vs IV routes is very important. This result indicates that particles injected IV accumulate in the lungs whereas those injected IC accumulate in other organs at the cost of dramatic reduction of the pulmonary uptake. Ergo, these particles do not target specifically any organ or, more precisely, any vascular area. Similar outcome has been described in multiple papers of radiolabeled imaging probes. Occam Razor logics dictates that the most simple and direct explanation of this outcome would likely include retention/occlusion/entrapment/uptake of the particles due to their charge, size and rapid aggregation in blood.

Thank you for the comments. The heteropolypeptide nanoparticles (NPs) developed in our manuscript indeed accumulate in the pulmonary tissues in a different manner with the active targeting molecules like antibodies, where the latter is dependent on interactions with certain acceptors in pulmonary tissues.

Nevertheless, we have to emphasize that *we have clear evidences that the lung-targeting of SiEV originated from the erythrocyte hitchhiking, which is distinctly different with the size-dependent mechanical filtration/entrapment* (e.g., *ACS Nano*, **2020**, *14*, 6693, *Acta Pharm. Sin. B*, **2025**, *15*, 2900, and *J. Am. Chem. Soc.*, **2026**, *148*, 12139). Briefly, (1) we have shown that SiEV were quite stable in plasma with negligible aggregations (Supplementary Fig. 6). (2) The dual-labeling experiments, together with the confocal images to visualize the desorption process, suggested that SiEV is in the form of erythrocyte complexes rather than aggregates right after injection (Figs. 4d-4g and Supplementary Fig. 16). (3) All heteropolypeptide NPs have similar surface charges and sizes, it is unlikely that only SiEV and SiEI showed pulmonary accumulation if the charge interactions or size aggregations make a significant contribution to the pulmonary targeting (Supplementary Fig. 1 and Supplementary Table 1).

All these evidences, which are included in our 2nd round of revision (response to Comment 3 from the same Reviewer), collectively supported the erythrocyte hitchhiking mechanism rather than physical entrapment due to specific charges, sizes, or aggregation behaviors.

2. This result was expected. Numerous papers from Langer's/Anderson team have reported similar results. The authors must not be disappointed, because as they state astutely, most likely they have discovered an important and novel phenomenon. They have a good chance to decode physicochemical parameters of their novel tentative drug delivery system including the role of hemodynamics, functional and morphological status of endothelium, glycocalyx, blood and its

clotting and other pathological and physiological characteristics. However, it is categorically unadvisable to call this phenomenon in the way offered in the manuscript. Authors must abandon terms distorting and misrepresenting the nature of their discovery peppering the manuscript by flashy words such as "targeting", "specific" "elucidation of targeting mechanism" and alike. Using these "terminological bragging points" has no scientific basis and emanates an aroma of self-promotion. It is unfair to criticize antibody-mediated targeting for delicate nature and high price tag while hailing your own preliminary findings by reciting tentative but not proven advantages, some of which most likely will not bear the fruit. The field of pulmonary vascular targeting yielded scores of reproducible, controlled and scientifically sound studies. The literature on PECAM targeting unequivocally proves this point. In fact, authors support this statement by including PECAM targeted NP studies in their work.

We appreciate but respectfully disagree with the comments from the Reviewer. In the field of nanomedicine or nano-delivery system, "targeting" is used to describe *(1) the "active targeting" through the modification of antibodies or other targeting ligands and (2) the "passive targeting" with the fine-tuning of physicochemical properties of NPs* (please check *Adv. Drug Delivery Rev.*, **2014**, *66*, 2, *Nat. Rev. Drug Discov.*, **2021**, *20*, 101, *Nat. Rev. Mater.*, **2023**, *8*, 282, and *Chem. Soc. Rev.*, **2023**, *52*, 7579). The pulmonary targeting of our heteropolypeptide NPs can be classified as "passive targeting", which indeed exhibited significant pulmonary accumulations. We therefore believe the term "targeting" is appropriate for our system. In fact, several previously reported systems on erythrocyte hitchhiking NPs are described as "lung-targeting" (e.g., *Nat. Commun.*, **2018**, *9*, 2684, *Adv. Mater.* **2024**, *36*, 2310306, and *Nat. Commun.*, **2025**, *16*, 10280). Meanwhile, *we do agree with the Reviewer that some terminologies like "specific" is not necessary*. These words are removed to ensure a more precise presentation.

On the other hand, we fully recognize the significant values and broad applications of antibody-based drugs in precision medicine. However, the success of antibody modification does not necessarily deny the development of alternative targeting strategies. The challenges related to delicate nature and high cost of antibodies are widely acknowledged in the field. Therefore, our purpose is *to develop additional options for the design of lung-targeting nanomedicine, rather than replacing the existing antibody strategy by criticizing and ignoring its success*. In fact, the comparison with antibody strategy was actually required by the same Reviewer in the 1st round of revision (Comment 7).

As requested by the Reviewer, we have carefully checked the manuscript to remove inaccurate word uses like "specific", and revised the description of antibody strategies accordingly (Pages 9 and 24, highlighted):

> "SiEV thus served as a promising alternative choice to antibody-decorated, lung-targeting NPs."
>
> "The *in situ* erythrocyte hitchhiking strategy is based on synthetic polypeptides with higher stability and simplified preparation strategy, serving as another option for antibody-based targeting strategies."

3. This is difficult to explain, though, why authors use as the reference to PECAM-directed targeting ref.39 that deals with totally different aspects of immunological signaling instead of the pubs that address the question of interest directly and beyond any doubts [PMID 10051650; 12851209; 12652312,]. This reviewer made a specific comment in the previous iteration of the

manuscript, which authors preferred to ignore, and which I request to address directly and using appropriate literature: "There are issues with literature. The second sentence in the abstract says: "the rational design of lung-targeted nanocarriers through fine-tuning of molecular structures remains challenging'. This reviewer prefers to take this as a human error rather than either intentional misleading attempt to self-inflate, or lack of relevant knowledge. Lots of studies established specific targeted delivery to the pulmonary endothelium of a plethora of cargoes, carriers and conjugates using ligands of PECAM, ACE, thrombomodulin, caveolar determinants, etc., published in PNAS, Nature Series, ACS Nano, Blood, etc. Based on the literature and direct isotope tracing nanoparticles, the pulmonary endothelium is very precisely served by particles binding to ACE, TM and PV1, whereas PECAM ligands work as pan-endothelial binding sites. Even cargo for these truly pulmonary targeting studies have been similar to the current study, including quencher of superoxide and hydrogen peroxide. Authors need to compare their approach and results obtained with previous studies focused on specific targeting of agents to the pulmonary endothelium. What exactly is better in the submitted manuscript in terms of targeting or protective effects of targeted delivery to EC of SOD and catalase in animal models of ALI, pulmonary embolism, radiation injury, inflammation, edema – all of which were responding to these targeted therapeutics with clear and compelling improvement?

Thank you for the suggestion. We agree with the Reviewer that the suggested references are better than our selection, which are directly related to the impact of PECAM-1 targeting on drug delivery. The reference citations have been updated accordingly (i.e., Refs. 39-41 in our revised manuscript).

We want to clarify that we did not "prefer to ignore" any comments or suggestions by the Reviewer. Specifically, for the question/comment mentioned here, we have explained that our synthetic polymer system is completely different with the antibody strategy (in our 1st round of revision, response to Comment 7 by the same Reviewer). Accordingly, we have already revised the second sentence in the abstract, performed new experiments to include the antibody-directed pulmonary targeting as a positive control (Supplementary Figs. 9, 11, and 17), and updated reference citations. We are puzzled whether we should make a more detailed comparison, given that the last comment from the Reviewer expressed concern about too much criticism of antibody-based strategies.

Finally, we would like to emphasize again that *we regard our work as "a unique example to show the significant impact on copolymer sequence on the biofate of polymers" and "a breakthrough in erythrocyte hitchhiking by circumventing the isolation of RBCs"*. Our background description and literature citations are thus mainly focused on prior work on structural modulation of polymeric/lipid-based nanocarriers and erythrocyte hitchhiking. Meanwhile, *we believe it is a complementary design of pulmonary-targeting nanomedicine, not the replacement of antibody-based strategy*. The vascular targeting by antibodies is thus discussed and cited but is not the major focus.

4. In this context, the conclusion section offered in the current version looks as an attempt to sell data presented in the manuscript, despite the fact that the results are preliminary and incomplete. This desire to embellish the impact is problematic in biomedical aspects. For example, the NP rigidity may be cause for vascular abnormalities in the microvasculature in the lungs. The morphology of the RBC/NP complexes in figure 3d looks like upper panel RBC

is coated by nearly the monolayer of small particles. Is this impression correct? If so, this reviewer believes that this complex will be problematic.

We appreciate the comments from the Reviewer. We respectfully disagree with the Reviewer that our results are "preliminary and incomplete". (1) We have strong supporting data to show the *lung accumulation* of heteropolypeptide NPs (up to 37% injected dose, highest lung-to-liver ratio ~ 14). (2) The mechanism studies suggested that the *sequence of the heteropolypeptide plays an important role* in mediating *in situ erythrocyte hitchhiking*, a more simplified method compared with conventional erythrocyte hitchhiking. (3) We also demonstrated *the use of the targeting ability for the treatment of ALI*, which showed significant advantages than non-targeting NPs. Considering it is only the first report of the system (we do have more projects on-going along this direction), we believe we have a complete study, with several details greatly improved after the suggestions and comments from the Editorial office and Reviewers.

Specifically, in our 1st round of revision, we have performed additional experiments such as H&E staining and Masson staining (Supplementary Fig. 31), both histopathological experiments showed that no obvious inflammatory infiltration, tissue damage, or fibrotic changes were observed in the lung tissues (response to Comment 1 from the same Reviewer). Furthermore, NPs with rigid inorganic cores have been used to treat pulmonary diseases without significant issues of "vascular abnormalities" (e.g., *Adv. Drug Delivery Rev.*, **2021**, *177*, 113953 and *Sci. Adv.,* **2024**, *10,* eabd7904).

The surface of RBCs from SEM images (Fig. 3d) indeed looks rough, which is distinct from the smooth surface with non-targeting, SiENV control NPs. Nevertheless, it is difficult to conclude from SEM images alone, whether it represents a monolayer of NPs or just the surface morphology change after NP binding. After the calculation of the number of average NPs adsorbed per RBC (~ 5,700, please check the details in the response to Comment 6 below), it is unlikely that the NPs formed a dense layer on the surface of RBCs. Meanwhile, the RBC agglutination and Hb release experiments confirmed the hemocompatibility of the NP (Supplementary Fig. 2).

5. Further emphasizing this point: Fig.S4 shows patchy deposits of fluorescent materials in the lung sections which looks exactly as microemboli, size of particles in suppl fig.17 does not fit with size bar, PDI is not systematically shown and size of panels and axes make them illegible.

Thank you for the comments. The red color in Supplementary Fig. S4 originates from the Cy5 fluorescent signals, which should not be confused with the microemboli with several clear evidences. (1) CLSM analysis showed that Cy5-labeled SiEV strongly co-localize with the endothelial marker CD31 (Fig. R1), rather than forming clump-like aggregates within the capillary lumen as observed for microemboli. (2) H&E-stained lung sections at 7 d post-injection of SiEV revealed no typical microthrombus-related pathologies, including perivascular erythrocyte extravasation, local tissue edema, or eosinophilic deposits in the capillary lumen (Supplementary Fig. 31). (3) The size of smaller fluorescent foci in Fig. 2g and Supplementary Fig. 4 was only 1-3 μm, which was significantly smaller than microemboli (typically > 20 μm). Thus, these evidences fully resolve the Reviewer's concerns regarding morphological resemblance to microthrombi.

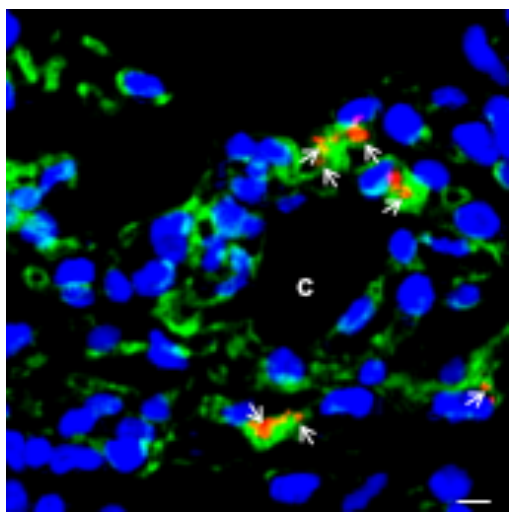

Fig. R1. Merged CLSM image of pulmonary tissue section. C: capillary lumen; green: ECs; red: SiEV; white arrows: co-localization of SiEV and ECs. Scale bar = 5 μm.

The particle size observed under TEM was consistent with the scale bar (50 nm). To further clarify the consistency, 20 NPs were randomly selected from TEM images of all NPs and manually measured with ImageJ. The results agree well with the size data obtained from DLS, validating our TEM analysis and the consistency of scale bars. Meanwhile, the PDI of all SiEV NPs was also provided in the revised manuscript (< 0.2), indicating narrow size distributions.

The new results, including size distribution from TEM and PDI, were incorporated as Supplementary Fig. 18 (Page S41). The following discussion was incorporated to highlight the consistency of size measurement (Page 17, highlighted):

> "Both TEM and DLS analyses confirmed the well-defined and uniform NPs with different sizes, which showed negative surface charges ~ -25 mV (Supplementary Figs. 18, 19, and Supplementary Table 2)."

6. The hypothetical scenario how this delivery system may work is hanging in the air. Measuring plasma effects on NP must be compared with whole blood, under static conditions and hydrodynamic stress in terms of agglutination, stability, damage and dissociation. How fast does NP bind to RBC? How many particles added and bound per RBC? How the kinetics of attachment and detachment look like? All these questions, that can be at least initially addressed in vitro, are vitally relevant to the real attempts to figure out what does take place in vivo, namely how fast and where NP stick to and detach from RBCs after injection.

We appreciate the suggestions from the Reviewer. The interactions between SiEV and RBCs have been thoroughly studied in our manuscript. The interactions between SiEV and RBCs were either performed *in vitro* with purified RBCs (Fig. 2b, Supplementary Fig. 2, Fig. 3i, Supplementary Fig. 12a, and Figs. 4b, 4c) or even directly studied *in vivo* (i.e., blood sampling after *i.v.* injection of SiEV or SiEV-RBC complexes, Supplementary Fig. 5, Figs. 3b-3e, Supplementary Fig. 10, Supplementary Fig. 12b, Figs 4d-4g, and Supplementary Fig. 16). Meanwhile, the hemocompatibility of SiEV has been well supported with new experiments in the 1st round of revision, including the hemagglutination assay and Hb release studies (Supplementary Fig. 2, response to Comment 4 by the same Reviewer).

In our initially submitted manuscript, the adsorption and desorption behaviors were studied after *i.v.* injection of SiEV or SiEV-RBC complexes (Figs. 3b-3e, Supplementary Fig. 10, Figs 4d-4g, and Supplementary Fig. 16). To further investigate the binding kinetics, the binding was studied under both *in vitro* and *in vivo* conditions. Flow cytometric analysis suggested significant binding interactions under static, *in vitro* mixing conditions, with gradually more bound SiEV as the extension of incubation time. In contrast, no obvious fluorescence signal was detected on the RBCs at 10 min after *i.v.* injection of SiEV, which was mainly attributed

to the complex *in vivo* environment, such as the blood flow and the formation of protein corona. Nevertheless, the binding became obvious after 30-min incubation/injection under both *in vitro* and *in vivo* conditions, supporting the *in situ* RBC hitchhiking mechanism.

Based on the size and density of SiEV, we have calculated that the *in vitro* complexation was done at SiEV feeding of ~ 8,000 NPs per RBC. After incubation, the RBC-SiEV complexes were separated, lysed, and quantified, and the fluorescent intensity accounted for 71% of that from Cy5-labeled SiEV before RBC incubation. After calculation, there are roughly 5,700 NPs bound to RBC *in vitro*. Meanwhile, the SiEV feeding ratio was much lower *in vivo*, which was determined to be ~ 600 NPs per RBC after *i.v.* injection. Because tracking free NPs is challenging *in vivo*, it is difficult to quantify the bound numbers *in vivo* at this stage.

The new experimental results on adsorption kinetics were added as Supplementary Fig. 12 (Page S35), and the calculation of adsorbed number of SiEV has been updated in the *Experimental Procedures* section in Supplementary Information (Page S12-S13, highlighted). The discussion on the binding was updated accordingly (Page 10, highlighted):

> "In addition, the adsorption of SiEV onto RBC surface was further studied under both static, *in vitro* conditions and dynamic, *in vivo* conditions. The results showed the rapid adsorption of SiEV after ~ 10 min incubation, with more NPs adsorbed as the increase in incubation time (Supplementary Fig. 12). After 1-h incubation, it was calculated that roughly ~ 5,700 SiEV were adsorbed onto the RBC surface. Meanwhile, the adsorption became obvious at 30 min post injection, suggesting slower adsorption kinetics *in vivo* that was attributed to the dynamic blood flow and complex physiological environment."

7. The glimpses of kinetic data provided in the current version, to a great surprise imply that NP loading/detachment to/from RBCs takes a relatively long time, namely hours. Where does this happen? No convincing data prove the transfer in the lungs or any other organ. Authors repetitively state that NP detachment in the pulmonary capillaries is caused by high shear stress. However, a pulmonologist would not agree, because blood flow in the pulmonary vasculature is the slowest and least shear stressed in the body. Peripheral, splenic, cerebral and especially myocardial vasculature represent sites of much higher shear stress.

Thank you for the comments. From our response to the last question, we have shown that the binding between SiEV and RBC is rapid (within 30 min), but the detachment and pulmonary accumulation indeed take a relatively long time (~ 12 h). The desorption of each individual NP was correlated with various factors, including vascular size and geometry as well as the attachment site of NPs on RBCs, which contributed to the different desorption kinetics. For instance, literature reports suggested that NPs bound to the outer layer of RBCs may detach more rapidly than those attached to the central groove (*ACS Nano*, **2013**, *7*, 11129).

Meanwhile, we would also like to note that the translocation of SiEV from RBC complexes to endothelial cells has been addressed with supporting data in our earlier response (i.e., response to Comment 4 by the same Reviewer in both rounds). One of the most reliable experiments, the dual-labeling of RBC-SiEV complexes through either fluorescence or radioisotope, confirmed the gradual transfer of SiEV from RBCs to lung tissues (Figs. 4f, 4g, and Supplementary Fig. 16). Moreover, the dissociation of RBC-SiEV complexes was directly visualized through fluorescence analysis of pulmonary tissue sections (Figs. 4d, 4e).

The shear stress in pulmonary capillaries was introduced by the small diameter, the tortuous geometric architecture, the "sheet flow" (rather than tubular flow), and the dynamic shape distortion of RBCs, not necessarily from the stress from blood flows. The shear-dependent desorption has been previously verified and discussed in several erythrocyte hitchhiking literatures (e.g., *Exp. Biol. Med.*, **2007**, *232*, 958, *Sci. Adv.*, **2019**, *5,* eaax9250, and *Proc. Natl. Acad. Sci. U.S.A.*, **2020**, *117*, 17727). Since the RBC is slightly larger than the average diameter of pulmonary capillaries, the friction forces from endothelial wall and the cell-cell interactions likely contribute to the desorption process.

We have further clarified the shear stress in pulmonary capillaries in the revised manuscript (Page 13-14, highlighted):

> "Since RBC is slightly larger than the average diameter of pulmonary capillaries, the friction forces from endothelial wall and the cell-cell interactions likely contribute to the desorption process."

8. From the previous review: The daunting challenges of interpretation optical signal is clearly illustrated by problem of blood: RBC have so strong signal of Hb that it dwarfs or distorts any optical probe. Only radioisotopes afford the needed quality of objective, precise and accurate data. To study hitchhiking you must have RBC labeled by 51Cr and cargo NP labeled by 125Iodine, 111In or other appropriate isotope that can de clearly and accurately discerned from RBC label. Only dual-labeling approach, described in RBC-HH studies allows to distinguish the transfer from RBC to the vascular cells from mechanical, charge or other non-specific RETENTION or UPTAKE of the whole RBC-cargo complex in the microvasculature downstream injection site. This is especially important in the RBC-HH studies, because this mechanism of delivery is prone to these non-specific features. Authors mention in passing that they failed to carry experiments using dual isotope labeled RBC/NP. This is too bad, because optical probes fail the authors to prove their hypotheses of local in vivo hitchhiking on RBC, followed by swing from RBC to endothelium.

We appreciate the comments from the Reviewer. We are afraid the Reviewer was reading the wrong version of the manuscript. We have shown conducted the dual-labeling using radioisotope in the 2nd round of revision, and the results confirmed the translocation of SiEV from RBC to endothelium, as suggested by the Reviewer. The dual-labeling experiments, either through fluorescence or radioisotope tracing, have clearly suggested the gradual transfer of SiEV from plasma to pulmonary tissues, with the RBCs remained in systemic circulation with negligible signals in pulmonary tissues (Figs. 4f, 4g and Supplementary Fig. 16). This result indicated the translocation process, ruling out the possibility that the RBC-SiEV complexes were internalized together by pulmonary cells. The dual labeling results were included in our last two rounds of revisions (response to Comment 4 by the same Reviewer for both rounds).

9. The third sentence in second paragraph on page R5 is astonishingly revealing: In response to the comment that supernatant in figure 4 looks reddish while control is yellow, authors state that this optical artefact "most likely originated from the color distortion when we took photo" (Sic!). From the previous review: "Interaction with RBC must include effects of NP on RBC in vitro and in vivo (hemolysis by complement and osmotic stress, agglutination, persistence in the circulation and uptake by spleen and other organs). Have you measured Hb in the medium after filtering RBC-NP? F5 shows full lysis with water – please show the calibration curve of Hb A and describe the incubation conditions". The super of control RBCs in

Supplement Fig. 4 look reddish; why it looks blue in the third tube? Authors responded to this by admitting in passing that these are likely artefacts of color distortion inflicted by photography, which makes the whole issue of inadequacy of optical methods vs precision of isotope detection even more important.

Thank you for the comments. We are confused by the request, because the Reviewer originally asked us to explain the "reddish" but not yellow supernatant in the 1st round of revision (Comment 4). In our view, the supernatant of the PBS control looks slightly yellow, which likely originated from the residual plasma. Since we did not see any red colors, we have to attribute the differences to color distortion in the response, and removed the photograph accordingly to avoid any potential confusion. Therefore, we think this is no longer an issue since the Reviewer agreed with us that "the control is yellow". Additionally, the blue color originated from the conjugated Cy5 dye, which has been clarified in the 1st round of revision (Comment 4). Meanwhile, we need to emphasize that we have clear supporting data from hemagglutination, Hb release, and hemolysis test to show the integrity of RBCs after incubation with SiEV (Supplementary Fig. 2).

Meanwhile, we do agree with the Reviewer that the radioisotope experiment is important. It was presented in our 2nd round of revision (Figs. 4f, 4g, and Supplementary Fig. 8). The results were highly consistent with those obtained from fluorescence method and ICP-OES method, thus validating the reliability of our experimental designs.

10. In response, authors showed nearly ideally linear calibration for optical density of Hb measured in a buffer, which is appropriate basal level of this part of the study. Now you must reproduce this calibration using serial dilutions of Hb in plasma, whole blood and in the homogenates of organs after IV injection of NP and compare with the graph of Hb dilutions of Hb doses added directly to these biological specimen.

We appreciate the suggestion from the Reviewer. We performed new experiments to quantify hemoglobin levels in plasma following *i.v.* administration of SiEV, as requested by the Reviewer. The results clearly suggested the negligible RBC damage *in vivo*, as the plasma level of hemoglobin is comparable with the that from control group.

The new result of hemoglobin quantification was incorporated as Supplementary Fig. 2 (Page S25). The following discussion was incorporated to further highlight the biosafety of SiEV (Page 7, highlighted):

> "Additionally, the plasma level of hemoglobin remained low after *i.v.* injection of the NPs (Supplementary Fig. 2), validating their *in vivo* hemocompatibility."

The comments cited below have not been addressed fully and adequately. This reviewer invites authors and editors to revisit these points if the main issues are solved.

We respectfully disagree with the Reviewer that the comments below "have not been addressed fully and adequately". We believe that we have properly addressed all comments from the Reviewer, either with detailed discussions, new reference citations, or with new experiments. Specifically, for the three comments below, we provided a table to clearly summarize our efforts to perform new experiments and incorporate additional discussions in response to the comments, questions, and requests from the Reviewer. The corresponding experimental results and discussions have been included in our response letter in the 1st round of revision.

	Responses	New Figures
11	*Key: no specific vascular marker due to erythrocyte hitchhiking*<br>(1) Literature support from other erythrocyte hitchhiking papers<br>(2) New experiments: comparison with antibody-NPs, *i.c.* injection<br>(3) Discussions: three solid evidences to justify "no specific marker"	Figs. 4h, 4i<br>Supplementary Figs. 9, 11, and 17
12	*Key: biomedical application is equally important to mechanistic studies*<br>(1) Lung-targeting exhibits significantly enhanced therapeutic outcomes<br>(2) Discussions on three reasons why we choose ALI	-
13	*Key: low incidence of unexpected leukocyte interactions*<br>(1) New experiments: NP distribution among various blood cells<br>(2) Discussions: low uptake by leukocytes and platelets	Supplementary Fig. 5

Below are some additional discussions besides the responses in the 1st round of revision:

11. The nanoparticles devised in this study are very interesting. If (it is an important "if") the phenomenon and its specificity will be proven as requested, the question of what the tentative mechanism of the reported "specific precise delivery into the pulmonary endothelium is needed. Which component of nanoparticles recognize specifically and precisely pulmonary endothelium? In which vascular segment? Which surface marker on endothelium represents the binding site for these NP provides the precision and selectivity of the delivery? A singular interpretation of the interesting results pertaining to the functional effects as the result of specific and precise delivery of the described particles to the pulmonary endothelium is NOT established. The observed results may represent the effects of interactions of the injected materials with numerous migratory and resident pulmonary constituencies.

Thank you for your comments on the lung-targeting mechanism. As the Reviewer already recognized at the beginning of the comments this round (Comment 1), the targeting of SiEV does not rely on any specific vascular markers. The comparison between *i.v.* and *i.c.* administration provides the most reliable support, where the different injection routes led to completely different biodistributions. Meanwhile, the comparison between SiEV, non-targeting SiENV, and antibody-modified NPs also validated the absence of any specific markers to facilitate the targeting of SiEV.

12. Authors embarked on the testing protective effects of this drug delivery system in animal models of human lung diseases. Scientifically speaking, this direction of the continuation of this research project needs to answer the questions above first. This is especially relevant to the studies of such sort based on measuring totally non-specific markers of non-specific pathological conditions such as oxidative burst driven and manifested by ROS, MDA, etc.

We believe that the complete experimental design is critical for our manuscript, from the heteropolypeptide design and structural modulation to the targeting mechanism studies and therapeutic demonstration, which draws attention from researchers from different fields, such as polymer chemistry, biomaterials, nano-science, and drug delivery.

Meanwhile, we agree with the Reviewer that the mechanistic studies are critical and have performed several new experiments to clarify the mechanism during manuscript revisions (e.g., comparison with antibodies, dual-labeling experiments, and adsorption kinetic studies).

13. Particles may engage diverse cells in blood and organs, including marginated leukocytes in the pulmonary vasculature, RES and other sites. It became known recently that interaction of

particles with leukocytes and other reactive components of host defense may lead to unexpected consequences, such as hitchhiking particles by blood cells headed to the CNS.

We appreciate the comments from the Reviewer. The strong interactions between SiEV and RBC have been verified by several experiments, including the strongest binding among 12 NPs (i.e., ~ 40 folds higher) (Fig. 2b), high adsorption efficiency (40% RBCs *in vitro* and 10% RBCs *in vivo*) (Supplementary Fig. 12), selectivity of RBC over other blood cells (94%) (Supplementary Fig. 5), and rapid adsorption kinetics (< 10 min *in vitro* and < 30 min *in vivo*) (Supplementary Fig. 12). The strong interactions suggested low incidence that the NPs participate in strong interactions with leukocytes.

Meanwhile, H&E staining of major organs revealed no pathological abnormalities on 7 d post injection of the NPs (Supplementary Fig. 31). Specifically, there was no evidence of inflammation or infiltration of immune cells, which are common signs of adverse immune responses.

RESPONSES TO REFEREES

(Reviewer's comments in black, our response in blue, and text added to the paper in magenta)

Reviewer 1 (Remarks to the Author)

In the rebuttal, authors admit that PMID 40668829 presents a highly promising and innovative approach. Indeed, the level of the heart uptake of the cardiotropic LNP/RNA determined quantitatively using isotopes and optical probes dwarfs known attempts of systemic (IV) delivery to the CMC in the myocardium. Further, this approach yields measuring blood level and distribution between blood plasma and cellular elements. PK and biodistribution data are missing in most publications including present paper. Instead of appraisal of the informative value of methodologies, authors seem concerned that "Incorporating this system would introduce a confounding variable". This reviewer categorically disagrees. Authors should discuss and cite PMID 40668829, missing in the manuscript. In the rebuttal it is mistakenly attributed to Kenjo. The glitch reveals inconsistencies in the way of engagement of "careful considerations".

Thank you for the comment. We would like to respectfully point out that the lung-targeting system in our study is distinct from that described in reference PMID 40668829 (i.e., *Proc. Natl. Acad. Sci. U. S. A.* **2025**, *122*, e2409266122). Our study describes the *in situ* RBC hitchhiking strategy following *i.v.* injection to achieve pulmonary delivery of NPs, which is in stark contrast to the cardiac delivery reported in the reference. The differences are substantial across various aspects: materials (polymer *vs* lipid), mouse models (normal *vs* $Apoe^{-/-}$), target organs (lung *vs* heart), and therapeutic modalities (nanoenzyme *vs* siRNA) are all different. Notably, we did not cite or discuss PMID 40668829 in either our original manuscript or our previous responses, as it pertains to a different line of research.

Additionally, we would like to clarify that the pharmacokinetics and biodistribution data have already been clearly presented in our manuscript (Figs. 2c, 2e, and Supplementary Fig. 5). These results collectively demonstrate the lung-targeting ability of SiEV.

To avoid any potential misunderstanding, we have carefully reviewed our manuscript to ensure that the distinctive features of our system are explicitly conveyed.

Reviewer #5 (Remarks to the Author)

The authors of this manuscript have addressed a number of the comments made by Reviewer #1, and their data is consistent with lung delivery via in situ RBC hitchhiking. I agree with the reviewer that this phenomenon is to be expected based on prior published work, but see the value in the description of a new chemical approach to achieve this and agree with the prior reviewer that the real contribution of this work lies

in understanding the NP-RBC interactions and how these influence the in vivo performance of the materials.

We thank the Reviewer for the positive comments on our revised manuscript.

1. The authors assert (correctly) that in situ RBC hitchhiking offers advantages over antibody-mediated RBC (or pulmonary) targeted NPs and pre-incubated RBC-hitchhikers. The authors currently do not contextualize this work within the state-of-the-art of chemical-based (particularly ionic liquid-based) in situ RBC hitchhiking. Therefore, the most exciting claim of this manuscript is not the discovery of a chemical-based in situ RBC hitchhiker but the more delicate structural findings.

We appreciate the comment from the Reviewer. We have expanded the Introduction section to include chemical-based strategies for *in situ* RBC hitchhiking, especially those employing ionic liquids. Meanwhile, we fully agree with the Reviewer that the delicate structural findings, especially the sequence-dependent RBC hitchhiking and lung targeting that stemm from the incorporation of β-branched amino acids, are the key highlight of our manuscript.

The following text was added in our revised manuscript (Page 3, highlighted), which gave us a more comprehensive introduction of the state-of-the-art of RBC hitchhiking:

> "In fact, recent studies demonstrated the use of ionic liquid coatings to alter the biodistribution of various NPs through *in situ* RBC hitchhiking."

2. I am not convinced that the mechanism is as straight-forward as hydrophobic interactions. I do think it's likely that hydrophobic interactions play a role, but based on the structural specificity of the author's findings, I wonder if other transporters within the RBC membrane are also implicated (like LAT1).

Thank you for your comments on the lung-targeting mechanism. We agree with the Reviewer that the RBC-SiEV interactions may include multiple non-covalent forces beyond hydrophobic interactions. Nevertheless, our additional studies suggested strong adhesion of SiEV to protein-free GUVs, which were employed to simulate RBC membrane lipids (Fig. 3h), indicating that hydrophobic interactions alone could drive relatively strong binding in the absence of proteinaceous transporters.

Specifically regarding LAT1 as suggested by the Reviewer, we pretreated RBCs with 2-amino-2-norbornanecarboxylic acid (BCH, a competitive inhibitor of LAT1) prior to incubating with SiEV. Compared to untreated RBCs, the BCH treatment did not alter the adsorption of SiEV by RBCs, suggesting that LAT1 is not a major contributor to the interaction between SiEV and RBCs under our experimental conditions.

Taken together, our current data support that hydrophobic interactions play a dominant role in the *in situ* RBC hitchhiking mechanism. We cannot, however, exhaustively rule out the potential involvement of other transporters or membrane components.

The BCH inhibition results were incorporated as Supplementary Fig. 18 (Page S19). The following discussion has been added to the revised manuscript to clarify the complexation mechanism between RBC and SiEV (Page 10-11, highlighted):

> "It has to be noted that the RBC-SiEV complexation might involve multiple non-covalent forces beyond the hydrophobic interactions between PVal segments and lipid bilayers. While the specific receptor-mediated association was not observed at the moment (Supplementary Fig. 18), the Coulomb interactions and the hydrogen bonding interactions may further facilitate the stable anchoring of SiEV onto RBC membranes and contribute to the overall binding affinity."

3. I am curious about the decision to evaluate the RBC hitchhiking in vitro with isolated RBCs vs whole blood. Do the authors in vitro data better match the in vivo data when the NPs are first evaluated with whole blood instead of RBCs? This to me seems more rigorous than pre-incubating with plasma since it captures the competing dynamics. What percentage of NPs are ending up on the RBCs? (vs the number of RBCs with NPs on them) Is this consistent with the ~37% lung delivery they see?

We appreciate the comments from the Reviewer. We agree with the Reviewer that the experiments with the whole blood would offer a more comprehensive view, where the competition with other circulating components is present. Nevertheless, as the first screening experiment to identify promising candidate NPs from 10+ polypeptide-based NPs, we would like to simplify the experimental procedures to rapidly obtain a clean and unambiguous readout. Therefore, isolated RBCs offer straightforward information on RBC affinity. Given the overwhelming numerical predominance of RBCs over all other blood cells, we believe that the *in vitro* data with purified RBCs are physiologically relevant and informative, even without considering potential competing effects.

Following the constructive suggestion from the Reviewer, the whole blood was isolated and incubated with SiEV or SiENV for 1 h at 37 ºC, and the supernatant plasma was isolated for the quantification of unbound NPs. The results suggested that 71.7% and 4.8% NPs were ending up on the blood cells for SiEV and SiENV, respectively. The results obtained from the whole blood were consistent with those from isolated RBCs and the *in vivo* data. However, we note that this whole-blood approach is somewhat limited without the use of specific antibodies, as it does not permit the resolution of NP binding specifically to RBCs *versus* other blood cells. Interestingly, the *in vivo* flow cytometric results suggested that 94% of the NPs were associated with RBCs, leaving only a minor fraction bound to other blood cells (Supplementary Fig. 7). Although this observation was made retrospectively, it strongly suggests that the inclusion of other cellular components in whole blood would have introduced minimal perturbation to the RBC-specific interactions we aimed to measure.

Finally, the binding percentage of SiEV on RBC was much higher than the ID% in pulmonary tissues, which was presumably attributed to the accumulation of RBC-SiEV complexes in other organs and the release of SiEV to non-pulmonary tissues.

The new whole-blood experiment was included as Supplementary Fig. 15 (Page S16). The following discussion was incorporated in the revised manuscript to include the whole-blood result for comparison (Page 9, highlighted):

> "After 1-h static incubation with the whole blood, while only 4.8% of SiENV bound to RBCs, 71.7% of SiEV was associated with RBCs, corresponding to ~ 5,700 SiEV adsorbed onto the RBC surface (Supplementary Fig. 15), indicating strong RBC-SiEV interactions even in the presence of other circulating blood cells."

4. I am convinced by the isotope experiments that the NPs are leaving the RBCs at the lungs, but the slow adsorption and desorption kinetics is inconsistent and confusing with this phenomenon and is still unresolved. The SEM image of the RBC is also inconsistent with this, and I'm not sure whether I'm looking at a red blood cell that is post-hitchhiking or not. Could the authors use elemental mapping to establish if the features on the RBCs are NPs? An additional 50 nm scale bar in the zoomed in images would also help with this.

Thank you for the insightful suggestion. The RBC adsorption reached the peak at 1 h (Supplementary Fig. 15) while the desorption was maximized at 12 h post injection (Fig. 4g and Supplementary Fig. 20), both are consistent with the pulmonary accumulation kinetics (12 h, Fig. 2h). The pulmonary accumulation of our SiEV system is substantially longer than that observed in conventional, pre-assembled RBC hitchhiking systems. We believe that this discrepancy arises from two aspects: (1) the SiEV system starts with free NPs that require *in situ* complexation with RBCs, which is different with the injection of pre-formed RBC complexes in conventional setups. (2) SiEV, capable of *in situ* RBC hitchhiking, might have stronger associations with RBCs that alters the disassembly kinetics. Therefore, although we also observe the shear-force responsiveness on NP release that resembled that of conventional RBC hitchhiking, the *in vivo* association and dissociation processes could be more complex that deserve separate in-depth studies. In fact, we have a parallel, on-going study to elucidate the impact of flow rate, vessel diameter, etc. on the disassembly of RBC-SiEV complexes. The results will be included in our future publications.

Following the suggestion from the Reviewer, we performed elemental mapping (SEM-EDS) to confirm the presence of SiEV on RBC membranes. The results revealed the presence of Si element on the RBC membranes, indicating the attachment of the NPs. Additionally, a 50-nm scale ruler was added to the magnified SEM images to facilitate the accurate assessment of the size and distribution of the NPs.

The new result was incorporated as Supplementary Fig. 12 (Page S13). The following discussion has been added to prove that SiEV was successfully attached to RBCs. (Page 8, highlighted):

> "Further energy-dispersive X-ray spectroscopy (EDS) analysis confirmed the presence of Si-based NPs on the surface of RBCs (Supplementary Fig. 12)."

5. I would recommend couching the biocompatibility experiments as early stage and preliminary. This is the first report of the material, so I do think it is unnecessary to include long term, repeat dose safety studies, but these results should be presented as they are - a first run, single dose study that indicates promising potential biocompatibility.

We thank the Reviewer for the comment. we have revised the manuscript to clearly indicate that these data are derived from a single-dose study and represent an initial assessment of biocompatibility. Meanwhile, we also avoid overstating the conclusions and now explicitly note that long-term and repeat-dose safety studies are warranted in future work.

The following text has been modified in the manuscript (Page 18-19, highlighted):

> "To evaluate the systemic toxicity of SiEV, healthy mice were intravenously injected with a single dose of the NPs and sacrificed."
>
> "Furthermore, we will conduct more comprehensive long-term and repeat-dose safety evaluations in future work."
